# Second-Generation Synthesis and Analytical Application of TBBA for Chiral Analysis of Amino Acids and Oligopeptides by ^1^H and ^19^F NMR Spectroscopy

**DOI:** 10.1021/acs.joc.5c02693

**Published:** 2026-01-28

**Authors:** David Profous, Naděžda Cankařová, Jakob Enengl, Sarin Soji, Uwe Rinner, Petr Jurečka, Petr Cankař

**Affiliations:** † Department of Organic Chemistry, Faculty of Science, 48207 Palacký University Olomouc, 17. listopadu 1192/12, 779 00 Olomouc, Czech Republic; ‡ Institute of Applied Chemistry, 92703 IMC Krems University of Applied Sciences, Piaristengasse 1, 3500 Krems, Austria; § Department of Physical Chemistry, Faculty of Science, Palacký University Olomouc, 17. listopadu 1192/12, 779 00 Olomouc, Czech Republic

## Abstract

A concise and efficient second-generation synthesis of 2-(2-(trifluoromethyl)-1*H*-benzo­[*d*]­imidazol-1-yl)­benzoic acid (TBBA) has been developed. The synthesis affording enantiomerically pure TBBA atropisomers was significantly streamlined through optical resolution by diastereomeric salt formation, enabling preparation on a 32 mmol scale. The applicability of TBBA as a chiral derivatizing agent in the solid-phase synthesis of amino acid derivatives was demonstrated, allowing determination of the absolute configuration and optical purity by ^1^H and ^19^F NMR spectroscopy. Furthermore, ^19^F NMR analyses were successfully carried out on a low-field benchtop NMR spectrometer, including samples dissolved in nondeuterated solvents.

## Graphical abstract

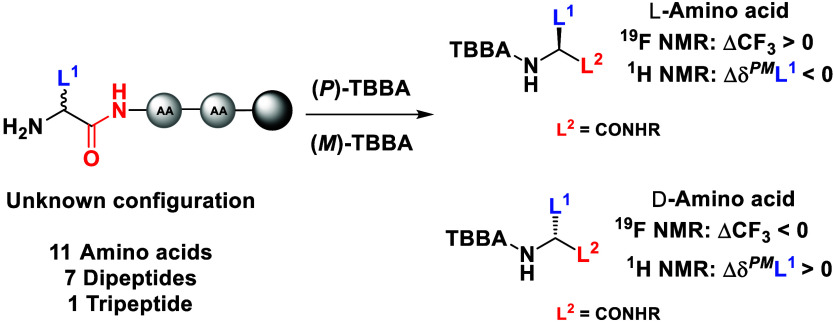

## Introduction

The stereochemical integrity of amino acids and peptides plays a crucial role in determining their biological activity and function. The discovery of solid-phase peptide synthesis^1^ (SPPS) by Robert Bruce Merrifield in 1963 fundamentally transformed peptide chemistry, enabling the efficient and reproducible synthesis of peptides with high purity. Despite significant advancements in SPPS, base- or reagent-induced racemization remains a persistent challenge,^2,3^ often leading to the formation of unwanted stereoisomers that adversely affect the biological properties of the final peptide.^4–6^ When a protected amino acid, particularly those bearing nucleophilic side chains such as histidine, cysteine, or serine, is activated by a coupling reagent, an intermediate prone to racemization is formed, resulting in α-carbon epimerization during peptide bond formation.^7^ These racemic products are difficult to remove with routine purification methods, which complicates achieving the desired stereochemical purity.

To accurately evaluate and control the stereochemical outcome in synthetic amino acid derivatives, it is essential to develop reliable analytical methods for determining both optical purity and absolute configuration. For this purpose, techniques such as chiral high-performance liquid chromatography (HPLC),^8,9^ mass spectrometry,^10,11^ circular dichroism,^12^ capillary electrophoresis,^13^ X-ray crystallography,^14^ and nuclear magnetic resonance (NMR) spectroscopy can be employed.^15–17^

The most frequently employed technique is chiral HPLC, which is widely regarded as the gold standard for the determination of enantiomeric purity. In general, chromatographic separations require authentic analytical standards for the unequivocal assessment of the quality and quantity of analyzed samples. These standards must often be synthesized when they are not commercially available. The structural similarity of derivatives prepared from amino acids can present a significant challenge in developing efficient and selective separation methods. Analytical difficulties are further exacerbated when employing conventional ultraviolet (UV) detection, particularly if the amino acid, oligopeptide, or small polypeptide derivatives contain only weakly UV-active chromophores.

The application of chiral ^19^F-labeled probes in ^19^F NMR spectroscopy provides an attractive alternative for assessing the chemical and stereochemical purity of derivatives prepared from amino acids by SPPS. The advantages of ^19^F NMR spectroscopy include exceptional sensitivity to the electronic environment, allowing the detection of subtle structural or electronic variations owing to its broad chemical shift range and excellent signal-to-noise ratio even at low concentrations.^18–22^ The absence of endogenous fluorine nuclei in most analytes and matrices is an additional advantage, particularly beneficial in studies of biological molecules.^23,24^

We have recently reported a method for determining the absolute configuration of primary amines and secondary alcohols using the axially chiral derivatizing agent (CDA) 2-(2-(trifluoromethyl)-1*H*-benzo­[*d*]­imidazol-1-yl)­benzoic acid (TBBA) by ^19^F NMR spectroscopy.^25^ Based on these findings, we sought to expand the application scope of TBBA for the assignment of absolute configuration and assessment of stereochemical purity of amino acid derivatives synthesized by SPPS. We envision that this approach will enable verification of the *N*-terminal amino acid configuration by ^19^F NMR and, optionally, by ^1^H NMR^26,27^ for corroboration. Since the ^19^F NMR spectra of TBBA derivatives typically exhibit sharp singlets corresponding to the trifluoromethyl group, the method allows straightforward evaluation of stereochemical purity, which is often complicated in ^1^H NMR spectra by overlapping resonances from structurally similar moieties.

We also demonstrate the preliminary applicability of this method using practical low-field benchtop NMR spectrometers, which do not require cryogenic cooling and are thus well suited for routine laboratory use. An additional advantage of benchtop NMR instrumentation is the feasibility of employing nondeuterated solvents in ^19^F NMR experiments.

Finally, we report a second-generation synthesis of enantiomerically pure, axially chiral TBBA via optical resolution of atropisomers through diastereomeric salt formation. This protocol significantly simplifies the previously described method, which relied on time-consuming and labor-intensive chromatographic separation of TBBA diastereomers.

## Results and Discussion

The second-generation synthesis of TBBA was carried out according to a previously reported procedure,^26^ with refined reaction conditions and optimized workup protocols, scaled to 32 mmol batch. The sequence begins with a nucleophilic aromatic substitution, in which benzoic acid **2** is obtained from anthranilic acid and fluorobenzene **1**. Subsequent catalytic hydrogenation furnishes diamine **3**, which is then cyclized to afford racemic TBBA (***rac*** **-** **4**). The resulting racemate was subjected to crystallization to ensure homogeneity and purity prior to optical resolution of the atropisomers via diastereomeric salt formation (Scheme 1).

**Scheme 1:**
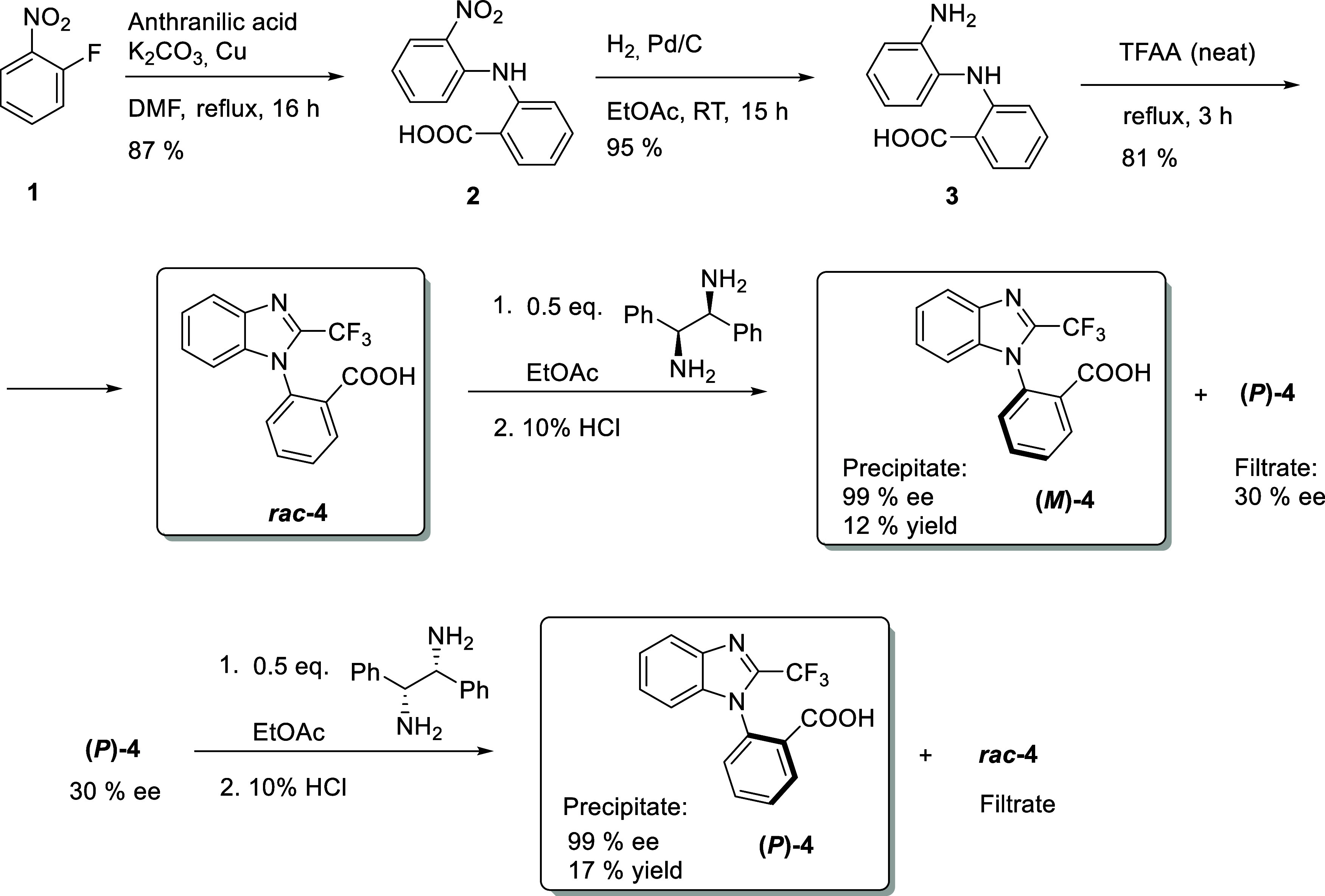
Synthesis of Racemic TBBA and Its Optical Resolution *via* Diastereomeric Salt Formation

Optical resolution was achieved by crystallization of diastereoisomeric salts formed between TBBA and 1,2-diphenylethyldiamine, identified as an efficient chiral resolving agent for this transformation. Crystallization was performed using 0.5 equiv of the diamine in ethyl acetate under vigorous stirring. A single resolution cycle, consisting of two successive crystallizations with the (*S*,*S*)- and (*R*,*R*)-enantiomers of the diamine, afforded 12% of enantiomerically pure (*M*)-TBBA (**(** ***M*** **)-4**) and 17% of enantiomerically pure (*P*)-TBBA (**(*P*)-4**). Both the starting amine and the remaining nearly racemic TBBA were recovered and reused in subsequent cycles. Compared to the previously reported method, this resolution offers several advantages, including larger scale, shorter processing time, lower cost, operational simplicity, and minimal waste generation.

The synthesis of TBBA-peptide derivatives was carried out using standard solid-phase methodology (Scheme 2).^28^ Initially, the Rink amide resin **5** was treated with 50% piperidine in DMF to remove the Fmoc protecting group. Subsequent acylation with Fmoc-protected amino acids was performed under standard coupling conditions.^28^ These two steps were repeated until the desired oligopeptide sequence **6** was assembled. Final Fmoc deprotection of the resin-bound peptide enabled acylation with TBBA to afford compound **7**, which was then cleaved from the resin to yield compound **8**. Notably, at each stage of the synthesis, resin-bound intermediates **6** (*n* = 1–3) could, in principle, be acylated with TBBA to afford compounds **7** (*n* = 1–3). After cleavage from the resin, these derivatives could be conveniently monitored by ^19^F and/or ^1^H NMR spectroscopy.

**Scheme 2:**
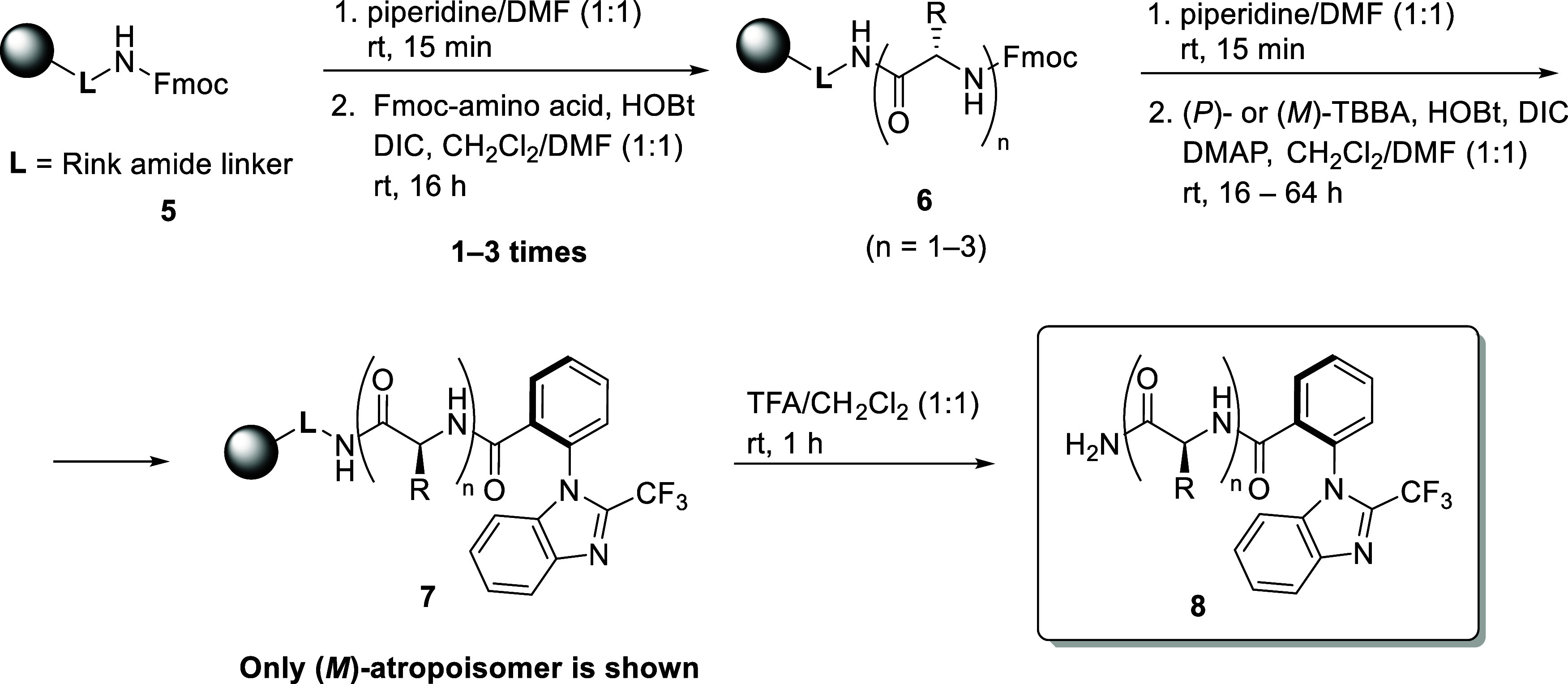
Preparation of TBBA Oligopeptide Derivatives *via* SPPS

Using this approach, we successfully synthesized 11 TBBA diastereomeric pairs of proteinogenic amino acid derivatives, six dipeptide pairs, and one tripeptide pair.

Our previously reported general conformational models (Figure 1A,C) predict the absolute configuration of chiral TBBA derivatives, such as carboxylic esters and amides, using ^1^H and ^19^F NMR spectroscopy when the samples are dissolved in CDCl_3_.^25,26^ However, CDCl_3_ is not suitable for derivatives with amide functionalities prepared from amino acids because of their limited solubility.

**Figure 1:**
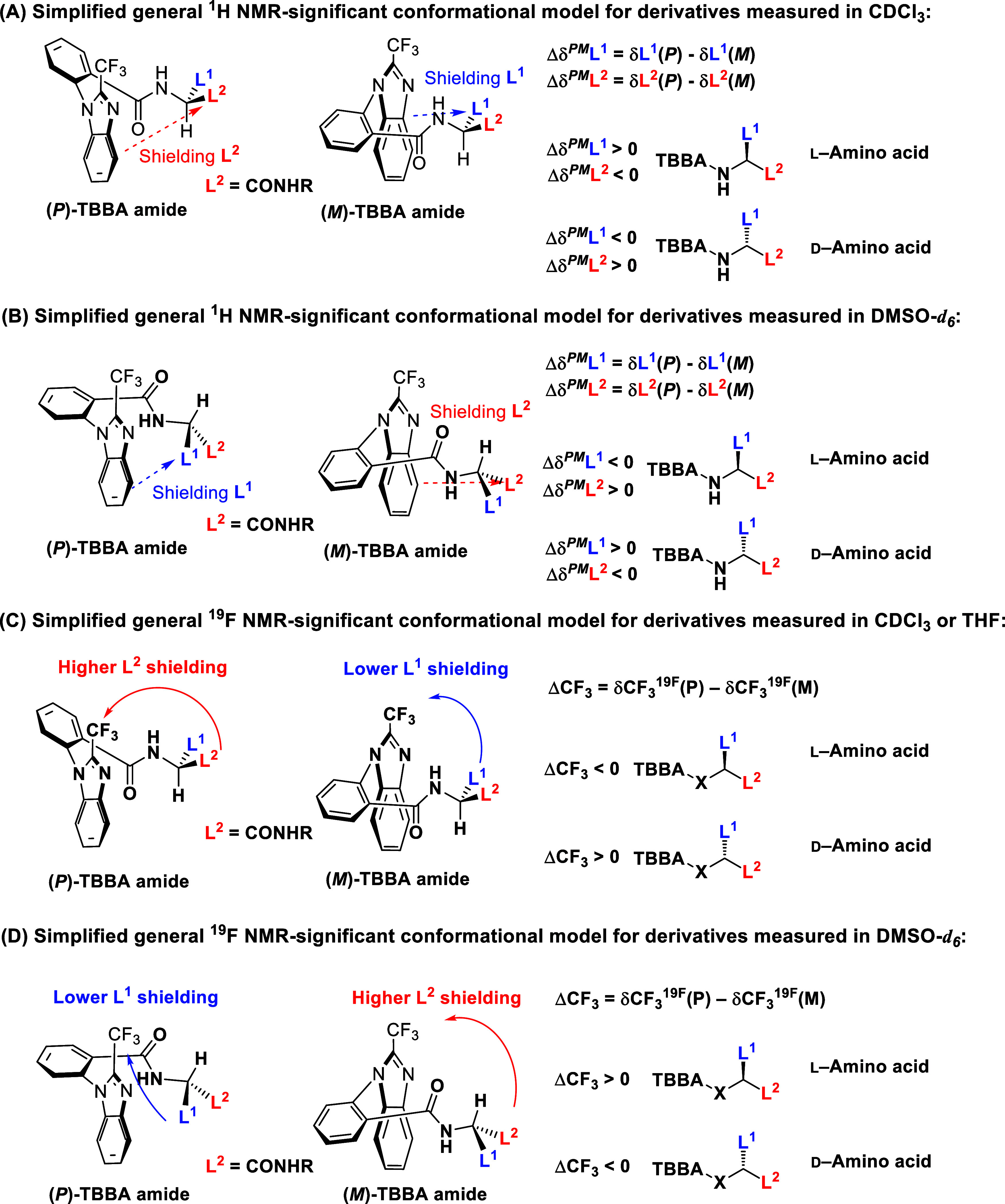
Simplified general conformational models: (A) For ^1^H NMR in CDCl_3_; (B) ^1^H NMR in DMSO-*d*_6_; (C) for ^19^F NMR in CDCl_3_ or THF; and (D) for ^19^F NMR in DMSO-*d*_6_. The amide group is a strongly shielding substituent, as previously reported.^25^

Due to the poor solubility of most amino acid derivatives in CDCl_3_, the solvent was subsequently changed to DMSO-*d*_6_. In this solvent, the Δδ^PM^ value observed for the most relevant methyl group of alanine derivative **9** in the ^1^H NMR spectrum was −0.24 ppm, while the ΔCF_3_ value in the ^19^F NMR spectrum was +0.18 ppm (Figure 3). Both values deviated significantly from the trends predicted by the general conformational models (Figure 1A,C)^25,26^ indicating a pronounced solvent effect on the NMR-significant conformation.

We hypothesize that the increased polarity of DMSO-*d*_6_ alters the conformational equilibrium, favoring an alternative intramolecular orientation. Specifically, the carbonyl oxygen of the amide group becomes oriented toward the trifluoromethyl substituent.^29,25^ This reorganization of substituent orientations consequently leads to an inversion of the signs of Δδ^PM^ and ΔCF_3_ (Figure 1B,D). These findings support the notion that TBBA-derived conformational models are highly sensitive to solvent polarity. To verify this hypothesis, we subsequently performed quantum mechanical (QM) calculations.

The QM modeling further illustrated the solvent-dependent conformational changes. We selected the (*P*)-atropisomer of the smallest compound **9** as a model system and searched for its lowest-energy conformer in both DMSO and CHCl_3_ using conformational sampling followed by QM optimizations and COSMO-RS solvation calculations (see Conformational Sampling and DFT Calculations in the Supporting Information).^30–42^ The lowest-energy structures identified were indeed different in the two solvents (Figure S1). In CHCl_3_, the NH group was oriented toward the CF_3_ group, as shown in Figure 1A,C, whereas in DMSO, the carbonyl oxygen of the amide group was positioned closer to the CF_3_ substituent, as depicted in Figure 1B,D. Furthermore, the intramolecular hydrogen bond formed in CHCl_3_ between the amide NH hydrogen and the carbonyl oxygen was disrupted in DMSO by competitive intermolecular hydrogen bonding with the solvent (Figure S1). Thus, our QM calculations support the expected conformational change upon switching the solvent from nonpolar CDCl_3_ to the more polar DMSO-*d*_6_.

A similar sampling for the (*M*)-atropisomer is less conclusive, as several conformations fall within a narrow energy window. It should be noted that accuracy of the QM calculations is limited mainly by the solvent model (See also Conformational Sampling and DFT Calculations in the Supporting Information) and may be insufficient to reliably rank conformers that are very close in energy.

Furthermore, we evaluated the separation of signals in the ^19^F NMR spectrum of alanine derivative **9** when both diastereomers were present in a mixture. For this purpose, a diastereomeric mixture was prepared from racemic TBBA and (d)-Ala (Figure 2).

**Figure 2:**
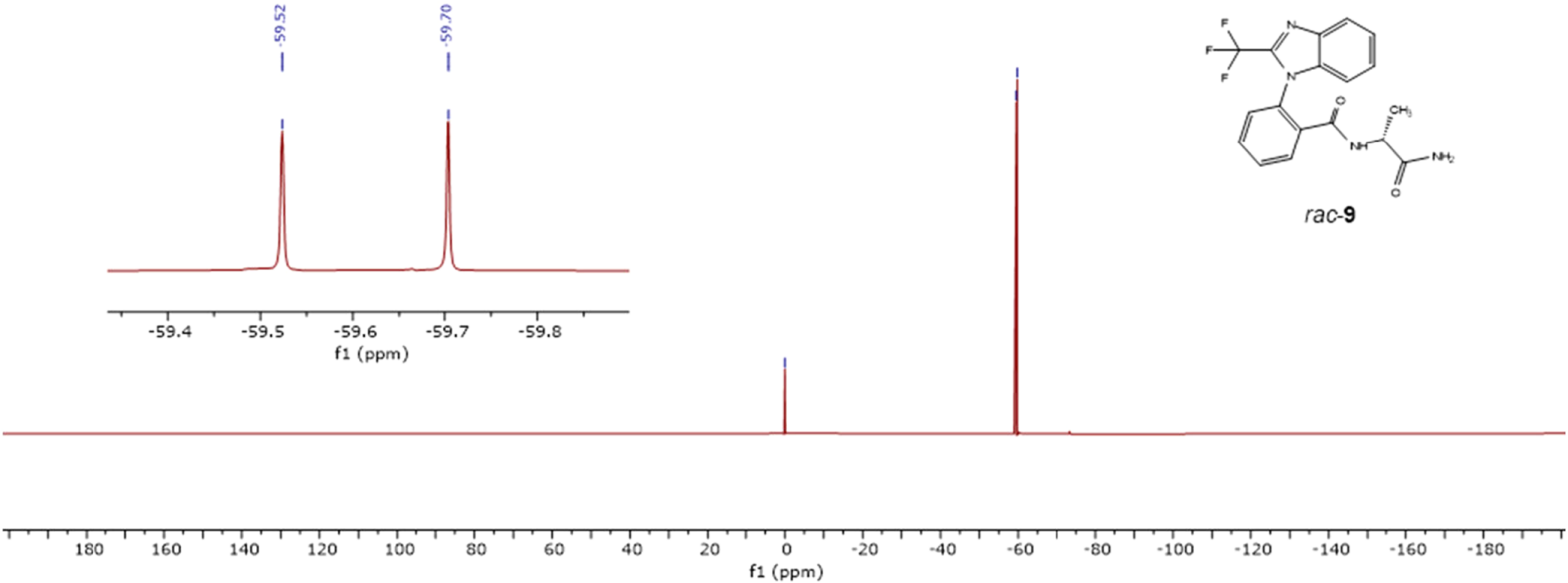
^19^F NMR spectrum of a diastereomeric mixture of **(** ***P*** **)-9** and **(** ***M*** **)-9**, prepared from (d)-Ala and recorded in DMSO-*d*_6_. CFCl_3_ was used as an internal standard.

The observed solvent-dependent conformational equilibrium prompted us to examine whether this trend extends to other amino acids and oligopeptides. Valine derivatives **10** exhibited a Δδ^PM^ of −0.16 ppm for the isopropyl CH group and a ΔCF_3_ of −0.14 ppm. Notably, the ΔCF_3_ value was inverted relative to alanine, whereas the Δδ^PM^ remained negative. Leucine **11** and methionine **12** displayed behavior similar to that of alanine **9**. For leucine **11**, the Δδ^PM^ was −0.22 ppm for the side-chain CH_2_ group, while methionine **12** showed a Δδ^PM^ of −0.19 ppm for the β-CH_2_ group. Interestingly, both compounds exhibited similar ΔCF_3_ values of +0.05 and +0.07 ppm, respectively.

Subsequently, the focus was shifted to aromatic amino acids. Structurally similar phenylalanine **13** and tyrosine **14** exhibited Δδ^PM^ values of −0.15 ppm and −0.14 ppm for the CH_2_ group, respectively, comparable to those observed for the aliphatic amino acids. The ΔCF_3_ values were +0.09 ppm for phenylalanine **13** and +0.02 ppm for tyrosine **14**. Nitrogen-containing heterocyclic amino acids were also examined. Tryptophan **15** followed the previously observed trend, exhibiting a Δδ^PM^ of −0.19 ppm for the CH_2_ group and a ΔCF_3_ of +0.05 ppm. In contrast, histidine **16** displayed a pronounced decrease in Δδ^PM^ to −0.03 ppm for the CH_2_ group, accompanied by an increase in ΔCF_3_ to +0.1 ppm.

Molecules containing a hydroxymethyl group can be problematic,^29^ as the hydroxyl moiety may form an intramolecular hydrogen bond with the TBBA amide carbonyl, thereby shifting the conformational equilibrium away from the general model. Serine **17** exhibited a Δδ^PM^ of −0.27 ppm for the CH_2_ group, the second highest among the derivatives studied, and a ΔCF_3_ of +0.08 ppm.

The highest Δδ^PM^ value for the CH_2_ group was observed for aspartic acid methyl ester **18** (−0.33 ppm). Asparagine **19** exhibited a lower Δδ^PM^ of −0.19 ppm. Interestingly, the ΔCF_3_ values for both amino acids were similar, measuring +0.14 ppm for aspartic acid **18** and +0.12 ppm for asparagine **19**.

Overall, the Δδ^PM^ values obtained in DMSO-*d*_6_ (Figure 3) were consistent with the proposed general conformational model for this solvent (Figure 1B). The ΔCF_3_ values were likewise consistent with the model in Figure 1D, with a single exception observed for valine derivative **10**, which showed a negative difference. However, the concurrently obtained Δδ^PM^ value from ^1^H NMR spectroscopy supported this apparent inconsistency. The absolute ΔCF_3_ differences ranged from 0.02 to 0.18 ppm, with only the value observed for tyrosine **14** being too small for reliable resolution on a benchtop NMR spectrometer operating at 80 MHz. A minimum difference of approximately 4 Hz (0.05 ppm) is required to achieve complete signal separation necessary for determining the enantio- or diastereomeric purity of stereoisomeric mixtures.

**Figure 3:**
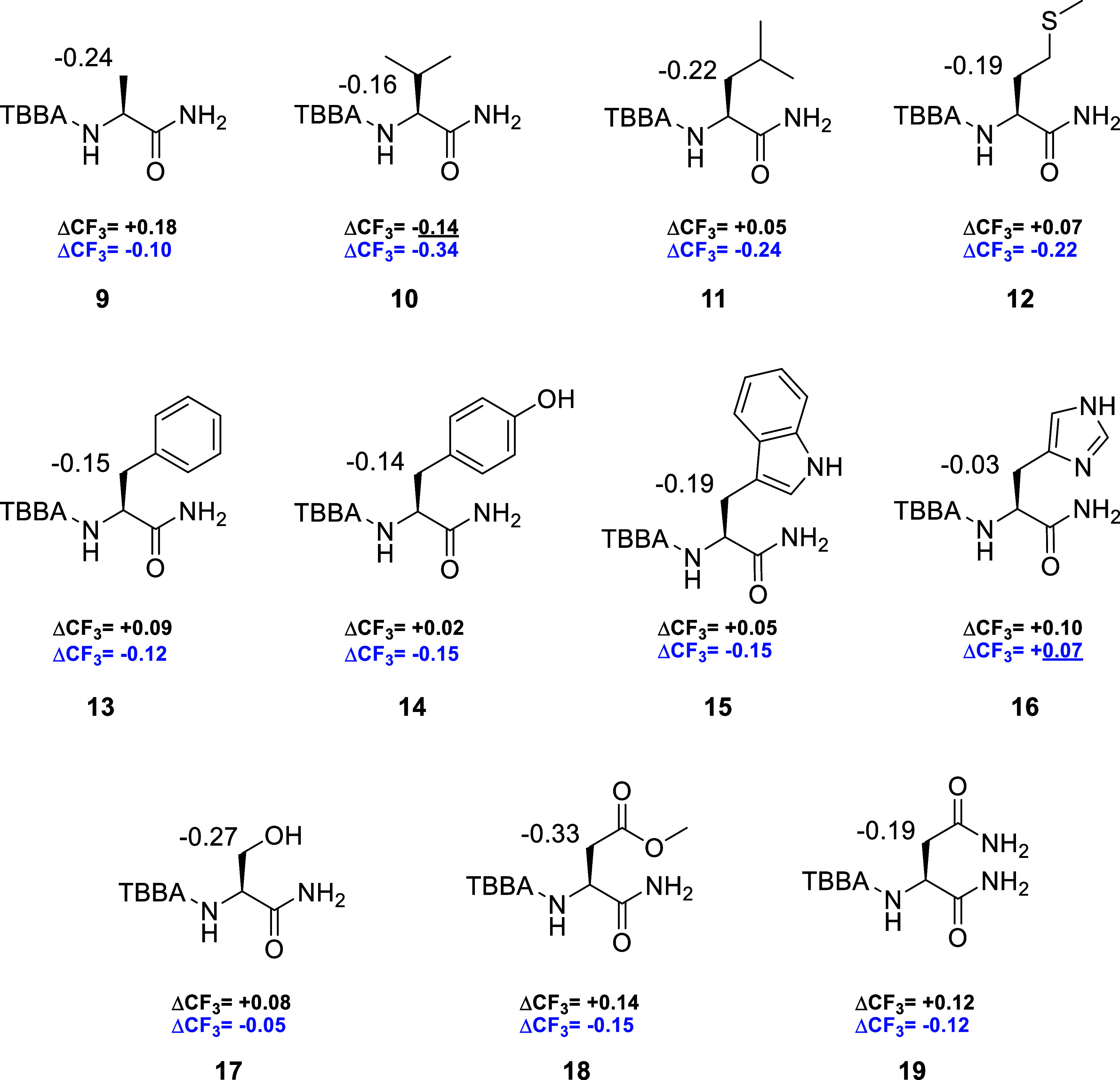
Differences in the chemical shifts (Δδ^PM^) of the β-protons in the ^1^H NMR spectra of TBBA amino acid amide diastereomers. The values were calculated according to the equation Δδ^PM^ = δL­(*P*) – δL­(*M*). Differences in the ^19^F NMR spectra are expressed as ΔCF_3_ (DMSO-*d*_6_ in black; THF in blue), with CFCl_3_ used as the internal standard. The chemical shift differences were calculated as ΔCF_3_ = δCF_3_^19^F­(*P*) – δCF_3_^19^F­(*M*). Anomalous values are underlined.

We further screened various nondeuterated solvents for benchtop NMR to identify one that would afford larger ΔCF_3_ values. Among these nondeuterated tetrahydrofuran (THF) provided the most pronounced ΔCF_3_ values. We hypothesized that the diastereoisomeric amino acid amide pairs would adopt conformations consistent with the previously described model in CDCl_3_ (Figure 1C). Alanine **9**, employed as a model compound, supported this assumption, displaying a ΔCF_3_ of −0.10 ppm. Valine **10** exhibited an even larger ΔCF_3_ of −0.34 ppm, substantially higher than in DMSO-*d*_6_. Leucine **11** and methionine **12** showed slightly smaller ΔCF_3_ values compared to valine **10** (−0.24 ppm for leucine **11** and −0.22 ppm for methionine **12**), yet these values still exceeded those measured in DMSO-*d*_6_.

Aromatic amino acids displayed lower ΔCF_3_ values in THF than aliphatic ones, although these values remained significantly higher than in DMSO-*d*_6_. Phenylalanine **13** exhibited a ΔCF_3_ of −0.12 ppm, while tyrosine **14** and tryptophan **15** both showed ΔCF_3_ −0.15 ppm. In contrast, histidine **16** was the only compound that yielded an anomalous ΔCF_3_ value of +0.07 ppm, which was likely caused by the formation of a competing intramolecular hydrogen bond that shifts the conformational equilibrium away from the general conformational model depicted in Figure 1C.

Serine **17** exhibited a lower ΔCF_3_ in THF compared to DMSO-*d*_6_, measuring −0.05 ppm. Interestingly, aspartic acid methyl ester **18** and asparagine **19** exhibited nearly identical ΔCF_3_ magnitudes in both THF and DMSO-*d*_6_.

The use of THF generally increases the ΔCF_3_ differences in ^19^F NMR spectra, which is particularly advantageous for low-field benchtop NMR measurements aimed at determining stereoisomeric ratios and assigning absolute configurations. Nevertheless, THF offers a narrower solubility range for various amino acid derivatives compared to DMSO.

After completing the study of individual amino acid amides (Figure 3), we extended our investigation to structurally related dipeptides and one tripeptide (Figure 4). This step aims to verify whether the application of TBBA remains suitable for assigning the absolute configuration of the *N*-terminal amino acid in the presence of an additional chiral center, even when the spectral interpretation may be complicated by overlapping signals.

**Figure 4:**
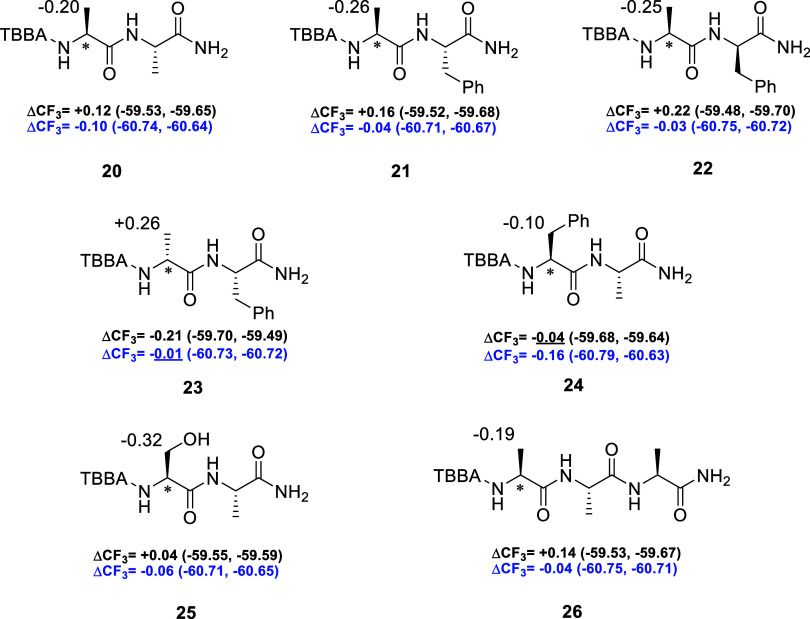
Differences in the chemical shifts (Δδ^PM^) of the β-protons in the ^1^H NMR spectra of TBBA peptide amide diastereomers. The values were calculated according to the equation Δδ^PM^ = δL­(*P*) – δL­(*M*). Differences in the ^19^F NMR spectra are expressed as ΔCF_3_ (DMSO-*d*_6_ in black; THF in blue), with CFCl_3_ used as the internal standard. The chemical shift differences were calculated as ΔCF_3_ = δCF_3_^19^F­(*P*) – δCF_3_^19^F­(*M*). Anomalous values are underlined. The analyzed α-chiral position is indicated with an asterisk.

Using the established models for analyzing individual amino acids by both ^1^H NMR and ^19^F NMR (Figure 1), we first examined dipeptide (l)-Ala-(l)-Ala **20** (Figure 4). In DMSO-*d*_6_, this dipeptide exhibited a Δδ^PM^ of −0.20 ppm in the ^1^H NMR spectrum, consistent with the behavior previously observed for alanine derivative **9**. In contrast, the ^19^F NMR spectrum revealed reduced ΔCF_3_ of +0.12 ppm, two-thirds the magnitude detected for the single amino acid derivative.

To evaluate the influence of absolute configurations on the chemical shift differences within Ala-Phe dipeptides, we analyzed (l)-Ala-(l)-Phe **21** and (l)-Ala-(d)-Phe **22** in DMSO-*d*_6_. The observed Δδ^PM^ were nearly identical (−0.26 and −0.25 ppm, respectively), suggesting that inversion of the phenylalanine stereocenter exerts only a minor effect on the proton environments near the chiral probe. In contrast, a more pronounced variation was detected in the ^19^F NMR spectra. (l)-Ala-(l)-Phe **21** exhibited a ΔCF_3_ of +0.16 ppm, whereas (l)-Ala-(d)-Phe **22** showed an increased value of +0.22 ppm. The corresponding (d)-Ala-(l)-Phe diastereomer **23** again displayed a similar Δδ^PM^ of +0.26 ppm, accompanied by a ΔCF_3_ of −0.21 ppm, consistent with the expected behavior for the opposite configuration. Importantly, a change in the configuration of the phenylalanine did not invert the sign of ΔCF_3_ which enabled a correct assignment of the absolute configuration of the N-terminal amino acid of dipeptides **21** – **23**.

Upon reversing the amino acid sequence, dipeptide (l)-Phe-(l)-Ala **24** exhibited a reduced Δδ^PM^ of −0.10 ppm, lower than those observed for the preceding Ala-Phe dipeptides. Notably, the ΔCF_3_ value of −0.04 ppm appeared anomalous and inconsistent with the predicted conformational model (Figure 1D). This anomalous value is likely caused by a conformation of the phenyl ring that produces a stronger shielding effect, which is enforced by the C-terminal alanine.

Dipeptide (l)-Ser-(l)-Phe **25** displayed a Δδ^PM^ of −0.32 ppm, exceeding that measured for serine derivative **17**. The corresponding ΔCF_3_ value decreased to +0.04 ppm, lower than that of serine **17**.

Tripeptide (l)-Ala-(l)-Ala-(l)-Ala **26** exhibited differences closely resembling those observed for monomeric alanine **9** and dipeptide (l)-Ala-(l)-Ala **20**. Specifically, the Δδ^PM^ value was measured at −0.19 ppm, while the ΔCF_3_ difference was +0.14 ppm, both in agreement with the proposed conformational model.

In THF, the absolute ΔCF_3_ values for the dipeptides and tripeptide were, in most cases, lower than those observed in DMSO-*d*_6_ and for the individual amino acids (Figure 3). (l)-Ala-(l)-Ala **20** exhibited an almost identical difference to that in DMSO-*d*_6_, with a value of −0.10 ppm. (l)-Ala-(l)-Phe **21** showed a ΔCF_3_ of −0.04 ppm, while (l)-Ala-(d)-Phe **22** exhibited a slightly lower value (−0.03 ppm). Both values were significantly lower compared to those obtained in DMSO-*d*_6_.

Additionally, (d)-Ala-(l)-Phe **23** displayed an even smaller ΔCF_3_ than its diastereoisomers, with a value of −0.01 ppm, which does not agree with the proposed conformational model for THF solution (Figure 1C). (l)-Phe-(l)-Ala **24** showed the highest ΔCF_3_ (−0.16 ppm) in THF among the oligopeptides synthesized. In the case of (l)-Ser-(l)-Phe **25**, the ΔCF_3_ in THF decreased to −0.06 ppm. Tripeptide **26** exhibited a ΔCF_3_ of −0.04 ppm, which was also lower than the corresponding values in DMSO-*d*_6_.

In general, the study of the oligopeptides presented in Figure 4 demonstrated that the assignment of the absolute configuration of the *N*-terminal amino acid is reliable by ^1^H NMR spectra (Δδ^PM^ differences). The use of ^19^F NMR spectroscopy is also feasible; however, one anomalous value was observed in DMSO-*d*_6_ (dipeptide **24**) and another in THF (dipeptide **23**). These deviations can nevertheless be resolved by complementary analysis of the ^1^H NMR spectra.

Moreover, it was demonstrated that the absolute configuration of the *N*-terminal amino acid can be determined even in the presence of a second chiral center located further from the TBBA moiety.

Furthermore, derivatization of oligopeptides with TBBA also enables the determination of optical purity by means of ^19^F NMR spectroscopy. It was demonstrated that the singlets corresponding to the trifluoromethyl group (see chemical shifts in parentheses in Figure 4) can be resolved in mixtures of structurally similar dipeptides **21**–**24** when measured using 400 MHz NMR instrumentation, and in some cases, even with a benchtop 80 MHz NMR spectrometer. The ^19^F NMR signal separations can be further modulated by the choice of (*P*)- or (*M*)-TBBA reagent and by the solvent employed.

## Conclusions

In summary, a second-generation synthesis of TBBA was developed, significantly streamlining the overall procedure and enabling the isolation of enantiomerically pure atropisomers through optical resolution via diastereomeric salt formation. The process was successfully scaled up to a 32 mmol batch, providing an operationally simple, cost-effective, and reproducible route. The first application of TBBA in SPPS demonstrated that this chiral derivatizing agent can be effectively employed for the determination of absolute configuration and stereochemical purity of amino acids and oligopeptides by means of both ^1^H and ^19^F NMR spectroscopy.

Due to the limited solubility of amino acid derivatives in CDCl_3_, a modified general conformational model was proposed to account for solvent-induced conformational changes in the amide group observed in polar DMSO-*d*_6_. The assignment of the absolute configuration of the *N*-terminal amino acid was found to be reliable by ^1^H NMR spectroscopy, while ^19^F NMR spectroscopy provided complementary information with only two exceptions among 18 derivatives studied in DMSO-*d*_6_ and THF. In these cases, combined analysis of ^1^H and ^19^F NMR spectra resolved the discrepancies.

The main advantage of ^19^F NMR analysis of TBBA derivatives lies in its ability to monitor stereochemical purity even for structurally similar stereoisomers, owing to the broad chemical shift range compared to ^1^H NMR, where signal overlap often limits accuracy. Overall, the use of TBBA as a chiral derivatizing agent for amino acid and peptide derivatives synthesized via solid-phase methodology represents a powerful NMR-based alternative to conventional chromatographic techniques. Moreover, the demonstrated applicability of benchtop ^19^F NMR instrumentation further extends the practicality and accessibility of this method.

## Supplementary Material



## Data Availability

The data underlying this study are available in the published article and its Supporting Information.

## Notes

Copies of spectra (^1^H NMR, ^13^C­{^1^H} NMR, and ^19^F NMR), HRMS, experimental procedures, conformational sampling and DFT calculations (PDF)
