# Supplementary material for: Second-Generation Synthesis and Analytical Application of TBBA for Chiral Analysis of Amino Acids and Oligopeptides by 1H and 19F NMR Spectroscopy
Source: J Org Chem. 2026 Jan 28;91(5):2065–73. doi: 10.1021/acs.joc.5c02693 (PMC12887996; doi:10.1021/acs.joc.5c02693)
Supplement: Supplementary file 1 [file jo5c02693_si_001.pdf]

Supporting Information

Second-Generation Synthesis and Analytical Application of TBBA for Chiral Analysis of Amino Acids and Oligopeptides by ^{1}H and ^{19}F NMR Spectroscopy

David Profous,[a] Naděžda Cankařová,[a] Jakob Enengl,[a] Sarin Soji,[a] Uwe Rinner,[b] Petr Jurečka,[c] and Petr Cankař[a,*]

[a]Department of Organic Chemistry, Faculty of Science, Palacký University, 17. listopadu 12, 771 46 Olomouc, Czech Republic

[b]Institute of Applied Chemistry, IMC Krems University of Applied Sciences, Piaristengasse 1, 3500 Krems, Austria

[c]Department of Physical Chemistry, Faculty of Science, Palacký University Olomouc, 17. listopadu 1192/12, 779 00 Olomouc, Czech Republic

Corresponding author: petr.cankar@upol.cz

Content

General Information S2

Second-Generation Racemic TBBA Synthesis S4

Optical Resolution of Racemic TBBA S5

Chiral SFC analysis of TBBA 4 ..... S6

General Procedure for the Synthesis of Amino Acid TBBA Amides[1] S7

Copies of NMR Spectra S27

Conformational Sampling and DFT Calculations S100

References S161

General Information

All reactions were performed under standard conditions without taking specific precautions to exclude moisture or air from the reaction, unless otherwise specified. All used chemical reagents were purchased from commercial sources. The solvents used for synthesis, reaction work-up and purification were reagent grade and were used without further purification, unless stated otherwise. Rink amide polystyrene resin (100–200 mesh, 1% DVB, 0.7 mmol/g) was used, and the solid-phase syntheses were performed in plastic reaction vessels (syringes, each equipped with a porous disc) using a manually operated synthesizer (www.torviq.com). The volume of wash solvent was 10 mL per 1 g of resin. For washing, resin slurry was shaken with the fresh solvent for at least 1 minute before changing the solvent. The yields of the products upon purification were calculated based on the initial loading of the first building block (amino acid).

For the LC/MS analysis, a sample of resin (~5 mg) was treated with 50% TFA in DCM, the cleavage cocktail was evaporated by a stream of nitrogen, and the cleaved compounds were extracted into 1 mL of MeOH. The LC/MS analyses were carried out using an Acquity UPLC H-class system (Waters, Milford, MA) (3 × 50 mm C18 reverse-phase column, 2.5 µm particles at 25 °C, and with a flow rate of 600 µL/min). Mobile phases: 10 mM ammonium acetate in HPLC grade water (A) and HPLC grade acetonitrile (B). The MS electrospray source was operated at a capillary voltage of 3 kV, probe temperature of 350 °C, and source temperature of 120 °C.

Purification was performed using a C18 reverse-phase column (19 × 100 mm, 5 µm particles). A 6-minute gradient was created using 10 mM aqueous ammonium acetate and acetonitrile in varying proportions, with a flow rate of 15 mL/min.

Analytical thin-layer chromatography (TLC) was performed using Kieselgel 60 F_{254} plates (Merck). Compounds were detected by UV light (255 nm) and then by basic $KMnO_4$ solution or ninhydrin solution. Flash chromatography was performed using silica gel (35−70 µm particle size).

^{1}H NMR, ^{13}C NMR, and ^{19}F NMR spectra were measured on Jeol ECA400II (400 MHz) or Jeol ECX-500SS (500 MHz) instrument in DMSO-d_6, as a solvent. ^{19}F NMR spectra were also measured on Spinsolve 80 Ultra (80 MHz) instrument in DMSO-d_6 or THF (non-deuterated), as solvents. ^{1}H and ^{13}C spectra were calibrated using residual non-deuterated solvent as an internal reference (2.50 and 39.52 ppm for DMSO-d_6,). ^{19}F spectra were calibrated by the addition of $CFCl_3$ as an internal reference (δ = 0.0 ppm). All ^{13}C NMR spectra were measured with broadband ^{1}H decoupling. ^{1}H NMR data are reported as follows: δ, chemical shift; coupling constants (*J* are given in Hertz, Hz) and integration. Abbreviations to denote the multiplicity of a particular signal were s (singlet), d (doublet), t (triplet), q (quartet), m (multiplet), p (pentet), app (appears as) and br (broad).

HRMS analyses were conducted using an Exactive Plus Orbitrap high-resolution mass spectrometer with electrospray ionization (Thermo Fisher Scientific, MA, USA). Chromatographic pre-separation was performed using an HPLC system Dionex Ultimate 3000 (Thermo Fisher Scientific, MA, USA) equipped with a Phenomenex Gemini column (C18, 50 × 2 mm, 3.0 µm). The samples were dissolved in MeOH or acetonitrile and injected using an autosampler. Mobile phase composition was isocratic elution with MeOH/water (95:5) containing 0.1% (v/v) HCOOH at a flow rate of 0.3 mL/min.

SFC chiral analyses were conducted using an Acquity UPC2 system (Waters), which included a binary solvent manager, sample manager, column manager, column heater, convergence manager, PDA detector 2998, QDa mass detector, and chiral analytical columns Chiralpak IA3 (4.6 mm × 100 mm, 3 µm particle size). The chromatographic runs were carried out at a flow rate of 2.2 mL/min, column temperature of 38 °C, and ABPR of 2000 psi.

Second-Generation Racemic TBBA Synthesis

2-((2-Nitrophenyl)amino)benzoic Acid 2

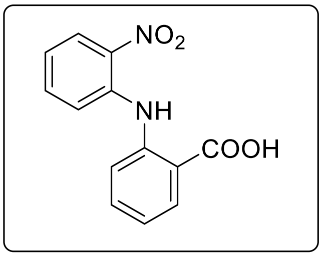

Anthranilic acid (244 mmol, 1 equiv, 33.5 g) was dissolved in DMF (60 mL), followed by the addition of 2-fluoronitrobenzene (244 mmol, 1 equiv, 26 mL), K_2CO_3 (244 mmol, 1 equiv, 33.7 g), and copper powder (4.9 mmol, 0.02 equiv, 0.32 g). The reaction mixture was refluxed for 16 hours. After completion, the resulting muddy suspension was cooled to room temperature and poured into a mixture of ice-cold distilled water (400 mL), which was acidified by the addition of glacial acetic acid (300 mL). The suspension was stirred for 4 hours, affording a fine brownish-yellow precipitate, which was filtered, washed with distilled water (300 mL), and dried in an oven at 90 °C to yield 53.9 g (87 %) of a yellow-brown solid. The compound can be recrystallized from glacial acetic acid.

2-((2-Aminophenyl)amino)benzoic Acid 3

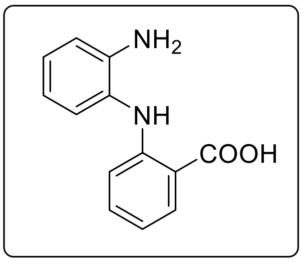

The 10 wt.% Pd on carbon (0.02 equiv; 3.9 g) was carefully suspended under a stream of argon in ethyl acetate (400 mL) in a 4 L three-necked flask. 2-((2-Nitrophenyl)amino)benzoic acid (**2**, 201 mmol, 1 equiv, 52 g) was added to the suspension, followed by ethyl acetate (2.6 L). The air was removed from the apparatus, and two hydrogen balloons were added. The reaction mixture was stirred vigorously at room temperature for 15 hours, while the hydrogen balloons were replaced every three hours. When the reaction reached complete conversion to product (monitored by NMR), it was filtered through Celite, washed three times with ethyl acetate (150 mL), and the filtrate was evaporated under reduced pressure to obtain 43.7 g (95 %) of a yellow-brown solid.

2-(2-(Trifluoromethyl)-1*H*-benzo[*d*]imidazol-1-yl)benzoic acid 4

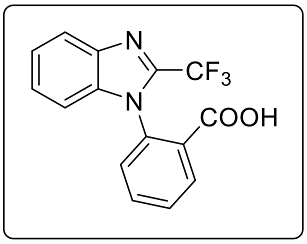

To the 2-((2-aminophenyl)amino)benzoic acid (**3**, 184 mmol, 1 equiv, 42 g) was slowly added trifluoroacetic anhydride (284 mL, c = 0.65 mmol/mL), and the reaction mixture was heated to reflux for 3 hours. The solution was then cooled to room temperature and poured dropwise, with vigorous stirring, into distilled water (3 L) containing a large amount of ice. The mixture was stirred until a fine white precipitate was formed, which was filtered and washed with ice-cold water (300 mL). The product was dried in an oven at 90 °C and recrystallized from a mixture of EtOAc/cyclohexane (1:1, 1 g TBBA per 9 mL of mixture). After dissolution of the product, it was

necessary to stir the mixture for 16 hours at room temperature. The precipitated solid was filtered, the filtrate was evaporated under reduced pressure, and dried in an oven at 90 °C. This recrystallization procedure was repeated three times with the filtrate. The precipitates were combined to obtain 45.6 g (81 %) of pinkish crystals, mp: 214 – 215 °C.

Optical Resolution of Racemic TBBA

Racemic 2-(2-(trifluoromethyl)-1*H*-benzo[*d*]imidazol-1-yl)benzoic acid (***rac*-4**, 32.6 mmol, 1 equiv, 10 g) was suspended in EtOAc (225 mL), and (1*S*,2*S*)-(−)-1,2-diphenylethylenediamine (16.3 mmol, 0.5 equiv, 3.46 g) was added. The reaction mixture was refluxed until the diastereomeric salt fully dissolved. If complete dissolution was not achieved, a small amount of additional EtOAc was added. After dissolution, heating was stopped, and the mixture was stirred at room temperature for 16 hours at a stirring rate above 1000. Gradually, crystals formed, and the solution developed a gel-like consistency.

The precipitate was filtered off and washed with EtOAc (15 mL) to remove residual amounts of the second diastereoisomeric salt, increasing the ee to >99%. The crystals were dried, followed by the addition of 10 % (v/v) HCl (30 mL), and the suspension was stirred at room temperature for 90 minutes. The mixture was filtered again, and the crystals were dried to give 1.2 g (12 % from racemic acid) of (*M)*-TBBA with ee >99%, mp: 217 – 218 °C.

The filtrate from the first filtration was evaporated under reduced pressure, dried using a freeze dryer, treated with 10 % (v/v) HCl (150 mL), and stirred at room temperature for 5 hours. The resulting precipitate was filtered and dried to afford 7.95 g of (*P)*-TBBA with 30% ee.

The mixture of TBBA enantiomers isolated from the filtrate (25.6 mmol, 1 equiv, 7.85 g) was dissolved in EtOAc (195 mL), and (1*R*,2*R*)-(+)-1,2-diphenylethylenediamine (12.8 mmol, 0.5 equiv, 2.72 g) was added. The same procedure as described above was followed. The yield of (*P)*-TBBA was 1.35 g (17 %) with ee >99 %, mp: 217 – 218 °C. The EtOAc filtrate contained (*P)*-TBBA and (*M)*-TBBA in approximately a 1:1 ratio, which can be reused.

Amine Recovery

The aqueous filtrates containing the same amine enantiomer were combined, and the pH was adjusted to 12 using a 10 % NaOH solution. The resulting solution was extracted with CH_2Cl_2, and the combined organic layers were dried over $MgSO_4$ and concentrated under reduced pressure. The amine was recovered in 2.77 g yield (80 %).

Chiral SFC analysis of TBBA 4

Racemic mixture

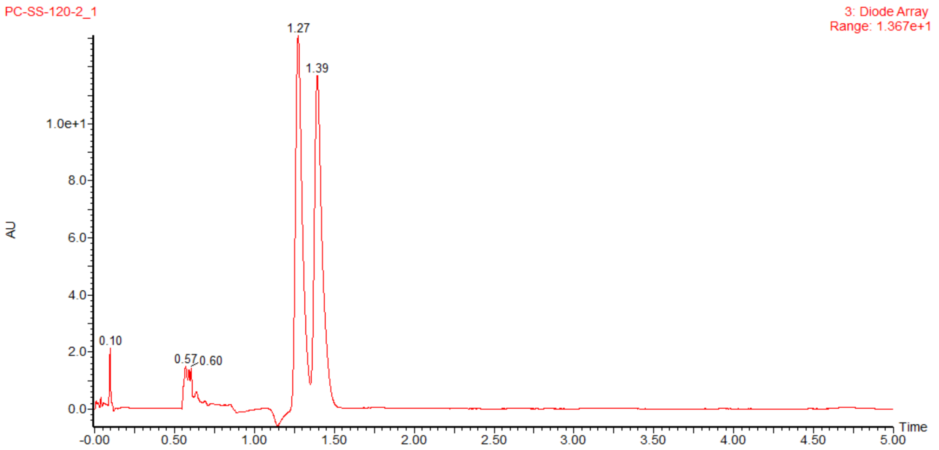

Enantiopure samples: (*M*)-TBBA (top), (*P*)-TBBA (bottom)

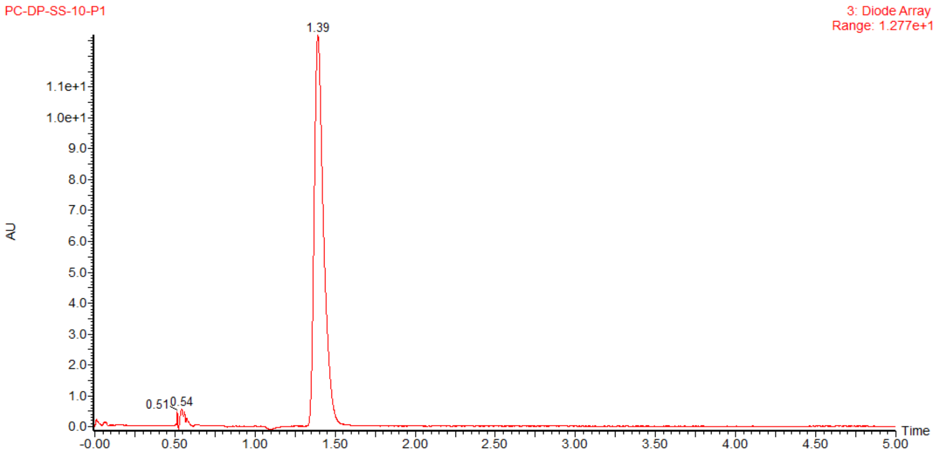

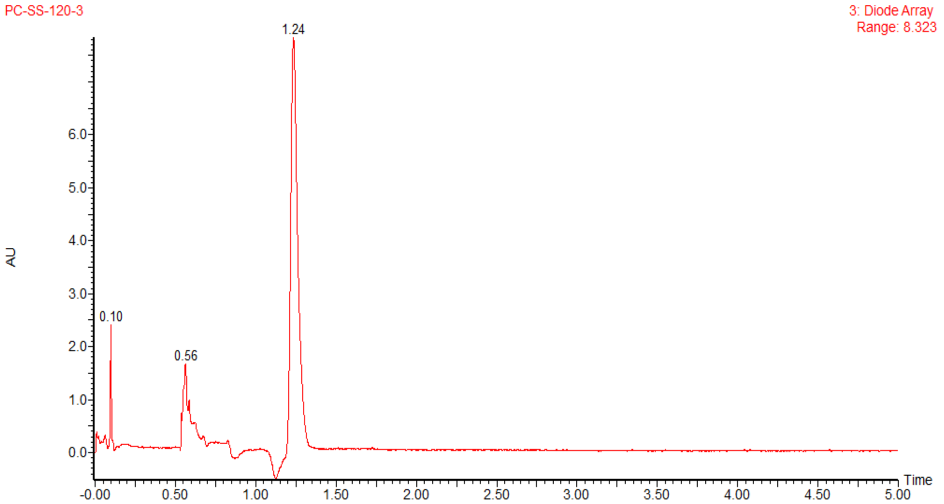

Conditions:

90% CO_2, 10% MeOH + 0,1% TFA + 1% H_2O, column CHIRALPAK IA3,
flow: 2,2 ml/min, column temperature: 38˚C, abpr: 2000, make-up pump flow: 0,45 ml/min (MeOH)

General Procedure for the Synthesis of Amino Acid TBBA Amides[1]

Cleavage of Fmoc-Protecting Group of Rink Amide Resin and Acylation with Fmoc-Protected Amino Acid (Resin 6)

Rink amide resin (**5**, 0.5 g) was washed three times with CH_2Cl_2 and three times with DMF and then treated with 50% piperidine in DMF (5 mL) at room temperature for 15 minutes. Subsequently, the resin was washed three times with DMF and five times with CH_2Cl_2. A solution of Fmoc-protected amino acid (0.38 mmol; 1 equiv), HOBt (0.38 mmol; 1 equiv; 57 mg), and DIC (0.38 mmol; 1 equiv; 60 µL) in 5 mL of CH_2Cl_2/DMF (1:1) was added to the resin. The mixture was shaken at room temperature for 16 hours and afterward washed three times with DMF and three times with CH_2Cl_2. **Synthesis of Peptides.** The previous part of the procedure was repeated multiple times to prepare target peptides.

Reaction with Fmoc-OSu and Determination of Resin Substitution (Loading).

Samples of dry resin **6** (n = 1; 10 mg) were treated with solution of 0.5 M Fmoc-Osu (0.5 mmol, 169 mg) in 1 mL CH_2Cl_2 for 30 minutes. The resin was washed five times with CH_2Cl_2 and treated with 50% TFA in CH_2Cl_2 for 30 minutes. The solutions were subsequently evaporated by a stream of nitrogen, and the cleaved compounds were extracted with 1 mL MeOH and analyzed by LC/MS. The area of peak of Fmoc-modified product was compared with the area of a standard solution (Fmoc-α-Ala-OH).

Cleavage of Fmoc-Protecting Group and Acylation with (P)- or (M)-TBBA (Resin 7)

The amino acids-substituted resin **6** was washed three times with DMF. It was then treated with 50% piperidine in DMF (5 mL) for 15 minutes. Afterwards, the resin was washed three times with DMF and three times with CH_2Cl_2. A solution of (*P*)- or (*M*)-TBBA (0.38 mmol; 1 equiv; 115 mg), HOBt (0.38 mmol; 1 equiv; 57 mg), DIC (0.38 mmol; 1 equiv; 60 µL), and DMAP (0.38 mmol; 1 equiv; 46 mg) in 5 mL of CH_2Cl_2/DMF (1:1) was added to the resin **7**. The reaction time varied from 16 hours to 64 hours. Subsequently, the resin was washed three times with DMF and five times with CH_2Cl_2. Acylation of peptides followed the same procedure.

Cleavage of Protecting Groups, Cleavage from the Resin (8)

Resin **(*P*)-7** or **(*M*)-7** (0.5 g) was treated with 5 mL of 50% TFA in CH_2Cl_2 at room temperature for 1 hour. The TFA solution was collected, the resin was washed three times with 4 mL 50% TFA in CH_2Cl_2, and the combined extracts were evaporated by a stream of nitrogen.

Purification of the Product 8

Products were purified via column chromatography (CC) or semipreparative reverse-phase HPLC by using mobile-phase MeCN and 10 mM aqueous ammonium acetate.

The derivatives **(*P*)-16**, **(*M*)-16**, **(*P*)-19**, and **(*M*)-19** were dissolved in 2 mL of CH_2Cl_2/MeOH (1:1) and 2 mL of triethylamine was added. This mixture was stirred for 6 hours and subsequently the volatile species were evaporated under a stream of nitrogen. The residue was dissolved in EtOAc (8 mL) and washed 3 times with distilled water (8 mL). The aqueous phases were combined and extracted 3 times with EtOAc (8 mL). The organic phases were combined, dried over $MgSO_4$ and evaporated under reduced pressure.

(*P*)-TBBA-(*L*)-Ala-NH_2 **(*P*)-9**

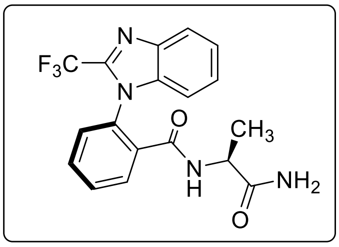

Following the general procedure, from Fmoc-Ala-OH (0.38 mmol; 1 equiv; 118 mg) and (*P*)-TBBA, reaction time 16 hours, with purification by CC (CH_2Cl_2/MeOH 20:1; column dimensions 4 × 10 cm), 80 mg (87% yield) of white solid was obtained. **^{1}H NMR** (400 MHz, DMSO-d_6) δ 8.45 (d, *J* = 7.7 Hz, 1H), 7.90 – 7.82 (m, 2H), 7.79 – 7.71 (m, 2H), 7.68 – 7.62 (m, 1H), 7.42 – 7.34 (m, 2H), 7.23 (s, 1H), 7.17 – 7.11 (m, 1H), 6.94 (s, 1H), 3.89 (p, *J* = 7.1 Hz, 1H), 0.85 (d, *J* = 7.1 Hz, 3H). **^{13}C{^{1}H} NMR** (101 MHz, DMSO-d_6) δ 173.6, 164.7, 140.4 (q, *J* = 38.3 Hz), 140.2, 136.8, 134.7, 131.7, 131.3, 130.2, 129.1, 129.1, 125.4, 123.6, 120.6, 118.7 (q, *J* = 271.9 Hz), 111.5, 48.0, 17.4. **^{19}F NMR** (376 MHz, DMSO-d_6) δ -59.52 (s). **^{19}F NMR** (76 MHz, THF) δ -60.80 (s). **HRMS** (ESI): *m/z* [M + H]$^+$ Calcd for $C_{18}H_{16}F_3N_4O_2$ 377.1220; Found 377.1216.

(*M*)-TBBA-(*L*)-Ala-NH_2 **(*M*)-9**

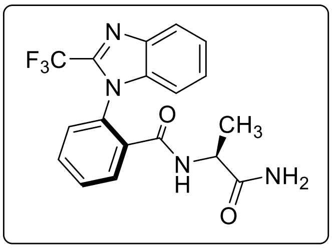

Following the general procedure, from Fmoc-Ala-OH (0.38 mmol; 1 equiv; 118 mg) and (*M*)-TBBA, reaction time 40 hours, with purification by CC (CH_2Cl_2/MeOH 20:1; column dimensions 4 × 10 cm), 54 mg (59% yield) of white solid was obtained. **^{1}H NMR** (400 MHz, DMSO-d_6) δ 8.40 (d, *J* = 7.4 Hz, 1H), 7.97 – 7.91 (m, 1H), 7.88 – 7.83 (m, 1H), 7.80 – 7.72 (m, 2H), 7.67 – 7.61 (m, 1H), 7.42 – 7.34 (m, 2H), 7.08 – 7.03 (m, 1H), 7.01 (br. s, 1H), 6.85 (br. s, 1H), 4.01 (p, *J* = 7.1 Hz, 1H), 1.09 (d, *J* = 7.1 Hz, 3H). **^{13}C{^{1}H} NMR** (101 MHz, DMSO-d_6) δ 173.5, 164.4, 140.0, 139.8 (q, *J* = 37.8 Hz), 137.5, 134.0, 131.9, 131.6, 130.3, 129.5, 125.7, 123.6, 120.6, 118.8 (q, *J* = 271.7 Hz), 111.6, 48.4, 17.7. **^{19}F NMR** (376 MHz, DMSO-d_6) δ -59.70 (s). **^{19}F NMR** (76 MHz, THF) δ -60.70 (s). **HRMS** (ESI): *m/z* [M + H]$^+$ Calcd for $C_{18}H_{16}F_3N_4O_2$ 377.1220; Found 377.1219.

(*P*)-TBBA-(*L*)-Val-NH$_2$ **(*P*)-10**

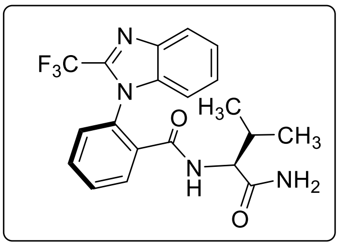

Following the general procedure, from Fmoc-Val-OH (0.38 mmol; 1 equiv; 129 mg) and (*P*)-TBBA, reaction time 32 hours, with purification by CC (CH_2Cl_2/MeOH 18:1; column dimensions 4 × 10 cm), 50 mg (59% yield) of white solid was obtained. **^{1}H NMR** (400 MHz, DMSO-d_6) δ 8.20 (d, *J* = 8.7 Hz, 1H), 7.87 – 7.78 (m, 2H), 7.78 – 7.70 (m, 2H), 7.67 – 7.61 (m, 1H), 7.40 – 7.32 (m, 2H), 7.29 (br. s, 1H), 7.16 (dd, *J* = 6.7, 2.6 Hz, 1H), 6.98 (br. s, 1H), 3.83 (dd, *J* = 8.6, 6.7 Hz, 1H), 1.77 (dq, *J* = 13.5, 6.8 Hz, 1H), 0.53 (d, *J* = 6.8 Hz, 3H), 0.50 (d, *J* = 6.8 Hz, 3H). **^{13}C{^{1}H} NMR** (101 MHz, DMSO-d_6) δ 172.4, 165.1, 140.4 (q, *J* = 38.0 Hz), 140.1, 137.1, 134.9, 131.7, 131.2, 130.2, 129.3, 129.2, 125.6, 123.6, 120.5, 118.7 (q, *J* = 271.8 Hz), 111.6, 58.0, 30.0, 18.9, 17.7. **^{19}F NMR** (376 MHz, DMSO-d_6) δ -59.60 (s). **^{19}F NMR** (76 MHz, THF) δ -60.86 (s). **HRMS** (ESI): *m/z* [M + H]$^+$ Calcd for $C_{20}H_{20}F_3N_4O_2$ 405.1533; Found 405.1534.

(*M*)-TBBA-(*L*)-Val-NH$_2$ **(*M*)-10**

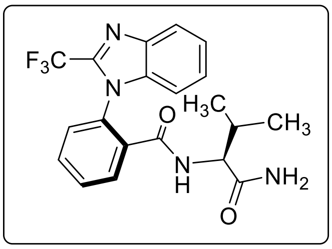

Following the general procedure, from Fmoc-Val-OH (0.38 mmol; 1 equiv; 129 mg) and (*M*)-TBBA, reaction time 32 hours, with purification by CC (CH_2Cl_2/MeOH 18:1; column dimensions 4 × 10 cm), 21 mg (25% yield) of white solid was obtained. **^{1}H NMR** (400 MHz, DMSO-d_6) δ 8.15 (d, *J* = 8.6 Hz, 1H), 7.91 – 7.81 (m, 2H), 7.78 – 7.73 (m, 2H), 7.66 – 7.61 (m, 1H), 7.40 – 7.33 (m, 2H), 7.11 – 7.03 (m, 2H), 6.89 (br. s, 1H), 3.89 (dd, *J* = 8.6, 6.7 Hz, 1H), 1.93 (hept, *J* = 6.9 Hz, 1H), 0.76 (d, *J* = 6.8 Hz, 3H), 0.74 (d, *J* = 6.8 Hz, 3H). **^{13}C{^{1}H} NMR** (101 MHz, DMSO-d_6) δ 172.2, 164.9, 140.0, 139.9 (q, *J* = 37.7 Hz), 137.3, 134.3, 131.7, 131.5, 130.3, 129.5, 129.4, 125.7, 123.6, 120.6, 118.8 (q, *J* = 271.9 Hz), 111.6, 58.1, 30.0, 19.1, 17.8. **^{19}F NMR** (376 MHz, DMSO-d_6) δ -59.46 (s). **^{19}F NMR** (76 MHz, THF) δ -60.52 (s). **HRMS** (ESI): *m/z* [M + H]$^+$ Calcd for $C_{20}H_{20}F_3N_4O_2$ 405.1533; Found 405.1535.

(*P*)-TBBA-(*L*)-Leu-NH$_2$ **(*P*)-11**

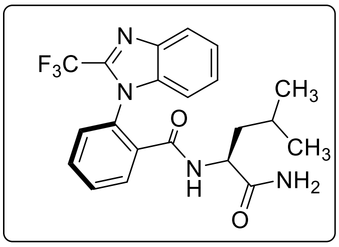

Following the general procedure, from Fmoc-Leu-OH (0.38 mmol; 1 equiv; 134 mg) and (*P*)-TBBA, reaction time 16 hours, with purification by CC (CH_2Cl_2/MeOH 18:1; column dimensions 4 × 10 cm), 50 mg (61% yield) of white solid was obtained. **^{1}H NMR** (400 MHz, DMSO-*d*$_6$) δ 8.43 (d, *J* = 8.3 Hz, 1H), 7.88 – 7.82 (m, 2H), 7.77 – 7.72 (m, 2H), 7.67 – 7.63 (m, 1H), 7.40 – 7.32 (m, 2H), 7.23 (br. s, 1H), 7.07 – 7.03 (m, 1H), 6.90 (br. s, 1H), 3.94 (ddd, *J* = 10.7, 8.4, 4.5 Hz, 1H), 1.35 – 1.28 (m, 1H), 1.22 – 1.17 (m, 1H), 1.12 – 1.03 (m, 1H), 0.65 (d, *J* = 6.6 Hz, 3H), 0.30 (d, *J* = 6.4 Hz, 3H). **^{13}C{^{1}H} NMR** (101 MHz, DMSO-*d*$_6$) δ 173.8, 164.9, 140.6 (q, *J* = 37.3 Hz), 140.2, 136.9, 134.8, 131.8, 131.3, 130.2, 129.2, 129.2, 125.5, 123.5, 120.5, 118.7 (q, *J* = 272.2 Hz), 111.2, 50.9, 23.8, 23.0, 20.6. **^{19}F NMR** (376 MHz, DMSO-*d*$_6$) δ -59.59 (s). **^{19}F NMR** (76 MHz, THF) δ -60.87 (s). **HRMS** (ESI): *m/z* [M + H]$^+$ Calcd for $C_{21}H_{22}F_3N_4O_2$ 419.1689; Found 419.1687.

(*M*)-TBBA-(*L*)-Leu-NH$_2$ **(*M*)-11**

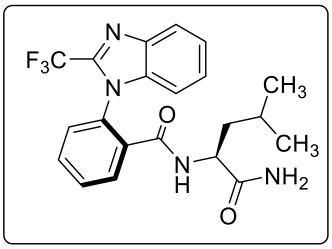

Following the general procedure, from Fmoc-Leu-OH (0.38 mmol; 1 equiv; 134 mg) and (*M*)-TBBA, reaction time 40 hours, with purification by CC (CH_2Cl_2/MeOH 18:1; column dimensions 4 × 10 cm), 38 mg (47% yield) of white solid was obtained. **^{1}H NMR** (400 MHz, DMSO-*d*$_6$) δ 8.42 (d, *J* = 8.1 Hz, 1H), 7.95 – 7.89 (m, 1H), 7.87 – 7.82 (m, 1H), 7.79 – 7.73 (m, 2H), 7.66 – 7.60 (m, 1H), 7.40 – 7.33 (m, 2H), 7.07 – 7.02 (m, 1H), 6.99 (br. s, 1H), 6.79 (br. s, 1H), 4.03 (ddd, *J* = 10.3, 8.1, 4.5 Hz, 1H), 1.54 – 1.42 (m, 2H), 1.40 – 1.30 (m, 1H), 0.82 (d, *J* = 6.3 Hz, 3H), 0.71 (d, *J* = 6.3 Hz, 3H). **^{13}C{^{1}H} NMR** (101 MHz, DMSO-*d*$_6$) δ 173.5, 164.7, 140.0, 139.8 (q, *J* = 37.1 Hz), 137.5, 134.1, 131.9, 131.5, 130.3, 129.6, 129.4, 125.6, 123.5, 120.5, 118.8 (q, *J* = 271.8 Hz), 111.7, 51.3, 24.1, 23.0, 21.0. **^{19}F NMR** (376 MHz, DMSO-*d*$_6$) δ -59.64 (s). **^{19}F NMR** (76 MHz, THF) δ -60.63 (s). **HRMS** (ESI): *m/z* [M + H]$^+$ Calcd for $C_{21}H_{22}F_3N_4O_2$ 419.1689; Found 419.1689.

(*P*)-TBBA-(L)-Met-NH_2 **(*P*)-12**

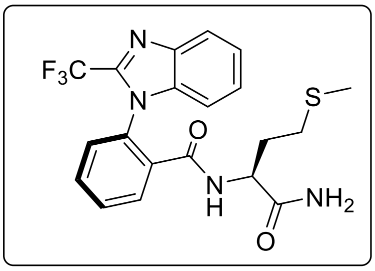

Following the general procedure, from Fmoc-Met-OH (0.38 mmol; 1 equiv; 141 mg) and (*P*)-TBBA, reaction time 32 hours, with purification by CC (CH_2Cl_2/MeOH 18:1; column dimensions 4 × 10 cm), 52 mg (72% yield) of white solid was obtained. **^{1}H NMR** (400 MHz, DMSO-d_6) δ 8.52 (d, J = 8.2 Hz, 1H), 7.91 – 7.82 (m, 2H), 7.81 – 7.71 (m, 2H), 7.70 – 7.60 (m, 1H), 7.43 – 7.32 (m, 2H), 7.26 (br. s, 1H), 7.16 – 7.07 (m, 1H), 7.01 (br. s, 1H), 3.99 (td, J = 8.7, 4.6 Hz, 1H), 2.11 – 2.01 (m, 1H), 1.98 – 1.90 (m, 1H), 1.86 (s, 3H), 1.73 – 1.63 (m, 1H), 1.60 – 1.49 (m, 1H). **^{13}C{^{1}H} NMR** (101 MHz, DMSO-d_6) δ 172.6, 165.1, 140.4 (q, J = 38.0 Hz), 140.1, 136.9, 134.6, 131.8, 131.4, 130.2, 129.3, 129.2, 125.5, 123.7, 120.6, 118.7 (q, J = 271.9 Hz), 111.3, 52.0, 31.4, 29.5, 14.4. **^{19}F NMR** (376 MHz, DMSO-d_6) δ -59.54 (s). **^{19}F NMR** (76 MHz, THF) δ -60.83 (s). **HRMS** (ESI): *m/z* $[M + H]^+$ Calcd for $C_{20}H_{20}F_3N_4O_2S$ 437.1254; Found 437.1252.

(*M*)-TBBA-(*L*)-Met-NH_2 **(*M*)-12**

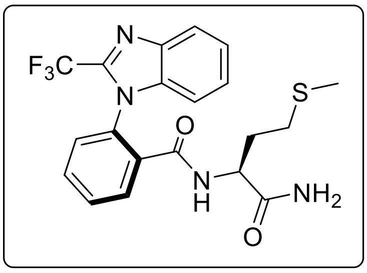

Following the general procedure, from Fmoc-Met-OH (0.38 mmol; 1 equiv; 141 mg) and (*M*)-TBBA, reaction time 64 hours, with purification by CC (CH_2Cl_2/MeOH 18:1; column dimensions 4 × 10 cm), 49 mg (68% yield) of white solid was obtained. **^{1}H NMR** (400 MHz, DMSO-d_6) δ 8.51 (d, J = 7.9 Hz, 1H), 7.99 – 7.92 (m, 1H), 7.88 – 7.83 (m, 1H), 7.80 – 7.73 (m, 2H), 7.67 – 7.60 (m, 1H), 7.42 – 7.34 (m, 2H), 7.07 – 7.02 (m, 1H), 7.00 (br. s, 1H), 6.90 (br. s, 1H), 4.07 (td, J = 8.9, 4.7 Hz, 1H), 2.40 – 2.25 (m, 2H), 1.99 (s, 3H), 1.88 – 1.72 (m, 2H). **^{13}C{^{1}H} NMR** (101 MHz, DMSO-d_6) δ 172.5, 164.9, 140.0, 139.8 (q, J = 37.4 Hz), 137.5, 134.0, 131.9, 131.6, 130.3, 129.6, 125.6, 123.6, 120.6, 118.8 (q, J = 271.9 Hz), 111.6, 52.2, 31.2, 29.7, 14.5. **^{19}F NMR** (376 MHz, DMSO-d_6) δ -59.61 (s). **^{19}F NMR** (76 MHz, THF) δ -60.61 (s). **HRMS** (ESI): *m/z* $[M + H]^+$ Calcd for $C_{20}H_{20}F_3N_4O_2S$ 437.1254; Found 437.1253.

(P)-TBBA-(L)-Phe-NH_2 **(P)-13**

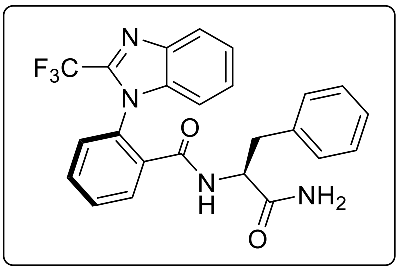

Following the general procedure, from Fmoc-Phe-OH (0.38 mmol; 1 equiv; 147 mg) and (P)-TBBA, reaction time 16 hours, with purification by CC (CH_2Cl_2/MeOH 30:1; column dimensions 4 × 10 cm), 70 mg (56% yield) of white solid was obtained. **^{1}H NMR** (400 MHz, DMSO-d_6) δ 8.59 (d, J = 8.2 Hz, 1H), 7.83 (dt, J = 8.2, 0.9 Hz, 1H), 7.81 – 7.77 (m, 1H), 7.77 – 7.70 (m, 2H), 7.63 – 7.58 (m, 1H), 7.35 (ddd, J = 8.2, 7.2, 1.1 Hz, 1H), 7.30 – 7.23 (m, 2H), 7.22 – 7.13 (m, 3H), 7.12 – 7.03 (m, 2H), 6.98 (br. s, 1H), 6.92 (dt, J = 8.2, 0.9 Hz, 1H), 4.21 (td, J = 9.0, 5.3 Hz, 1H), 2.84 (dd, J = 13.9, 5.3 Hz, 1H), 2.65 (dd, J = 13.9, 9.2 Hz, 1H). **^{13}C{^{1}H} NMR** (101 MHz, DMSO-d_6) δ 172.5, 164.6, 140.1 (q, J = 37.7 Hz), 140.0, 137.9, 137.2, 133.9, 132.0, 131.6, 130.3, 129.5, 129.3, 128.9, 128.0, 126.1, 125.6, 123.4, 120.5, 118.7 (q, J = 271.9 Hz), 111.4, 54.1, 37.0. **^{19}F NMR** (376 MHz, DMSO-d_6) δ -59.61 (s). **^{19}F NMR** (76 MHz, THF) δ -60.79 (s). **HRMS** (ESI): m/z [M + H]$^+$ Calcd for $C_{24}H_{20}F_3N_4O_2$ 453.1533; Found 453.1530.

(M)-TBBA-(L)-Phe-NH_2 **(M)-13**

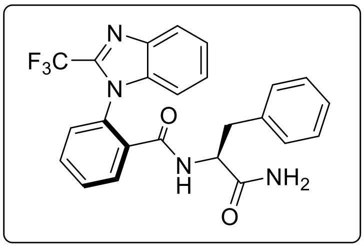

Following the general procedure, from Fmoc-Phe-OH (0.38 mmol; 1 equiv; 147 mg) and (M)-TBBA, reaction time 40 hours, with purification by CC (CH_2Cl_2/MeOH 30:1; column dimensions 4 × 10 cm), 58 mg (43% yield) of white solid was obtained. **^{1}H NMR** (400 MHz, DMSO-d_6) δ 8.56 (d, J = 8.4 Hz, 1H), 7.85 – 7.80 (m, 1H), 7.77 – 7.67 (m, 3H), 7.63 – 7.56 (m, 1H), 7.39 – 7.31 (m, 2H), 7.29 – 7.16 (m, 5H), 7.06 – 7.00 (m, 1H), 6.96 (br. s, 1H), 6.87 (br. s, 1H), 4.27 (ddd, J = 10.0, 8.5, 4.6 Hz, 1H), 2.98 (dd, J = 13.8, 4.6 Hz, 1H), 2.81 (dd, J = 13.8, 10.1 Hz, 1H). **^{13}C{^{1}H} NMR** (101 MHz, DMSO-d_6) δ 172.4, 164.6, 140.1 (q, J = 37.9 Hz), 140.0, 138.0, 137.1, 133.8, 132.0, 131.6, 130.2, 129.5, 129.2, 129.0, 128.0, 126.2, 125.6, 123.6, 120.5, 118.6 (q, J = 271.8 Hz), 111.4, 54.1, 37.2. **^{19}F NMR** (376 MHz, DMSO-d_6) δ -59.70 (s). **^{19}F NMR** (76 MHz, THF) δ -60.67 (s). **HRMS** (ESI): m/z [M + H]$^+$ Calcd for $C_{24}H_{20}F_3N_4O_2$ 453.1533; Found 453.1530.

(*P*)-TBBA-(*L*)-Tyr-NH_2 **(*P*)-14**

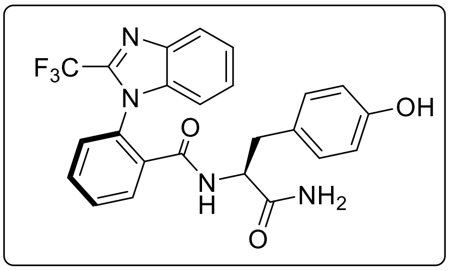

Following the general procedure, from Fmoc-Tyr(*t*Bu)-OH (0.38 mmol; 1 equiv; 175 mg) and (*P*)-TBBA, reaction time 16 hours, with purification by CC (CH_2Cl_2/MeOH 15:1; column dimensions 4 × 10 cm), 29 mg (21% yield) of white solid was obtained. **^{1}H NMR** (400 MHz, DMSO-d_6) δ 9.13 (br. s, 1H), 8.49 (d, *J* = 8.2 Hz, 1H), 7.85 – 7.77 (m, 2H), 7.77 – 7.70 (m, 2H), 7.61 (dt, *J* = 4.3, 3.0 Hz, 1H), 7.35 (ddd, *J* = 8.2, 7.2, 1.2 Hz, 1H), 7.27 (ddd, *J* = 8.2, 7.2, 1.1 Hz, 1H), 7.21 (br. s, 1H), 6.93 (dt, *J* = 8.1, 1.0 Hz, 2H), 6.89 – 6.84 (m, 2H), 6.61 – 6.55 (m, 2H), 4.11 (td, *J* = 8.9, 5.4 Hz, 1H), 2.72 (dd, *J* = 13.9, 5.3 Hz, 1H), 2.54 (dd, *J* = 14.5, 8.9 Hz, 1H). **^{13}C{^{1}H} NMR** (101 MHz, DMSO-d_6) δ 172.7, 164.6, 155.7, 140.1 (q, *J* = 37.8 Hz), 140.0, 137.2, 133.9, 132.0, 131.5, 130.3, 129.8, 129.5, 129.3, 128.0, 125.6, 123.4, 120.5, 118.7 (q, *J* = 271.8 Hz), 114.8, 111.4, 54.5, 36.3. **^{19}F NMR** (376 MHz, DMSO-d_6) δ -59.61 (s). **^{19}F NMR** (76 MHz, THF) δ -60.78 (s). **HRMS** (ESI): *m/z* $[M + H]^+$ Calcd for $C_{24}H_{20}F_3N_4O_3$ 469.1482; Found 469.1483.

(M)-TBBA-(*L*)-Tyr-NH_2 **(*M*)-14**

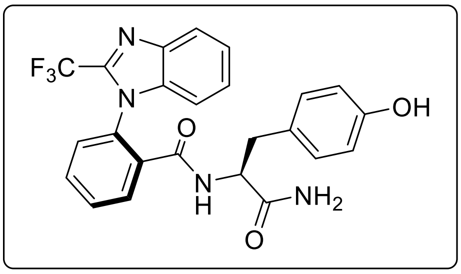

Following the general procedure, from Fmoc-Tyr(*t*Bu)-OH (0.38 mmol; 1 equiv; 175 mg) and (*M*)-TBBA, reaction time 40 hours, with purification by CC (CH_2Cl_2/MeOH 15:1; column dimensions 4 × 10 cm), 31 mg (19% yield) of white solid was obtained. **^{1}H NMR** (400 MHz, DMSO-d_6) δ 9.14 (s, 1H), 8.46 (d, *J* = 8.4 Hz, 1H), 7.83 (dd, *J* = 7.0, 1.4 Hz, 1H), 7.75 – 7.68 (m, 3H), 7.62 – 7.58 (m, 1H), 7.39 – 7.31 (m, 2H), 7.05 – 6.97 (m, 3H), 6.89 (br. s, 1H), 6.82 (br. s, 1H), 6.67 – 6.62 (m, 2H), 4.21 – 4.13 (m, 1H), 2.85 (dd, *J* = 13.9, 4.7 Hz, 1H), 2.69 (dd, *J* = 13.9, 9.7 Hz, 1H). **^{13}C{^{1}H} NMR** (101 MHz, DMSO-d_6) δ 172.5, 164.6, 155.8, 140.1 (q, *J* = 37.7 Hz), 140.0, 137.1, 133.9, 132.0, 131.6, 130.3, 129.9, 129.5, 129.2, 127.9, 125.6, 123.6, 120.5, 118.7 (q, *J* = 271.8 Hz), 114.8, 111.4, 54.4, 36.5. **^{19}F NMR** (376 MHz, DMSO-d_6) δ -59.63 (s). **^{19}F NMR** (76 MHz, THF) δ -60.63 (s). **HRMS** (ESI): *m/z* $[M + H]^+$ Calcd for $C_{24}H_{20}F_3N_4O_3$ 469.1482; Found 469.1487.

(*P*)-TBBA-(*L*)-Trp-NH2 ***(P)*-15**

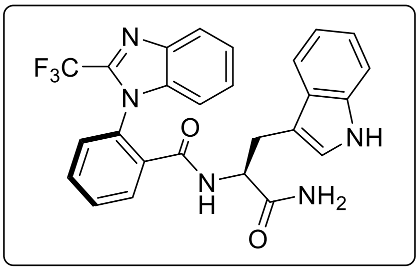

Following the general procedure, from Fmoc-Trp(Boc)-OH (0.38 mmol; 1 equiv; 200 mg) and (*P*)-TBBA, reaction time 16 hours, with purification by semipreparative HPLC (CH_3CN/H_2O + ammonium acetate, gradient: 40% CH_3CN to 60% CH_3CN), 41 mg (20% yield) of white solid was obtained. **^{1}H NMR** (400 MHz, DMSO-d_6) δ 10.78 – 10.66 (m, 1H), 8.56 (d, *J* = 8.0 Hz, 1H), 7.84 – 7.77 (m, 2H), 7.76 – 7.69 (m, 2H), 7.64 – 7.59 (m, 1H), 7.38 (d, *J* = 7.8 Hz, 1H), 7.33 – 7.27 (m, 2H), 7.25 (br. s, 1H), 7.22 (td, *J* = 7.7, 7.2, 1.1 Hz, 1H), 7.04 (ddd, *J* = 8.1, 7.1, 1.1 Hz, 1H), 7.00 – 6.89 (m, 4H), 4.21 (td, *J* = 8.1, 6.2 Hz, 1H), 2.94 (dd, *J* = 14.7, 6.0 Hz, 1H), 2.71 (dd, *J* = 14.7, 8.2 Hz, 1H). **^{13}C{^{1}H} NMR** (101 MHz, DMSO-d_6) δ 172.9, 164.6, 140.1 (q, *J* = 38.0 Hz), 140.0, 137.1, 136.0, 134.1, 131.9, 131.5, 130.2, 129.4, 129.2, 127.2, 125.5, 123.5, 123.2, 120.8, 120.5, 118.8 (q, *J* = 271.9 Hz), 118.3, 118.1, 111.4, 111.2, 110.2, 53.6, 27.2. **^{19}F NMR** (376 MHz, DMSO-d_6) δ -59.57 (s). **^{19}F NMR** (76 MHz, THF) δ -60.76 (s). **HRMS** (ESI): *m/z* [M + H]$^+$ Calcd for $C_{26}H_{21}F_3N_5O_2$ 492.1642; Found 492.1635.

(M)-TBBA-(*L*)-Trp-NH2 **(*M*)-15**

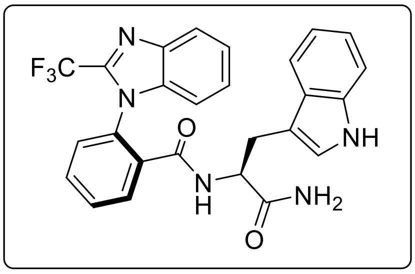

Following the general procedure, from Fmoc-Trp(Boc)-OH (0.38 mmol; 1 equiv; 200 mg) and (*M*)-TBBA, reaction time 40 hours, with purification by semipreparative HPLC (CH_3CN/H_2O + ammonium acetate, gradient: 40% CH_3CN to 60% CH_3CN), 21 mg (18% yield) of white solid was obtained. **^{1}H NMR** (400 MHz, DMSO-d_6) δ 10.80 (d, *J* = 1.6 Hz, 1H), 8.45 (d, *J* = 8.3 Hz, 1H), 7.88 – 7.79 (m, 1H), 7.74 – 7.65 (m, 3H), 7.60 (d, *J* = 8.0 Hz, 1H), 7.54 (d, *J* = 7.9 Hz, 1H), 7.39 – 7.30 (m, 3H), 7.11 (d, *J* = 2.3 Hz, 1H), 7.08 – 7.03 (m, 2H), 6.96 (ddd, *J* = 7.9, 7.0, 1.0 Hz, 1H), 6.92 (br. s, 1H), 6.83 (br. s, 1H), 4.29 (td, *J* = 8.6, 5.2 Hz, 1H), 3.08 (dd, *J* = 14.8, 5.1 Hz, 1H), 2.94 (dd, *J* = 14.7, 8.8 Hz, 1H). **^{13}C{^{1}H} NMR** (101 MHz, DMSO-d_6) δ 172.7, 164.5, 140.1 (q, *J* = 38.0 Hz), 140.0, 137.0, 136.1, 133.8, 132.0, 131.6, 130.2, 129.5, 129.2, 127.3, 125.7, 123.6, 123.3, 120.9, 120.6, 118.7 (q, *J* = 271.6 Hz), 118.3, 118.2, 111.3, 111.3, 110.1, 53.5, 27.5. **^{19}F NMR** (376 MHz, DMSO-d_6) δ -59.62 (s). **^{19}F NMR** (76 MHz, THF) δ -60.61 (s). **HRMS** (ESI): *m/z* [M + H]$^+$ Calcd for $C_{26}H_{21}F_3N_5O_2$ 492.1642; Found 492.1639.

(P)-TBBA-(L)-His-NH_2 **(P)-16**

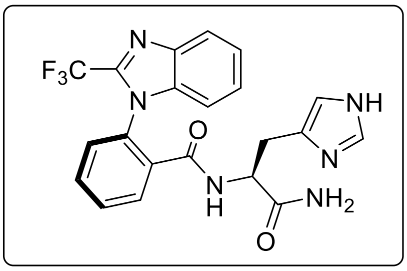

Following the general procedure, from Fmoc-His(Trt)-OH (0.38 mmol; 1 equiv; 235 mg) and (P)-TBBA, reaction time 16 hours, with purification by CC (CH_2Cl_2/MeOH 15:1; column dimensions 4 × 10 cm) and removal of trifluoroacetate salt by trimethylamine, 48 mg (94% yield) of white solid was obtained. **^{1}H NMR** (400 MHz, DMSO-d_6) δ 11.78 (br. s, 1H), 8.59 (d, J = 7.7 Hz, 1H), 7.92 – 7.87 (m, 1H), 7.84 (d, J = 8.1 Hz, 1H), 7.79 – 7.73 (m, 2H), 7.66 – 7.61 (m, 1H), 7.51 (s, 1H), 7.36 (td, J = 7.2, 1.4 Hz, 1H), 7.29 (td, J = 7.2, 1.0 Hz, 1H), 7.15 (br. s, 1H), 6.98 (d, J = 8.1 Hz, 1H), 6.95 (br. s, 1H), 6.64 (s, 1H), 4.11 (td, J = 7.9, 5.9 Hz, 1H), 2.72 (dd, J = 14.8, 5.6 Hz, 1H), 2.62 (dd, J = 14.8, 8.1 Hz, 1H).; Carbon was measured as the trifluoroacetate salt. **^{13}C{^{1}H} NMR** (101 MHz, DMSO-d_6) δ 171.9, 164.9, 158.2 (q, J = 30.9 Hz), 140.2 (q, J = 37.7 Hz), 140.0, 137.0, 134.0, 133.8, 131.9, 131.7, 130.9, 130.3, 129.5, 129.4, 125.5, 123.5, 120.6, 118.7 (q, J = 271.8 Hz), 117.3 (q, J = 300.0 Hz), 116.4, 111.1, 52.4, 27.3. **^{19}F NMR** (376 MHz, DMSO-d_6) δ -59.57 (s). **^{19}F NMR** (76 MHz, THF) δ -60.56 (s). **HRMS** (ESI): m/z $[M + H]^+$ Calcd for $C_{21}H_{18}F_3N_6O_2$ 443.1438; Found 443.1437.

(M)-TBBA-(L)-His-NH_2 **(M)-16**

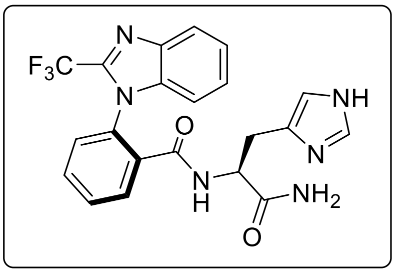

Following the general procedure, from Fmoc-His(Trt)-OH (0.38 mmol; 1 equiv; 235 mg) and (M)-TBBA, reaction time 32 hours, with purification by CC (CH_2Cl_2/MeOH 15:1; column dimensions 4 × 10 cm) and removal of trifluoroacetate salt by trimethylamine, 18 mg (35% yield) of white solid was obtained. **^{1}H NMR** (400 MHz, DMSO-d_6) δ 11.85 (s, 1H), 8.58 (d, J = 8.0 Hz, 1H), 7.89 – 7.81 (m, 2H), 7.79 – 7.73 (m, 2H), 7.66 – 7.61 (m, 1H), 7.56 (s, 1H), 7.36 (pd, J = 7.2, 1.2 Hz, 2H), 7.07 (dd, J = 7.1, 1.5 Hz, 1H), 6.92 – 6.68 (m, 3H), 4.20 (td, J = 8.0, 5.5 Hz, 1H), 2.88 – 2.72 (m, 2H). **^{13}C{^{1}H} NMR** (101 MHz, DMSO-d_6) δ 172.3, 164.5, 140.0 (q, J = 37.6 Hz), 140.0, 137.1, 134.7, 133.9, 132.0, 131.7, 130.4, 129.6, 129.3, 125.6, 123.6, 120.6, 118.7 (q, J = 272.0 Hz), 116.3, 111.4, 53.2, 29.2. **^{19}F NMR** (376 MHz, DMSO-d_6) δ -59.67 (s). **^{19}F NMR** (76 MHz, THF) δ -60.63 (s). **HRMS** (ESI): m/z $[M + H]^+$ Calcd for $C_{21}H_{18}F_3N_6O_2$ 443.1438; Found 443.1439.

(P)-TBBA-(*L*)-Ser-NH$_2$ ***(P)*-17**

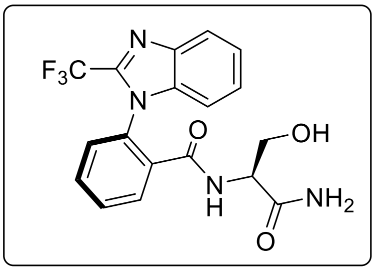

Following the general procedure, from Fmoc-Ser(*t*Bu)-OH (0.38 mmol; 1 equiv; 146 mg) and (*P*)-TBBA, reaction time 16 hours, with purification by CC (CH_2Cl_2/MeOH 15:1; column dimensions 4 × 10 cm), 36 mg (47% yield) of white solid was obtained. **^{1}H NMR** (400 MHz, DMSO-*d*$_6$) δ 8.28 (d, *J* = 8.0 Hz, 1H), 7.96 – 7.90 (m, 1H), 7.88 – 7.82 (m, 1H), 7.79 – 7.72 (m, 2H), 7.68 – 7.61 (m, 1H), 7.42 – 7.34 (m, 2H), 7.21 (br. s, 1H), 7.14 – 7.09 (m, 1H), 7.05 (br. s, 1H), 4.66 (br. s, 1H), 3.92 (dt, *J* = 8.0, 5.6 Hz, 1H), 3.28 – 3.20 (m, 2H). **^{13}C{^{1}H} NMR** (101 MHz, DMSO-*d*$_6$) δ 171.3, 164.8, 140.0, 140.0 (q, *J* = 37.8 Hz), 137.3, 133.9, 132.0, 131.7, 130.4, 129.6, 125.7, 123.6, 120.6, 118.8 (q, *J* = 273.4, 272.7 Hz), 111.5, 61.5, 55.5. **^{19}F NMR** (376 MHz, DMSO-*d*$_6$) δ -59.56 (s). **^{19}F NMR** (76 MHz, THF) δ -60.73 (s). **HRMS** (ESI): *m/z* $[M + H]^+$ Calcd for $C_{18}H_{16}F_3N_4O_3$ 393.1169; Found 393.1165.

(M)-TBBA-(*L*)-Ser-NH$_2$ ***(M)*-17**

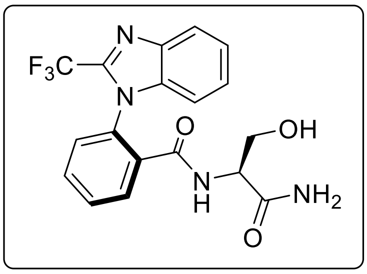

Following the general procedure, from Fmoc-Ser(*t*Bu)-OH (0.38 mmol; 1 equiv; 146 mg) and (*M*)-TBBA, reaction time 40 hours, with purification by CC (CH_2Cl_2/MeOH 15:1; column dimensions 4 × 10 cm), 29 mg (42% yield) of white solid was obtained. **^{1}H NMR** (400 MHz, DMSO-*d*$_6$) δ 8.25 (d, *J* = 8.0 Hz, 1H), 8.02 – 7.94 (m, 1H), 7.88 – 7.82 (m, 1H), 7.80 – 7.74 (m, 2H), 7.68 – 7.61 (m, 1H), 7.40 – 7.32 (m, 2H), 7.12 – 7.06 (m, 1H), 6.94 (br. s, 2H), 4.85 (br. s, 1H), 4.00 (dt, *J* = 8.0, 5.4 Hz, 1H), 3.55 – 3.47 (m, 2H). **^{13}C{^{1}H} NMR** (101 MHz, DMSO-*d*$_6$) δ 171.5, 164.9, 140.2 (q, *J* = 37.8 Hz), 140.1, 137.0, 134.2, 131.8, 131.5, 130.3, 129.3, 129.3, 125.5, 123.6, 120.6, 118.8 (q, *J* = 271.8 Hz), 111.5, 61.2, 55.1. **^{19}F NMR** (376 MHz, DMSO-*d*$_6$) δ -59.64 (s). **^{19}F NMR** (76 MHz, THF) δ -60.68 (s). **HRMS** (ESI): *m/z* $[M + H]^+$ Calcd for $C_{18}H_{16}F_3N_4O_3$ 393.1169; Found 393.1166.

(P)-TBBA-(*L*)-Asp(OMe)-NH$_2$ ***(P)*-18**

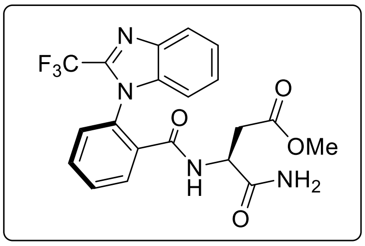

Following the general procedure, from Fmoc-Asp(OMe)-OH (0.38 mmol; 1 equiv; 140 mg) and (*P*)-TBBA, reaction time 32 hours, with purification by CC (CH_2Cl_2/MeOH 18:1; column dimensions 4 × 10 cm), 69 mg (78% yield) of white solid was obtained. **^{1}H NMR** (400 MHz, DMSO-d_6) δ 8.74 (d, J = 8.2 Hz, 1H), 7.93 – 7.88 (m, 1H), 7.88 – 7.84 (m, 1H), 7.80 – 7.73 (m, 2H), 7.68 – 7.62 (m, 1H), 7.43 – 7.34 (m, 2H), 7.26 (br. s, 1H), 7.10 (br. s, 1H), 7.07 – 7.02 (m, 1H), 4.31 (dt, J = 7.9, 6.9 Hz, 1H), 3.45 (s, 3H), 2.37 (dd, J = 16.2, 7.1 Hz, 1H), 1.99 (dd, J = 16.2, 6.7 Hz, 1H). **^{13}C{^{1}H} NMR** (101 MHz, DMSO-d_6) δ 171.4, 170.3, 164.9, 140.3 (q, J = 38.0 Hz), 140.1, 136.9, 134.1, 131.9, 131.6, 130.2, 129.4, 129.3, 125.6, 123.6, 120.7, 118.7 (q, J = 271.8 Hz), 111.3, 51.3, 49.2, 35.0. **^{19}F NMR** (376 MHz, DMSO-d_6) δ -59.58 (s). **^{19}F NMR** (76 MHz, THF) δ -60.83 (s). **HRMS** (ESI): *m/z* [M + H]$^+$ Calcd for $C_{20}H_{18}F_3N_4O_4$ 435.1275; Found 435.1275.

(M)-TBBA-(*L*)-Asp(OMe)-NH$_2$ ***(M)*-18**

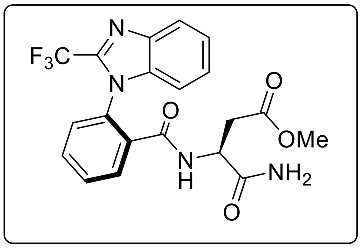

Following the general procedure, from Fmoc-Asp(OMe)-OH (0.38 mmol; 1 equiv; 140 mg) and (*M*)-TBBA, reaction time 64 hours, with purification by CC (CH_2Cl_2/MeOH 18:1; column dimensions 4 × 10 cm), 68 mg (77% yield) of white solid was obtained. **^{1}H NMR** (400 MHz, DMSO-d_6) δ 8.72 (d, J = 8.1 Hz, 1H), 7.94 – 7.89 (m, 1H), 7.88 – 7.84 (m, 1H), 7.80 – 7.74 (m, 2H), 7.67 – 7.62 (m, 1H), 7.43 – 7.30 (m, 2H), 7.07 – 7.03 (m, 1H), 6.97 (br. s, 1H), 6.91 (br. s, 1H), 4.37 (td, J = 7.9, 5.9 Hz, 1H), 3.54 (s, 3H), 2.62 (dd, J = 16.1, 5.9 Hz, 1H), 2.41 (dd, J = 16.1, 7.9 Hz, 1H). **^{13}C{^{1}H} NMR** (101 MHz, DMSO-d_6) δ 171.4, 170.5, 164.8, 140.0, 139.9 (q, J = 37.9 Hz), 137.4, 133.7, 132.0, 131.8, 130.3, 129.6, 129.6, 125.6, 123.6, 120.6, 118.7 (q, J = 272.0 Hz), 111.5, 51.3, 49.5, 35.4. **^{19}F NMR** (376 MHz, DMSO-d_6) δ -59.72 (s). **^{19}F NMR** (76 MHz, THF) δ -60.68 (s). **HRMS** (ESI): *m/z* [M + H]$^+$ Calcd for $C_{20}H_{18}F_3N_4O_4$ 435.1275; Found 435.1277.

(P)-TBBA-(*L*)-Asn-NH$_2$ ***(P)*-19**

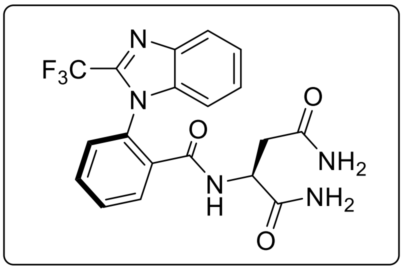

Following the general procedure, from Fmoc-Asn-OH (0.38 mmol; 1 equiv; 134 mg) and (*P*)-TBBA, reaction time 16 hours, with purification by CC (CH_2Cl_2/MeOH 20:1; column dimensions 4 × 10 cm) and removal of trifluoroacetate salt by trimethylamine, 36 mg (38% yield) of white solid was obtained. **^{1}H NMR** (400 MHz, DMSO-d_6) δ 8.58 (d, *J* = 8.1 Hz, 1H), 7.94 – 7.89 (m, 1H), 7.88 – 7.84 (m, 1H), 7.79 – 7.74 (m, 2H), 7.67 – 7.62 (m, 1H), 7.41 – 7.36 (m, 2H), 7.19 (br. s, 1H), 7.07 – 7.03 (m, 1H), 7.02 (br. s, *J* = 7.4 Hz, 1H), 6.98 (br. s, 1H), 6.77 (br. s, 1H), 4.23 (q, *J* = 6.7 Hz, 1H), 2.27 (dd, *J* = 15.2, 6.5 Hz, 1H), 2.08 (dd, *J* = 15.2, 6.9 Hz, 1H). **^{13}C{^{1}H} NMR** (101 MHz, DMSO-d_6) δ 172.2, 171.4, 164.6, 140.1 (q, *J* = 37.8 Hz), 140.1, 137.1, 134.0, 131.9, 131.6, 130.3, 129.5, 129.4, 125.7, 123.6, 120.6, 118.8 (q, *J* = 271.9 Hz), 111.4, 49.8, 36.5. **^{19}F NMR** (376 MHz, DMSO-d_6) δ -59.55 (s). **^{19}F NMR** (76 MHz, THF) δ -60.81 (s). **HRMS** (ESI): *m/z* [M + H]$^+$ Calcd for $C_{19}H_{17}F_3N_5O_3$ 420.1278; Found 420.1278.

(M)-TBBA-(*L*)-Asn-NH$_2$ ***(M)*-19**

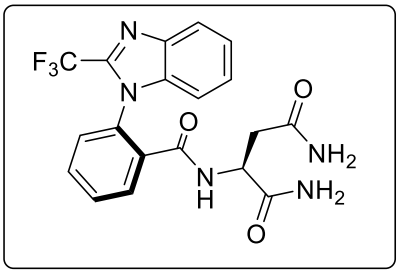

Following the general procedure, from Fmoc-Asn-OH (0.38 mmol; 1 equiv; 134 mg) and (*M*)-TBBA, reaction time 40 hours, with purification by CC (CH_2Cl_2/MeOH 20:1; column dimensions 4 × 10 cm) and removal of trifluoroacetate salt by trimethylamine, 19 mg (20% yield) of white solid was obtained. **^{1}H NMR** (400 MHz, DMSO-d_6) δ 8.57 (d, *J* = 8.1 Hz, 1H), 7.92 – 7.88 (m, 1H), 7.86 (dd, *J* = 6.9, 1.5 Hz, 1H), 7.80 – 7.74 (m, 2H), 7.68 – 7.62 (m, 1H), 7.41 – 7.33 (m, 2H), 7.28 (br. s, 1H), 7.08 (dd, *J* = 7.0, 1.6 Hz, 1H), 6.86 (br. s, 2H), 6.68 (br. s, 1H), 4.30 (td, *J* = 7.6, 5.9 Hz, 1H), 2.41 (dd, *J* = 15.2, 5.7 Hz, 1H), 2.31 (dd, *J* = 15.2, 7.5 Hz, 1H). **^{13}C{^{1}H} NMR** (101 MHz, DMSO-d_6) δ 172.1, 171.5, 164.6, 140.1 (d, *J* = 38.0 Hz), 140.0, 137.2, 133.8, 132.0, 131.7, 130.3, 129.6, 129.4, 125.6, 123.6, 120.6, 118.7 (q, *J* = 272.0 Hz), 111.4, 49.9, 36.7. **^{19}F NMR** (376 MHz, DMSO-d_6) δ -59.67 (s). **^{19}F NMR** (76 MHz, THF) δ -60.69 (s). **HRMS** (ESI): *m/z* [M + H]$^+$ Calcd for $C_{19}H_{17}F_3N_5O_3$ 420.1278; Found 420.1277.

(P)-TBBA-(*L*)-Ala-(*L*)-Ala-NH_2 ***(P)*-20**

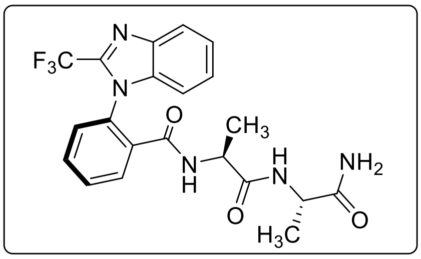

Following the general procedure, Fmoc-Ala-OH (0.38 mmol; 1 equiv; 118 mg) was attached to Rink resin. Then the second Fmoc-Ala-OH (0.38 mmol; 1 equiv; 118 mg) was attached to the resin-bound amino acid. The dipeptide underwent acylation with (*P*)-TBBA for 16 hours. After cleavage from the resin, the crude product was purified by CC (CH_2Cl_2/MeOH 15:1; column dimensions 4 × 10 cm), 112 mg (60% yield) of white solid was obtained. **^{1}H NMR** (400 MHz, DMSO-d_6) δ 8.56 (d, *J* = 7.7 Hz, 1H), 7.88 – 7.84 (m, 1H), 7.84 – 7.79 (m, 2H), 7.78 – 7.72 (m, 2H), 7.69 – 7.63 (m, 1H), 7.42 – 7.34 (m, 2H), 7.22 (br. s, 1H), 7.17 – 7.11 (m, 1H), 6.94 (br. s, 1H), 4.14 (p, *J* = 7.1 Hz, 1H), 4.00 (p, *J* = 7.1 Hz, 1H), 1.17 (d, *J* = 7.1 Hz, 3H), 0.86 (d, *J* = 7.1 Hz, 3H). **^{13}C{^{1}H} NMR** (101 MHz, DMSO-d_6) δ 174.0, 171.1, 164.9, 140.4 (q, *J* = 37.7 Hz), 140.2, 136.8, 134.7, 131.7, 131.3, 130.3, 129.2, 129.0, 125.5, 123.7, 120.7, 118.7 (q, *J* = 272.1 Hz), 111.5, 48.1, 47.9, 18.2, 17.3. **^{19}F NMR** (376 MHz, DMSO-d_6) δ -59.53 (s). **^{19}F NMR** (76 MHz, THF) δ -60.74 (s). **HRMS** (ESI): *m/z* $[M + H]^+$ Calcd for $C_{21}H_{21}F_3N_5O_3$ 448.1591; Found 448.1586.

(M)-TBBA-(*L*)-Ala-(*L*)-Ala-NH_2 ***(M)*-20**

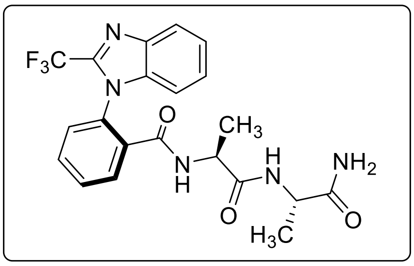

Following the general procedure, Fmoc-Ala-OH (0.38 mmol; 1 equiv; 118 mg) was attached to Rink resin. Then the second Fmoc-Ala-OH (0.38 mmol; 1 equiv; 118 mg) was attached to the resin-bound amino acid. The dipeptide underwent acylation with (*M*)-TBBA for 40 hours. After cleavage from the resin, the crude product was purified by CC (CH_2Cl_2/MeOH 15:1; column dimensions 4 × 10 cm), 70 mg (38% yield) of white solid was obtained. **^{1}H NMR** (400 MHz, DMSO-d_6) δ 8.58 (d, *J* = 7.7 Hz, 1H), 7.94 – 7.87 (m, 1H), 7.87 – 7.83 (m, 1H), 7.80 – 7.74 (m, 2H), 7.72 (d, *J* = 7.5 Hz, 1H), 7.66 – 7.61 (m, 1H), 7.41 – 7.33 (m, 2H), 7.23 (br. s, 1H), 7.07 – 6.99 (m, 1H), 6.94 (br. s, 1H), 4.13 (p, *J* = 7.1 Hz, 1H), 4.05 (p, *J* = 7.1 Hz, 1H), 1.11 (d, *J* = 7.1 Hz, 3H), 1.06 (d, *J* = 7.1 Hz, 3H). **^{13}C{^{1}H} NMR** (101 MHz, DMSO-d_6) δ 174.0, 171.0, 164.5, 140.0, 139.8 (q, *J* = 37.8 Hz), 137.4, 133.9, 131.9, 131.6, 130.4, 129.6, 129.4, 125.7, 123.6, 120.5, 118.8 (q, *J* = 271.8 Hz), 111.6, 48.4, 47.9, 18.3, 17.5. **^{19}F NMR** (376 MHz, DMSO-d_6) δ -59.65 (s). **^{19}F NMR** (76 MHz, THF) δ -60.64 (s). **HRMS** (ESI): *m/z* $[M + H]^+$ Calcd for $C_{21}H_{21}F_3N_5O_3$ 448.1591; Found 448.1588.

(P)-TBBA-(*L*)-Ala-(*L*)-Phe-NH_2 ***(P)*-21**

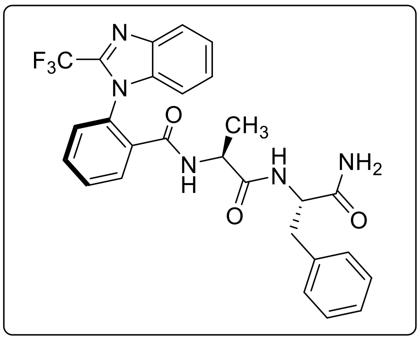

Following the general procedure, Fmoc-Phe-OH (0.38 mmol; 1 equiv; 147 mg) was attached to Rink resin. Then Fmoc-Ala-OH (0.38 mmol; 1 equiv; 118 mg) was attached to the resin-bound amino acid. The dipeptide underwent acylation with (*P*)-TBBA for 16 hours. After cleavage from the resin, the crude product was purified by CC (CH_2Cl_2/MeOH 20:1; column dimensions 4 × 10 cm), 55 mg (25%) of white solid was obtained. **^{1}H NMR** (400 MHz, DMSO-*d*$_6$) δ 8.47 (d, *J* = 7.8 Hz, 1H), 7.89 – 7.81 (m, 2H), 7.79 – 7.69 (m, 3H), 7.68 – 7.63 (m, 1H), 7.41 – 7.34 (m, 2H), 7.33 (br. s, *J* = 4.7 Hz, 1H), 7.25 – 7.15 (m, 5H), 7.15 – 7.11 (m, 1H), 7.02 (br. s, 1H), 4.36 (td, *J* = 8.6, 5.0 Hz, 1H), 3.95 (p, *J* = 7.1 Hz, 1H), 2.97 (dd, *J* = 13.8, 5.0 Hz, 1H), 2.80 (dd, *J* = 13.8, 8.9 Hz, 1H), 0.77 (d, *J* = 7.0 Hz, 3H). **^{13}C{^{1}H} NMR** (101 MHz, DMSO-*d*$_6$) δ 172.7, 171.3, 164.8, 140.4 (q, *J* = 37.9 Hz), 140.2, 137.8, 136.7, 134.6, 131.7, 131.3, 130.2, 129.2, 129.1, 129.0, 128.0, 126.2, 125.4, 123.7, 120.6, 118.7 (q, *J* = 272.0 Hz), 111.4, 53.7, 48.2, 37.3, 17.1. **^{19}F NMR** (376 MHz, DMSO-*d*$_6$) δ -59.52 (s). **^{19}F NMR** (76 MHz, THF) δ -60.71 (s). **HRMS** (ESI): *m/z* $[M + H]^+$ Calcd for $C_{27}H_{25}F_3N_5O_3$ 524.1904; Found 524.1900.

(M)-TBBA-(*L*)-Ala-(*L*)-Phe-NH_2 ***(M)*-21**

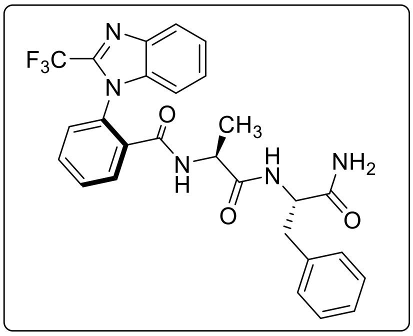

Following the general procedure, Fmoc-Phe-OH (0.38 mmol; 1 equiv; 147 mg) was attached to Rink resin. Then Fmoc-Ala-OH (0.38 mmol; 1 equiv; 118 mg) was attached to the resin-bound amino acid. The dipeptide underwent acylation with (*M*)-TBBA for 40 hours. After cleavage from the resin, the crude product was purified by CC (CH_2Cl_2/MeOH 20:1; column dimensions 4 × 10 cm), 78 mg (36%) of white solid was obtained. **^{1}H NMR** (400 MHz, DMSO-*d*$_6$) δ 8.49 (d, *J* = 7.6 Hz, 1H), 7.86 – 7.82 (m, 2H), 7.79 – 7.74 (m, 2H), 7.72 (d, *J* = 8.1 Hz, 1H), 7.66 – 7.62 (m, 1H), 7.36 (dt, *J* = 7.2, 3.6 Hz, 2H), 7.32 (br. s, 1H), 7.18 – 7.10 (m, 5H), 7.06 – 6.97 (m, 2H), 4.32 (td, *J* = 8.2, 5.4 Hz, 1H), 4.09 (p, *J* = 7.1 Hz, 1H), 2.88 (dd, *J* = 13.7, 5.3 Hz, 1H), 2.73 (dd, *J* = 13.7, 8.3 Hz, 1H), 1.03 (d, *J* = 7.1 Hz, 3H). **^{13}C{^{1}H} NMR** (101 MHz, DMSO-*d*$_6$) δ 172.5, 171.2, 164.4, 140.0, 139.7 (q, *J* = 37.6 Hz), 137.6, 137.5, 133.9, 131.9, 131.7, 130.3, 129.6, 129.4, 129.2, 127.9, 126.1, 125.7, 123.5, 120.6, 118.8 (q, *J* = 271.7 Hz), 111.7, 53.5, 48.6, 37.4, 17.4. **^{19}F NMR** (376 MHz, DMSO-*d*$_6$) δ -59.68 (s). **^{19}F NMR** (76 MHz, THF) δ -60.67 (s). **HRMS** (ESI): *m/z* $[M + H]^+$ Calcd for $C_{27}H_{25}F_3N_5O_3$ 524.1904; Found 524.1903.

(P)-TBBA-(*L*)-Ala-(*D*)-Phe-NH_2 ***(P)*-22**

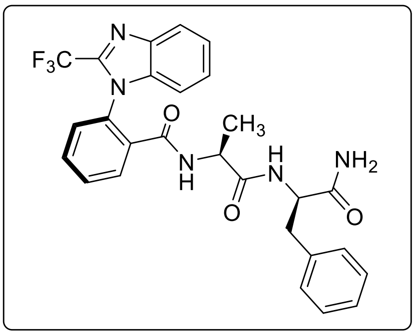

Following the general procedure, Fmoc-*D*-Phe-OH (0.38 mmol; 1 equiv; 147 mg) was attached to Rink resin. Then Fmoc-Ala-OH (0.38 mmol; 1 equiv; 118 mg) was attached to the resin-bound amino acid. The dipeptide underwent acylation with (*P*)-TBBA for 16 hours. After cleavage from the resin, the crude product was purified by CC (CH_2Cl_2/MeOH 20:1; column dimensions 4 × 10 cm), 72 mg (83% yield) of white solid was obtained. **^{1}H NMR** (400 MHz, DMSO-d_6) δ 8.46 (d, *J* = 7.4 Hz, 1H), 8.05 (d, *J* = 8.7 Hz, 1H), 7.87 – 7.84 (m, 1H), 7.76 – 7.71 (m, 3H), 7.69 – 7.61 (m, 1H), 7.40 – 7.33 (m, 2H), 7.30 (br. s, 1H), 7.22 – 7.12 (m, 6H), 7.10 (br. s, 1H), 4.39 (ddd, *J* = 10.3, 8.8, 4.3 Hz, 1H), 3.93 (p, *J* = 7.0 Hz, 1H), 3.03 (dd, *J* = 13.7, 4.2 Hz, 1H), 2.69 (dd, *J* = 13.7, 10.3 Hz, 1H), 0.62 (d, *J* = 7.1 Hz, 3H). **^{13}C{^{1}H} NMR** (101 MHz, DMSO-*d6*) δ 172.8, 171.4, 164.8, 140.4 (q, *J* = 38.0 Hz), 140.1, 138.0, 136.7, 134.6, 131.7, 131.3, 130.2, 129.1, 128.9, 127.9, 126.1, 125.4, 123.7, 120.6, 118.7 (q, *J* = 271.8 Hz), 111.5, 53.5, 48.3, 37.4, 17.3. **^{19}F NMR** (376 MHz, DMSO-d_6) δ -59.48 (s). **^{19}F NMR** (76 MHz, THF) δ -60.75 (s). **HRMS** (ESI): *m/z* $[M + H]^+$ Calcd for $C_{27}H_{25}F_3N_5O_3$ 524.1904; Found 524.1909.

(M)-TBBA-(*L*)-Ala-(*D*)-Phe-NH_2 ***(M)*-22**

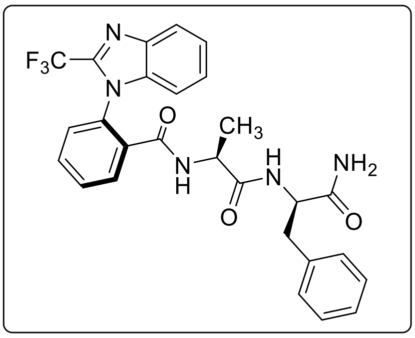

Following the general procedure, Fmoc-*D*-Phe-OH (0.38 mmol; 1 equiv; 147 mg) was attached to Rink resin. Then Fmoc-Ala-OH (0.38 mmol; 1 equiv; 118 mg) was attached to the resin-bound amino acid. The dipeptide underwent acylation with (*M*)-TBBA for 40 hours. After cleavage from the resin, the crude product was purified by CC (CH_2Cl_2/MeOH 20:1; column dimensions 4 × 10 cm), 39 mg (45% yield) of white solid was obtained. **^{1}H NMR** (400 MHz, DMSO-d_6) δ 8.40 (d, *J* = 7.1 Hz, 1H), 7.96 (d, *J* = 8.6 Hz, 1H), 7.89 – 7.80 (m, 2H), 7.78 – 7.71 (m, 2H), 7.66 – 7.59 (m, 1H), 7.42 – 7.34 (m, 2H), 7.24 – 7.11 (m, 6H), 7.06 – 6.99 (m, 1H), 6.94 (br. s, 1H), 4.34 (td, *J* = 9.9, 4.3 Hz, 1H), 4.07 (p, *J* = 6.9 Hz, 1H), 3.00 (dd, *J* = 13.7, 4.2 Hz, 1H), 2.66 (dd, *J* = 13.6, 10.2 Hz, 1H), 0.87 (d, *J* = 7.1 Hz, 3H). **^{13}C{^{1}H} NMR** (101 MHz, DMSO-d_6) δ 172.8, 171.4, 164.5, 140.0, 139.7 (q, *J* = 38.0 Hz), 137.9, 137.6, 133.9, 131.9, 131.7, 130.4, 129.7, 129.4, 129.1, 127.9, 126.2, 125.7, 123.6, 120.6, 118.8 (q, *J* = 271.7 Hz), 111.8, 53.6, 48.6, 37.4, 17.5. **^{19}F NMR** (376 MHz, DMSO-d_6) δ -59.70 (s). **^{19}F NMR** (76 MHz, THF) δ -60.72 (s). **HRMS** (ESI): *m/z* $[M + H]^+$ Calcd for $C_{27}H_{25}F_3N_5O_3$ 524.1904; Found 524.1907.

(P)-TBBA-(*D*)-Ala-(*L*)-Phe-NH$_2$ **(*P*)-23**

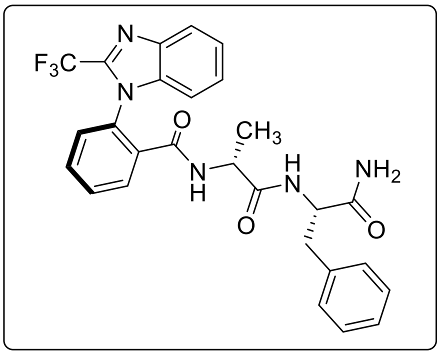

Following the general procedure, Fmoc-Phe-OH (0.38 mmol; 1 equiv; 147 mg) was attached to Rink resin. Then Fmoc-*D*-Ala-OH (0.38 mmol; 1 equiv; 118 mg) was attached to the resin-bound amino acid. The dipeptide underwent acylation with (*P*)-TBBA for 16 hours. After cleavage from the resin, the crude product was purified by CC (CH_2Cl_2/MeOH 20:1; column dimensions 4 × 10 cm), 64 mg (74% yield) of white solid was obtained. **^{1}H NMR** (400 MHz, DMSO-*d*$_6$) δ 8.40 (d, *J* = 7.2 Hz, 1H), 7.96 (d, *J* = 8.6 Hz, 1H), 7.88 – 7.83 (m, 2H), 7.78 – 7.73 (m, 2H), 7.66 – 7.61 (m, 1H), 7.40 – 7.35 (m, 2H), 7.22 – 7.12 (m, 6H), 7.07 – 7.01 (m, 1H), 6.95 (br. s, 1H), 4.35 (td, *J* = 9.5, 4.3 Hz, 1H), 4.08 (p, *J* = 7.1 Hz, 1H), 3.00 (dd, *J* = 13.7, 4.3 Hz, 1H), 2.66 (dd, *J* = 13.7, 10.1 Hz, 1H), 0.87 (d, *J* = 7.1 Hz, 3H). **^{13}C{^{1}H} NMR** (101 MHz, DMSO-*d*$_6$) δ 172.7, 171.4, 164.5, 139.9, 139.7 (q, *J* = 38.0 Hz), 137.9, 137.6, 133.9, 131.9, 131.7, 130.3, 129.6, 129.4, 129.1, 127.9, 126.1, 125.7, 123.6, 120.5, 118.8 (q, *J* = 271.8 Hz), 111.8, 53.5, 48.5, 37.4, 17.5. **^{19}F NMR** (376 MHz, DMSO-*d*$_6$) δ -59.70 (s). **^{19}F NMR** (76 MHz, THF) δ -60.73 (s). **HRMS** (ESI): *m/z* $[M + H]^+$ Calcd for $C_{27}H_{25}F_3N_5O_3$ 524.1904; Found 524.1908.

(M)-TBBA-(*D*)-Ala-(*L*)-Phe-NH$_2$ **(*M*)-23**

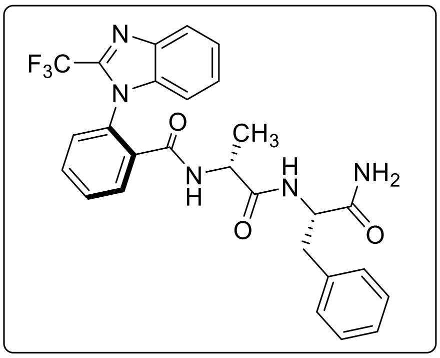

Following the general procedure, Fmoc-Phe-OH (0.38 mmol; 1 equiv; 147 mg) was attached to Rink resin. Then Fmoc-*D*-Ala-OH (0.38 mmol; 1 equiv; 118 mg) was attached to the resin-bound amino acid. The dipeptide underwent acylation with (*M*)-TBBA for 40 hours. After cleavage from the resin, the crude product was purified by CC (CH_2Cl_2/MeOH 20:1; column dimensions 4 × 10 cm), 75 mg (87% yield) of white solid was obtained. **^{1}H** NMR (400 MHz, DMSO-*d*$_6$) δ 8.45 (d, *J* = 7.4 Hz, 1H), 8.05 (d, *J* = 8.7 Hz, 1H), 7.87 – 7.84 (m, 1H), 7.75 – 7.72 (m, 3H), 7.67 – 7.63 (m, 1H), 7.40 – 7.32 (m, 2H), 7.29 (br. s, 1H), 7.20 – 7.13 (m, 6H), 7.04 (br. s, 1H), 4.38 (ddd, *J* = 10.2, 8.8, 4.2 Hz, 1H), 3.92 (p, *J* = 7.0 Hz, 1H), 3.03 (dd, *J* = 13.7, 4.2 Hz, 1H), 2.69 (dd, *J* = 13.7, 10.3 Hz, 1H), 0.61 (d, *J* = 7.1 Hz, 3H). **^{13}C{^{1}H} NMR** (101 MHz, DMSO-*d*$_6$) δ 172.8, 171.3, 164.8, 140.4 (q, *J* = 37.7 Hz), 140.1, 137.9, 136.7, 134.6, 131.6, 131.3, 130.2, 129.1, 128.9, 127.9, 126.1, 125.4, 123.7, 120.6, 118.7 (q, *J* = 271.9 Hz), 111.4, 53.5, 48.2, 37.4, 17.3. **^{19}F NMR** (376 MHz, DMSO-*d*$_6$) δ -59.49 (s). **^{19}F NMR** (76 MHz, THF) δ -60.72 (s). **HRMS** (ESI): *m/z* $[M + H]^+$ Calcd for $C_{27}H_{25}F_3N_5O_3$ 524.1904; Found 524.1906.

(P)-TBBA-(*L*)-Phe-(*L*)-Ala-NH_2 ***(P)*-24**

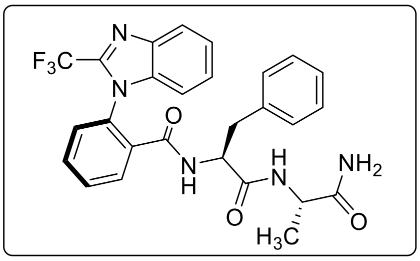

Following the general procedure, Fmoc-Ala-OH (0.38 mmol; 1 equiv; 118 mg) was attached to Rink resin. Then Fmoc-Phe-OH (0.38 mmol; 1 equiv; 147 mg) was attached to the resin-bound amino acid. The dipeptide underwent acylation with (*P*)-TBBA for 16 hours. After cleavage from the resin, the crude product was purified by CC (CH_2Cl_2/MeOH 20:1; column dimensions 4 × 10 cm), 94 mg (30% yield) of white solid was obtained. **^{1}H NMR** (400 MHz, DMSO-*d*$_6$) δ 8.69 (d, *J* = 8.3 Hz, 1H), 7.94 (d, *J* = 7.4 Hz, 1H), 7.83 (dt, *J* = 8.2, 0.9 Hz, 1H), 7.80 – 7.76 (m, 1H), 7.76 – 7.71 (m, 2H), 7.62 – 7.58 (m, 1H), 7.35 (ddd, *J* = 8.2, 7.2, 1.1 Hz, 1H), 7.25 (ddd, *J* = 8.2, 7.2, 1.1 Hz, 1H), 7.21 – 7.15 (m, 3H), 7.14 – 7.11 (m, 2H), 7.09 (br. s, 1H), 6.94 (br. s, 1H), 6.90 (dt, *J* = 8.2, 1.0 Hz, 1H), 4.32 (ddd, *J* = 9.6, 8.5, 4.9 Hz, 1H), 4.14 (p, *J* = 7.1 Hz, 1H), 2.90 (dd, *J* = 14.0, 4.8 Hz, 1H), 2.71 (dd, *J* = 14.0, 9.7 Hz, 1H), 1.15 (d, *J* = 7.1 Hz, 3H). **^{13}C{^{1}H} NMR** (101 MHz, DMSO-*d*$_6$) δ 173.9, 170.3, 164.7, 140.0 (q, *J* = 37.8 Hz), 140.0, 137.9, 137.3, 133.7, 132.0, 131.6, 130.2, 129.6, 129.3, 128.9, 128.0, 126.1, 125.6, 123.4, 120.5, 118.7 (q, *J* = 271.8 Hz), 111.3, 54.1, 48.1, 36.8, 18.2. **^{19}F NMR** (376 MHz, DMSO-*d*$_6$) δ -59.68 (s). **^{19}F NMR** (76 MHz, THF) δ -60.79 (s). **HRMS** (ESI): *m/z* [M + H]$^+$ Calcd for $C_{27}H_{25}F_3N_5O_3$ 524.1904; Found 524.1902.

(M)-TBBA-(*L*)-Phe-(*L*)-Ala-NH_2 ***(M)*-24**

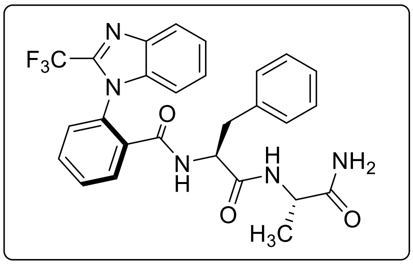

Following the general procedure, Fmoc-Ala-OH (0.38 mmol; 1 equiv; 118 mg) was attached to Rink resin. Then Fmoc-Phe-OH (0.38 mmol; 1 equiv; 147 mg) was attached to the resin-bound amino acid. The dipeptide underwent acylation with (*M*)-TBBA for 40 hours. After cleavage from the resin, the crude product was purified CC (CH_2Cl_2/MeOH 20:1; column dimensions 4 × 10 cm), 91 mg (35% yield) of white solid was obtained. **^{1}H NMR** (400 MHz, DMSO-*d*$_6$) δ 8.74 (d, *J* = 8.6 Hz, 1H), 7.85 – 7.77 (m, 2H), 7.75 – 7.66 (m, 3H), 7.61 – 7.56 (m, 1H), 7.37 – 7.29 (m, 2H), 7.28 – 7.23 (m, 4H), 7.20 – 7.15 (m, 1H), 7.11 (br. s, 1H), 7.00 – 6.93 (m, 2H), 4.37 (ddd, *J* = 10.4, 8.7, 4.4 Hz, 1H), 4.01 (p, *J* = 7.1 Hz, 1H), 2.98 (dd, *J* = 13.8, 4.4 Hz, 1H), 2.83 (dd, *J* = 13.8, 10.4 Hz, 1H), 1.00 (d, *J* = 7.1 Hz, 3H). **^{13}C{^{1}H} NMR** (101 MHz, DMSO-*d*$_6$) δ 174.0, 170.0, 164.5, 140.1 (q, *J* = 37.6 Hz), 140.0, 137.9, 136.9, 133.7, 132.0, 131.6, 130.2, 129.5, 129.1, 129.1, 128.0, 126.2, 125.6, 123.4, 120.5, 118.6 (q, *J* = 272.0 Hz), 111.3, 54.0, 47.9, 37.1, 18.3. **^{19}F NMR** (376 MHz, DMSO-*d*$_6$) δ -59.64 (s). **^{19}F NMR** (76 MHz, THF) δ -60.63 (s). **HRMS** (ESI): *m/z* [M + H]$^+$ Calcd for $C_{27}H_{25}F_3N_5O_3$ 524.1904; Found 524.1904.

(P)-TBBA-(*L*)-Ser-(*L*)-Ala-NH_2 ***(P)*-25**

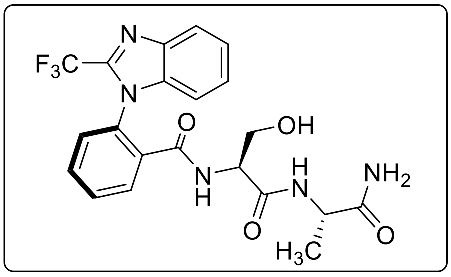

Following the general procedure, Fmoc-Ala-OH (0.38 mmol; 1 equiv; 118 mg) was attached to Rink resin. Then Fmoc-Ser(*t*Bu)-OH (0.38 mmol; 1 equiv; 146 mg) was attached to the resin-bound amino acid. The dipeptide underwent acylation with (*P*)-TBBA for 16 hours. After cleavage from the resin, the crude product was purified by CC (CH_2Cl_2/MeOH 15:1; column dimensions 4 × 10 cm), 143 mg (53% yield) of white solid was obtained. **^{1}H NMR** (400 MHz, DMSO-*d*$_6$) δ 8.43 (d, *J* = 8.0 Hz, 1H), 8.05 (d, *J* = 7.5 Hz, 1H), 7.89 – 7.80 (m, 2H), 7.79 – 7.72 (m, 2H), 7.68 – 7.62 (m, 1H), 7.42 – 7.34 (m, 2H), 7.21 (br. s, 1H), 7.17 – 7.12 (m, 1H), 7.04 (br. s, 1H), 4.95 (s, 1H), 4.16 (p, *J* = 7.2 Hz, 1H), 4.10 – 4.02 (m, 1H), 3.26 – 3.11 (m, 2H), 1.19 (d, *J* = 7.2 Hz, 3H). **^{13}C{^{1}H} NMR** (101 MHz, DMSO-*d*$_6$) δ 174.2, 169.3, 165.0, 140.3 (q, *J* = 37.7 Hz), 140.1, 136.9, 134.3, 131.8, 131.5, 130.3, 129.3, 129.1, 125.5, 123.7, 120.6, 118.7 (q, *J* = 272.0 Hz), 111.5, 61.2, 54.5, 48.2, 17.8. **^{19}F NMR** (376 MHz, DMSO-*d*$_6$) δ -59.55 (s). **^{19}F NMR** (76 MHz, THF) δ -60.71 (s). **HRMS** (ESI): *m/z* $[M + H]^+$ Calcd for $C_{21}H_{21}F_3N_5O_4$ 464.1544; Found 464.1540.

(M)-TBBA-(*L*)-Ser-(*L*)-Ala-NH_2 ***(M)*-25**

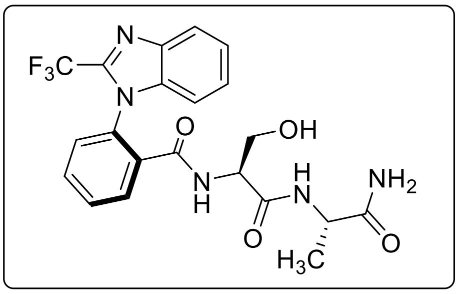

Following the general procedure, Fmoc-Ala-OH (0.38 mmol; 1 equiv; 118 mg) was attached to Rink resin. Then Fmoc-Ser(*t*Bu)-OH (0.38 mmol; 1 equiv; 146 mg) was attached to the resin-bound amino acid. The dipeptide underwent acylation with (*M*)-TBBA for 40 hours. After cleavage from the resin, the crude product was purified by CC (CH_2Cl_2/MeOH 15:1; column dimensions 4 × 10 cm), 117 mg (42% yield) of white solid was obtained. **^{1}H NMR** (400 MHz, DMSO-*d*$_6$) δ 8.44 (d, *J* = 8.0 Hz, 1H), 7.96 – 7.91 (m, 1H), 7.89 (d, *J* = 7.5 Hz, 1H), 7.87 – 7.82 (m, 1H), 7.80 – 7.73 (m, 2H), 7.67 – 7.61 (m, 1H), 7.40 – 7.32 (m, 2H), 7.18 (br. s, 1H), 7.08 – 6.97 (m, 2H), 5.05 (t, *J* = 5.7 Hz, 1H), 4.14 (dt, *J* = 7.9, 6.3 Hz, 1H), 4.06 (p, *J* = 7.2 Hz, 1H), 3.59 – 3.41 (m, *J* = 5.8 Hz, 2H), 1.09 (d, *J* = 7.2 Hz, 3H). **^{13}C{^{1}H} NMR** (101 MHz, DMSO-*d*$_6$) δ 174.1, 169.1, 164.8, 140.0, 139.9 (q, *J* = 37.9 Hz), 137.2, 133.7, 132.0, 131.7, 130.3, 129.6, 129.5, 125.7, 123.5, 120.5, 118.8 (q, *J* = 271.8 Hz), 111.5, 61.6, 54.9, 48.1, 17.9. **^{19}F NMR** (376 MHz, DMSO-*d*$_6$) δ -59.59 (s). **^{19}F NMR** (76 MHz, THF) δ -60.65 (s). **HRMS** (ESI): *m/z* $[M + H]^+$ Calcd for $C_{21}H_{21}F_3N_5O_4$ 464.1544; Found 464.1543.

(P)-TBBA-(*L*)-Ala-(*L*)-Ala-(*L*)-Ala-NH2 ***(P)*-26**

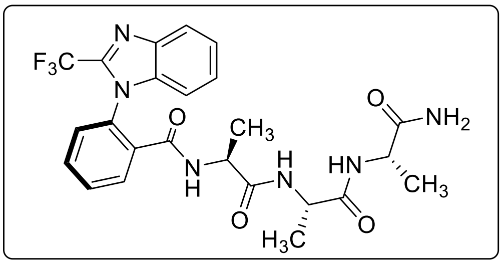

Following the general procedure, Fmoc-Ala-OH (0.38 mmol; 1 equiv; 118 mg) was attached to Rink resin (1 g). Then the second Fmoc-Ala-OH (0.38 mmol; 1 equiv; 118 mg) and third Fmoc-Ala-OH (0.38 mmol; 1 equiv; 118 mg) were attached to the resin-bound amino acid(s). The tripeptide underwent acylation with (*P*)-TBBA for 16 hours. After cleavage from the resin, the crude product was purified CC (CH_2Cl_2/MeOH 15:1; column dimensions 4 × 10 cm), 160 mg (72% yield) of white solid was obtained. **^{1}H NMR** (400 MHz, DMSO-d_6) δ 8.57 (d, *J* = 7.6 Hz, 1H), 7.98 (d, *J* = 7.2 Hz, 1H), 7.88 – 7.84 (m, 1H), 7.83 – 7.79 (m, 1H), 7.78 – 7.73 (m, 2H), 7.73 – 7.69 (m, 1H), 7.68 – 7.63 (m, 1H), 7.42 – 7.35 (m, 2H), 7.23 (br. s, 1H), 7.17 – 7.11 (m, 1H), 6.97 (br. s, 1H), 4.19 (p, *J* = 7.1 Hz, 1H), 4.14 (p, *J* = 7.1 Hz, 1H), 3.99 (p, *J* = 7.1 Hz, 1H), 1.17 (d, *J* = 7.1 Hz, 3H), 1.16 (d, *J* = 7.1 Hz, 3H), 0.88 (d, *J* = 7.1 Hz, 3H). **^{13}C{^{1}H} NMR** (101 MHz, DMSO-d_6) δ 174.5, 172.0, 172.0, 165.4, 140.9 (q, *J* = 37.6 Hz), 140.7, 137.3, 135.2, 132.2, 131.9, 130.8, 129.7, 129.6, 126.0, 124.2, 121.2, 119.3 (q, *J* = 270.7, 269.6 Hz), 112.0, 48.8, 48.6, 48.4, 18.9, 18.3, 17.8. **^{19}F NMR** (376 MHz, DMSO-d_6) δ -59.53 (s). **^{19}F NMR** (76 MHz, THF) δ -60.75 (s). **HRMS** (ESI): *m/z* $[M + H]^+$ Calcd for $C_{24}H_{26}F_3N_6O_4$ 519.1962; Found 519.1970.

(M)-TBBA-(*L*)-Ala-(*L*)-Ala-(*L*)-Ala-NH2 ***(M)*-26**

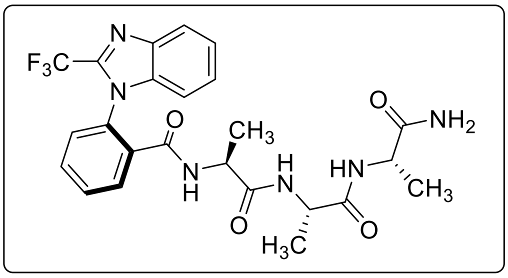

Following the general procedure, Fmoc-Ala-OH (0.38 mmol; 1 equiv; 118 mg) was attached to Rink resin (1 g). Then the second Fmoc-Ala-OH (0.38 mmol; 1 equiv; 118 mg) and third Fmoc-Ala-OH (0.38 mmol; 1 equiv; 118 mg) were attached to the resin-bound amino acid(s). The tripeptide underwent acylation with (*M*)-TBBA for 16 hours. After cleavage from the resin, the crude product was purified CC (CH_2Cl_2/MeOH 15:1; column dimensions 4 × 10 cm), 139 mg (63% yield) of white solid was obtained. **^{1}H NMR** (400 MHz, DMSO-d_6) δ 8.59 (d, *J* = 7.4 Hz, 1H), 7.92 – 7.82 (m, 3H), 7.79 – 7.73 (m, 2H), 7.68 (d, *J* = 7.5 Hz, 1H), 7.66 – 7.62 (m, 1H), 7.41 – 7.33 (m, 2H), 7.21 (br. s, 1H), 7.06 – 7.00 (m, 1H), 6.96 (br. s, 1H), 4.16 – 4.04 (m, 3H), 1.13 (d, *J* = 7.1 Hz, 3H), 1.12 (d, *J* = 7.0 Hz, 3H), 1.07 (d, *J* = 7.1 Hz, 3H). **^{13}C{^{1}H} NMR** (101 MHz, DMSO-d_6) δ 174.0, 171.5, 171.4, 164.6, 140.0, 139.7 (q, *J* = 38.0 Hz), 137.4, 133.9, 131.9, 131.6, 130.3, 129.5, 129.4, 125.6, 123.5, 120.5, 118.8 (q, *J* = 271.9 Hz), 111.6, 48.3, 48.2, 47.8, 18.3, 17.7, 17.5. **^{19}F NMR** (376 MHz, DMSO-d_6) δ -59.67 (s). **^{19}F NMR** (76 MHz, THF) δ -60.71 (s). **HRMS** (ESI): *m/z* $[M + H]^+$ Calcd for $C_{24}H_{26}F_3N_6O_4$ 519.1962; Found 519.1973.

Copies of NMR Spectra

rac-TBBA-(*D*)-Ala-NH_2 **rac-9**

^{1}H NMR (400 MHz, DMSO-*d*$_6$)

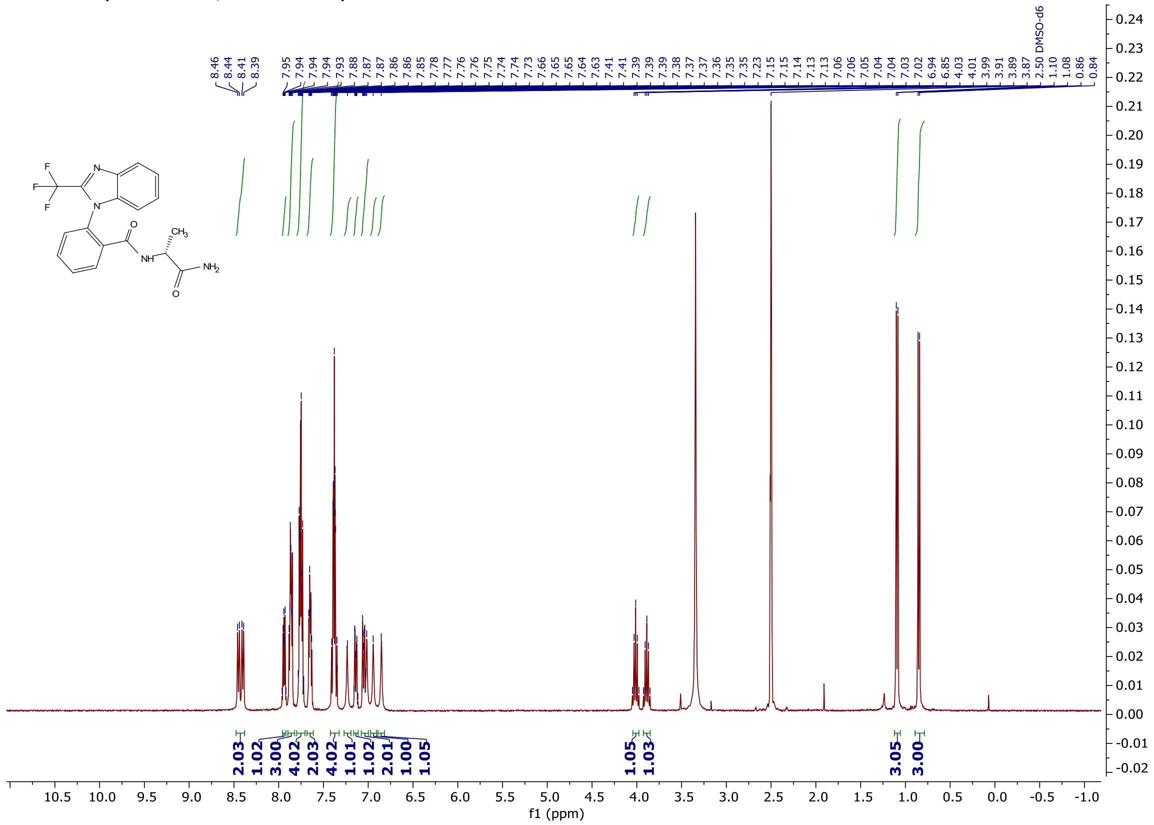

^{19}F NMR (376 MHz, DMSO-*d*$_6$)

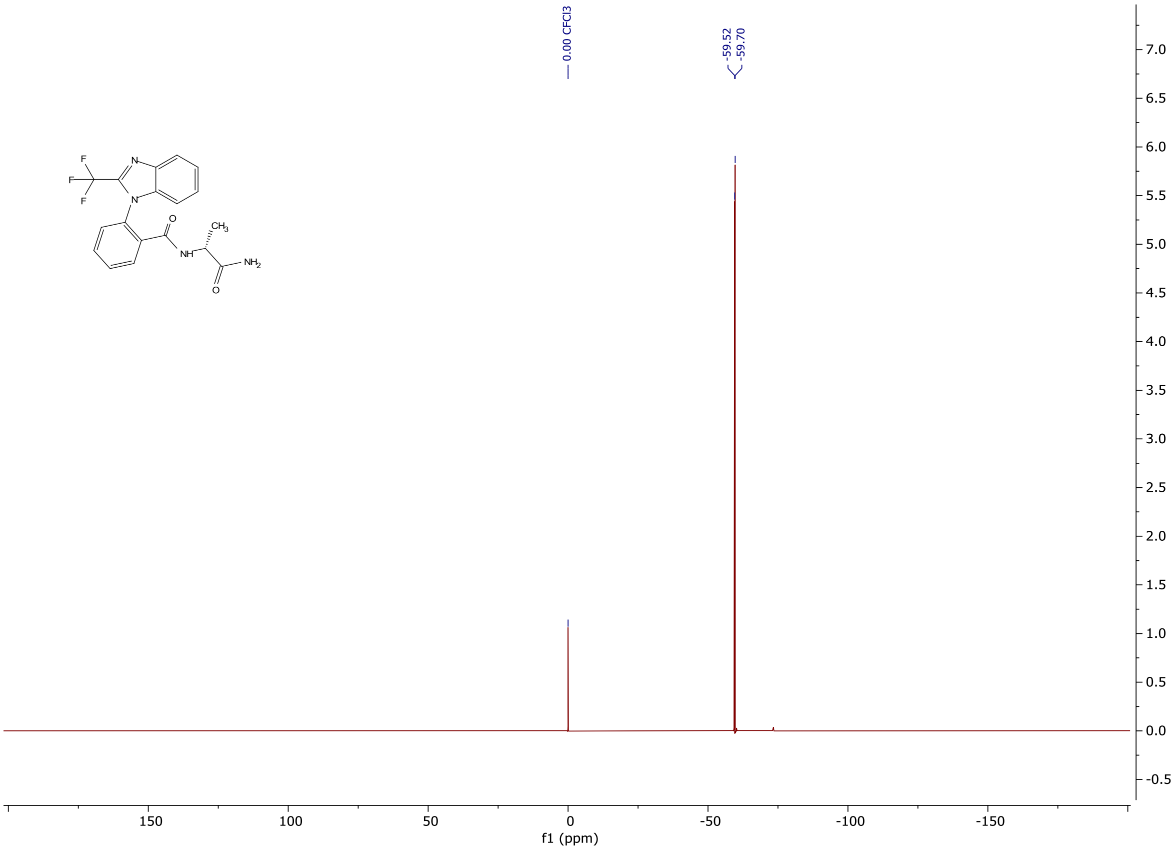

(*P*)-TBBA-(*L*)-Ala-NH_2 **(*P*)-9**

^{1}**H NMR** (400 MHz, DMSO-d_6)

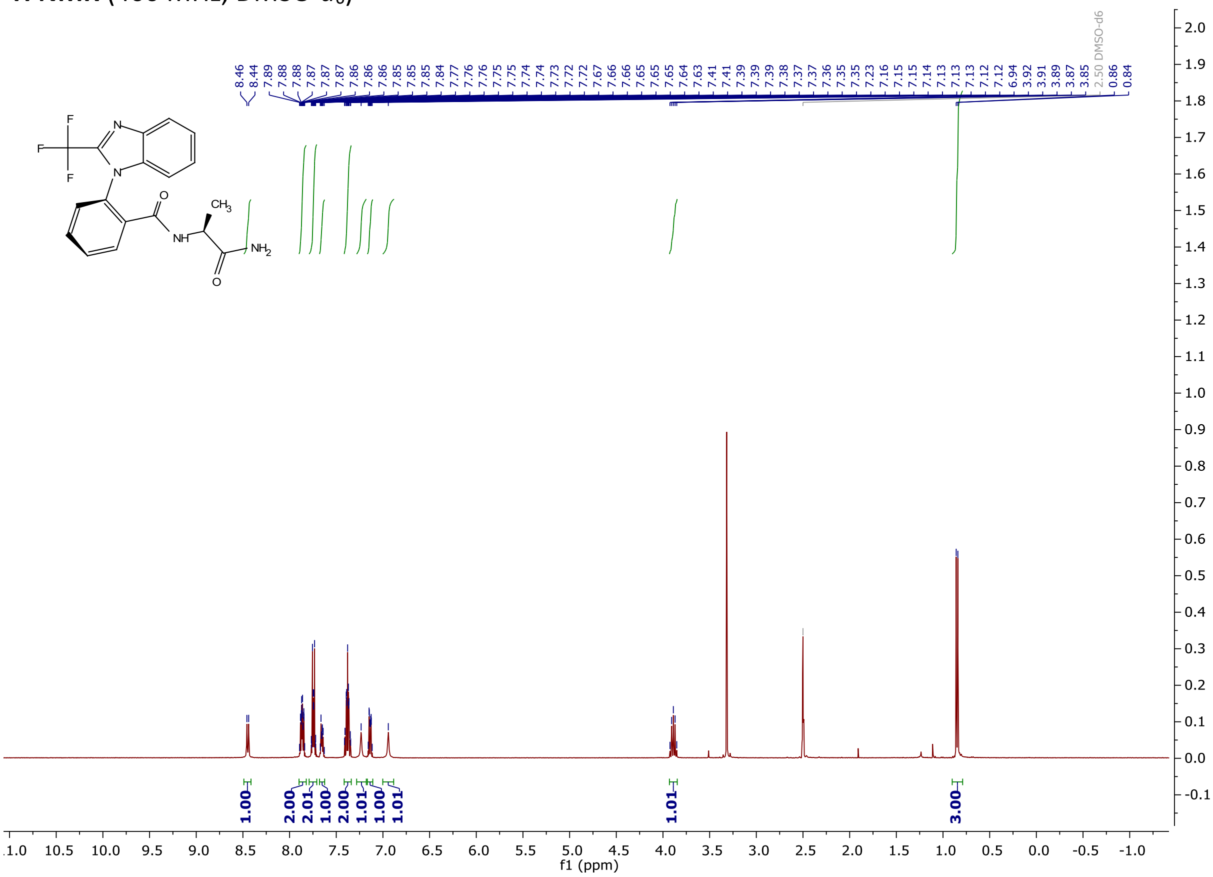

^{13}**C{^{1}H} NMR** (101 MHz, DMSO-d_6)

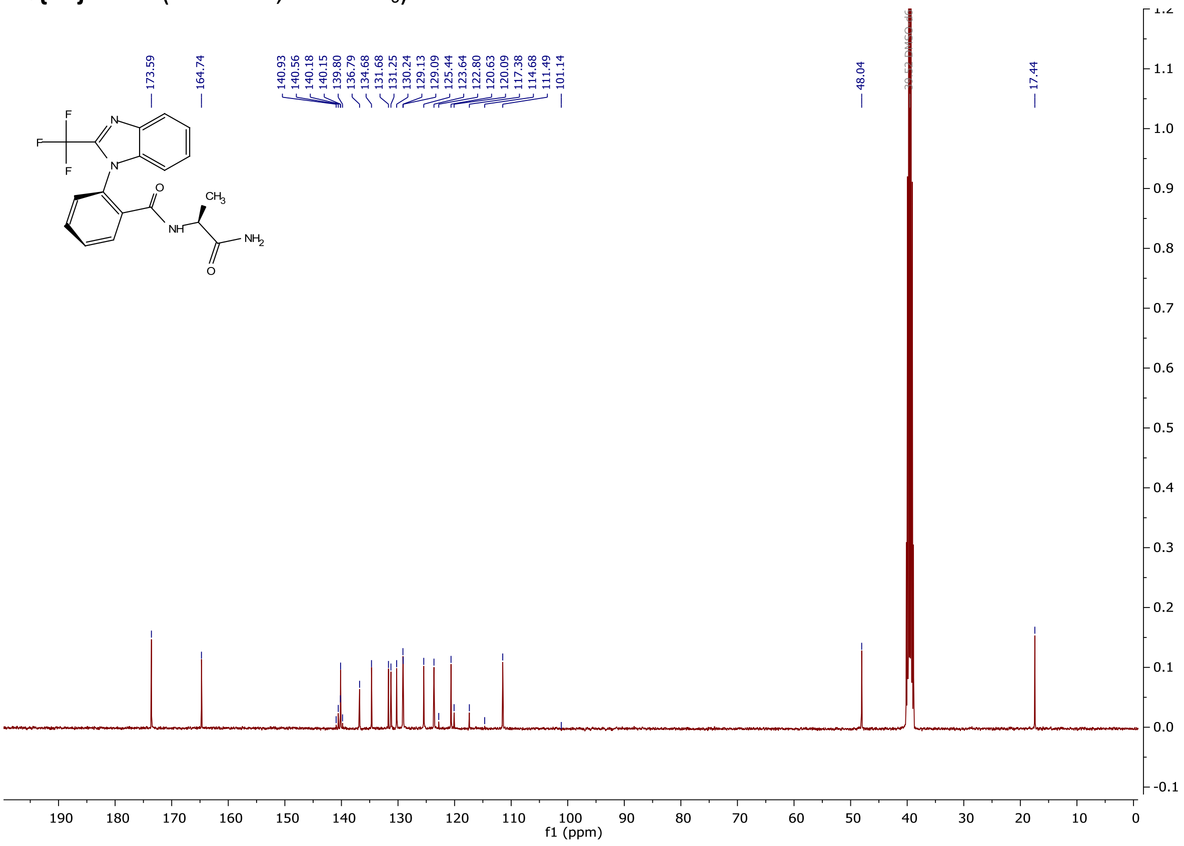

^{19}**F NMR** (376 MHz, DMSO-d_6)

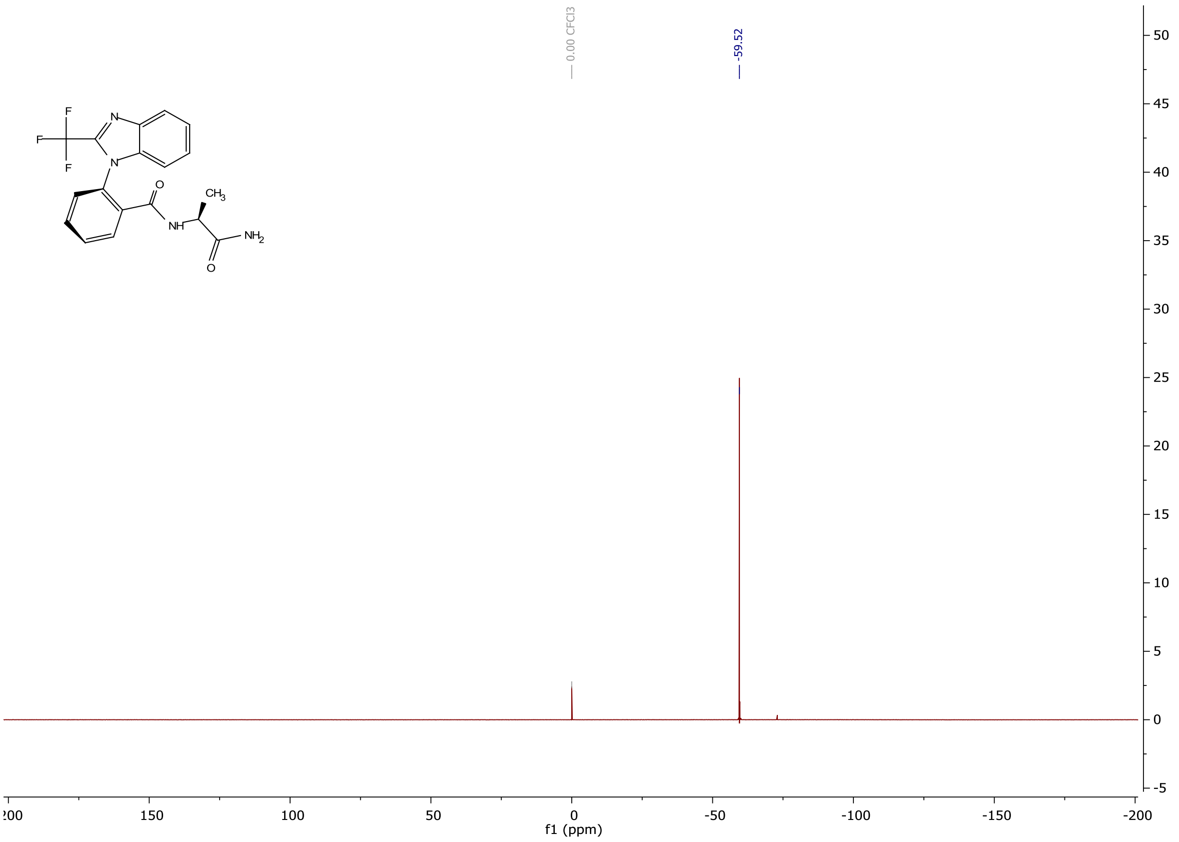

^{19}**F NMR** (76 MHz, THF)

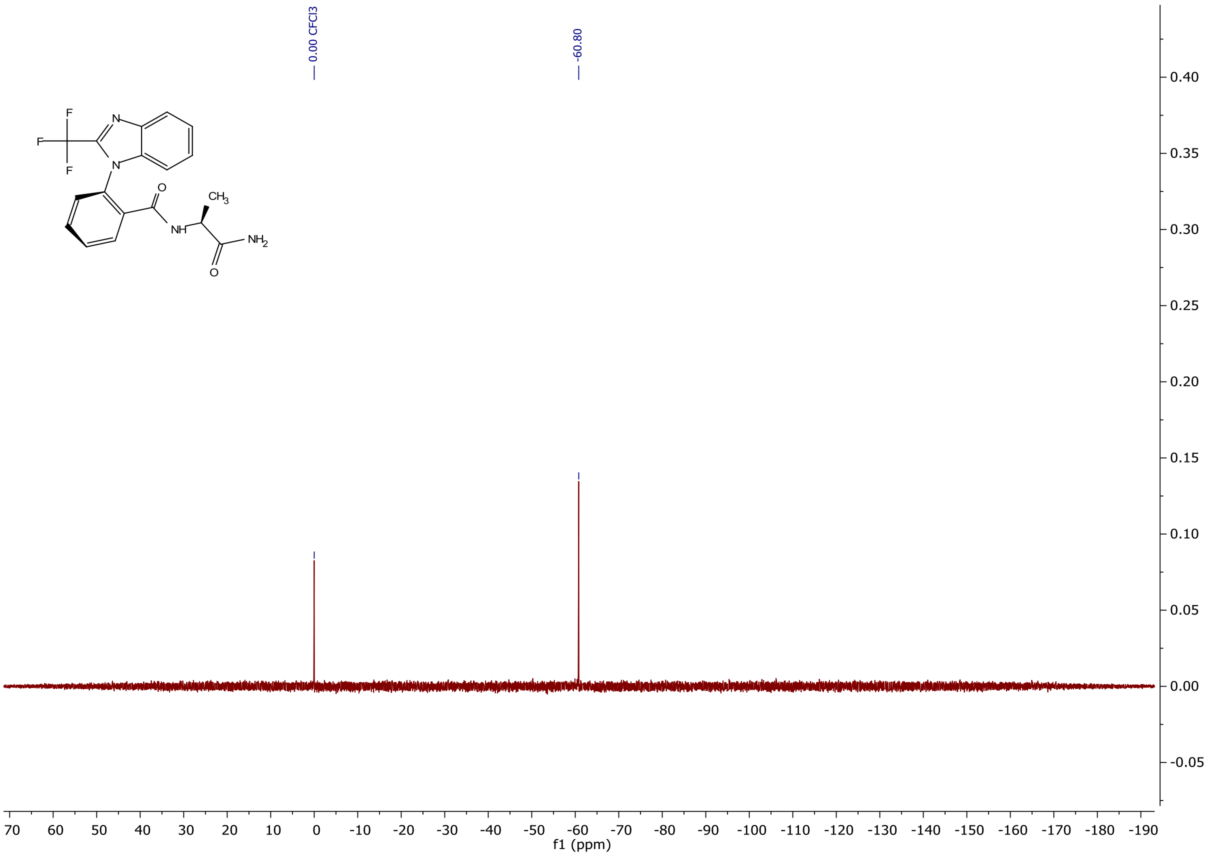

(*M*)-TBBA-(*L*)-Ala-NH_2 **(*M*)-9**

^{1}H NMR (400 MHz, DMSO-d_6)

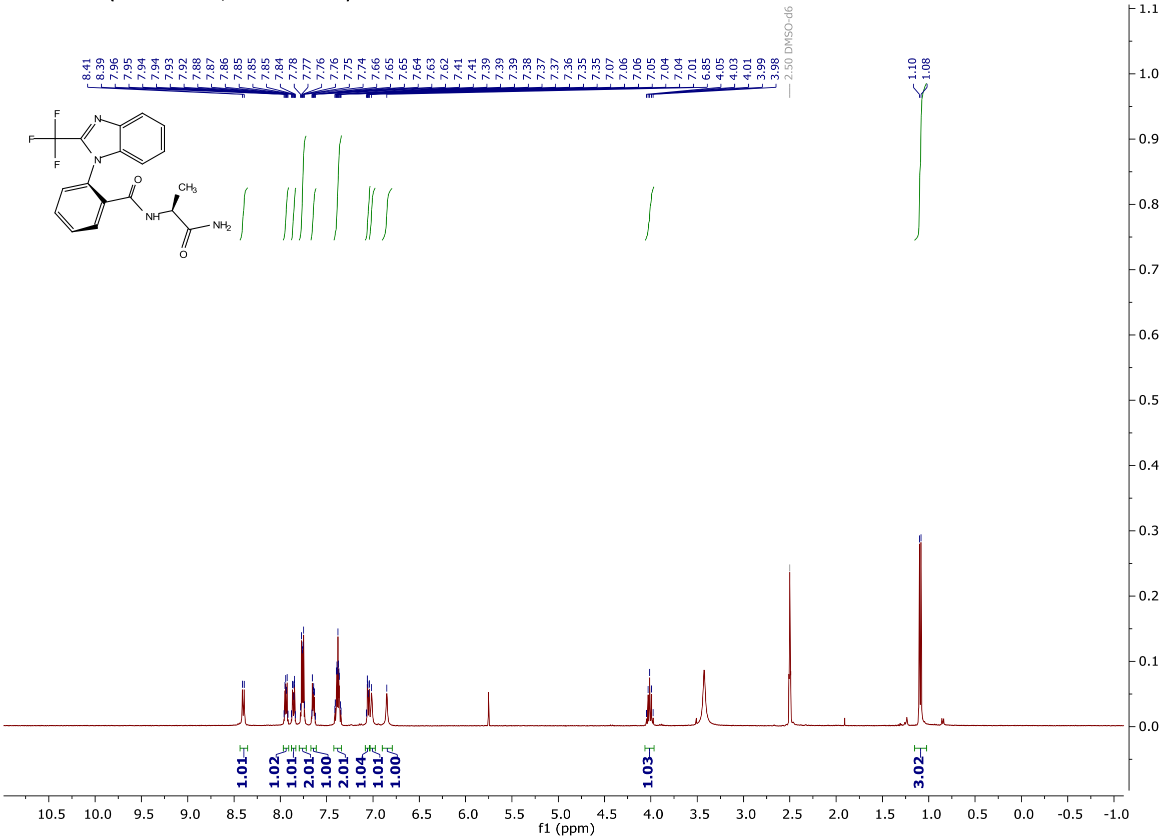

^{13}C{^{1}H} NMR (101 MHz, DMSO-d_6)

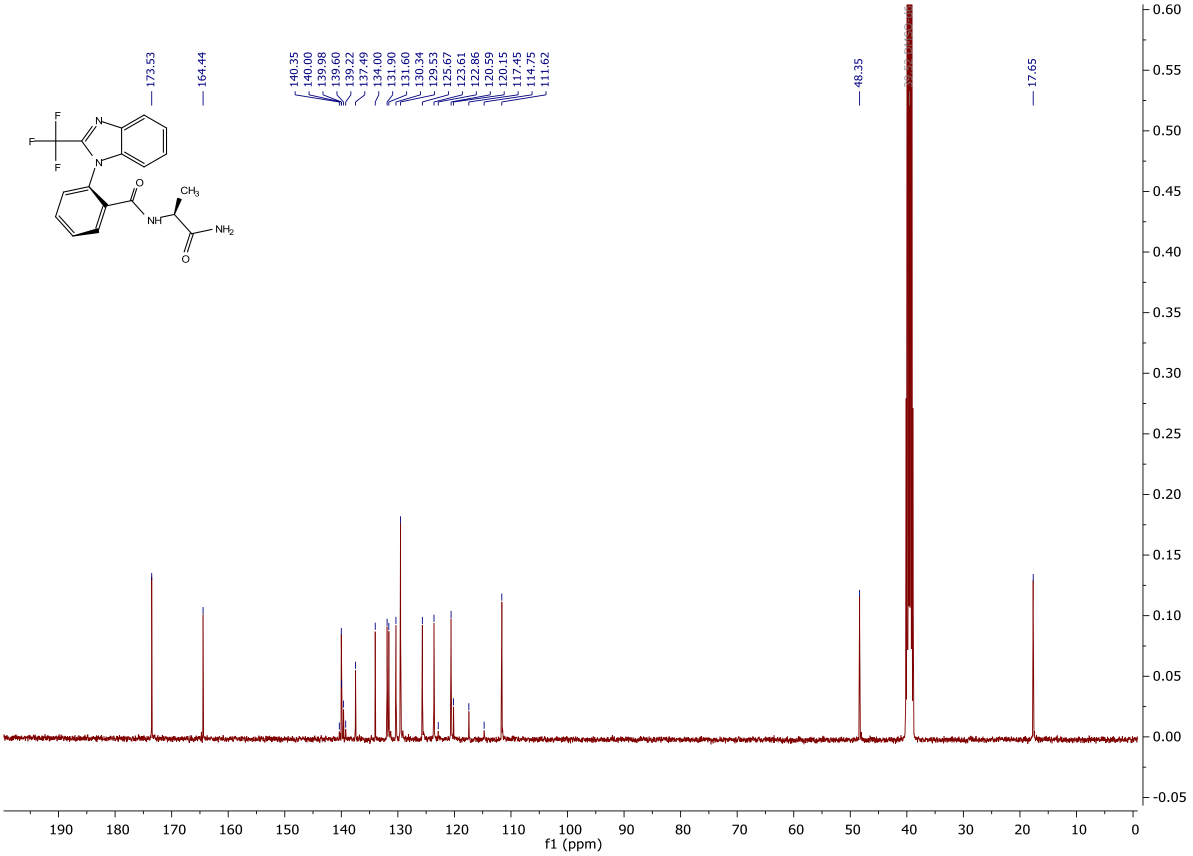

^{19}F NMR (376 MHz, DMSO-d_6)

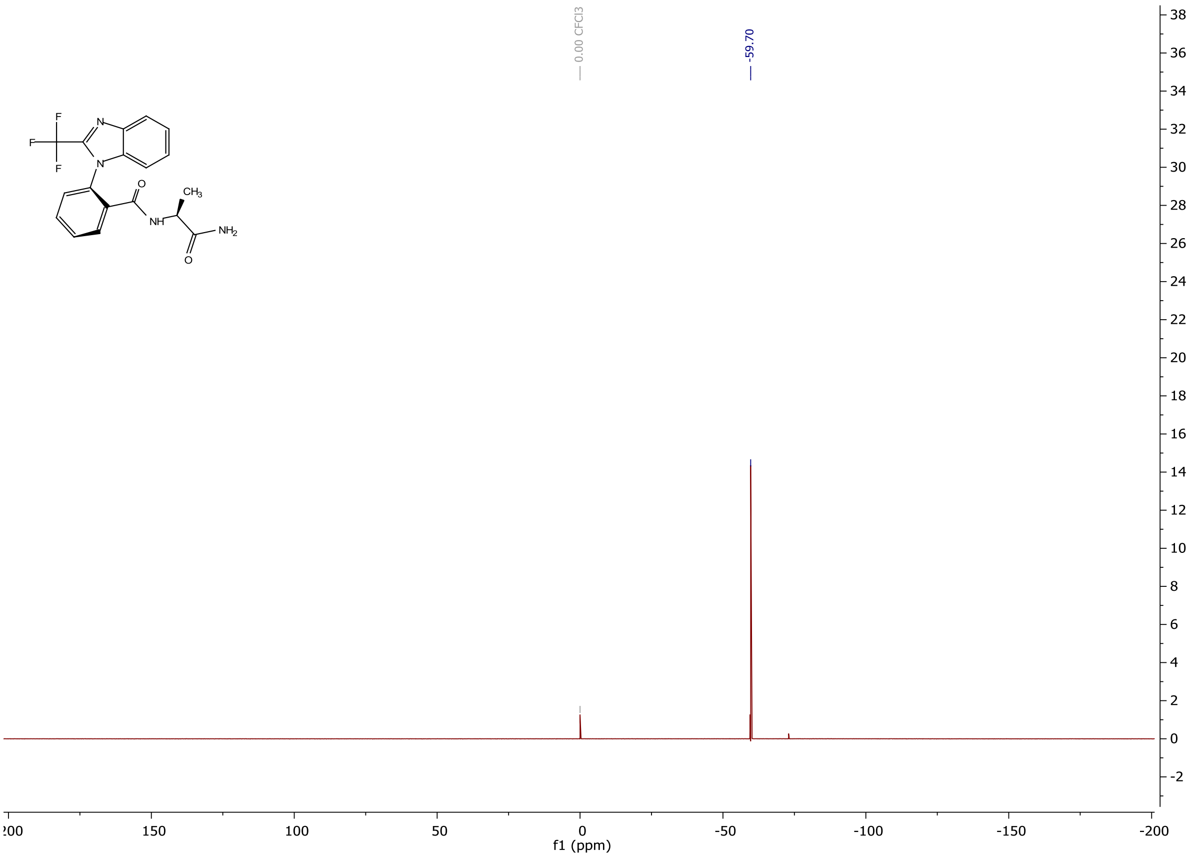

^{19}F NMR (76 MHz, THF)

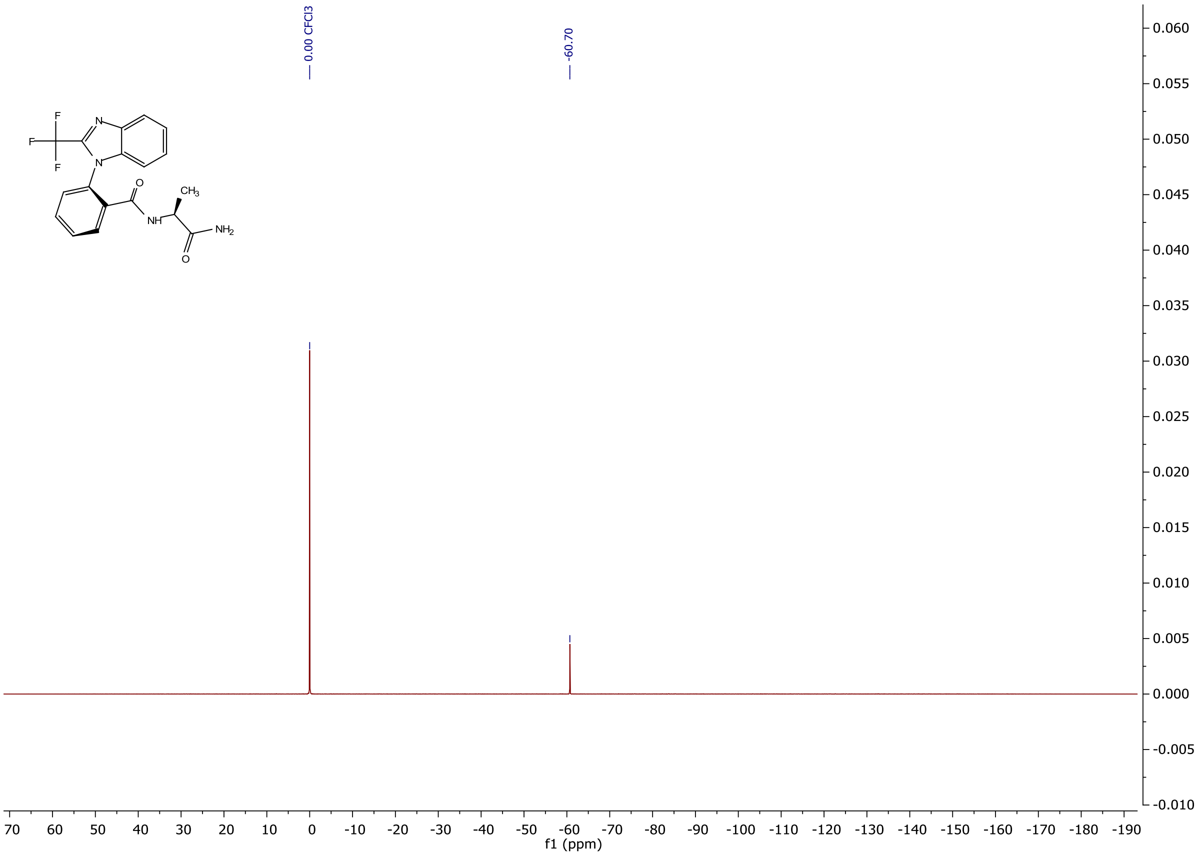

(*P*)-TBBA-(*L*)-Val-NH_2 **(*P*)-10**

^{1}H NMR (400 MHz, DMSO-d_6)

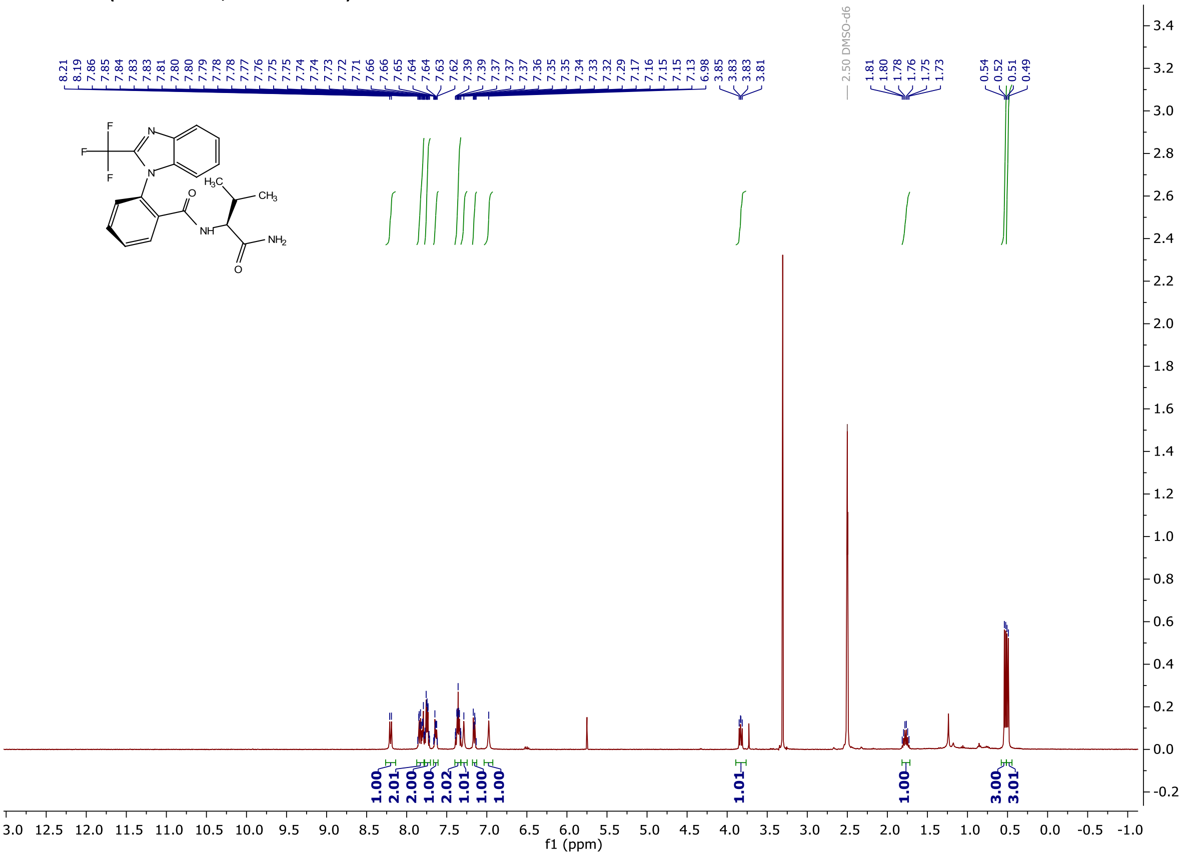

^{13}C{^{1}H} NMR (101 MHz, DMSO-d_6)

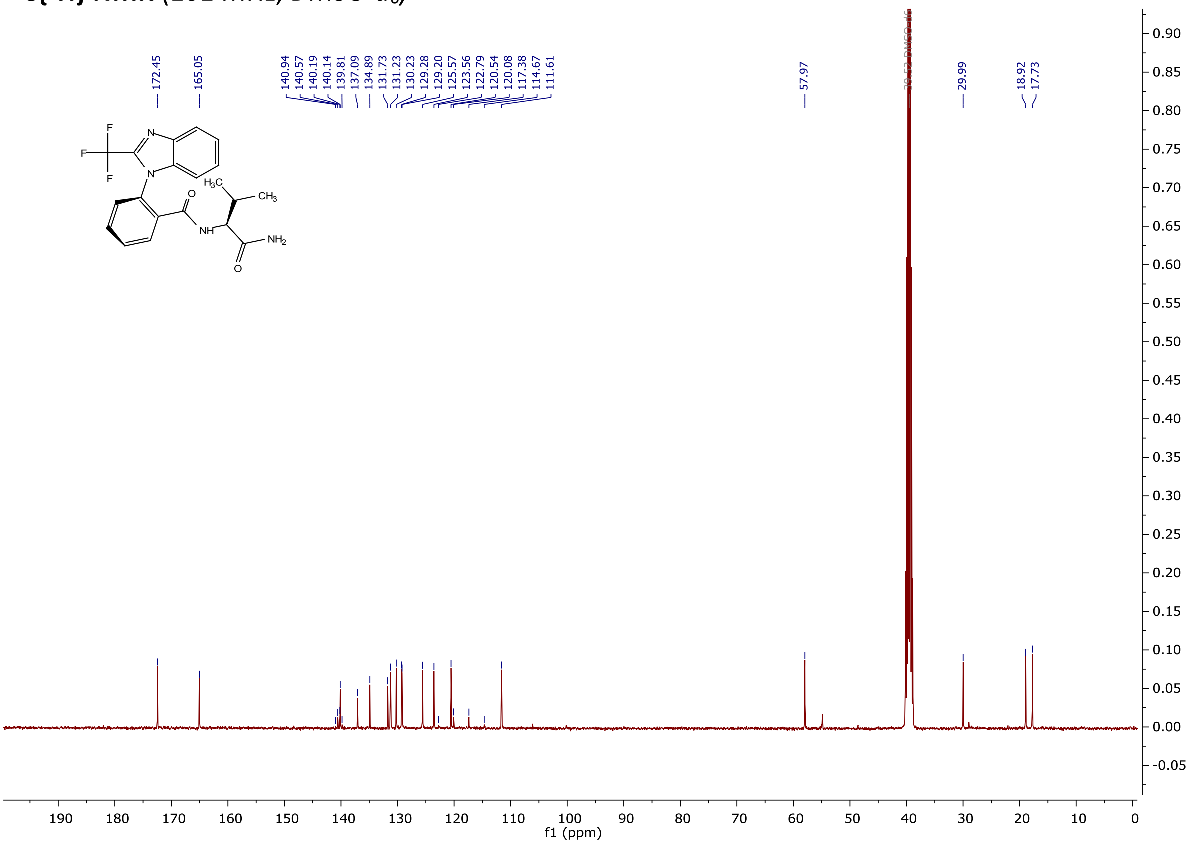

^{19}**F NMR** (376 MHz, DMSO-d_6)

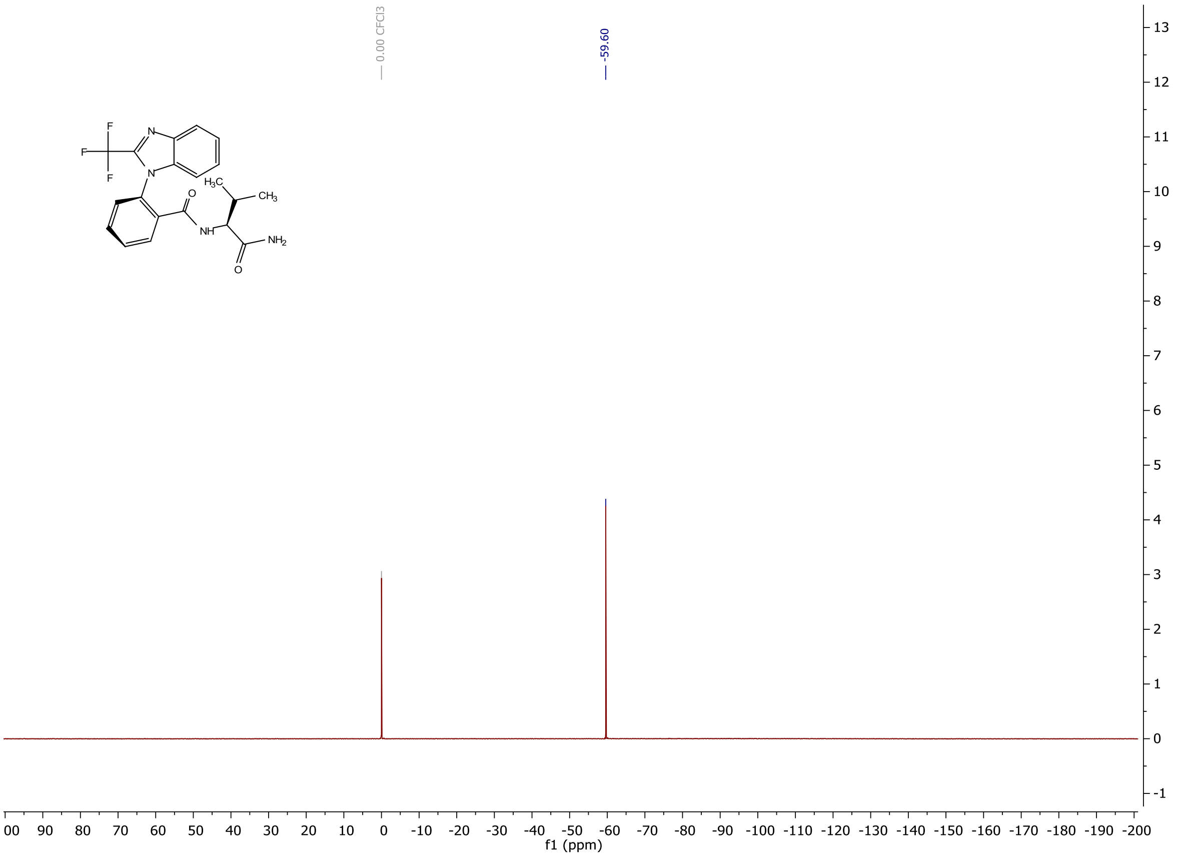

^{19}**F NMR** (76 MHz, THF)

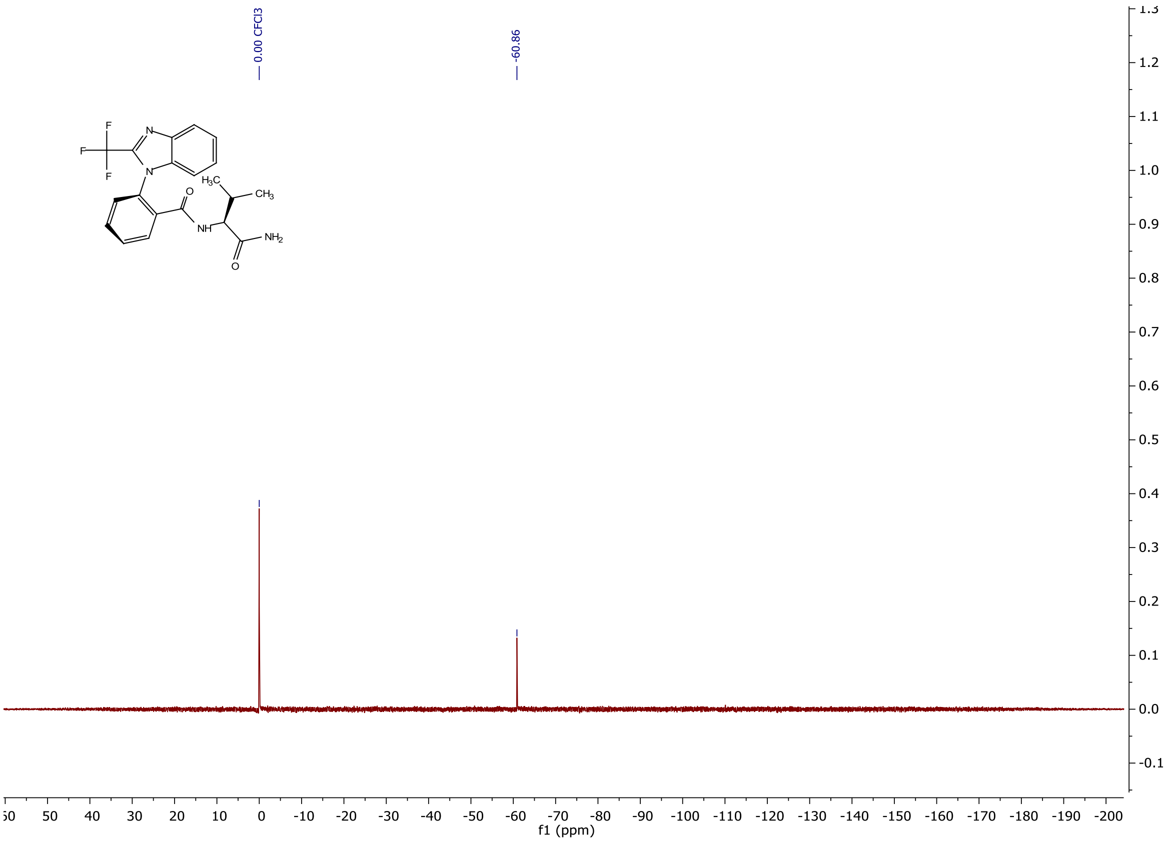

(*M*)-TBBA-(*L*)-Val-NH_2 **(*M*)-10**

^{1}H NMR (400 MHz, DMSO-d_6)

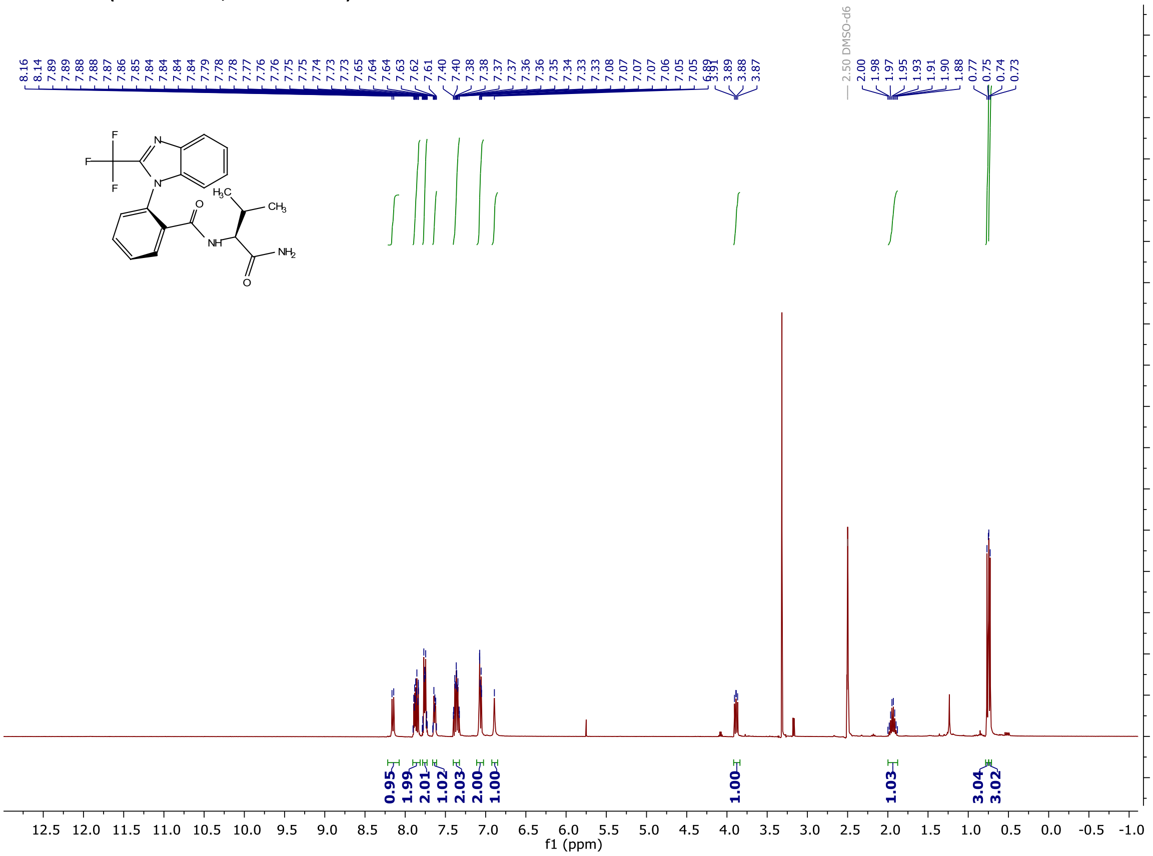

^{13}C{^{1}H} NMR (101 MHz, DMSO-d_6)

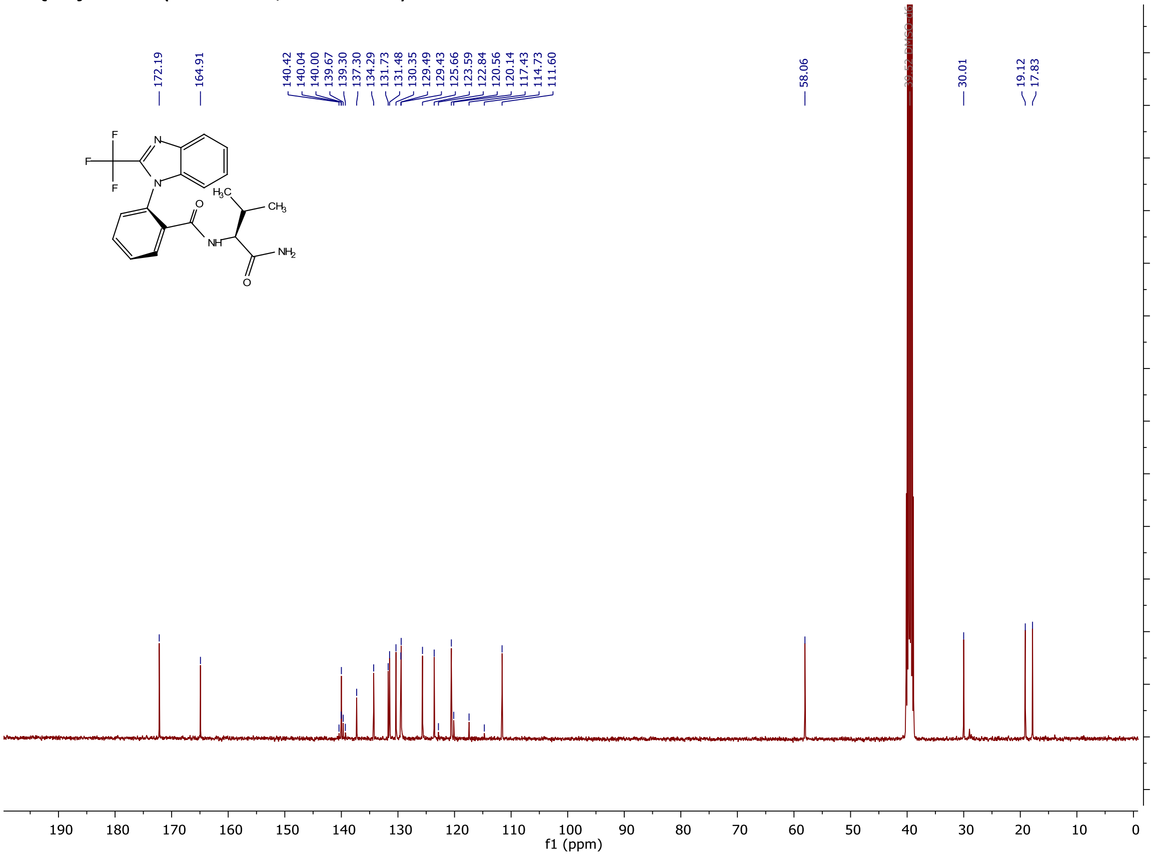

^{19}F NMR (376 MHz, DMSO-d_6)

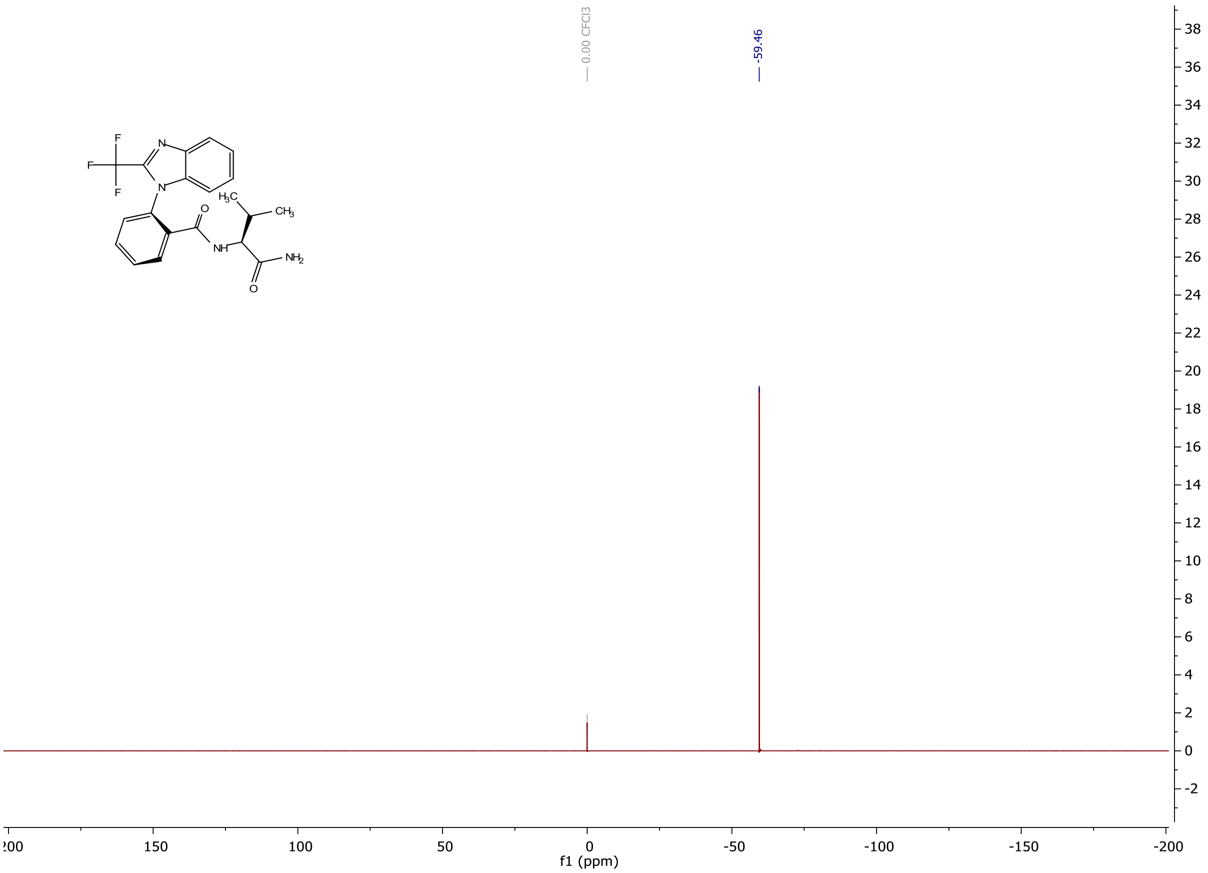

^{19}F NMR (76 MHz, THF)

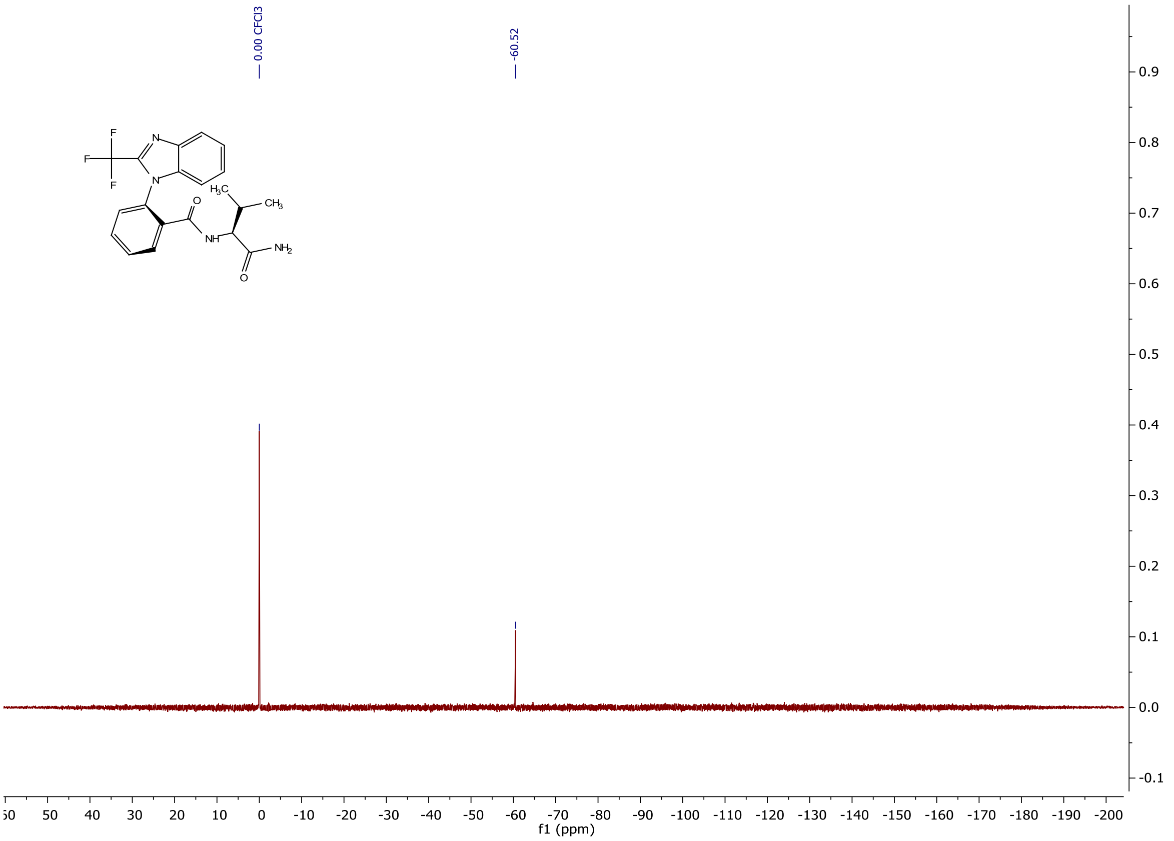

(*P*)-TBBA-(*L*)-Leu-NH_2 **(*P*)-11**

^{1}H NMR (400 MHz, DMSO-d_6)

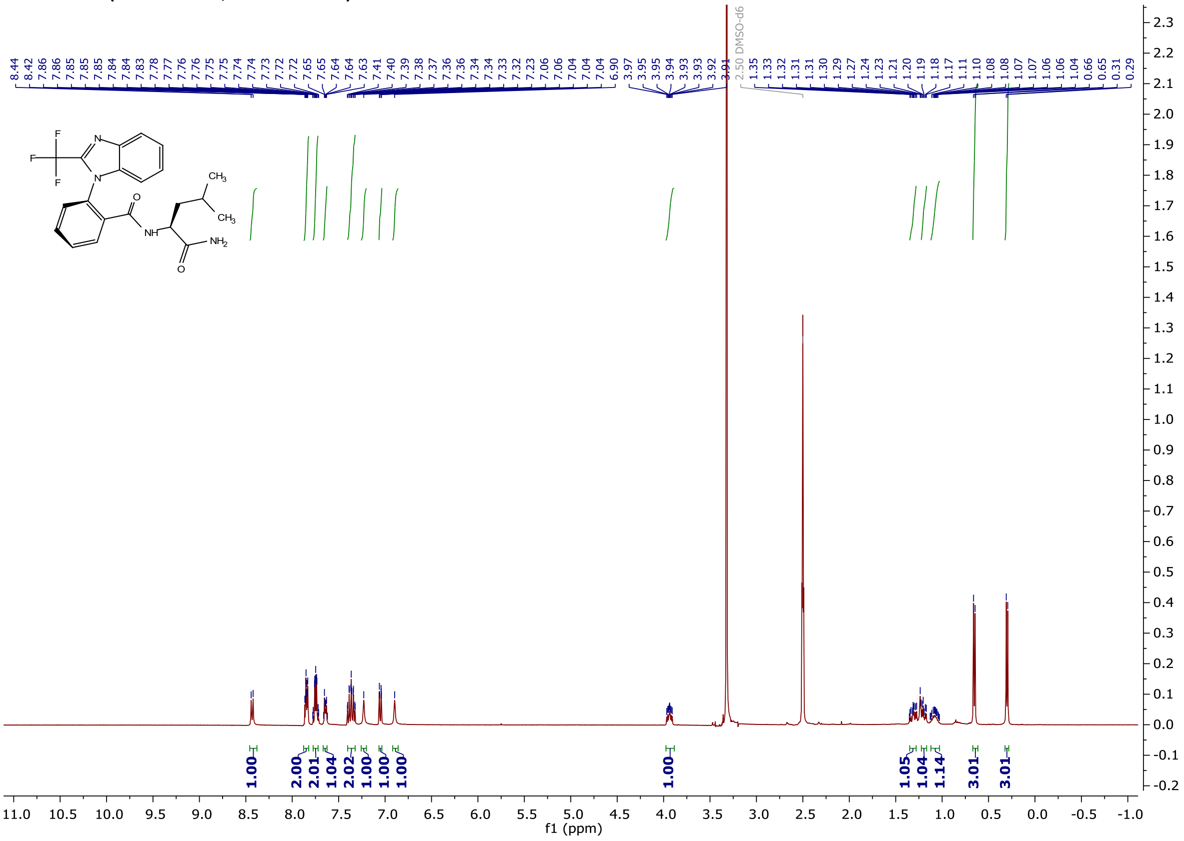

^{13}C{^{1}H} NMR (101 MHz, DMSO-d_6)

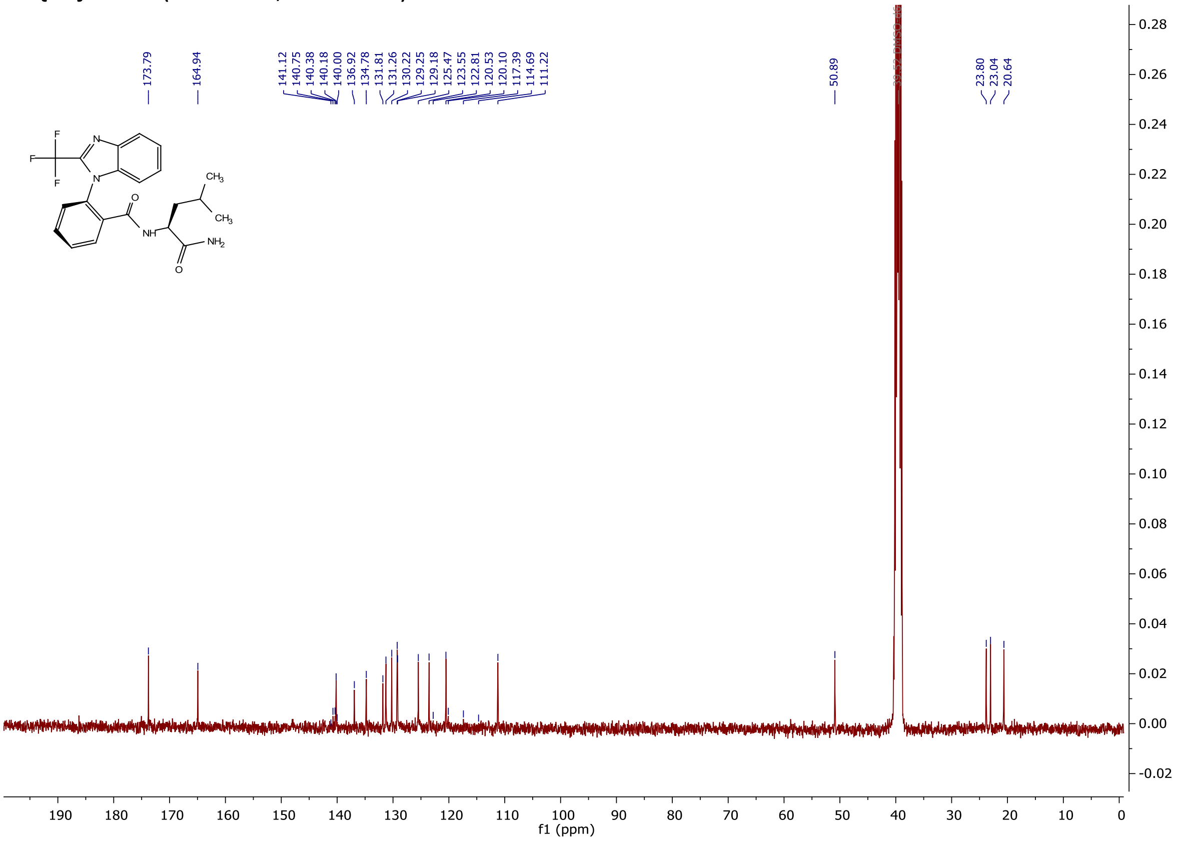

^{19}**F NMR** (376 MHz, DMSO-d_6)

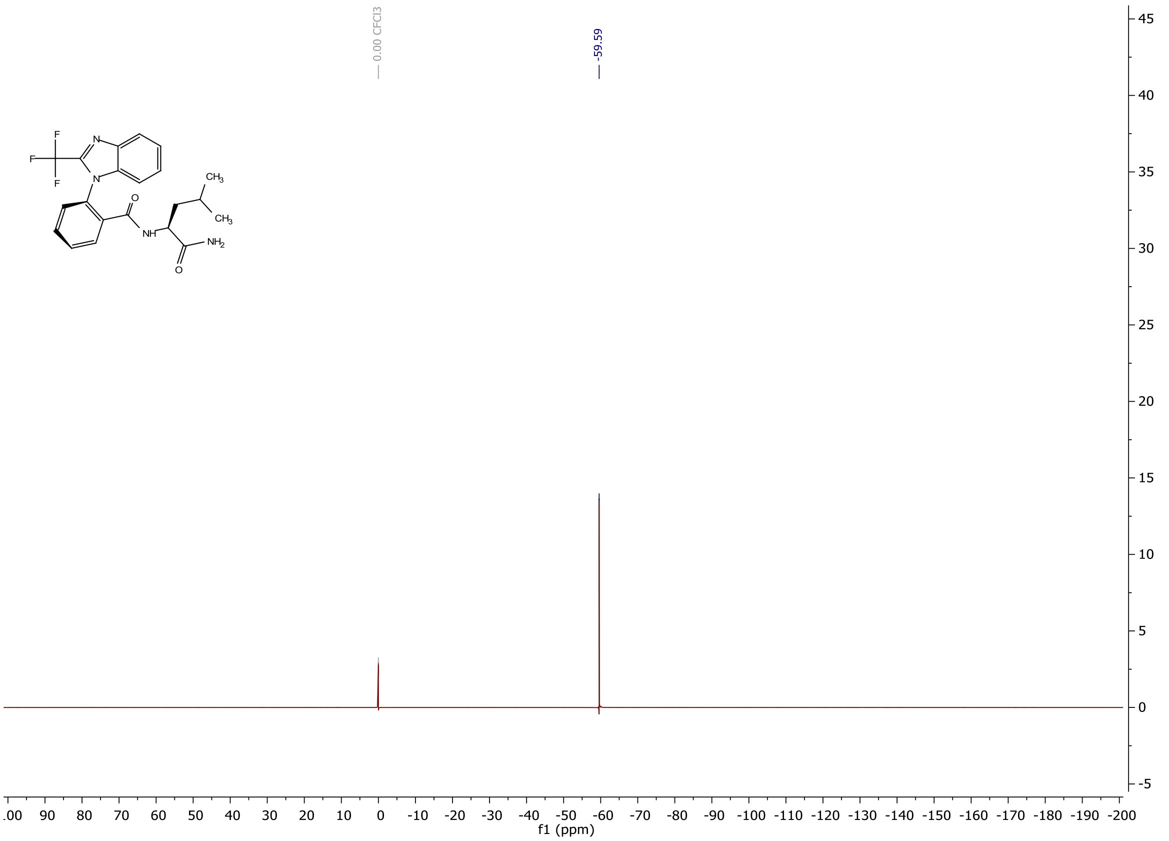

^{19}**F NMR** (76 MHz, THF)

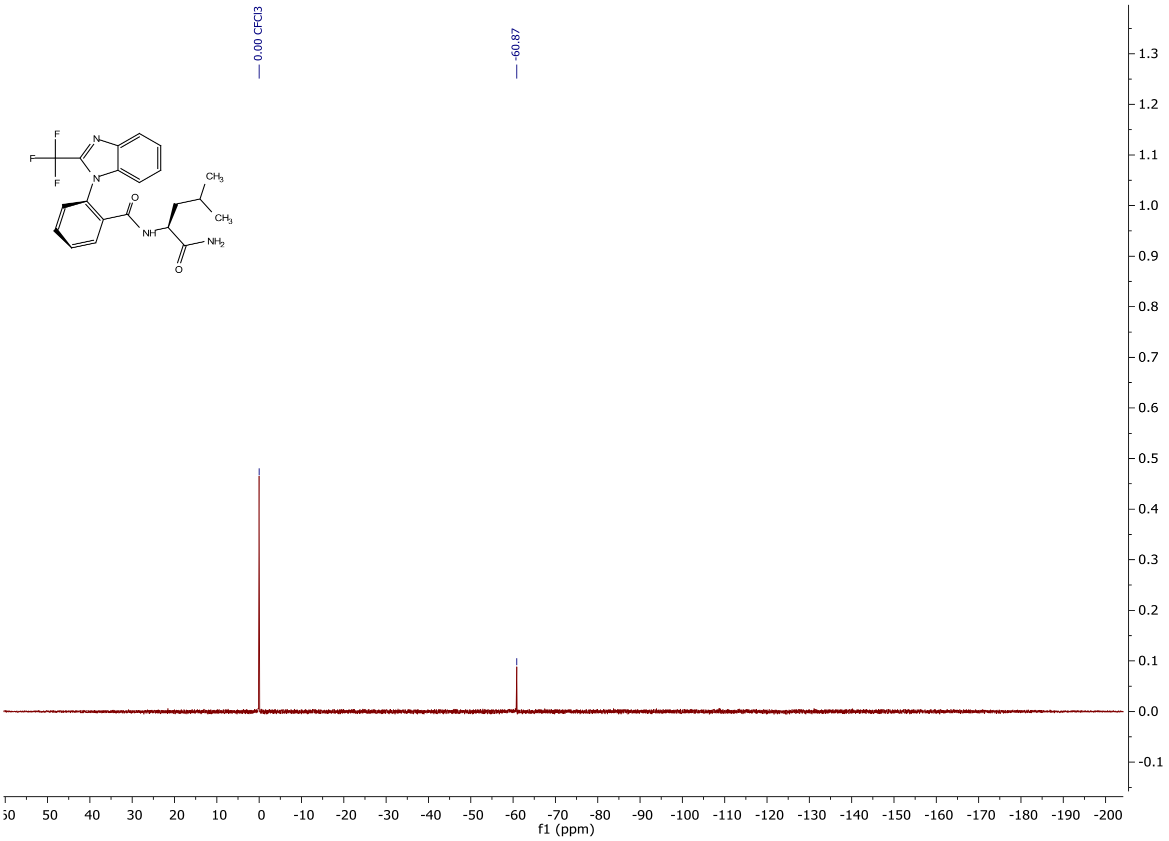

(*M*)-TBBA-(*L*)-Leu-NH_2 **(*M*)-11**

^{1}H NMR (400 MHz, DMSO-d_6)

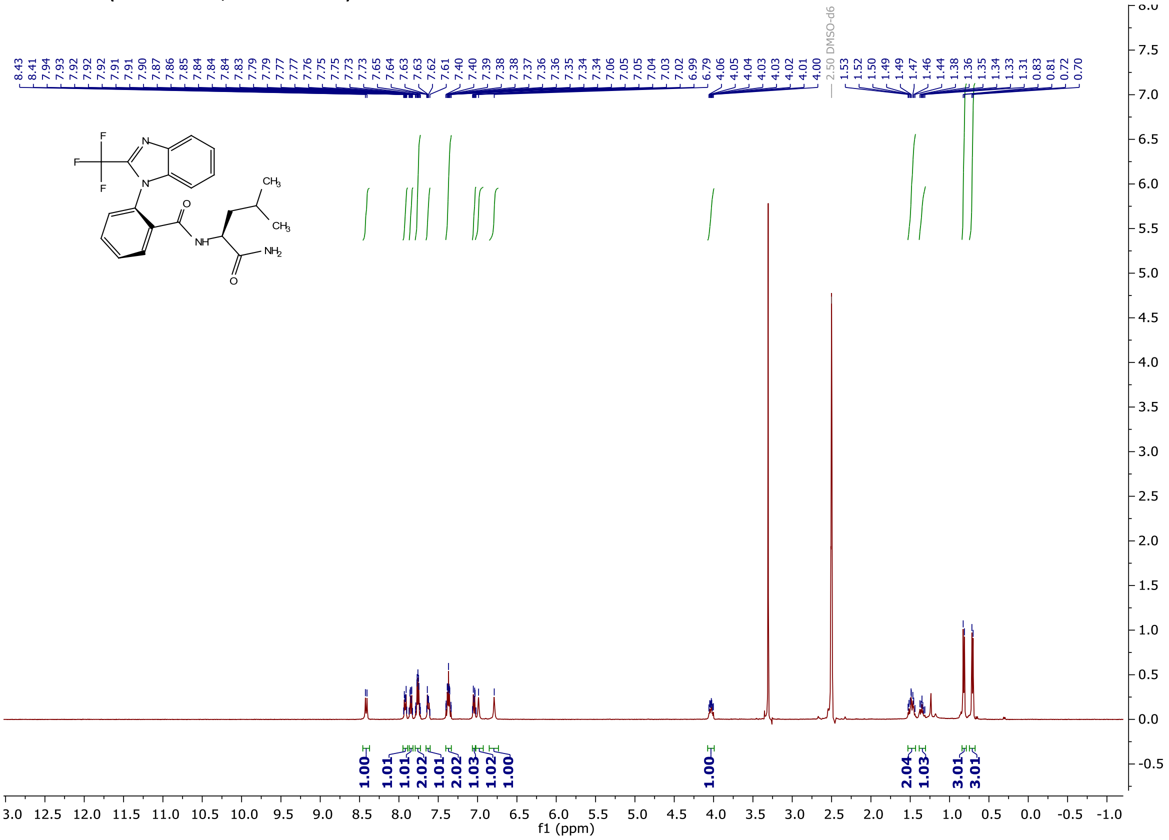

^{13}C{^{1}H} NMR (101 MHz, DMSO-d_6)

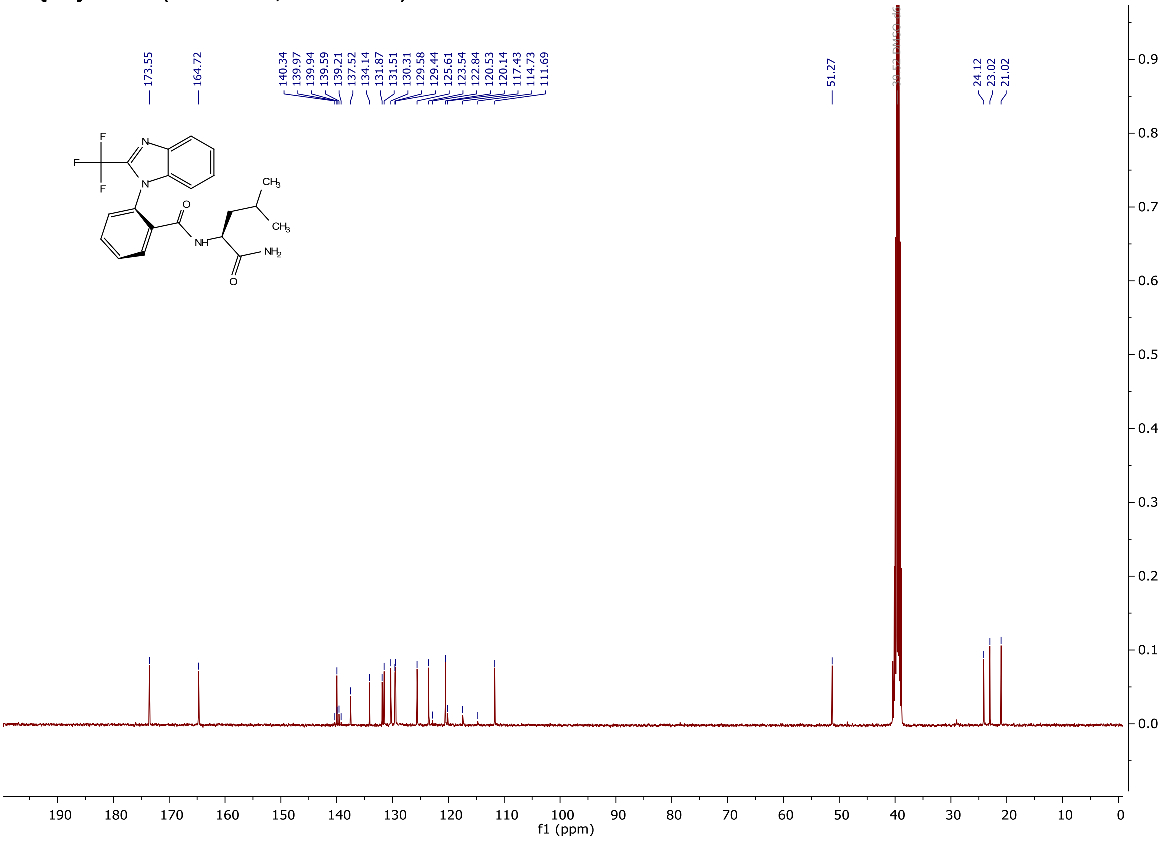

^{19}**F NMR** (376 MHz, DMSO-d_6)

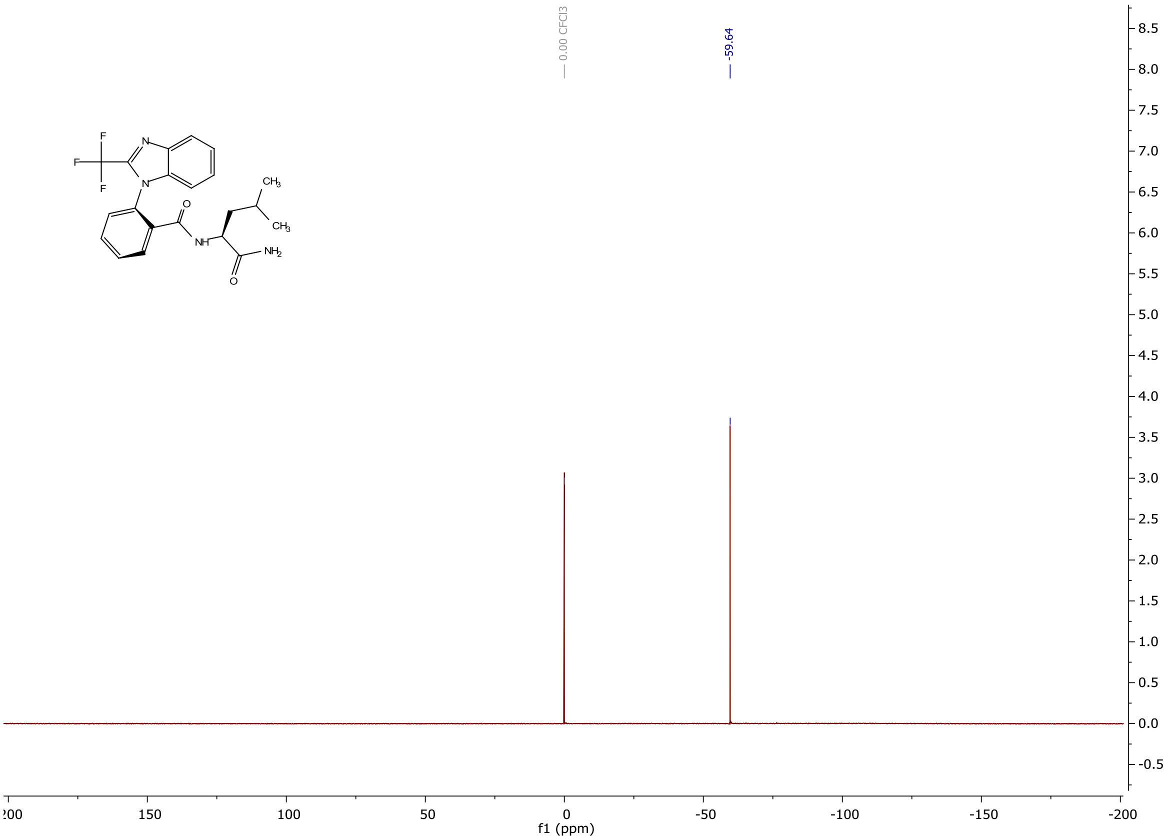

^{19}**F NMR** (76 MHz, THF)

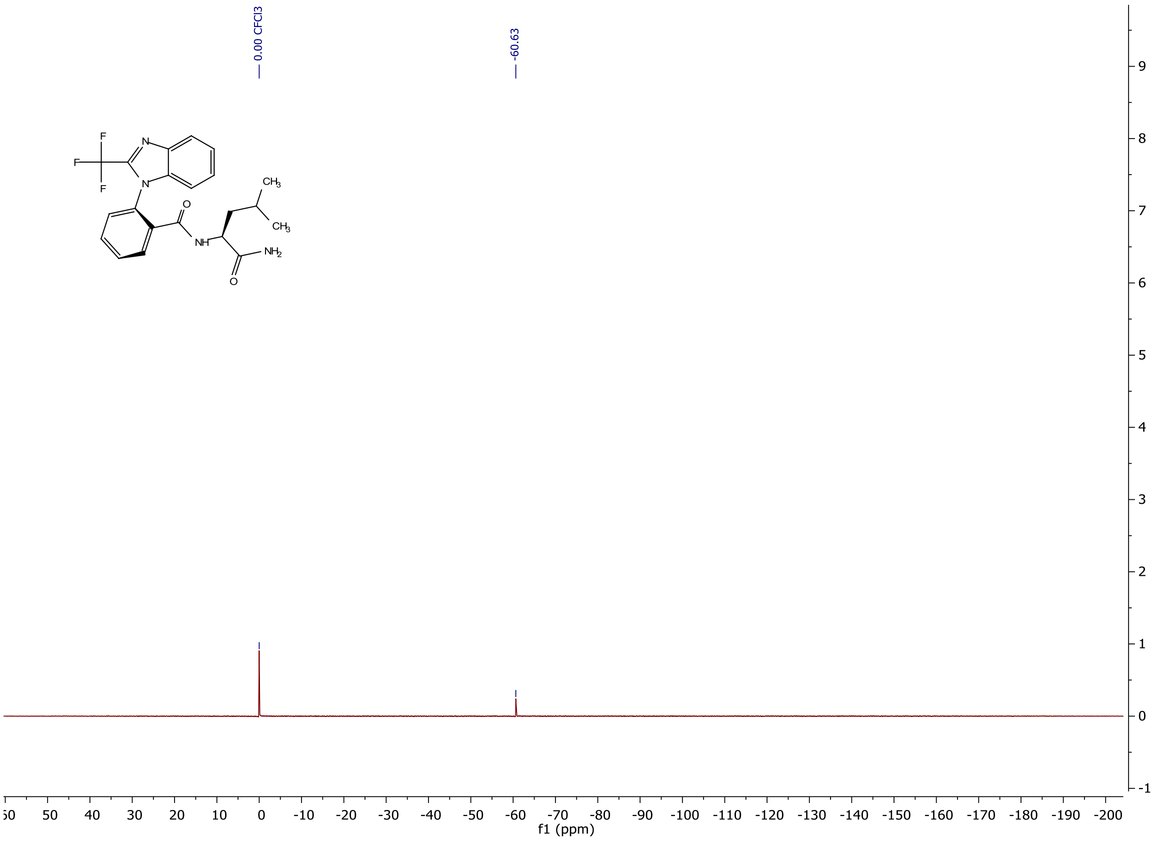

(*P*)-TBBA-(*L*)-Met-NH$_2$ **(*P*)-12**

^{1}**H NMR** (400 MHz, DMSO-d_6)

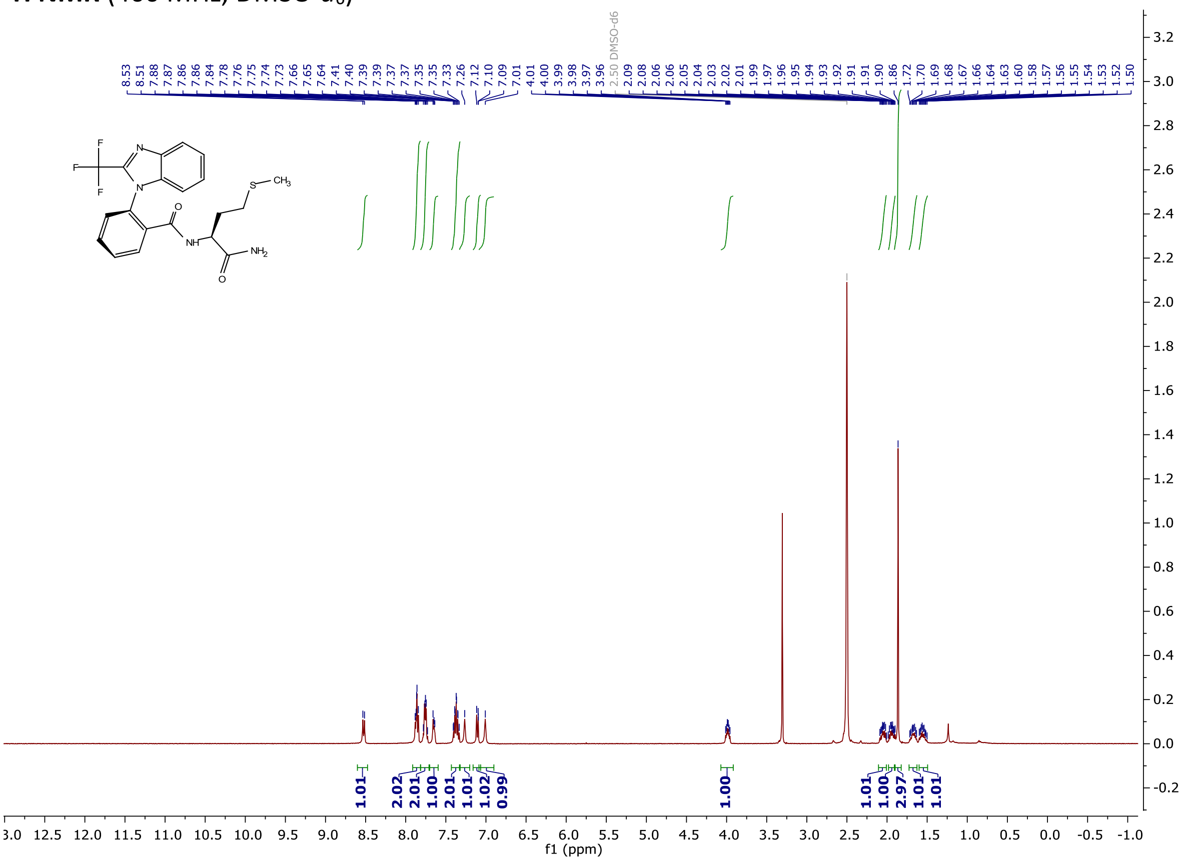

^{13}**C{^{1}H} NMR** (101 MHz, DMSO-d_6)

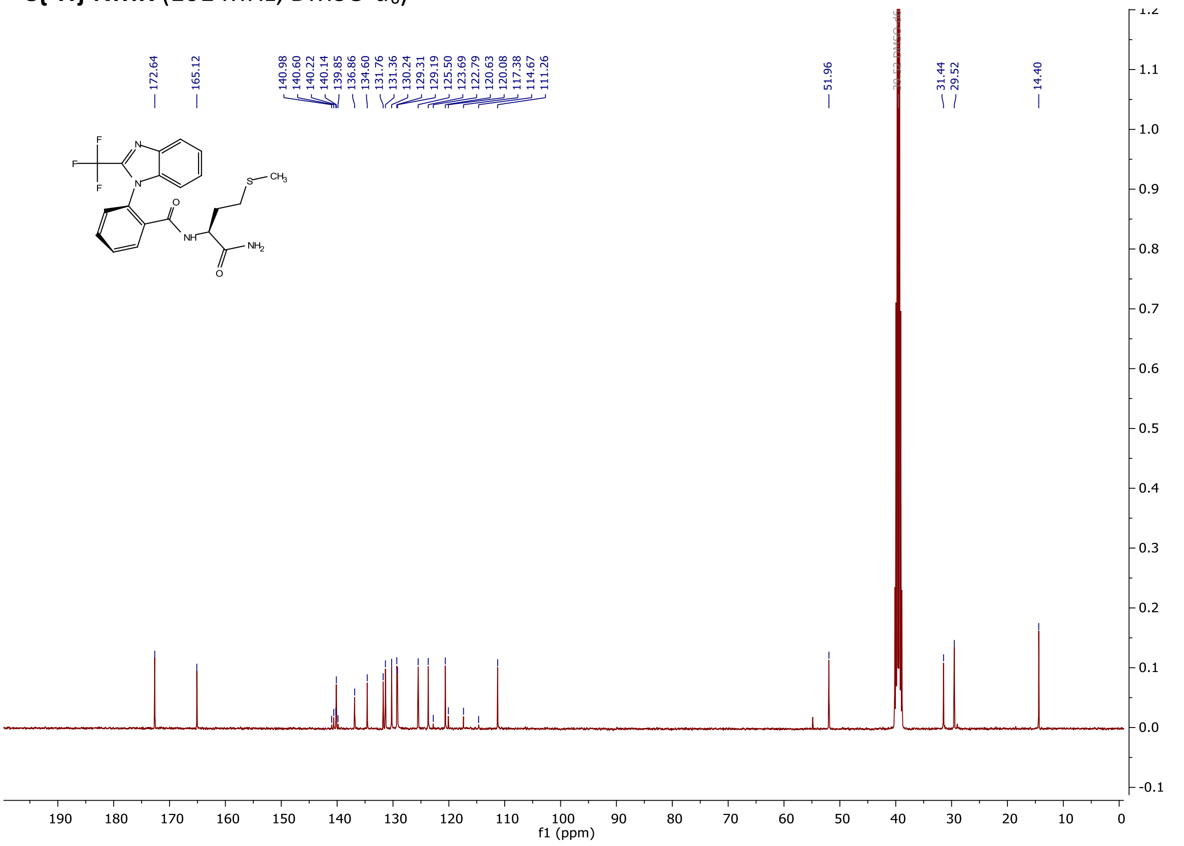

^{19}**F NMR** (376 MHz, DMSO-d_6)

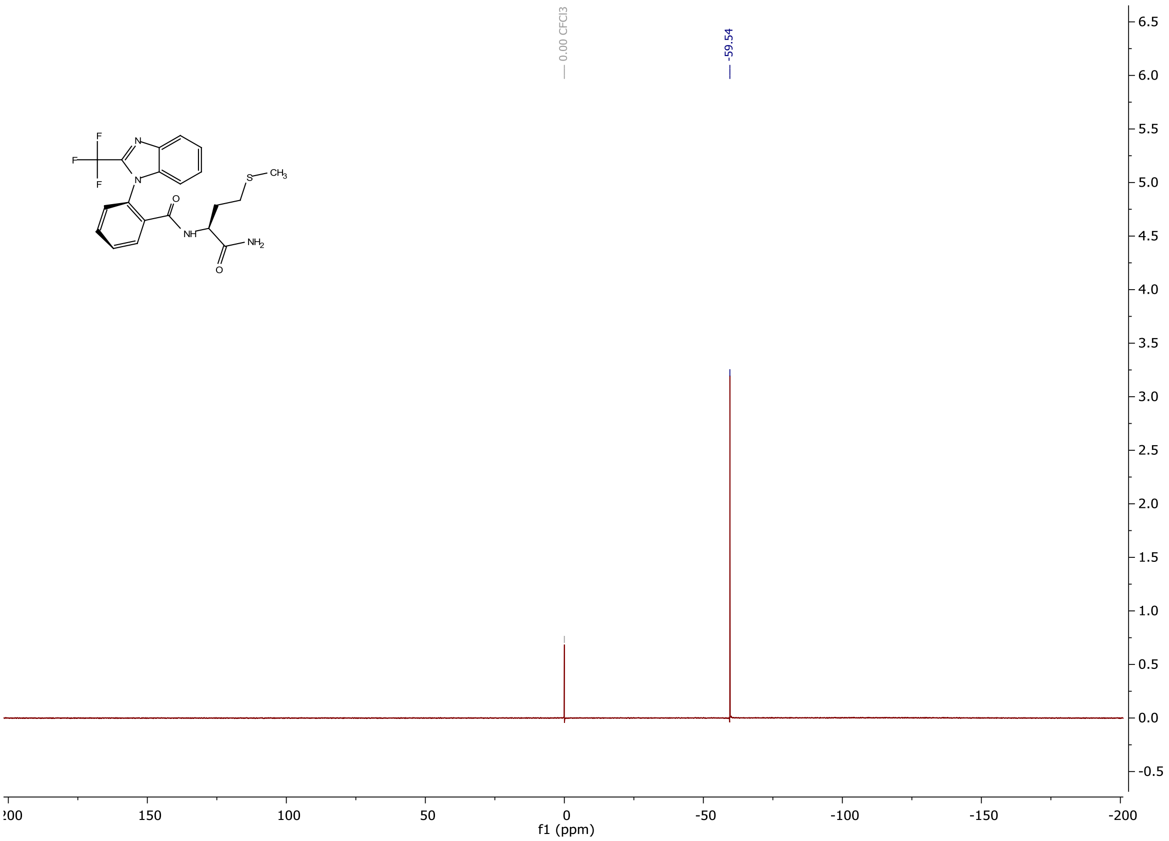

^{19}**F NMR** (76 MHz, THF)

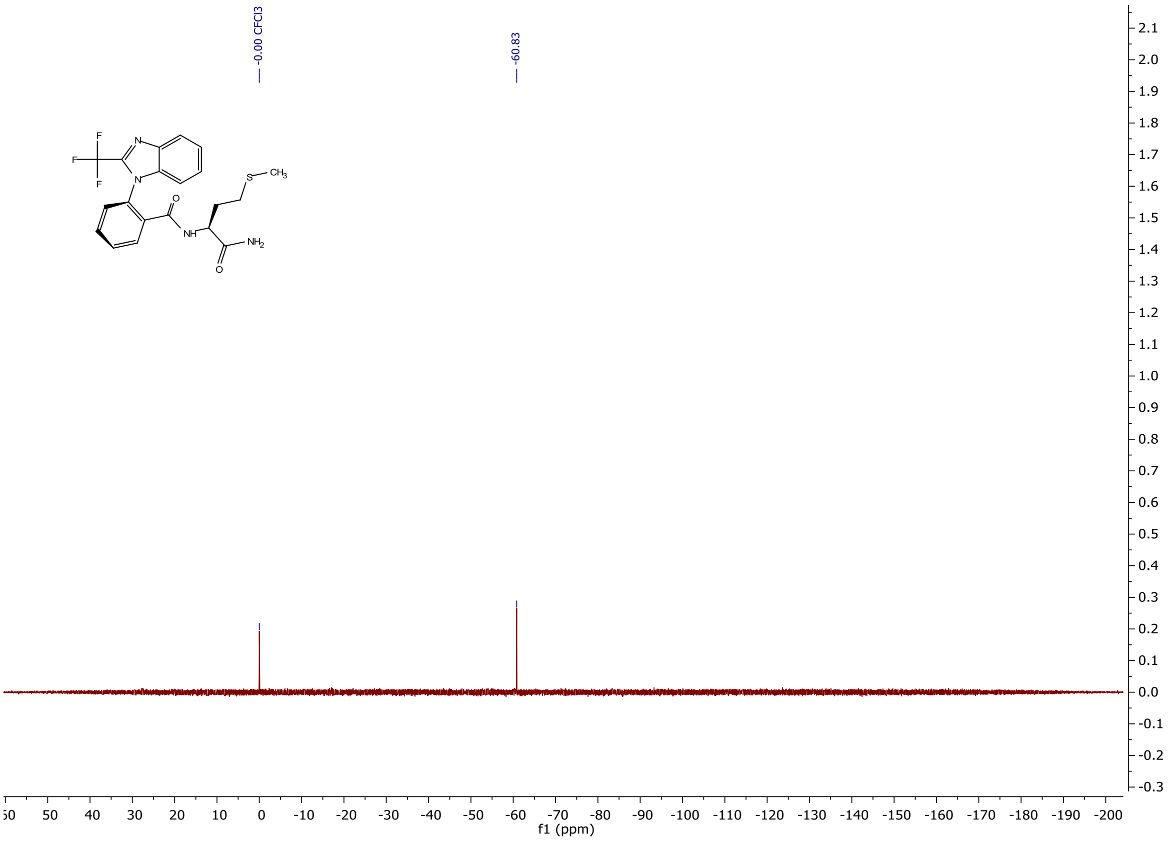

(*M*)-TBBA-(*L*)-Met-NH_2 **(*M*)-12**

^{1}H NMR (400 MHz, DMSO-d_6)

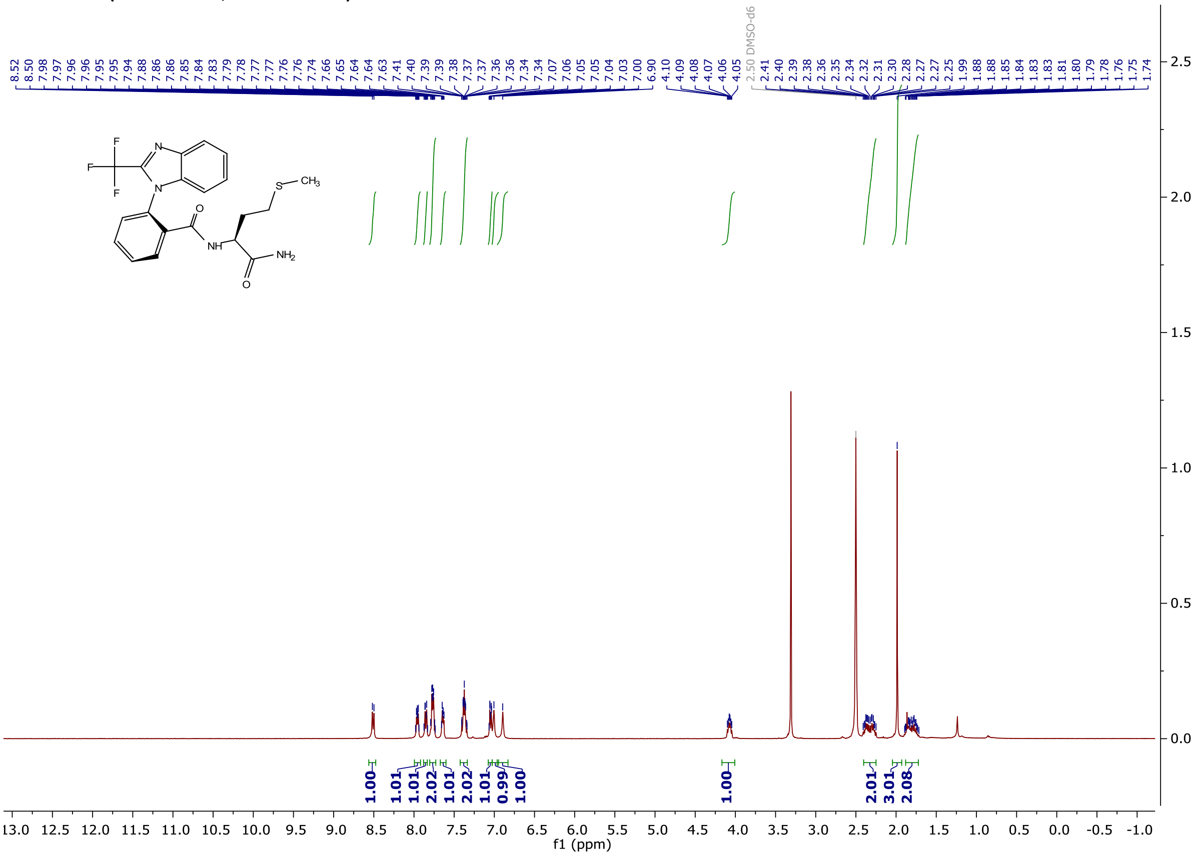

^{13}C{^{1}H} NMR (101 MHz, DMSO-d_6)

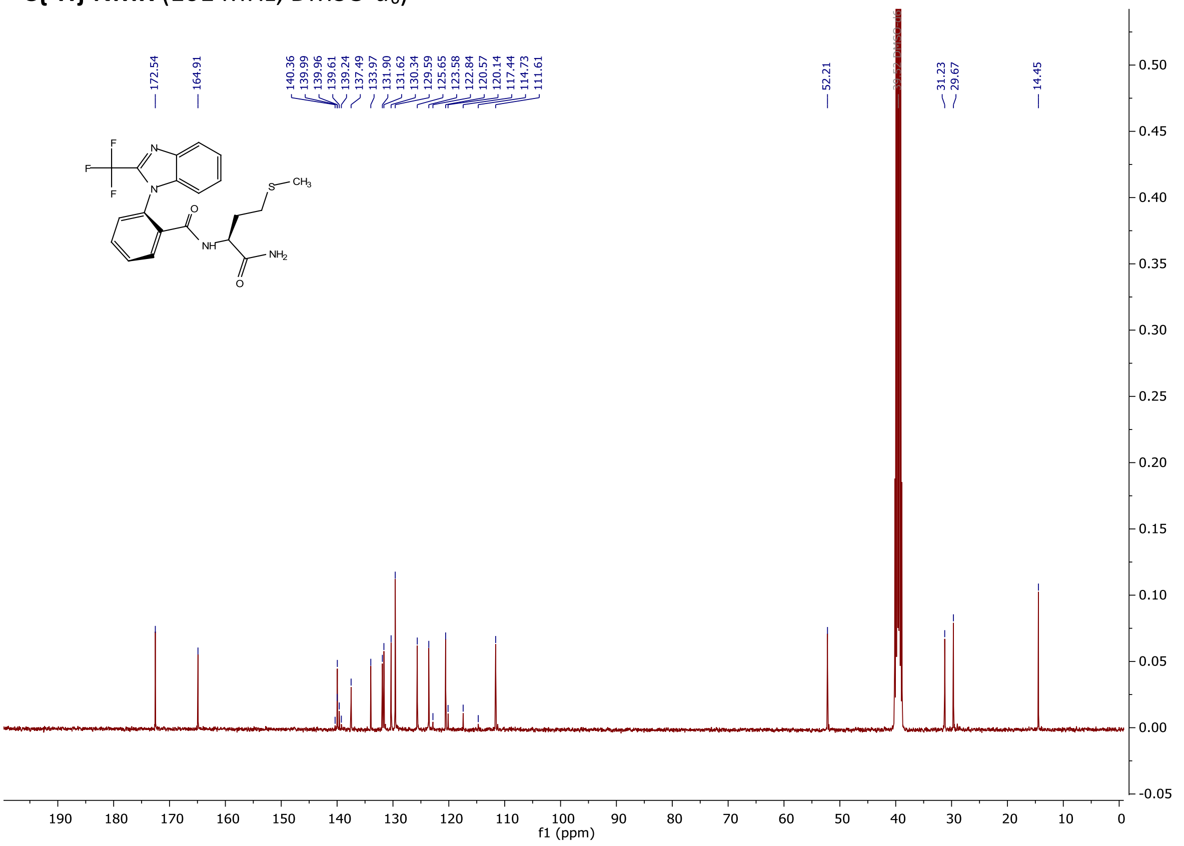

^{19}F NMR (376 MHz, DMSO-d_6)

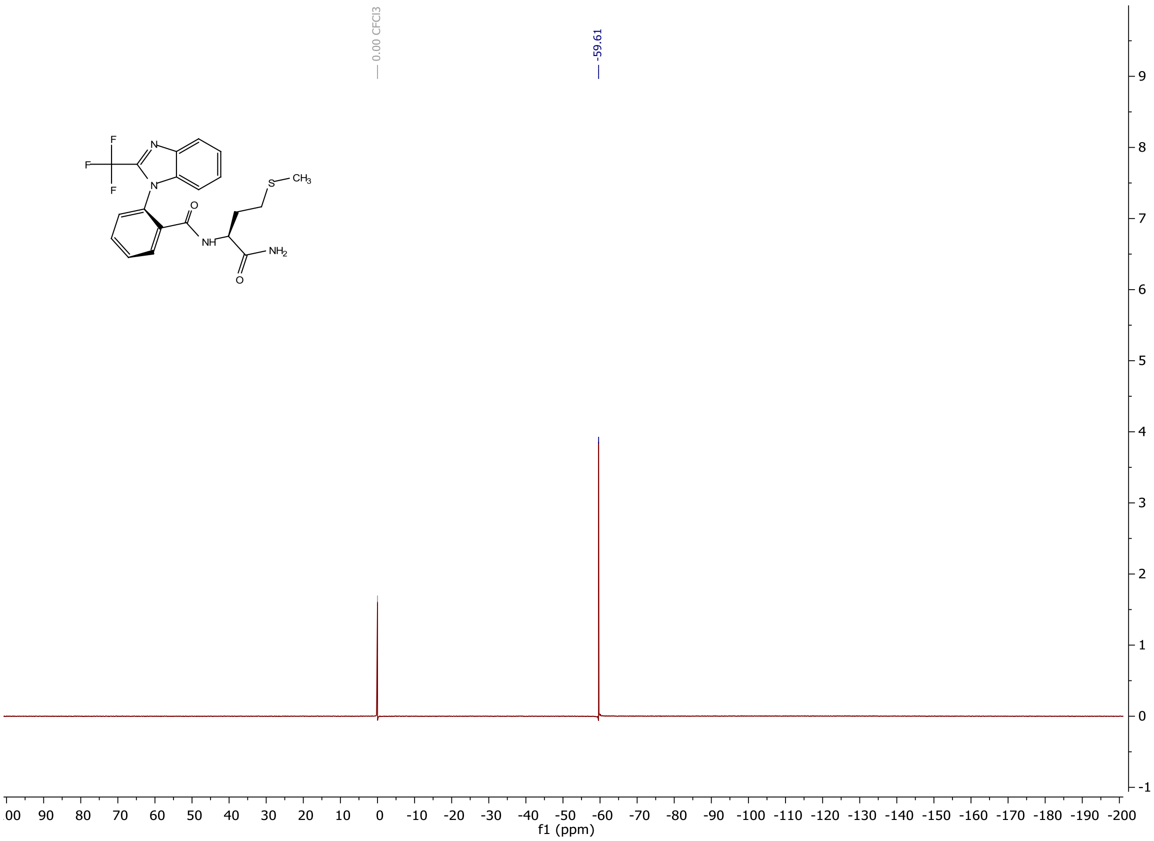

^{19}F NMR (76 MHz, THF)

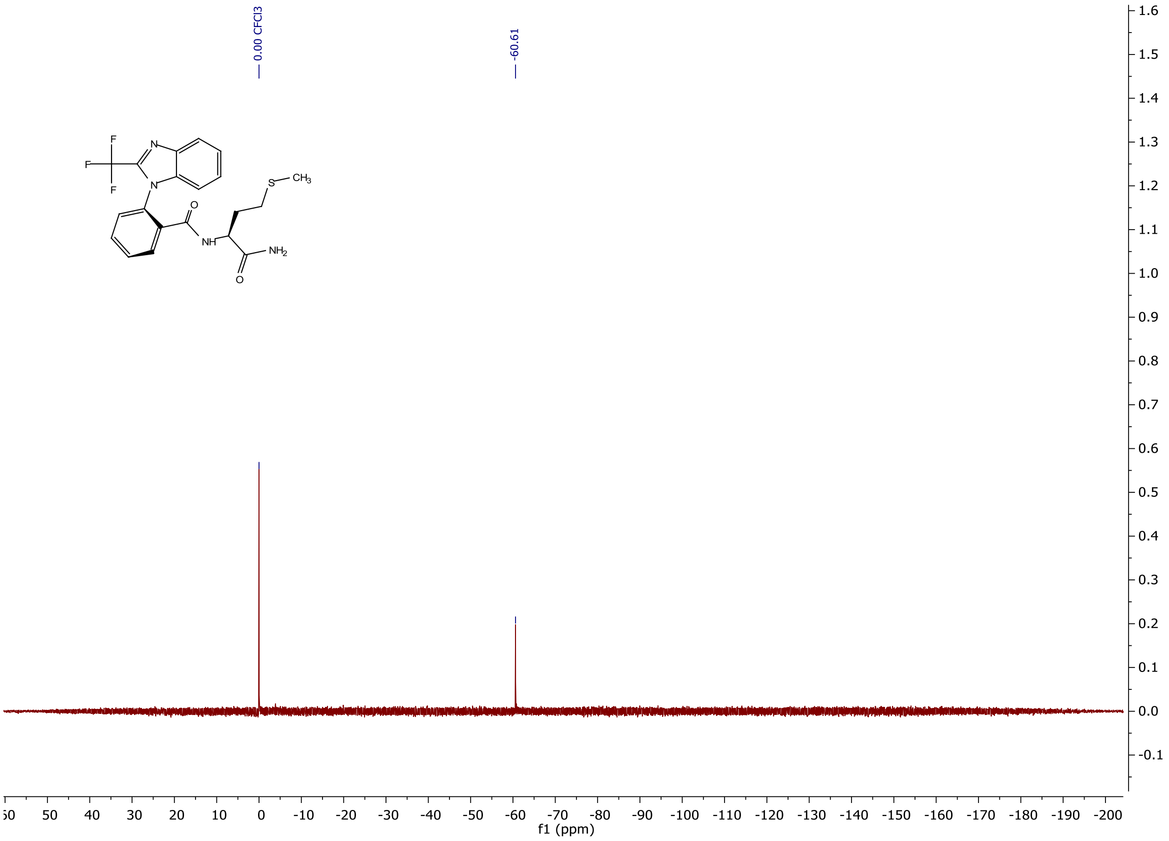

(*P*)-TBBA-(*L*)-Phe-NH_2 **(*P*)-13**

^{1}H NMR (400 MHz, DMSO-d_6)

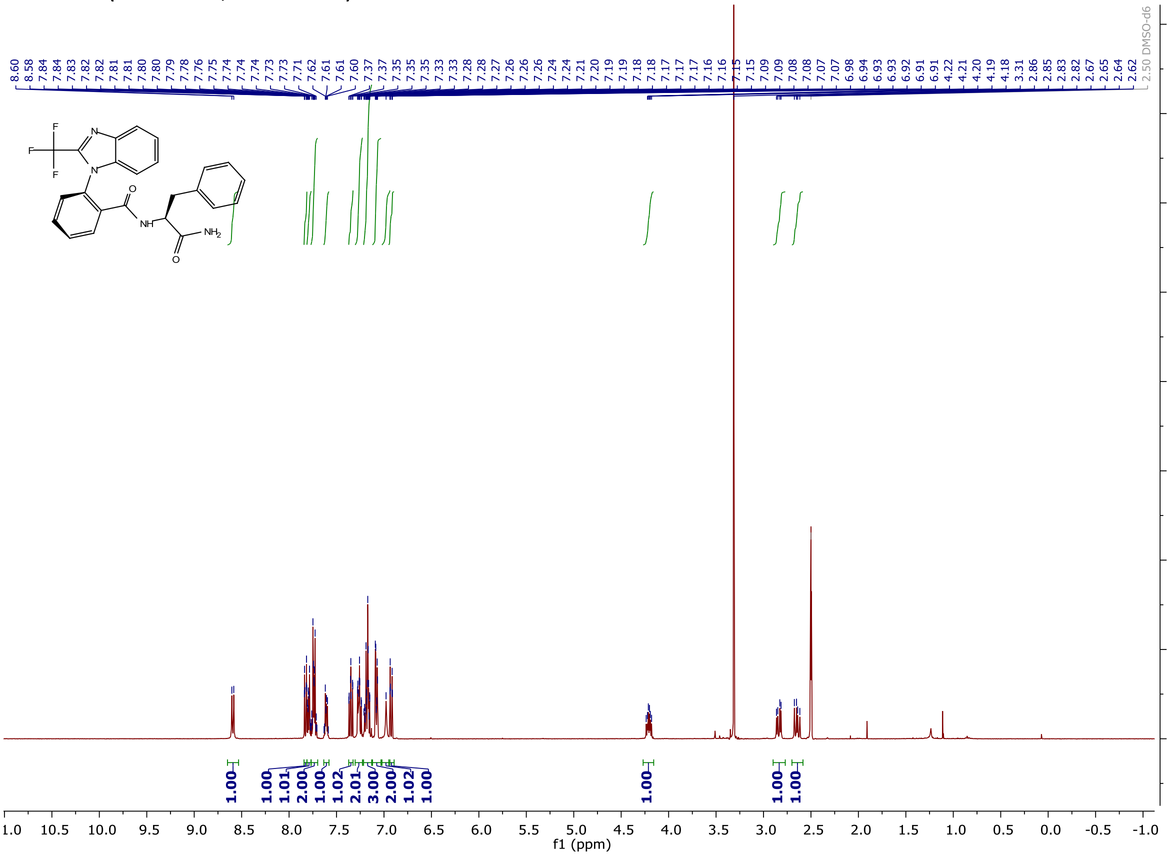

^{13}C{^{1}H} NMR (101 MHz, DMSO-d_6)

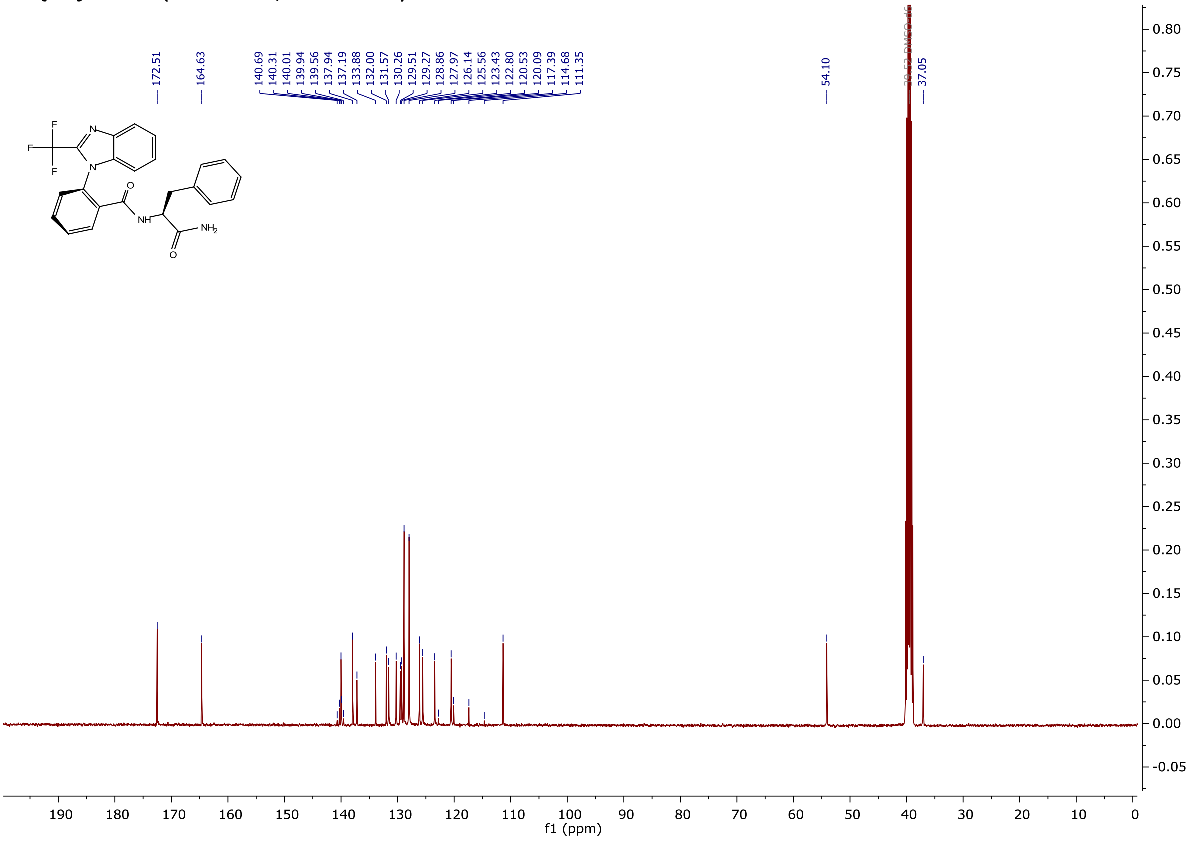

^{19}**F NMR** (376 MHz, DMSO-d_6)

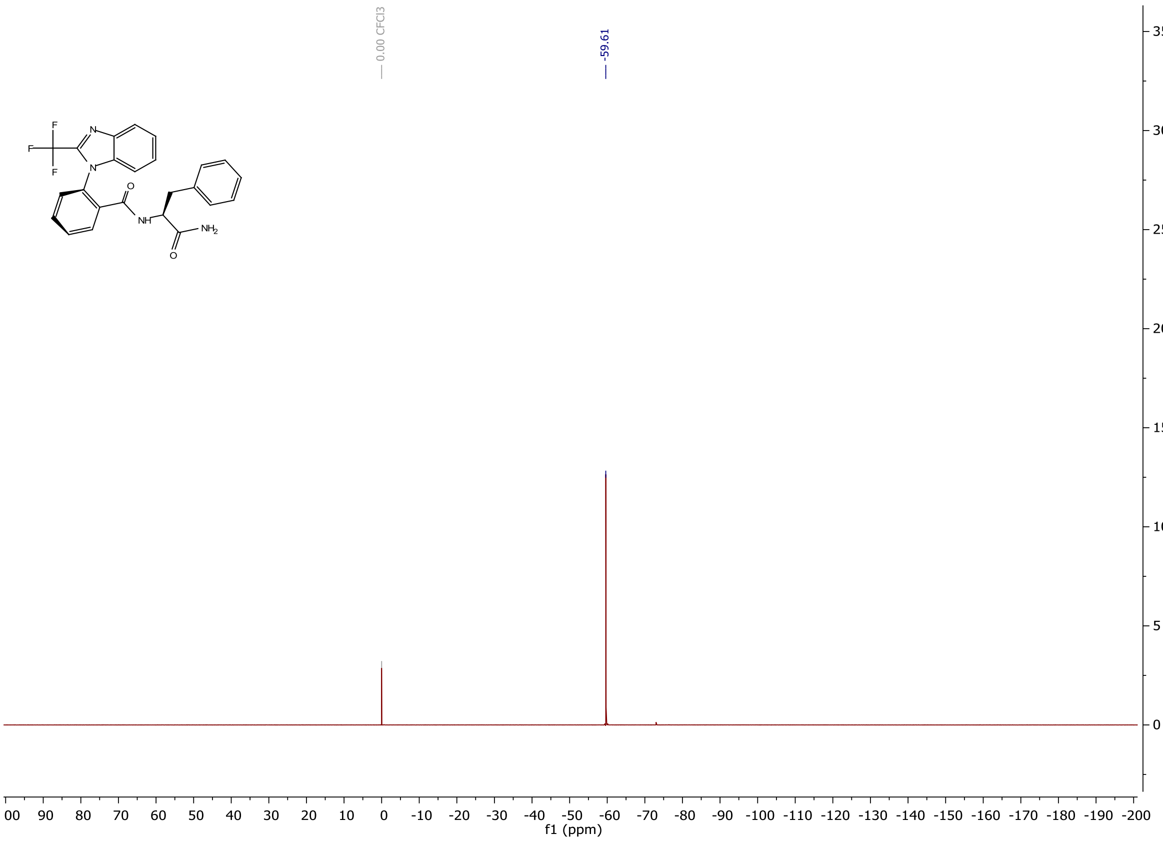

^{19}**F NMR** (76 MHz, THF)

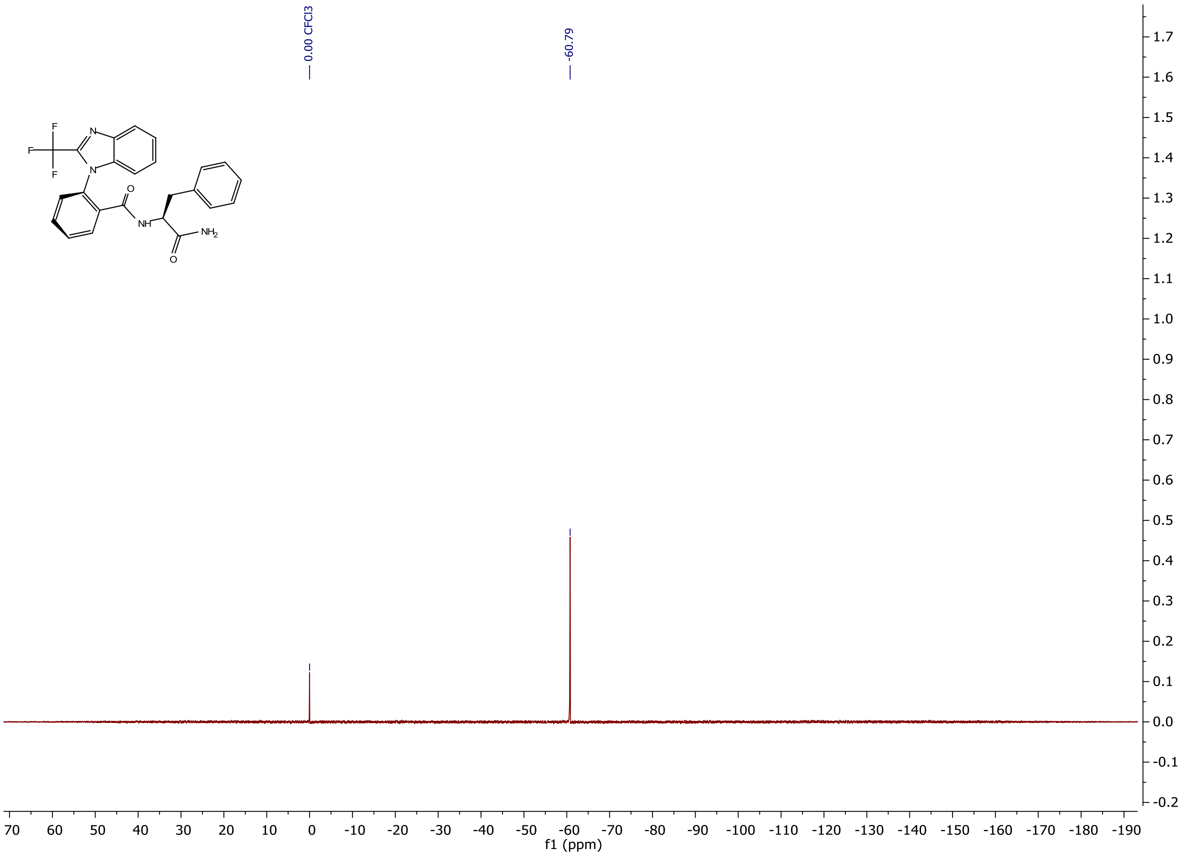

(*M*)-TBBA-(*L*)-Phe-NH_2 **(*M*)-13**

^{1}**H NMR** (400 MHz, DMSO-d_6)

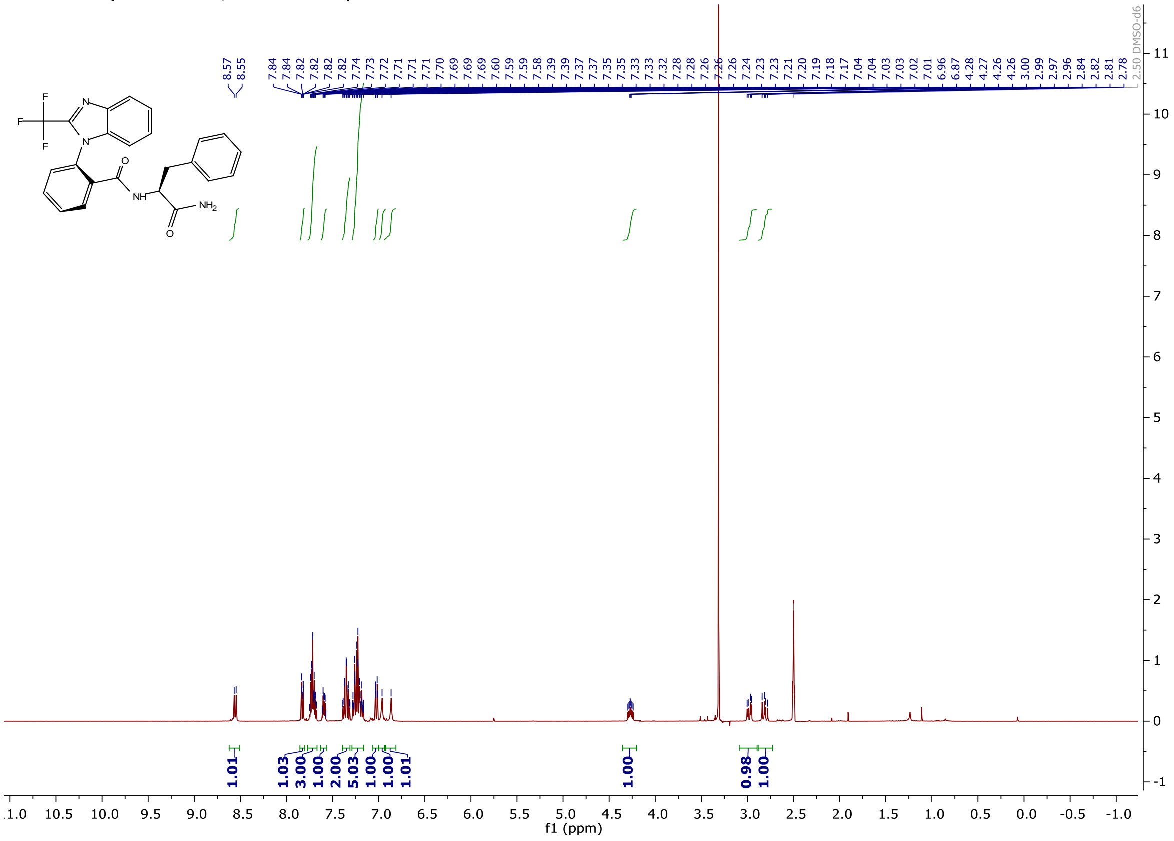

^{13}**C{^{1}H} NMR** (101 MHz, DMSO-d_6)

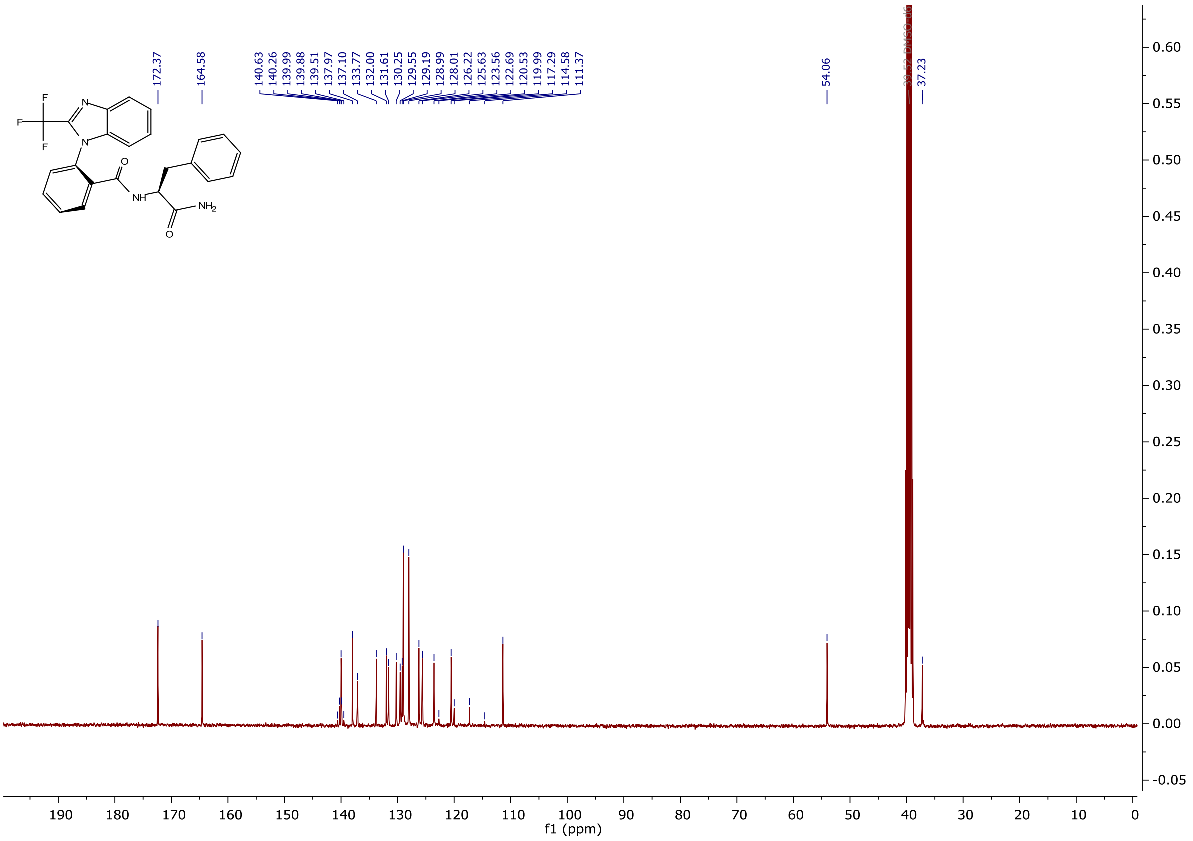

^{19}F NMR (376 MHz, DMSO-d_6)

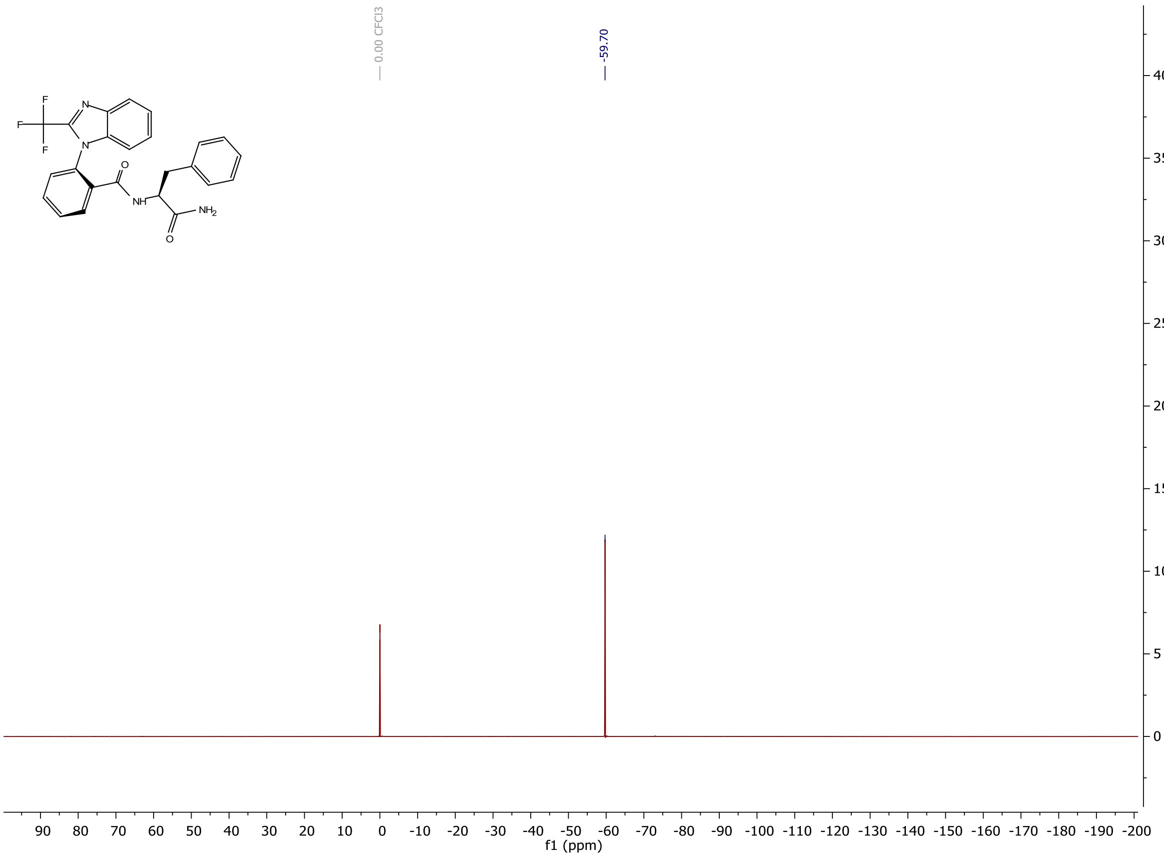

^{19}F NMR (76 MHz, THF)

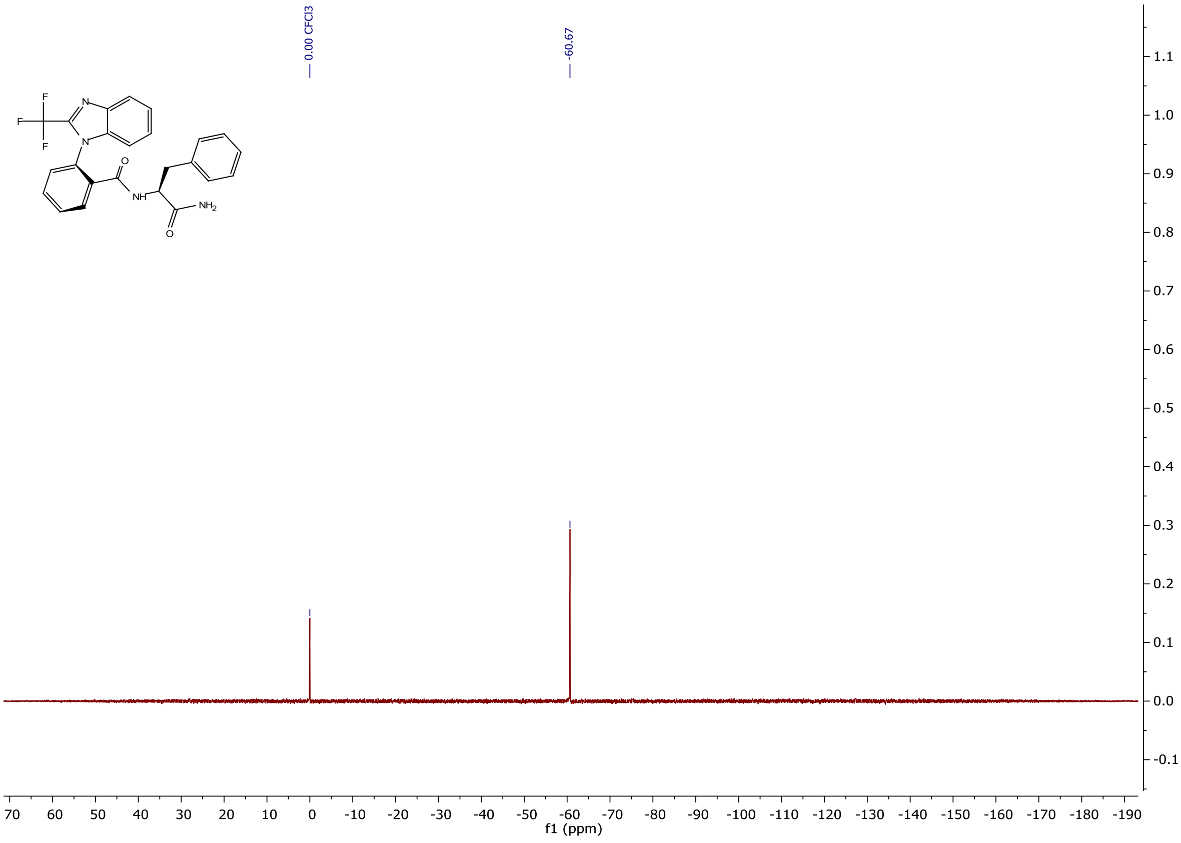

(*P*)-TBBA-(*L*)-Tyr-NH_2 **(*P*)-14**

^{1}**H NMR** (400 MHz, DMSO-d_6)

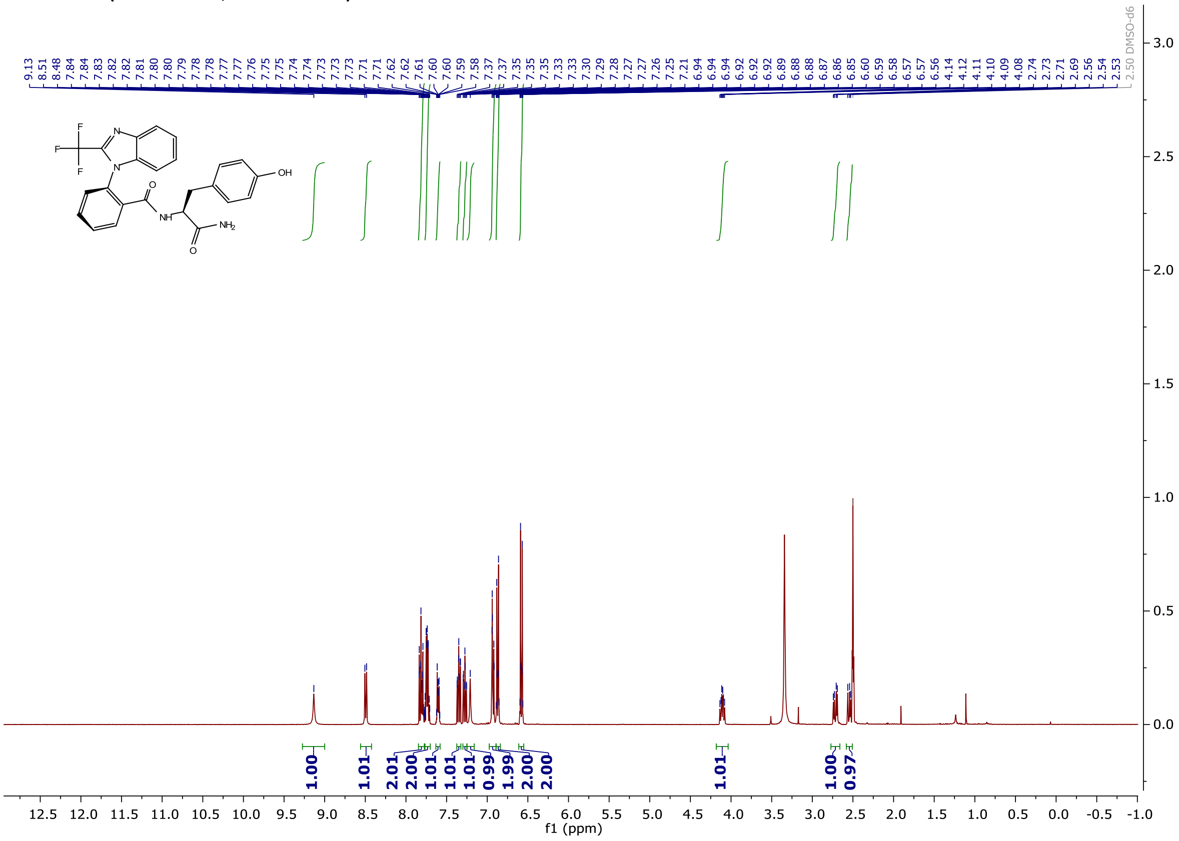

^{13}**C{^{1}H} NMR** (101 MHz, DMSO-d_6)

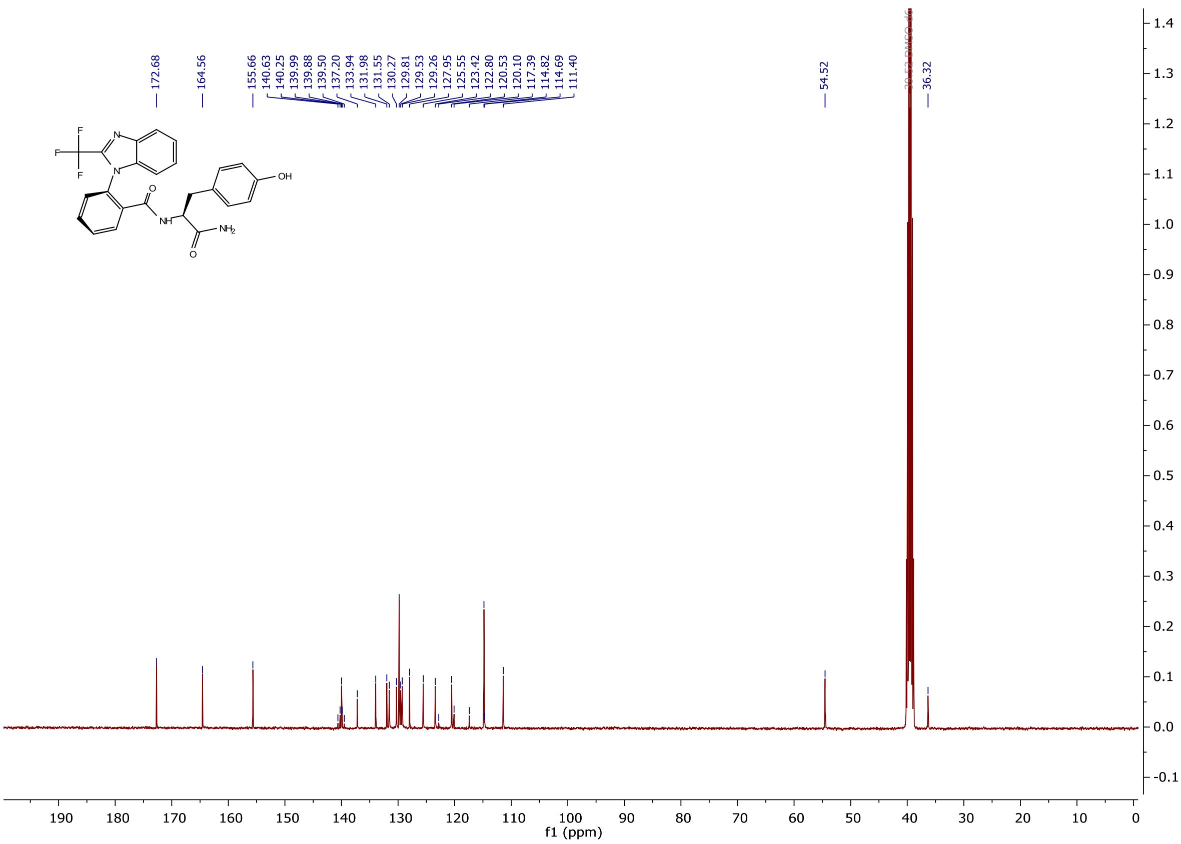

^{19}**F NMR** (376 MHz, DMSO-d_6)

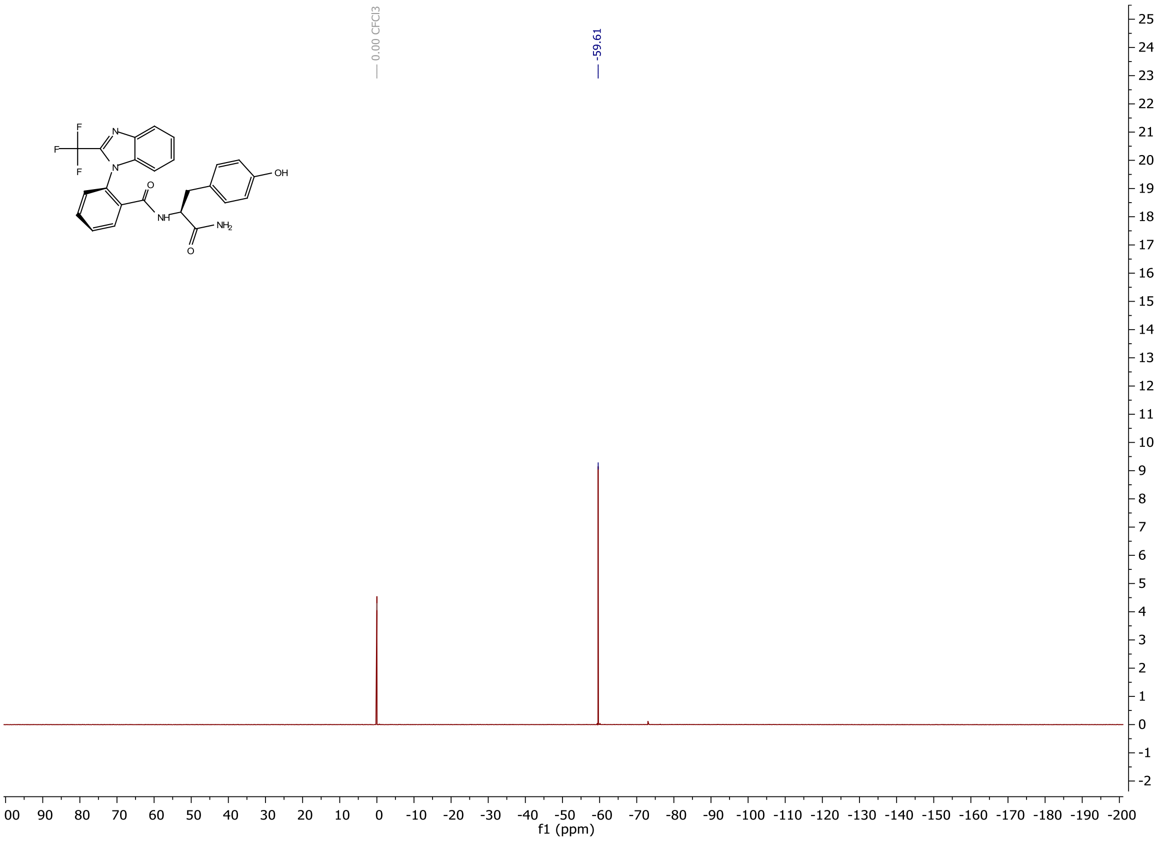

^{19}**F NMR** (76 MHz, THF)

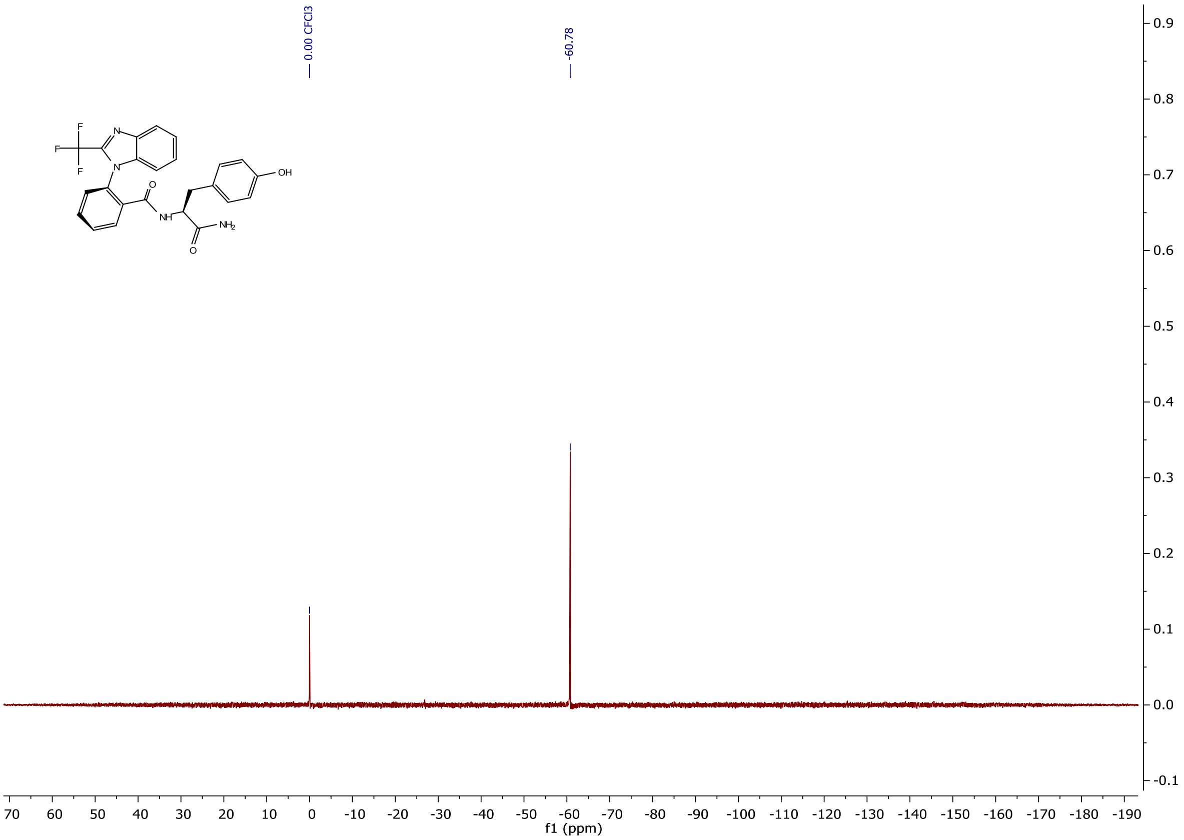

(*M*)-TBBA-(*L*)-Tyr-NH2 **(*M*)-14**

^{1}H NMR (400 MHz, DMSO-d_6)

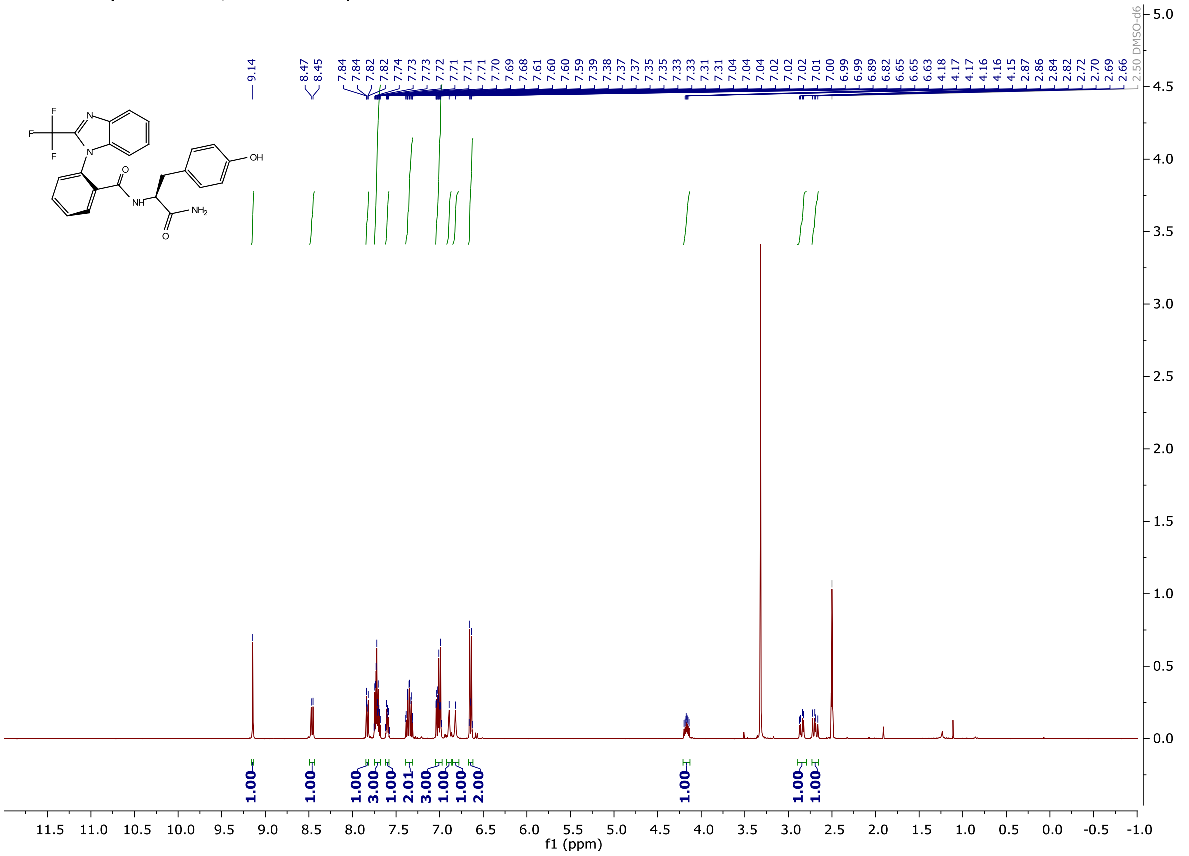

^{13}C{^{1}H} NMR (101 MHz, DMSO-d_6)

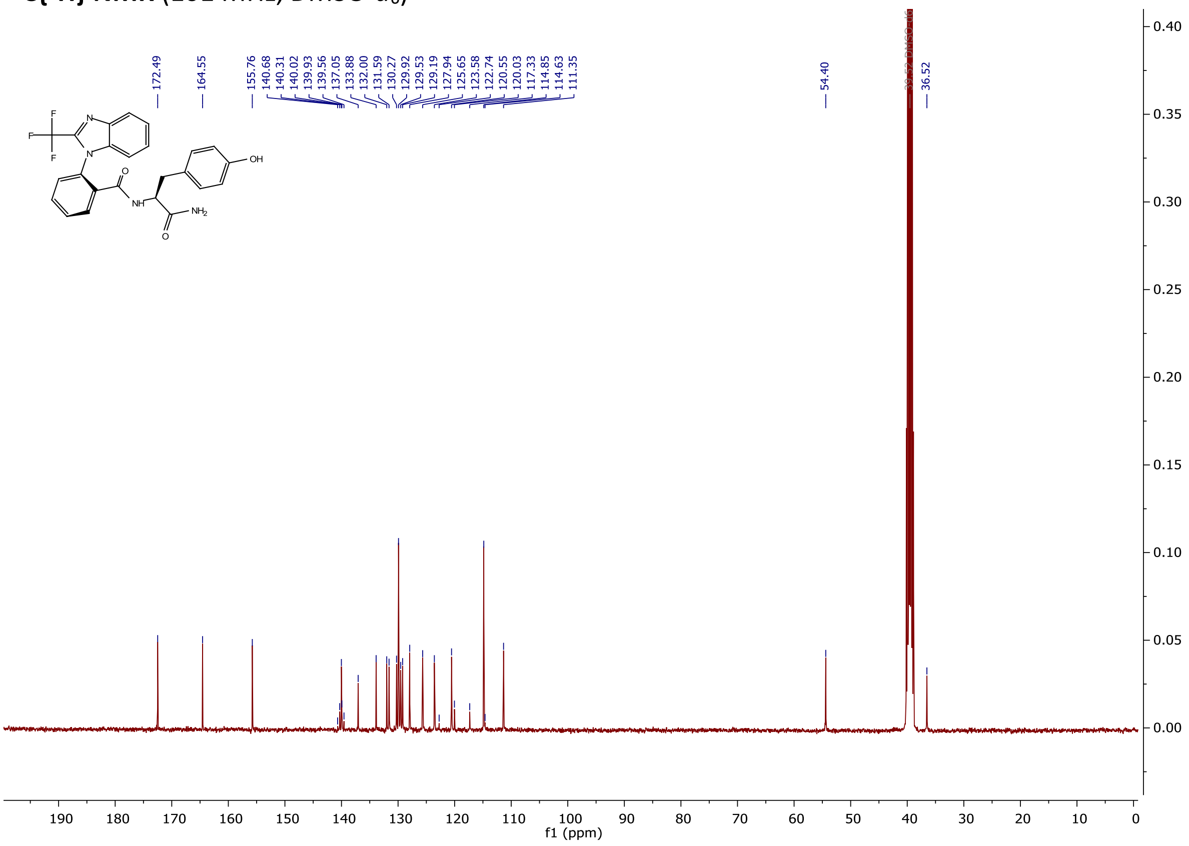

^{19}F NMR (376 MHz, DMSO-d_6)

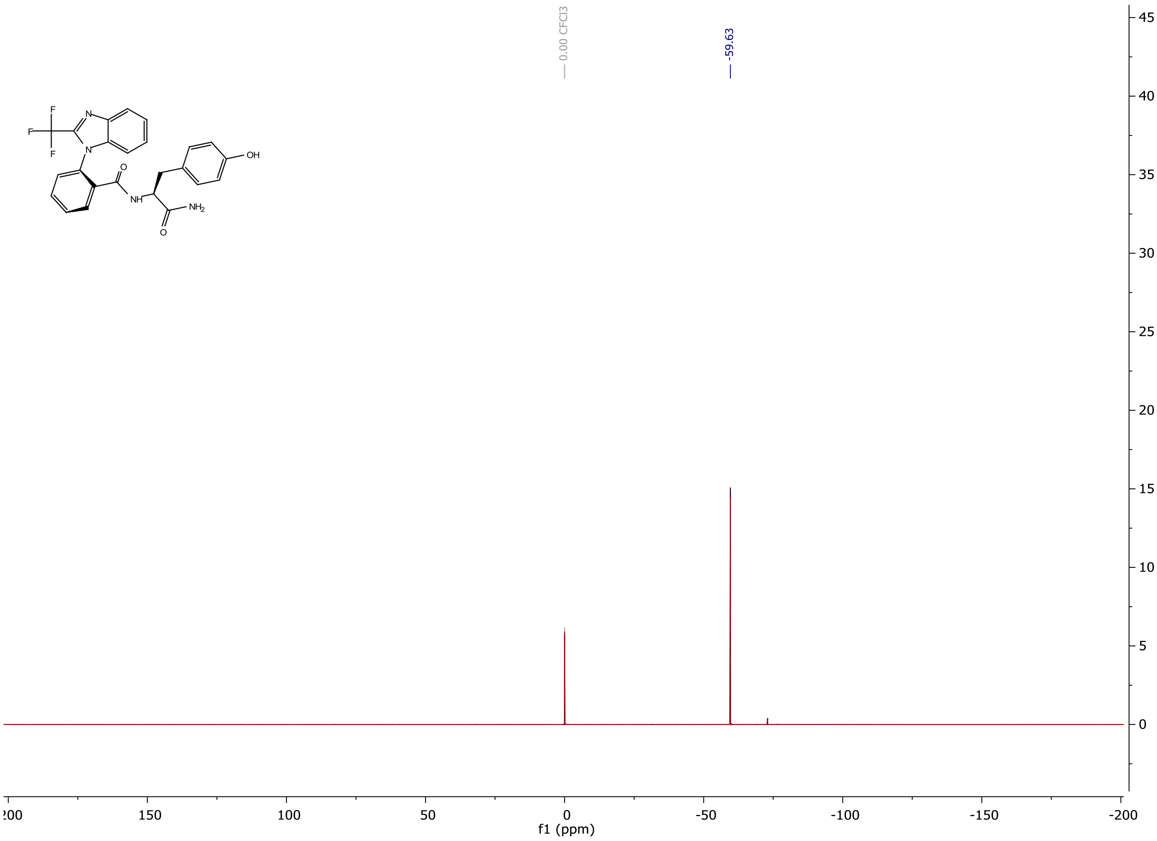

^{19}F NMR (76 MHz, THF)

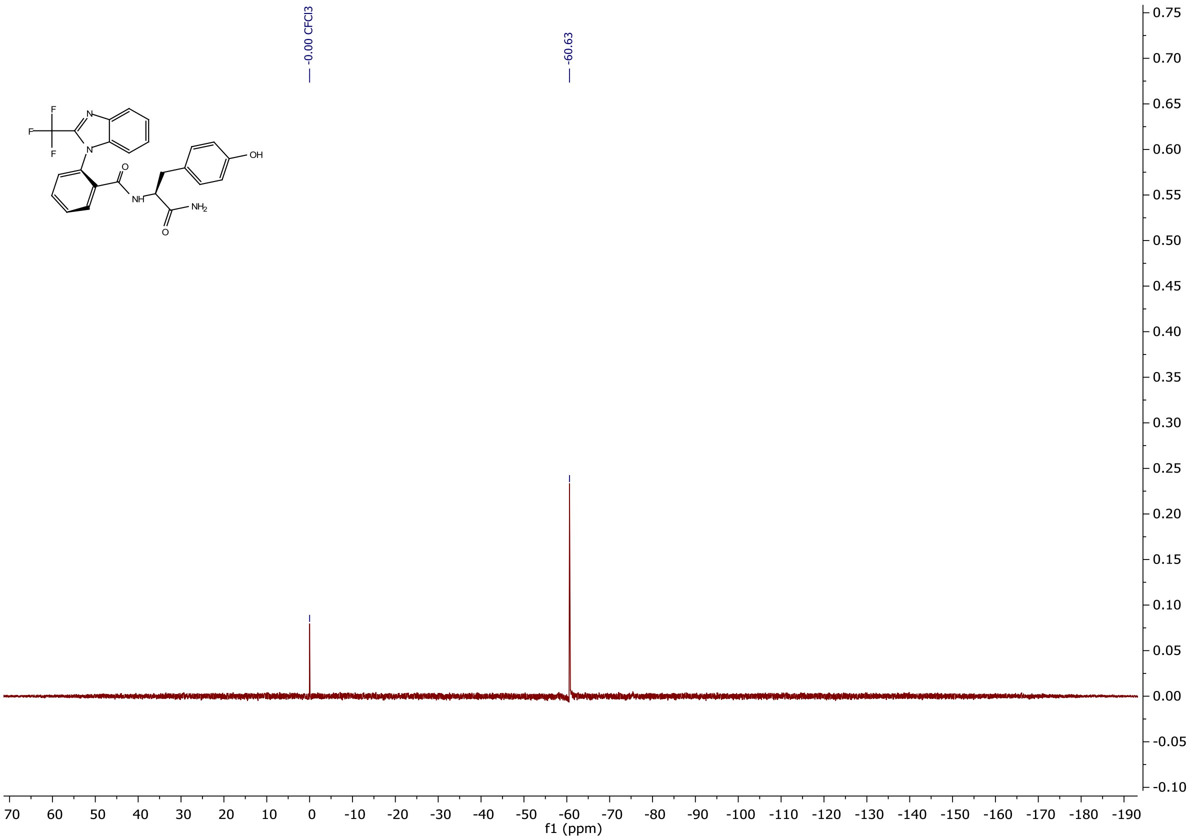

(*P*)-TBBA-(*L*)-Trp-NH_2 **(*P*)-15**

^{1}H NMR (400 MHz, DMSO-d_6)

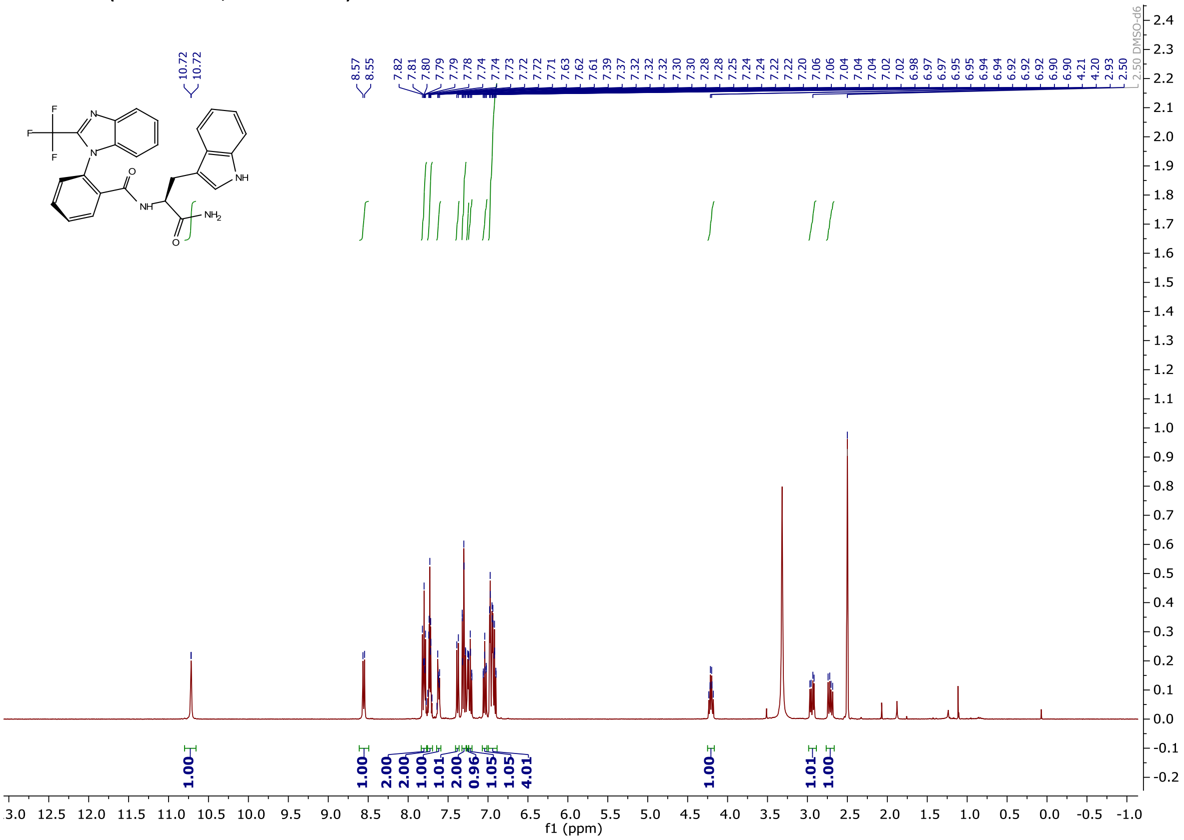

^{13}C{^{1}H} NMR (101 MHz, DMSO-d_6)

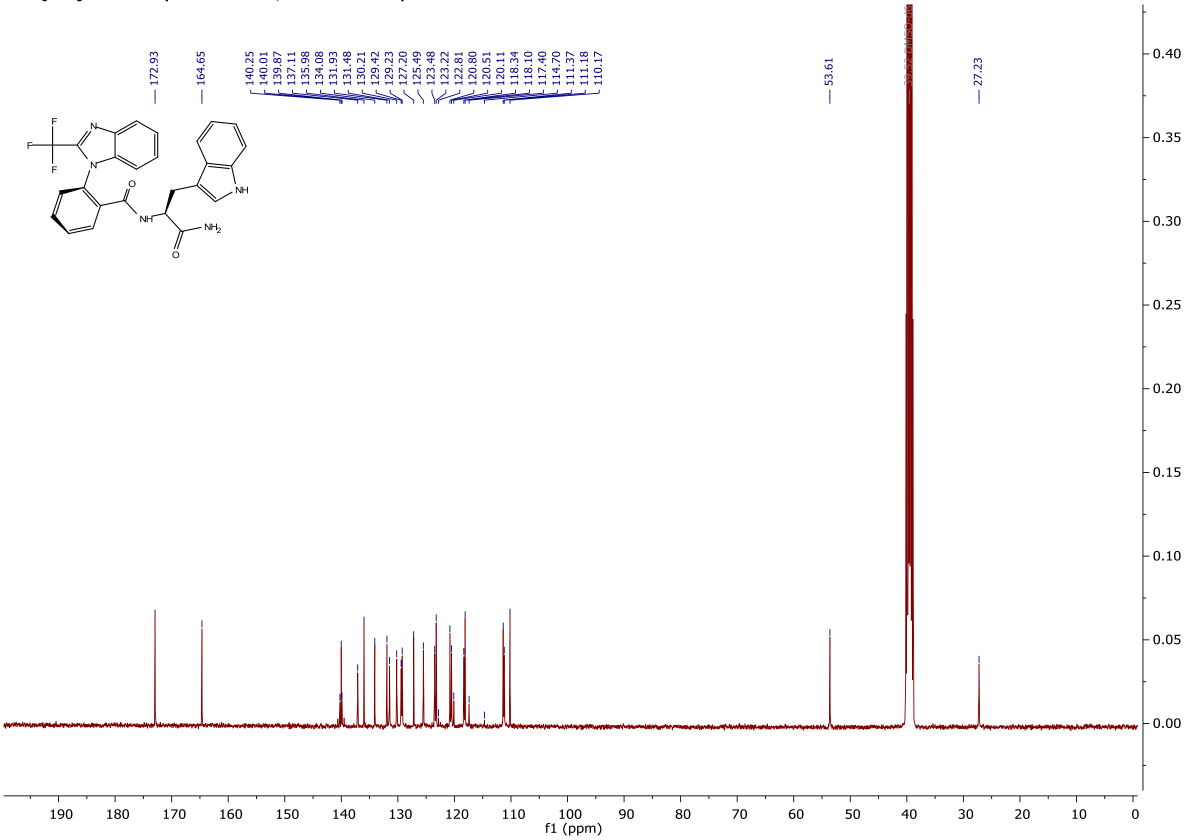

^{19}**F NMR** (376 MHz, DMSO-d_6)

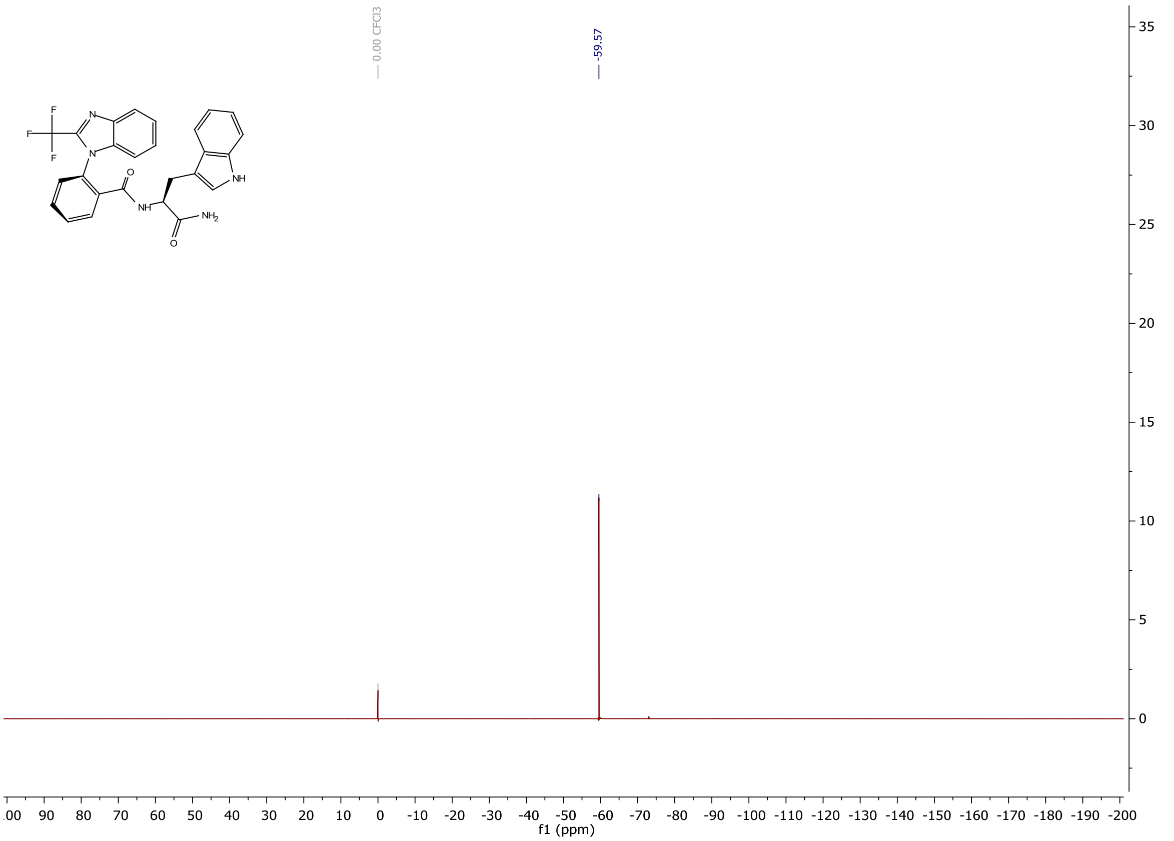

^{19}**F NMR** (76 MHz, THF)

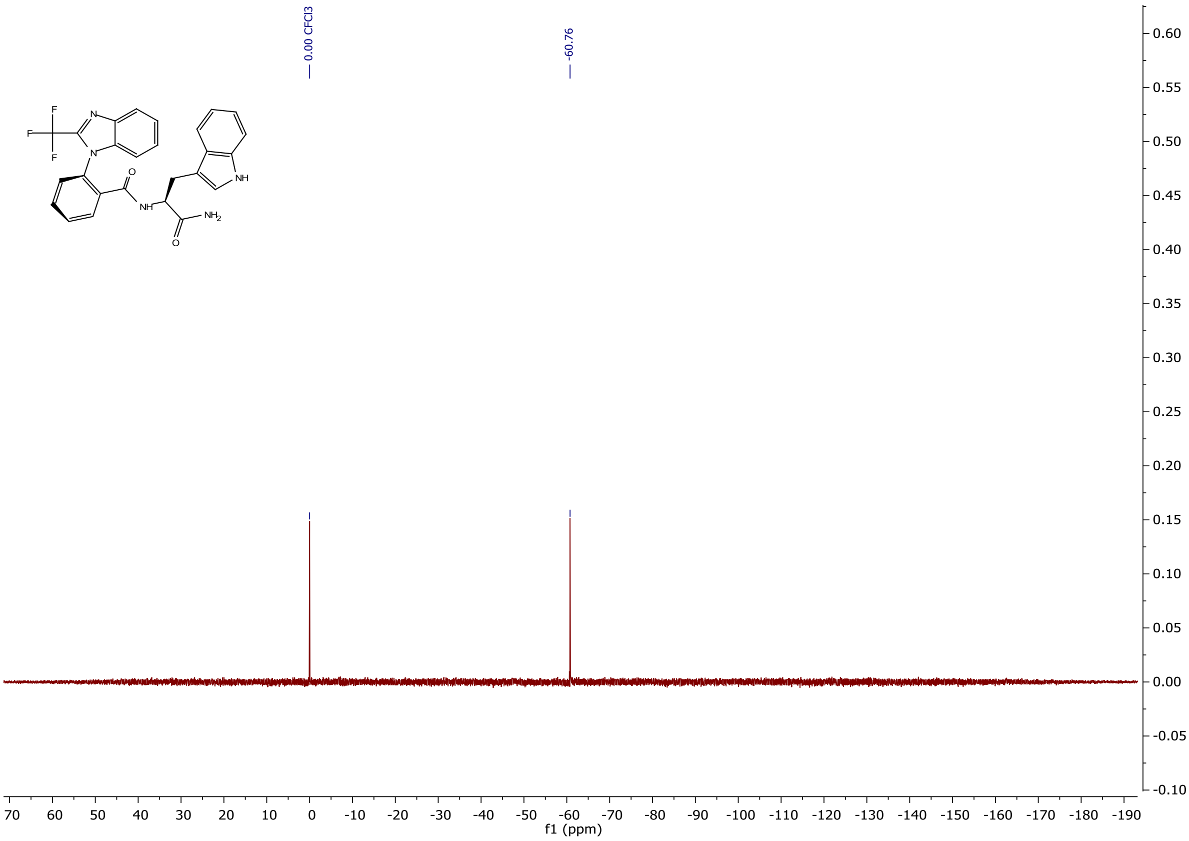

(*M*)-TBBA-(*L*)-Trp-NH_2 **(*M*)-15**

^{1}**H NMR** (400 MHz, DMSO-d_6)

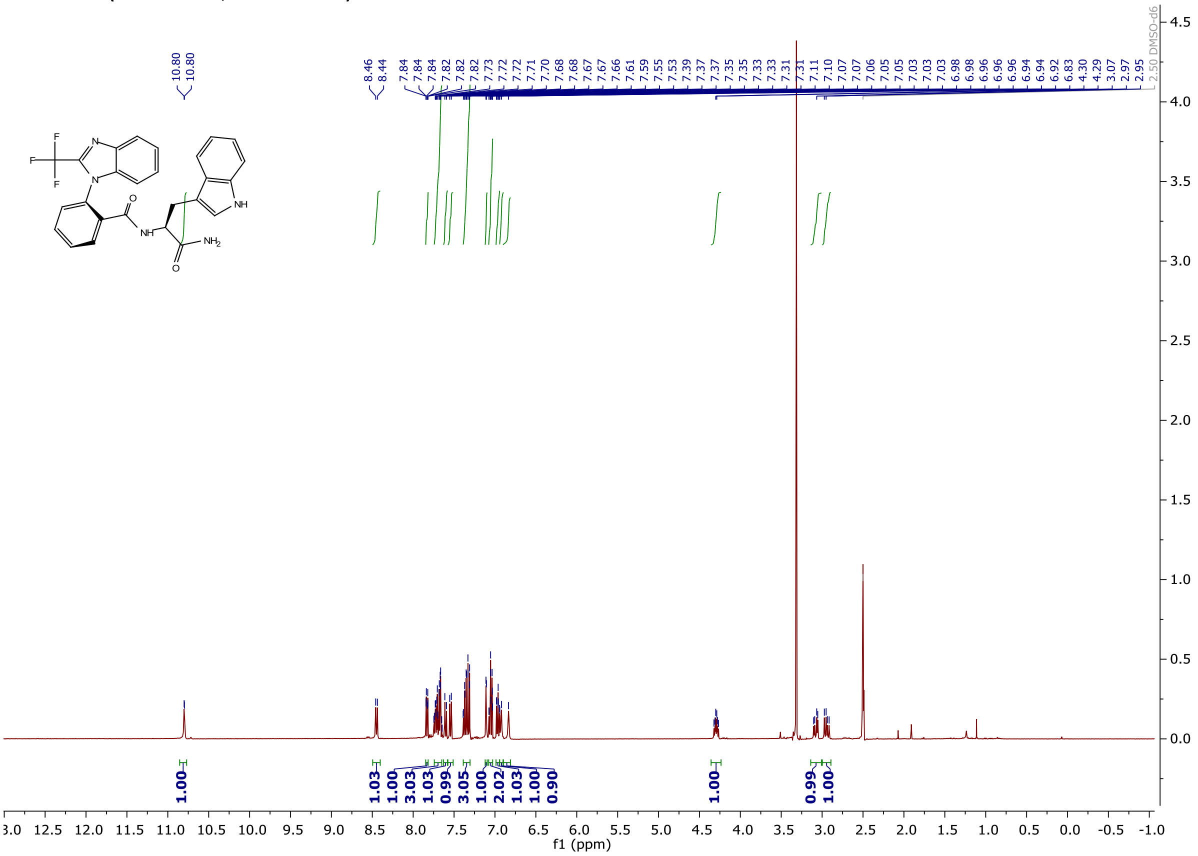

^{13}**C{^{1}H} NMR** (101 MHz, DMSO-d_6)

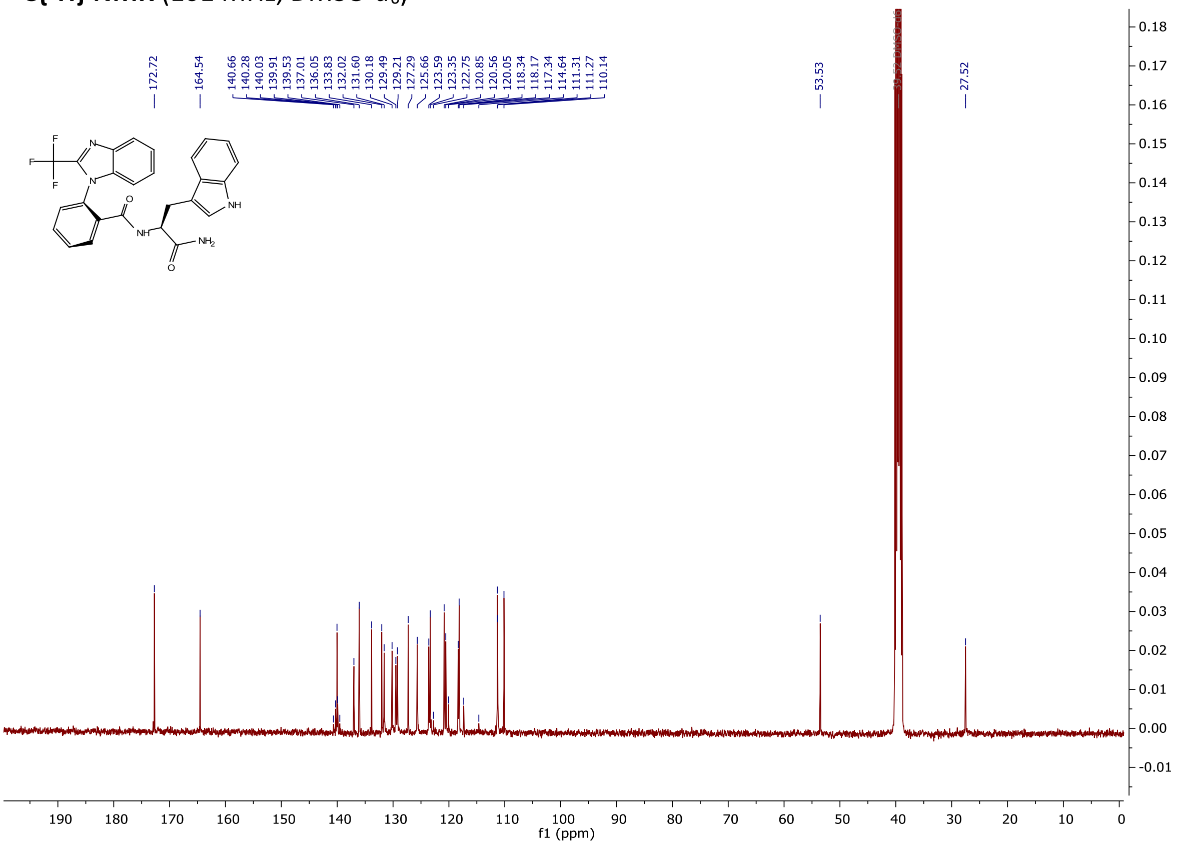

^{19}**F NMR** (376 MHz, DMSO-d_6)

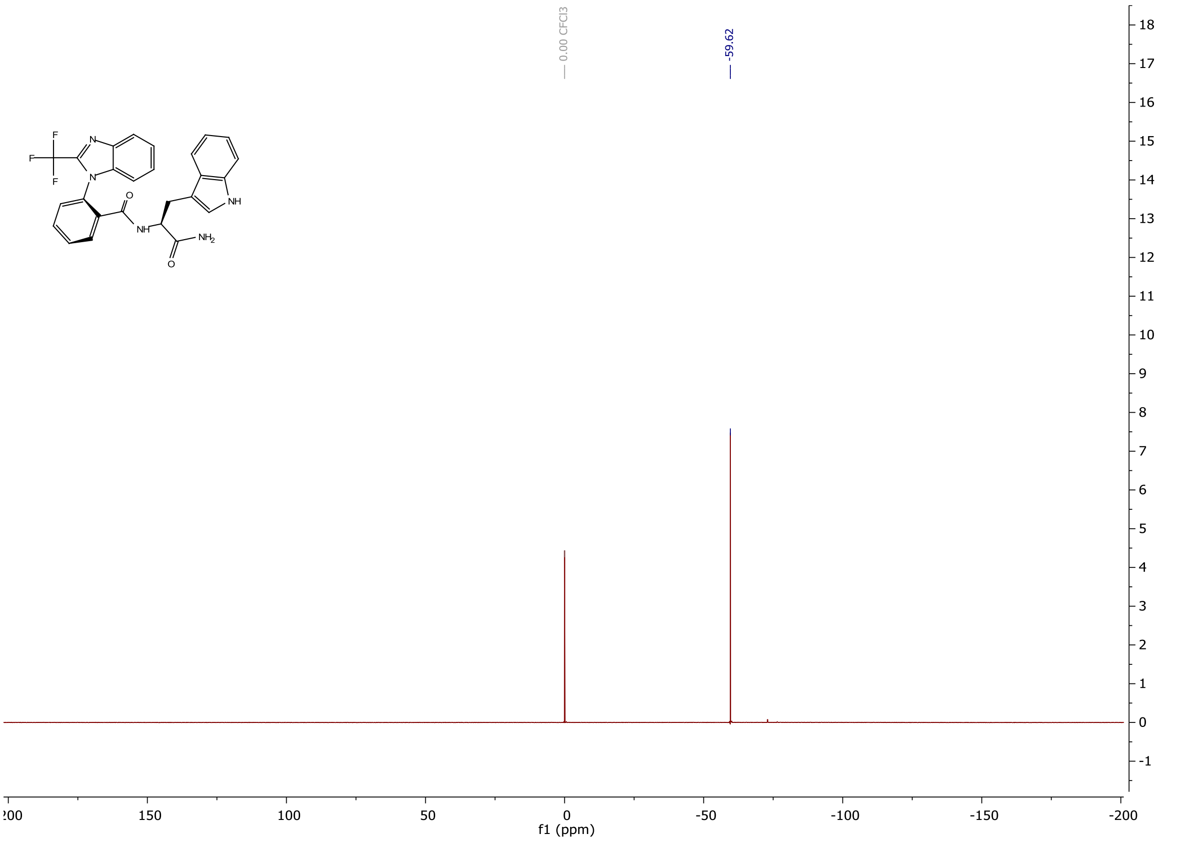

^{19}**F NMR** (76 MHz, THF)

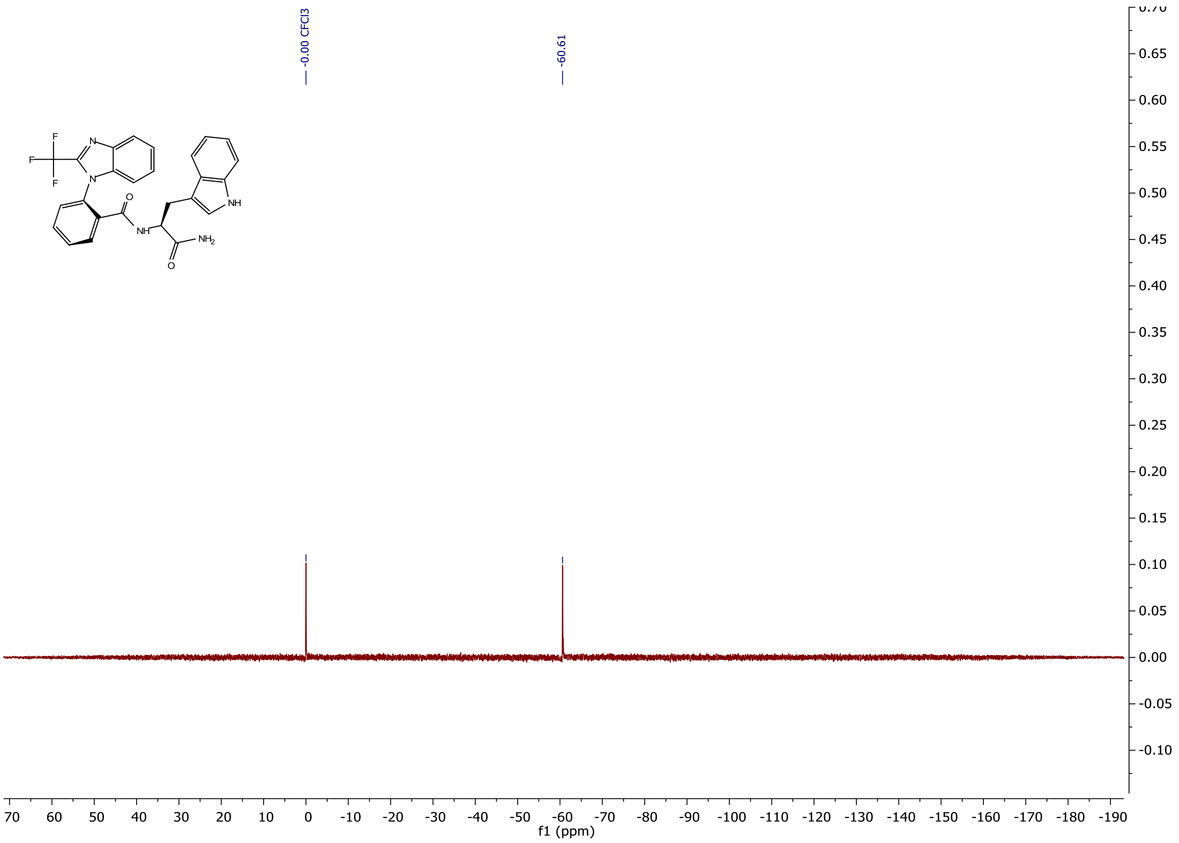

(*P*)-TBBA-(*L*)-His-NH_2 **(*P*)-16**

^{1}H NMR (400 MHz, DMSO-d_6)

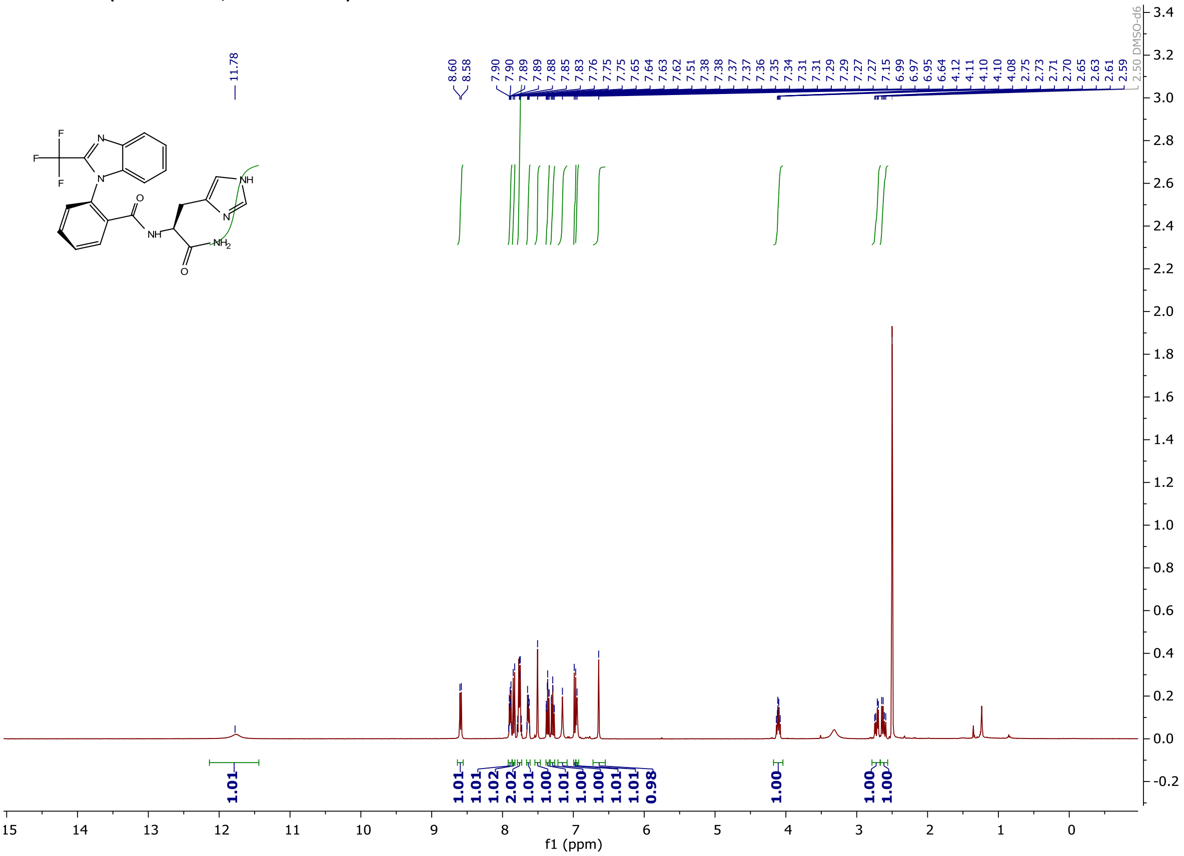

^{13}C{^{1}H} NMR (101 MHz, DMSO-d_6)

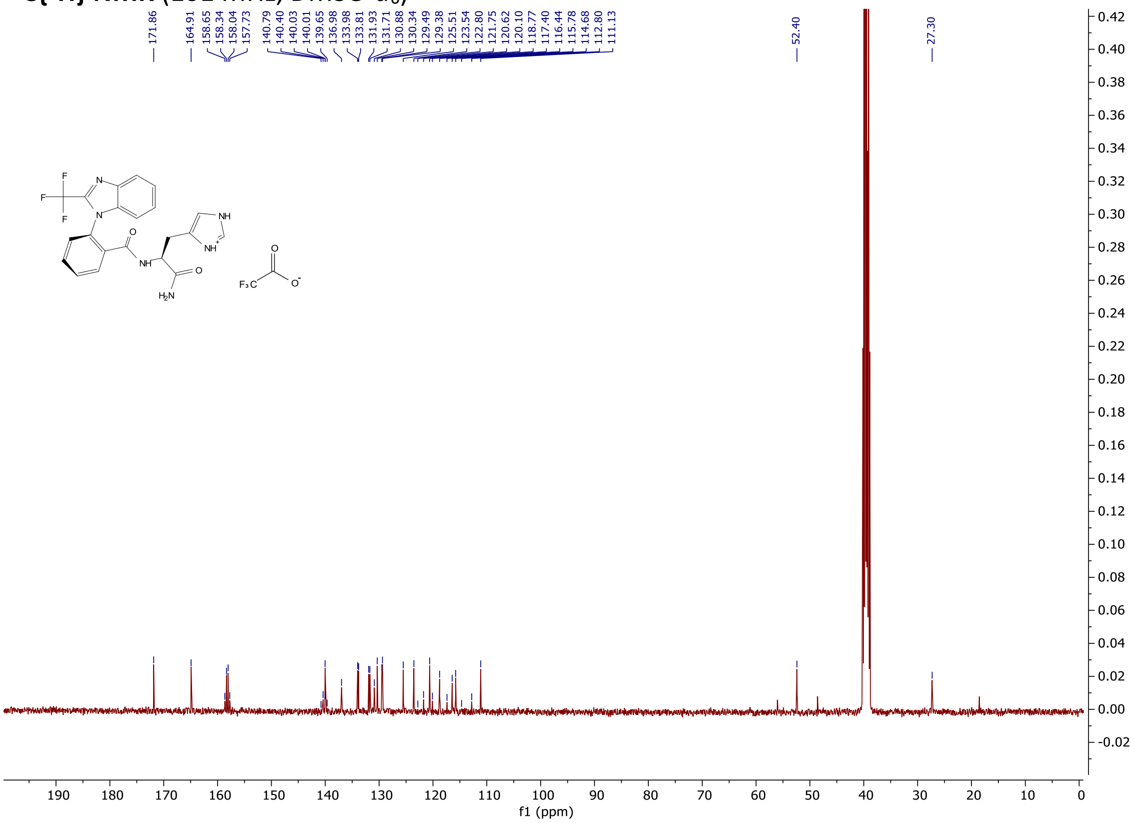

^{19}**F NMR** (376 MHz, DMSO-d_6)

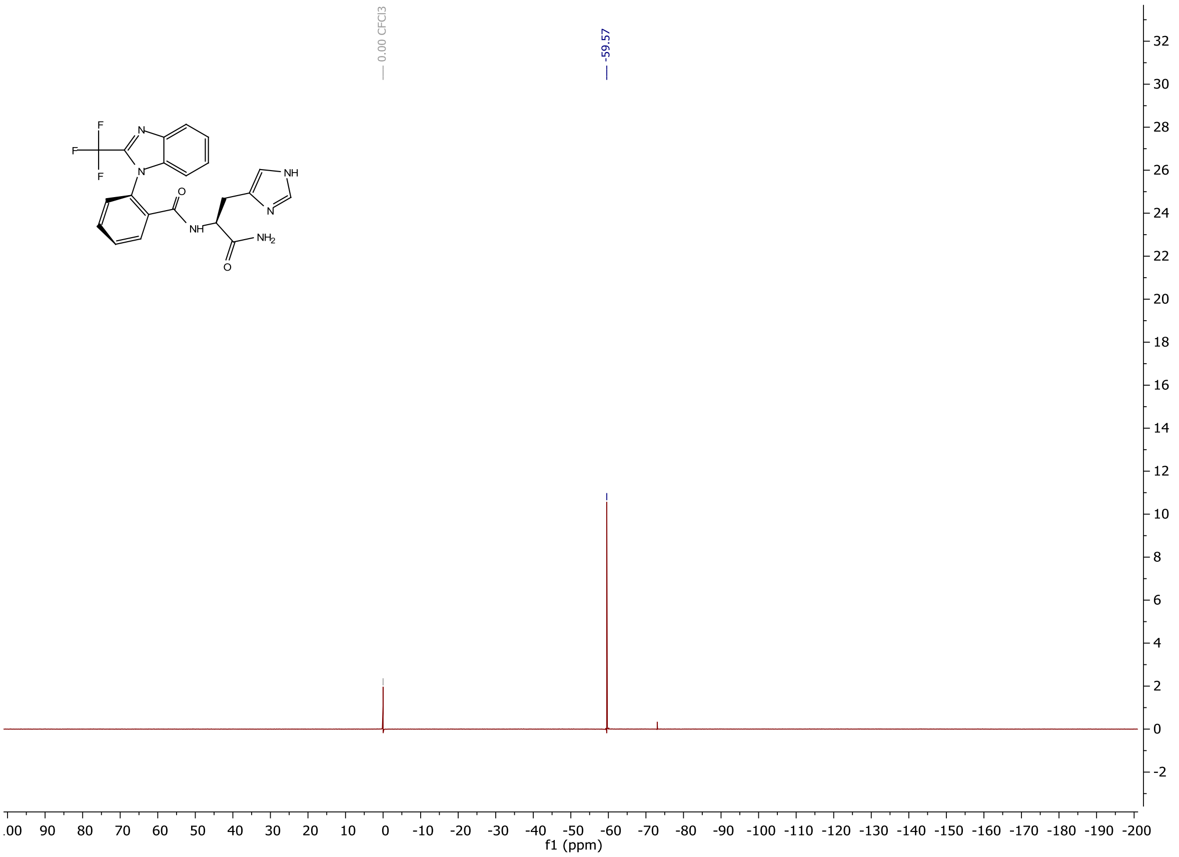

^{19}**F NMR** (76 MHz, THF)

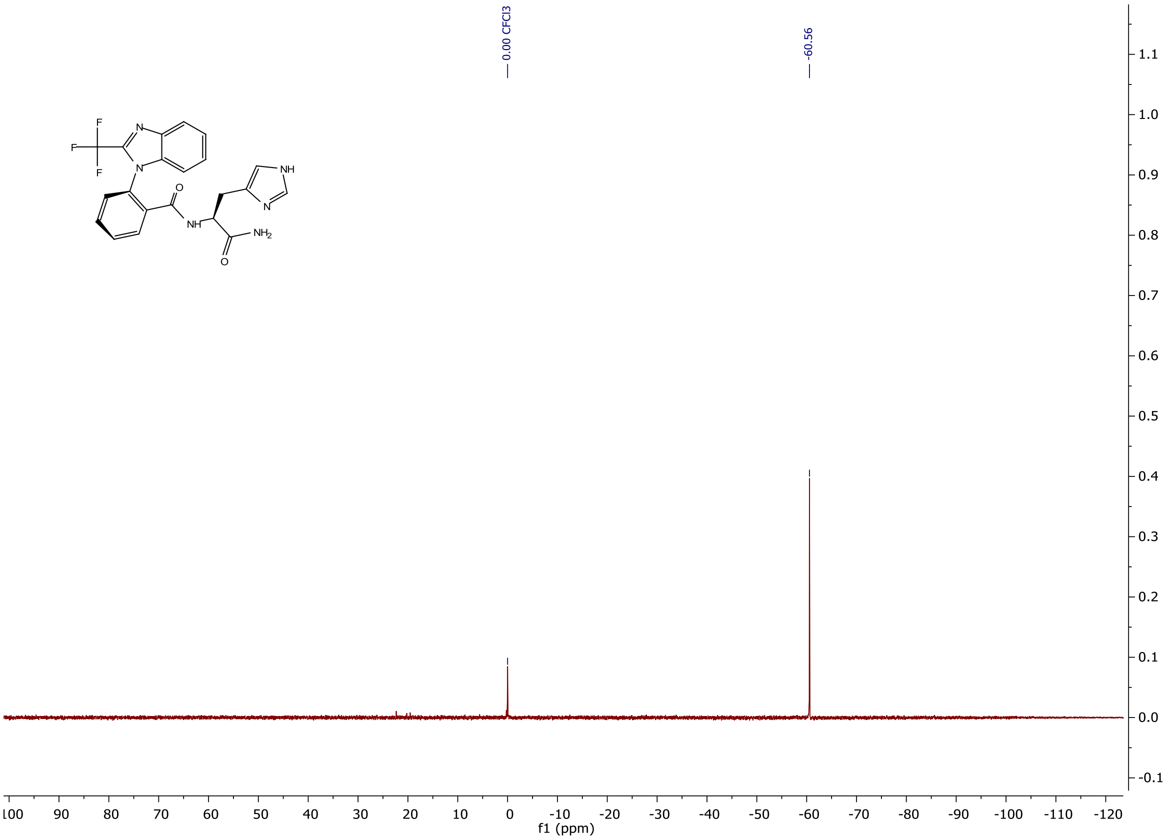

(*M*)-TBBA-(*L*)-His-NH_2 **(*M*)-16**

^{1}H NMR (400 MHz, DMSO-d_6)

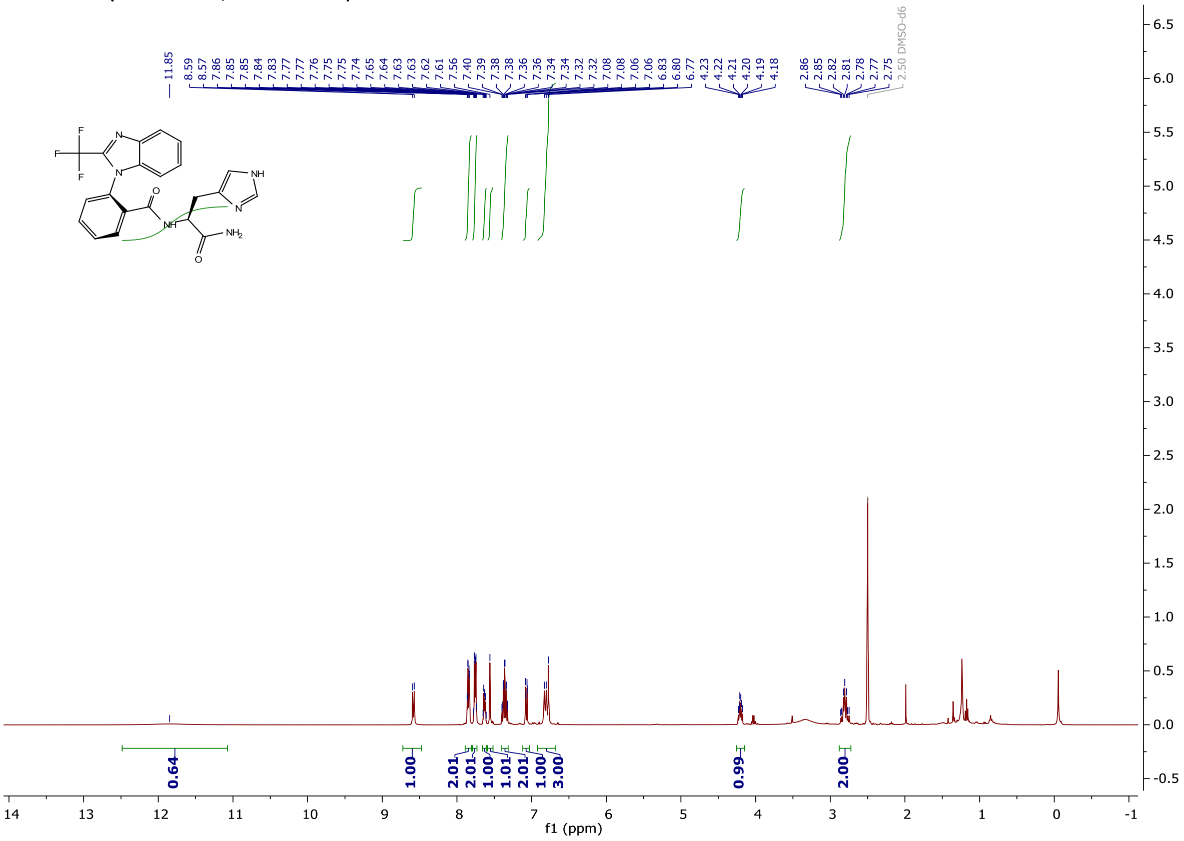

^{13}C{^{1}H} NMR (101 MHz, DMSO-d_6)

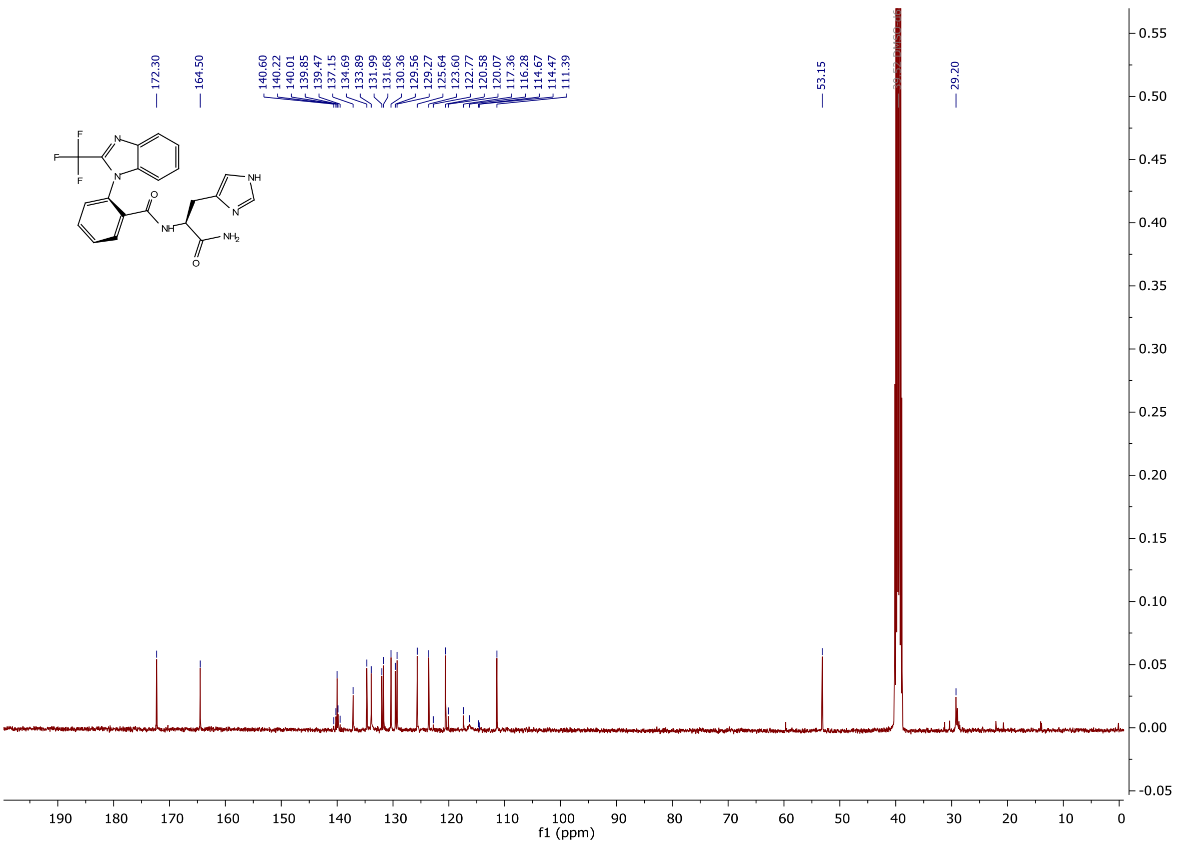

^{19}F NMR (376 MHz, DMSO-d_6)

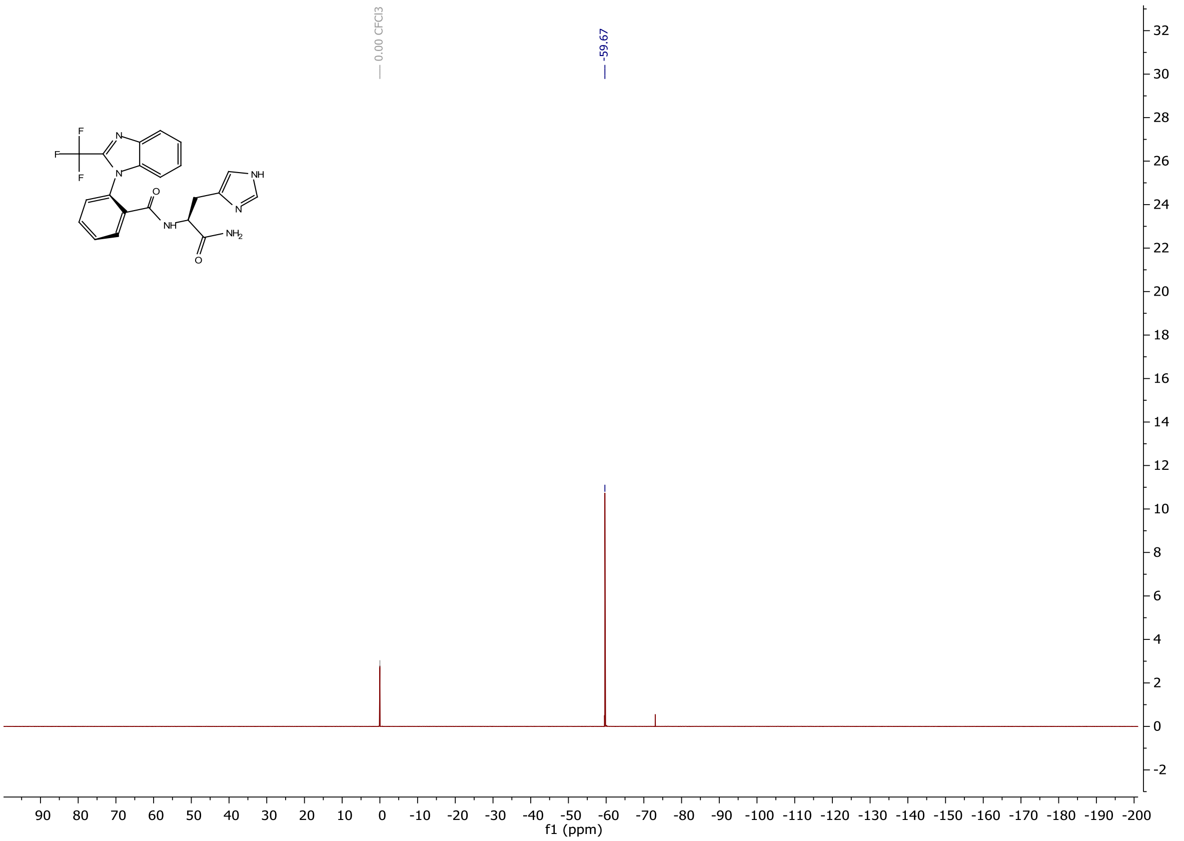

^{19}F NMR (76 MHz, THF)

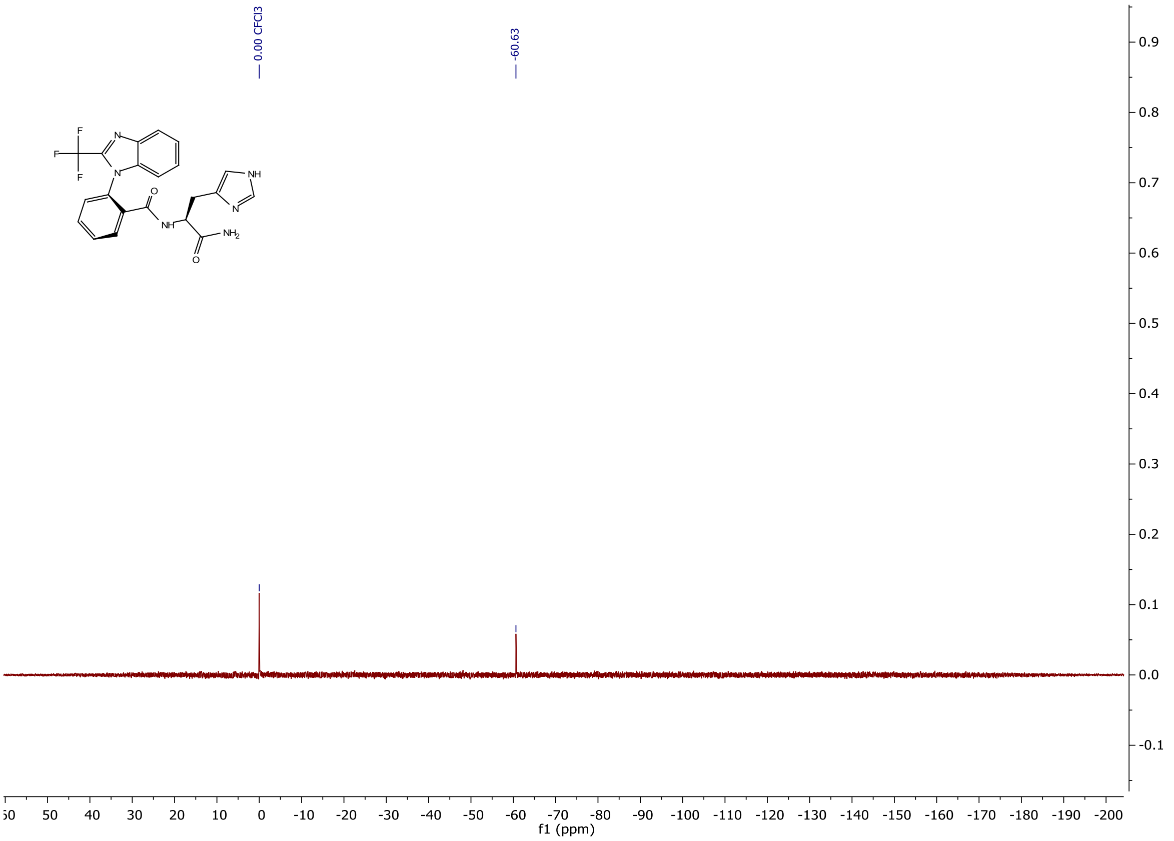

(*P*)-TBBA-(*L*)-Ser-NH_2 **(*P*)-17**

^{1}H NMR (400 MHz, DMSO-d_6)

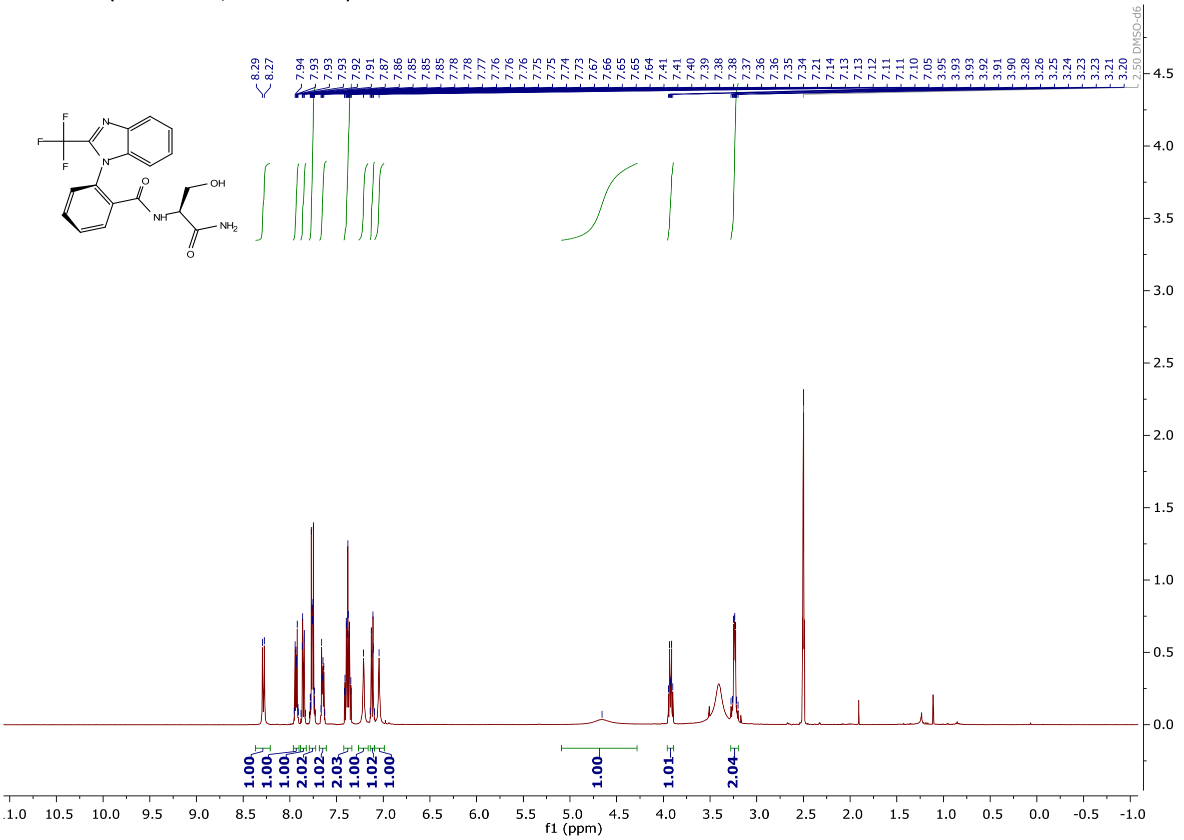

$^{13}C\{^{1}H\}$ NMR (101 MHz, DMSO-d_6)

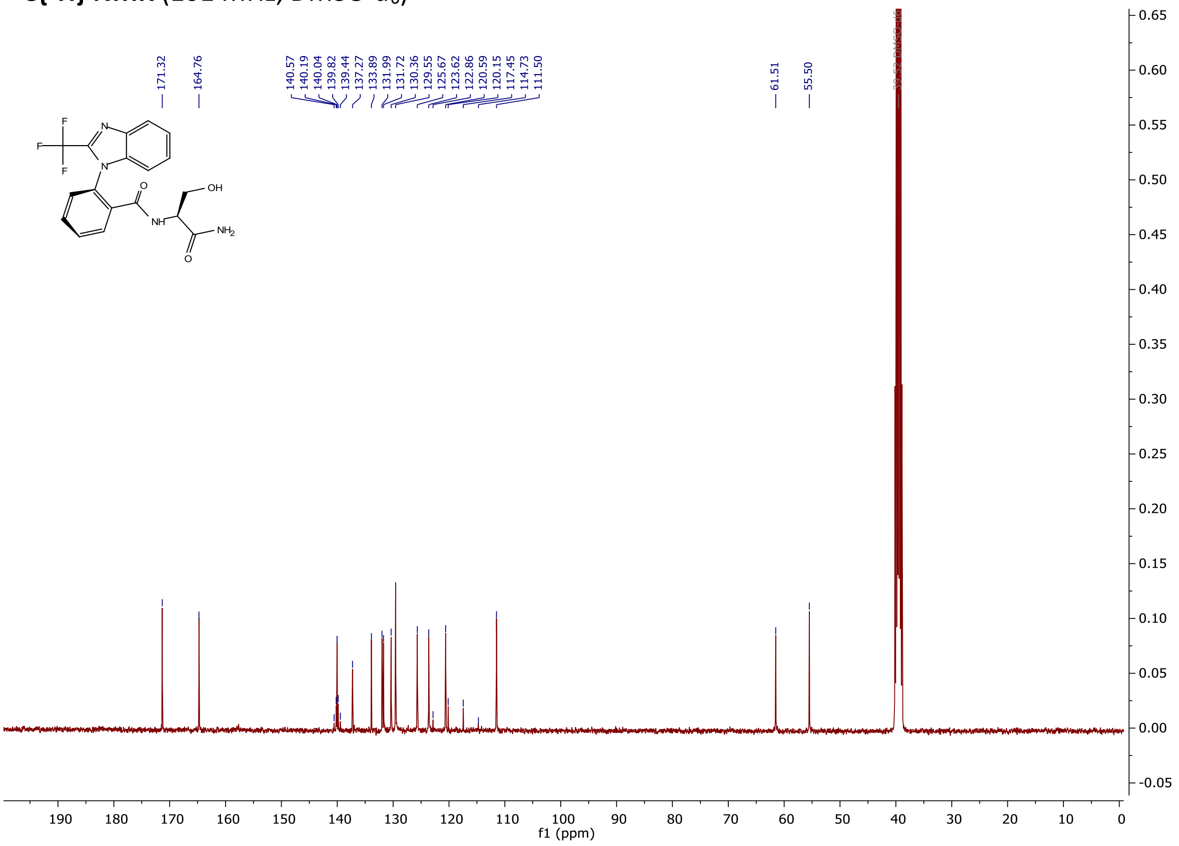

^{19}F NMR (376 MHz, DMSO-d_6)

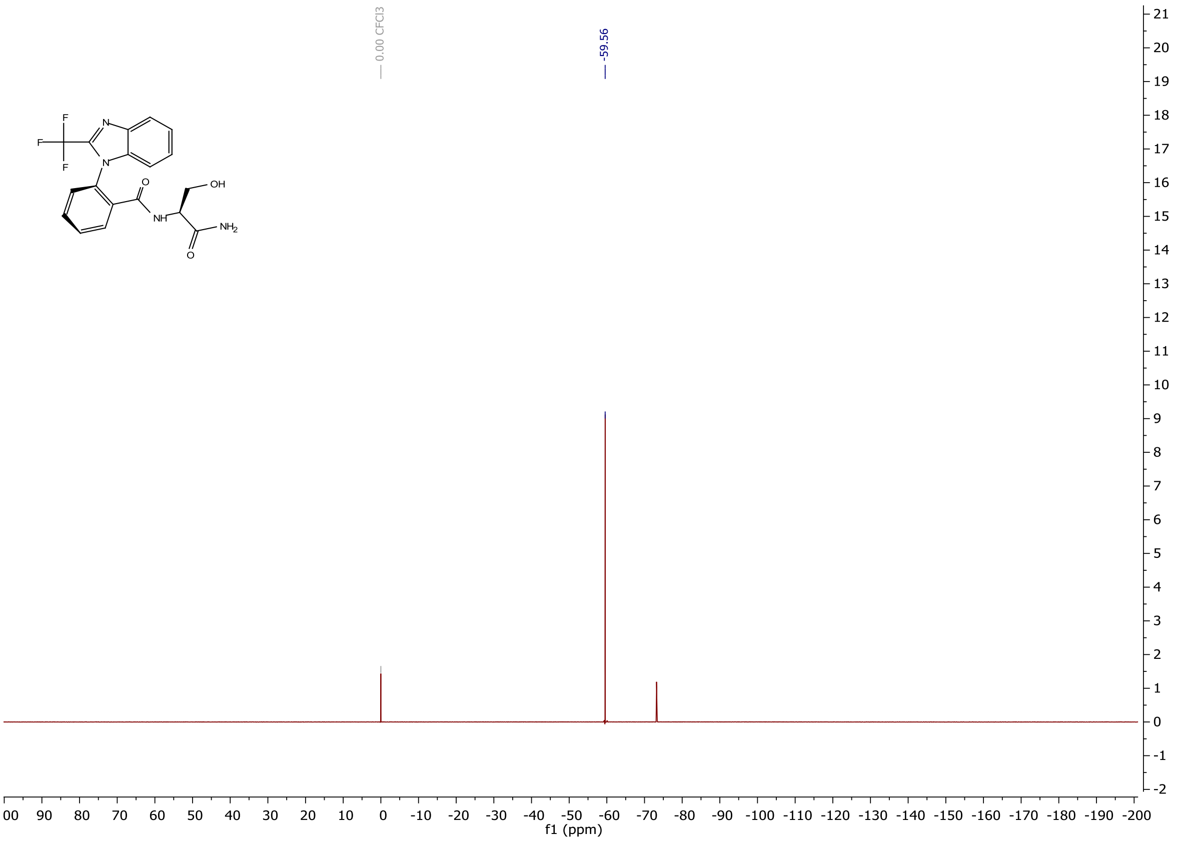

^{19}F NMR (76 MHz, THF)

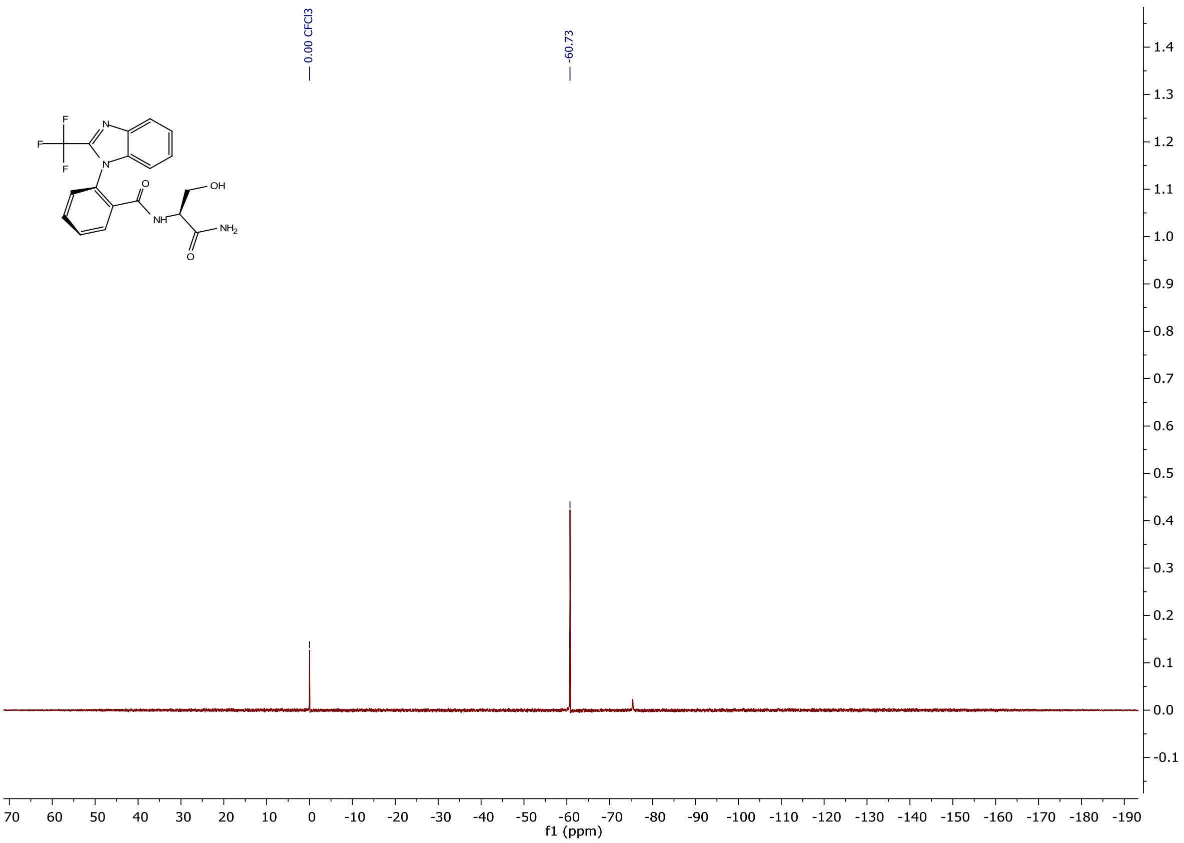

(*M*)-TBBA-(*L*)-Ser-NH2 **(*M*)-17**

^{1}H NMR (400 MHz, DMSO-*d*6)

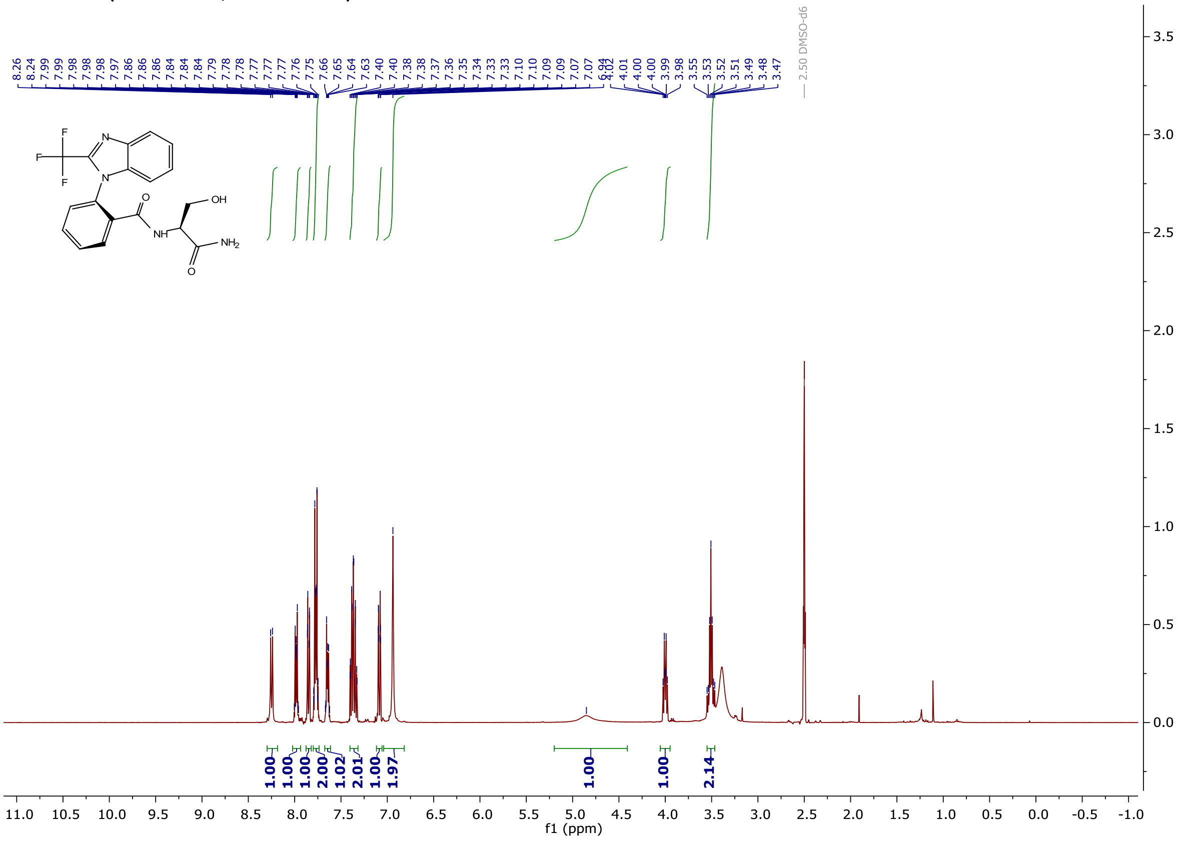

^{13}C{^{1}H} NMR (101 MHz, DMSO-*d*6)

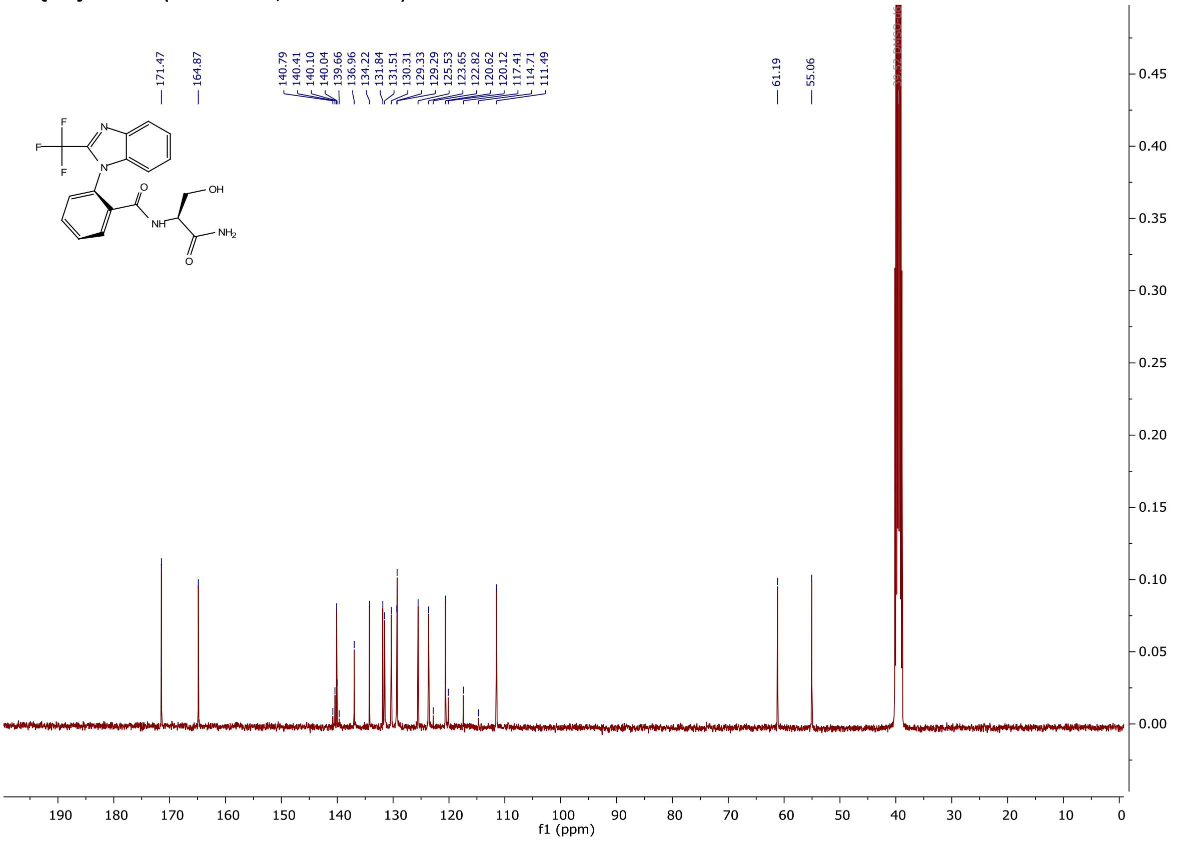

^{19}**F NMR** (376 MHz, DMSO-d_6)

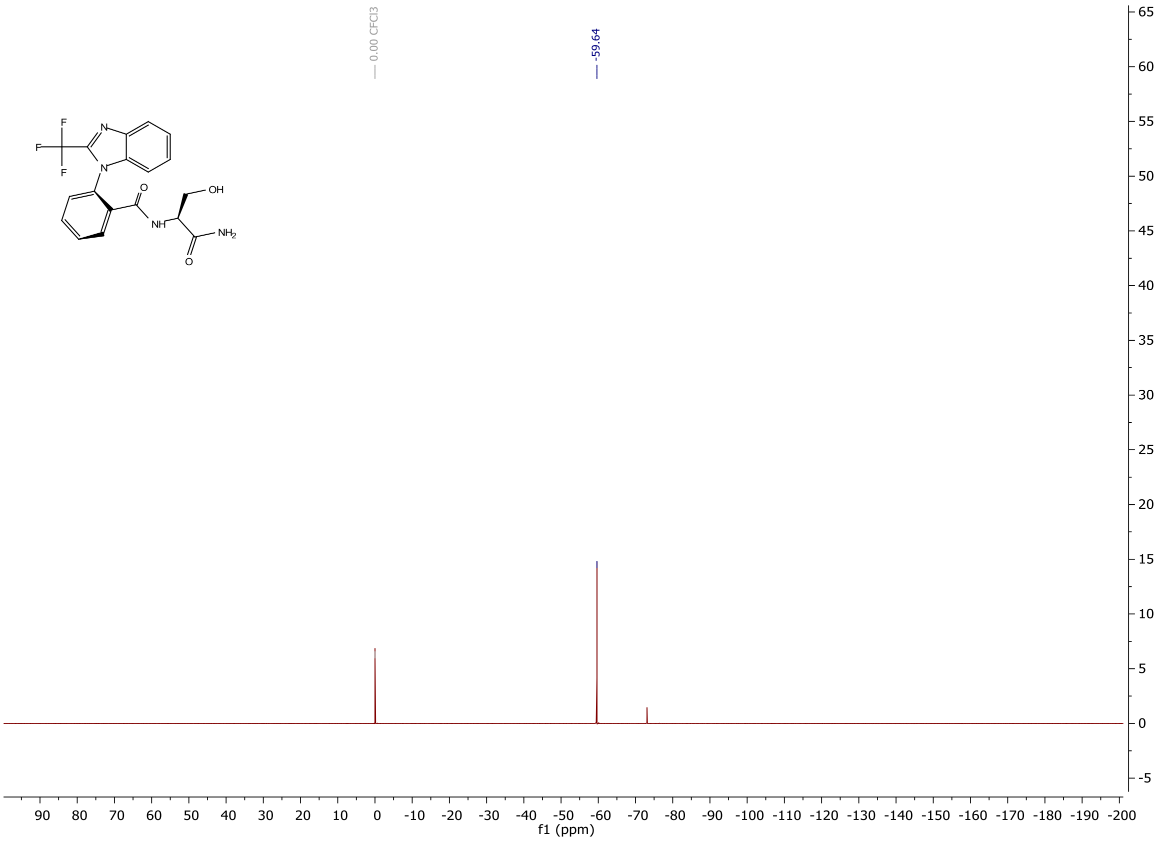

^{19}**F NMR** (76 MHz, THF)

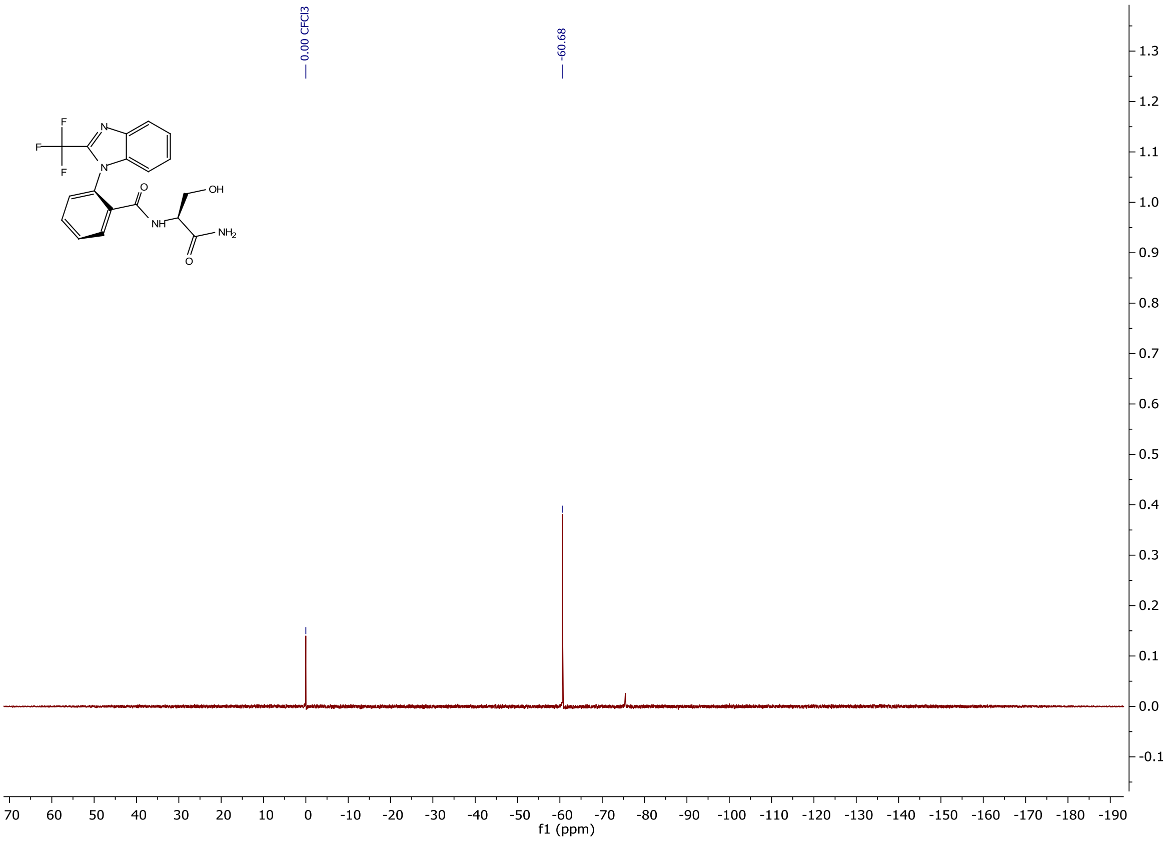

(*P*)-TBBA-(*L*)-Asp(OMe)-NH_2 **(*P*)-18**

^{1}H NMR (400 MHz, DMSO-d_6)

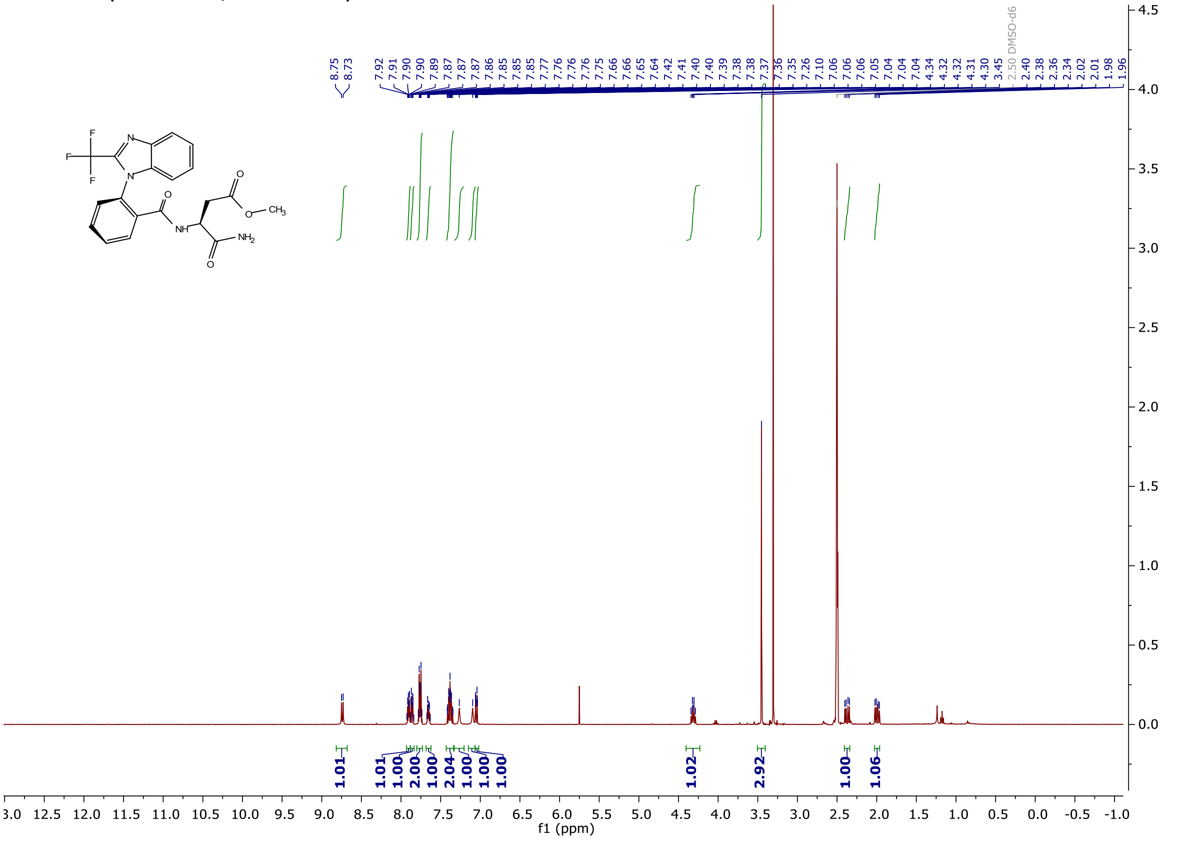

^{13}C{^{1}H} NMR (101 MHz, DMSO-d_6)

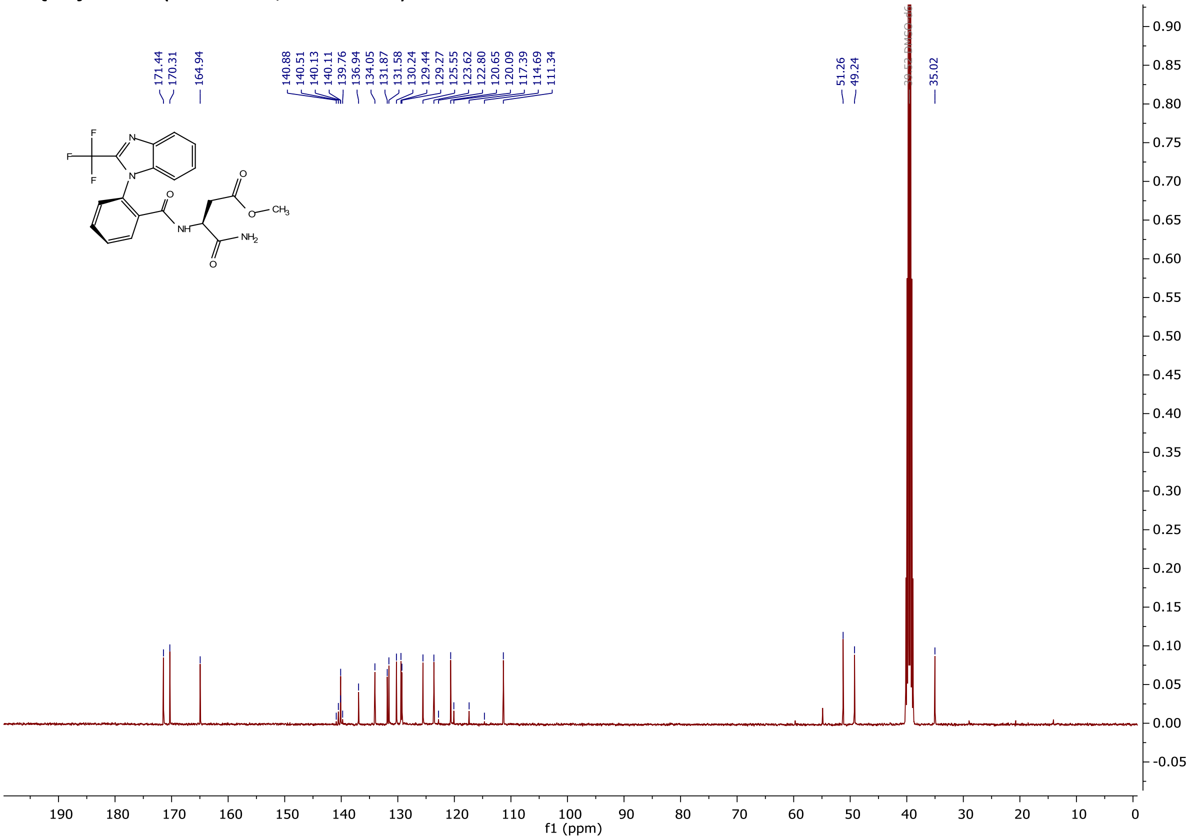

^{19}F NMR (376 MHz, DMSO-d_6)

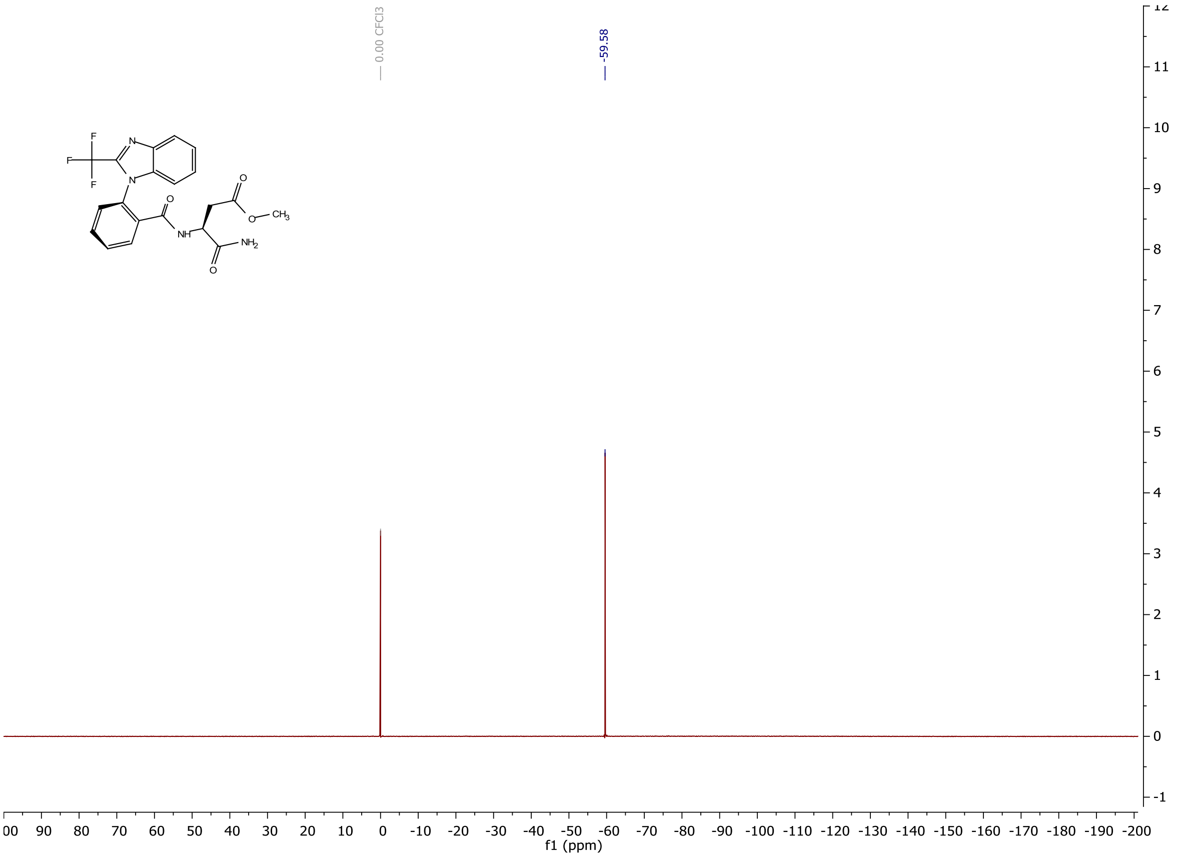

^{19}F NMR (76 MHz, THF)

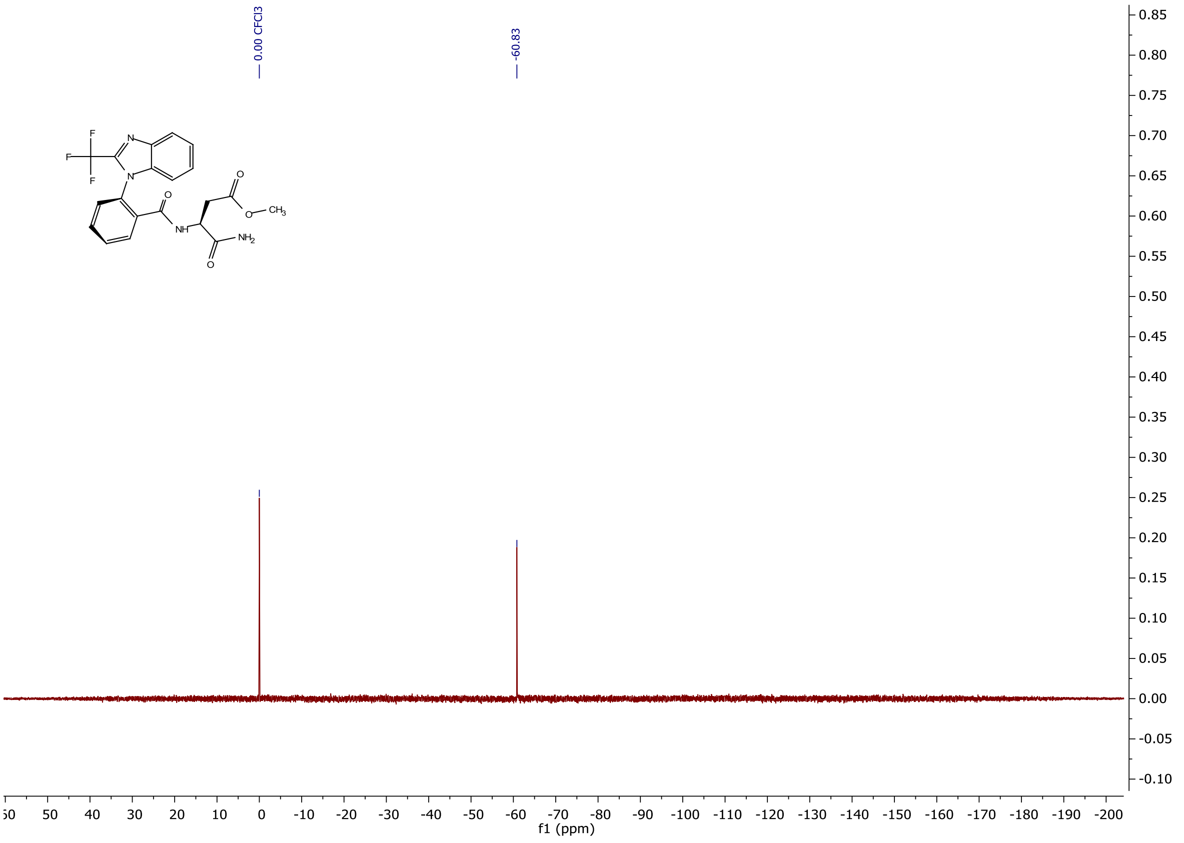

(*M*)-TBBA-(*L*)-Asp(OMe)-NH_2 **(*M*)-18**

^{1}H NMR (400 MHz, DMSO-d_6)

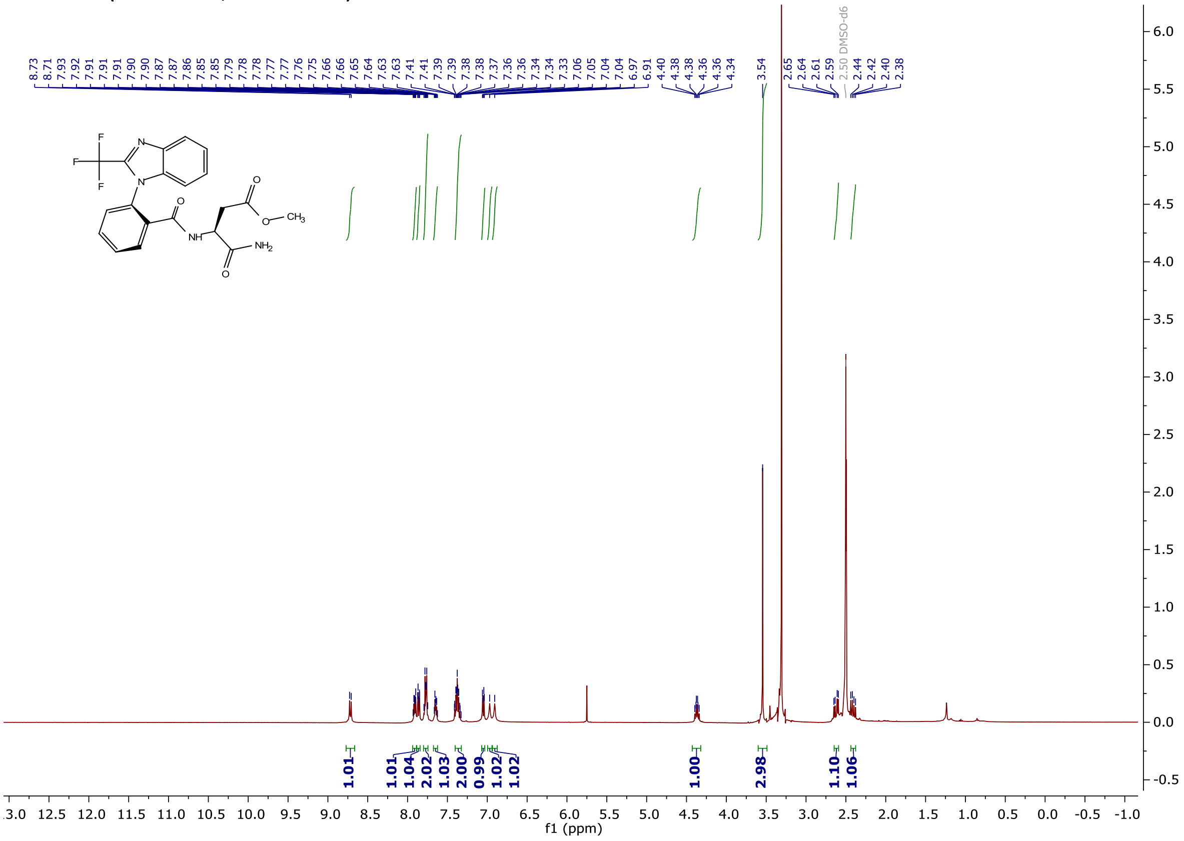

^{13}C{^{1}H} NMR (101 MHz, DMSO-d_6)

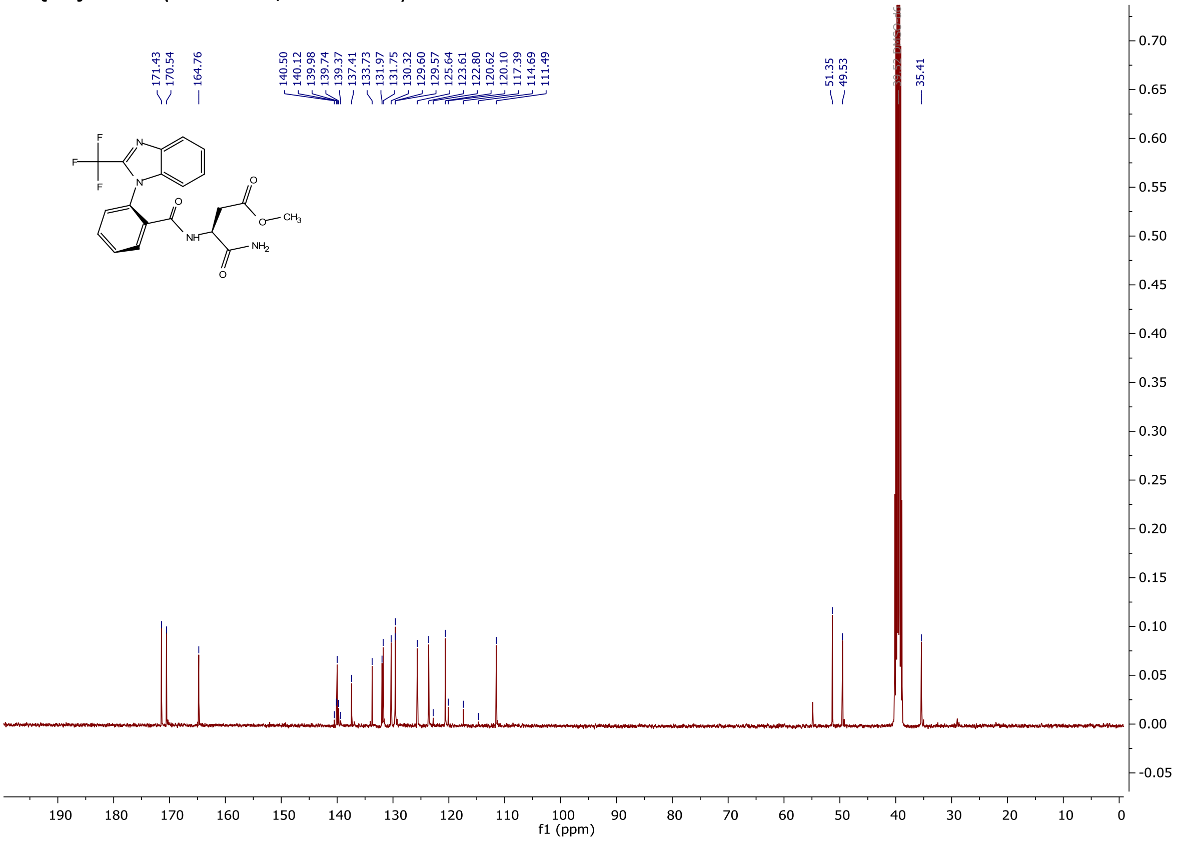

^{19}**F NMR** (376 MHz, DMSO-d_6)

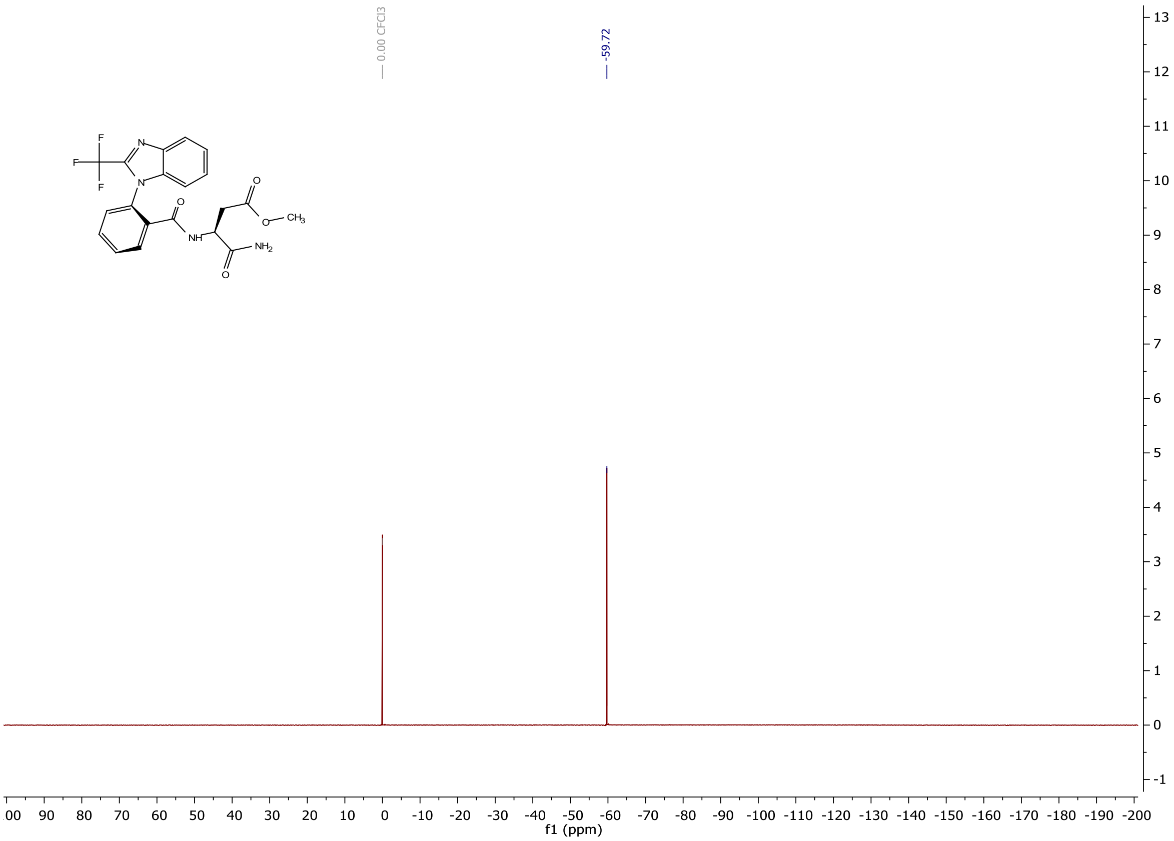

^{19}**F NMR** (76 MHz, THF)

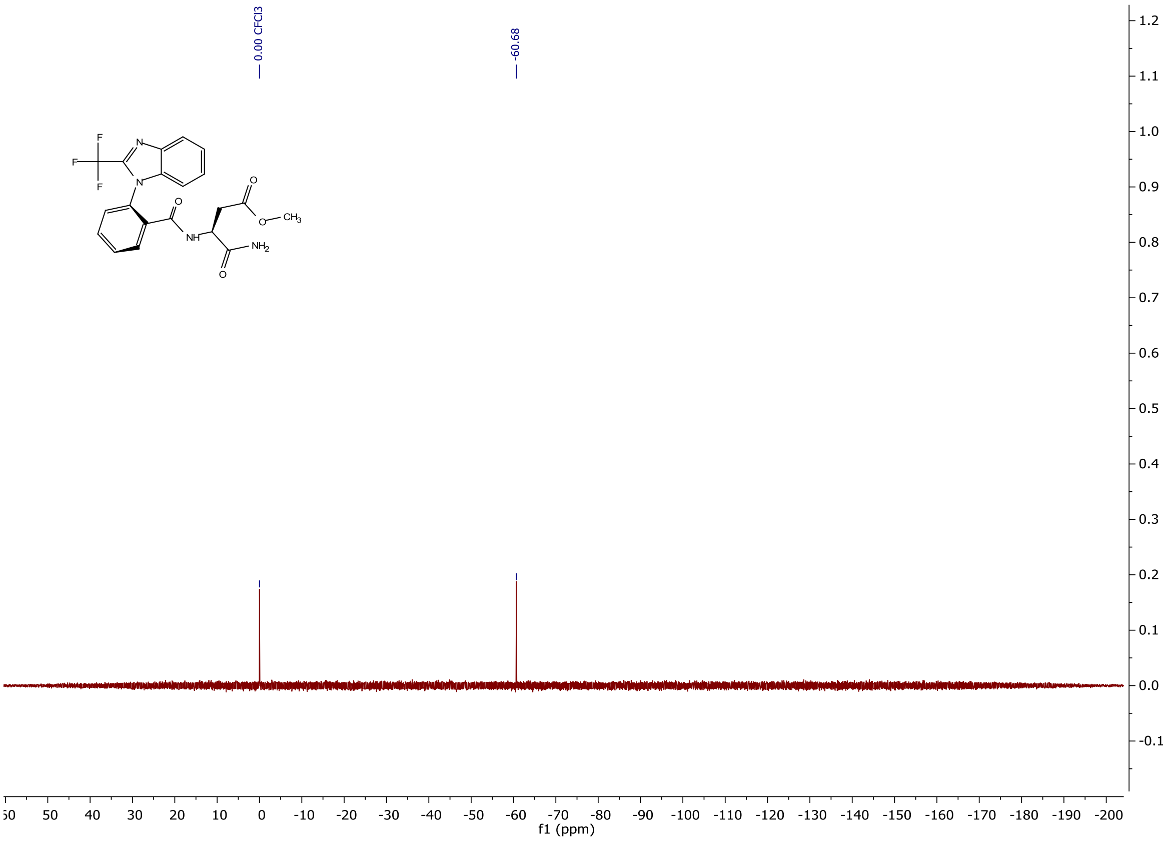

(*P*)-TBBA-(*L*)-Asn-NH₂ **(*P*)-19**

^{1}H NMR (400 MHz, DMSO-d_6)

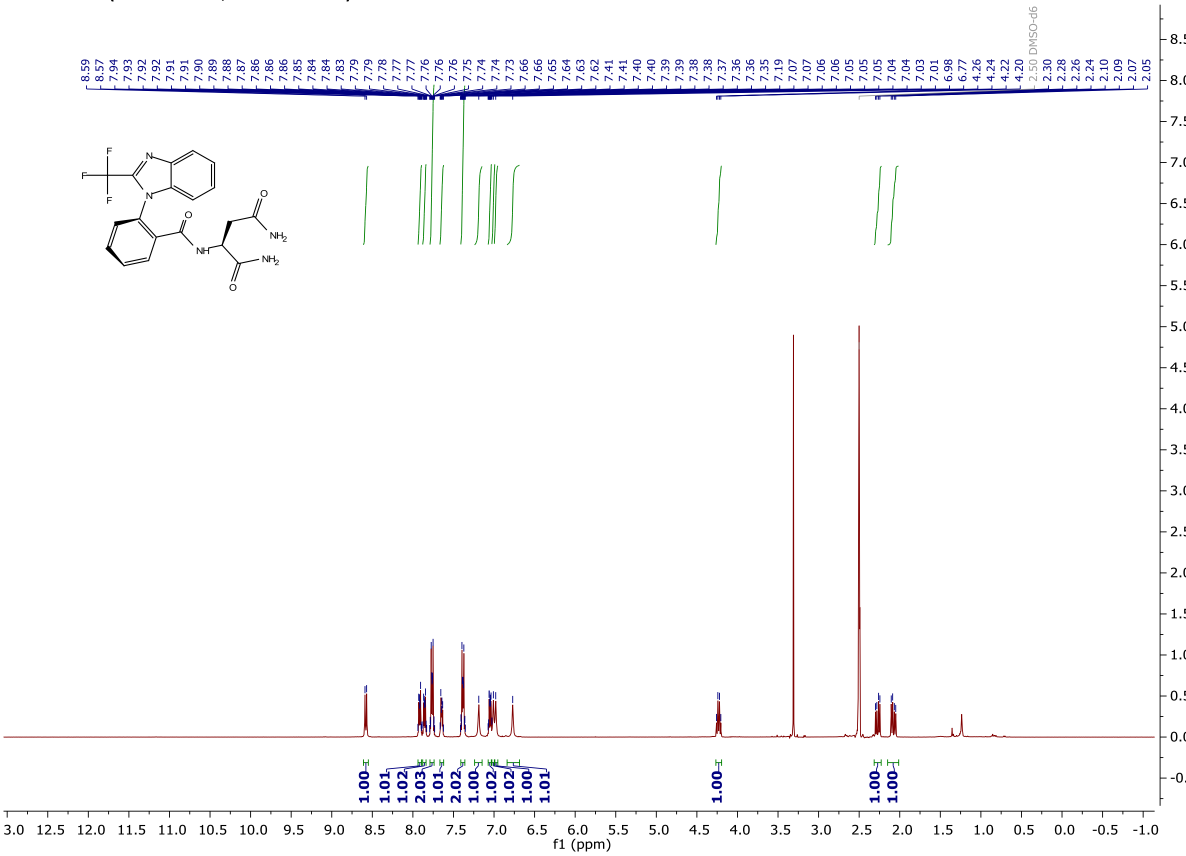

^{13}C{^{1}H} NMR (101 MHz, DMSO-d_6)

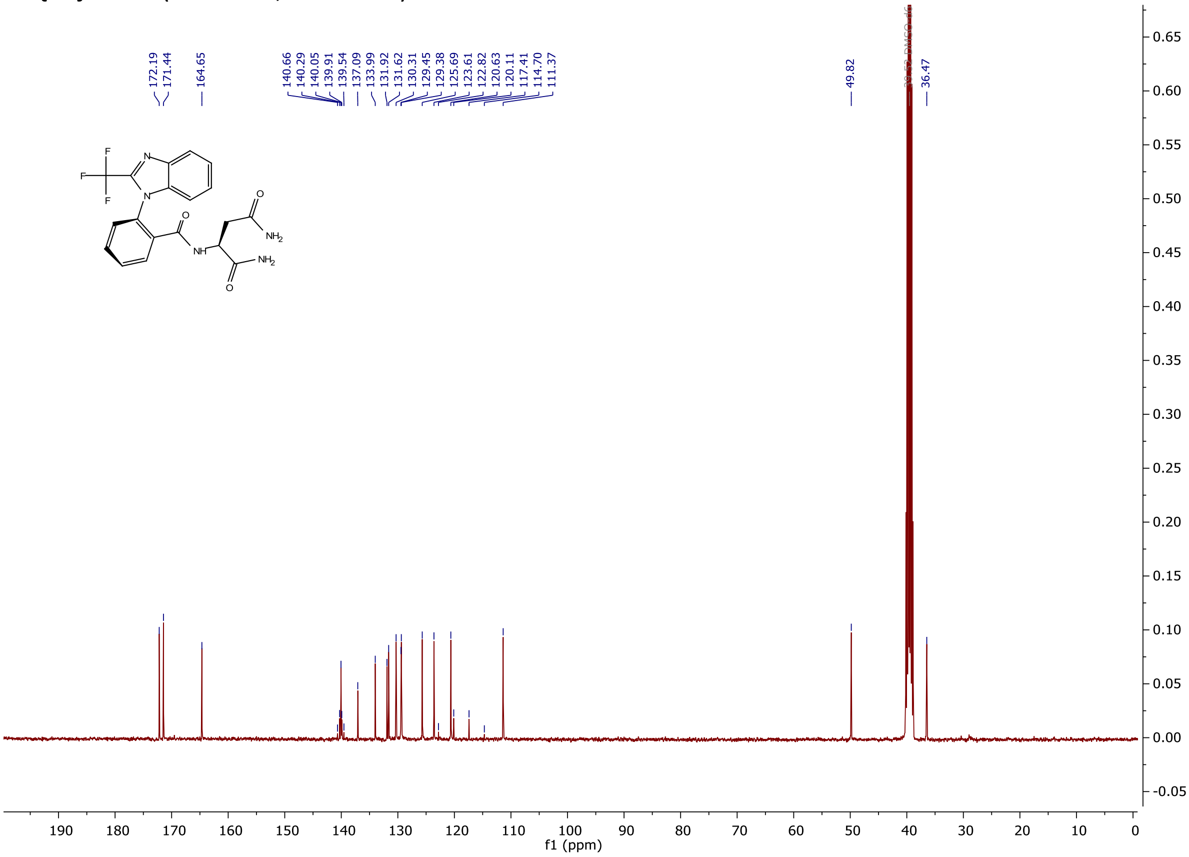

^{19}**F NMR** (376 MHz, DMSO-d_6)

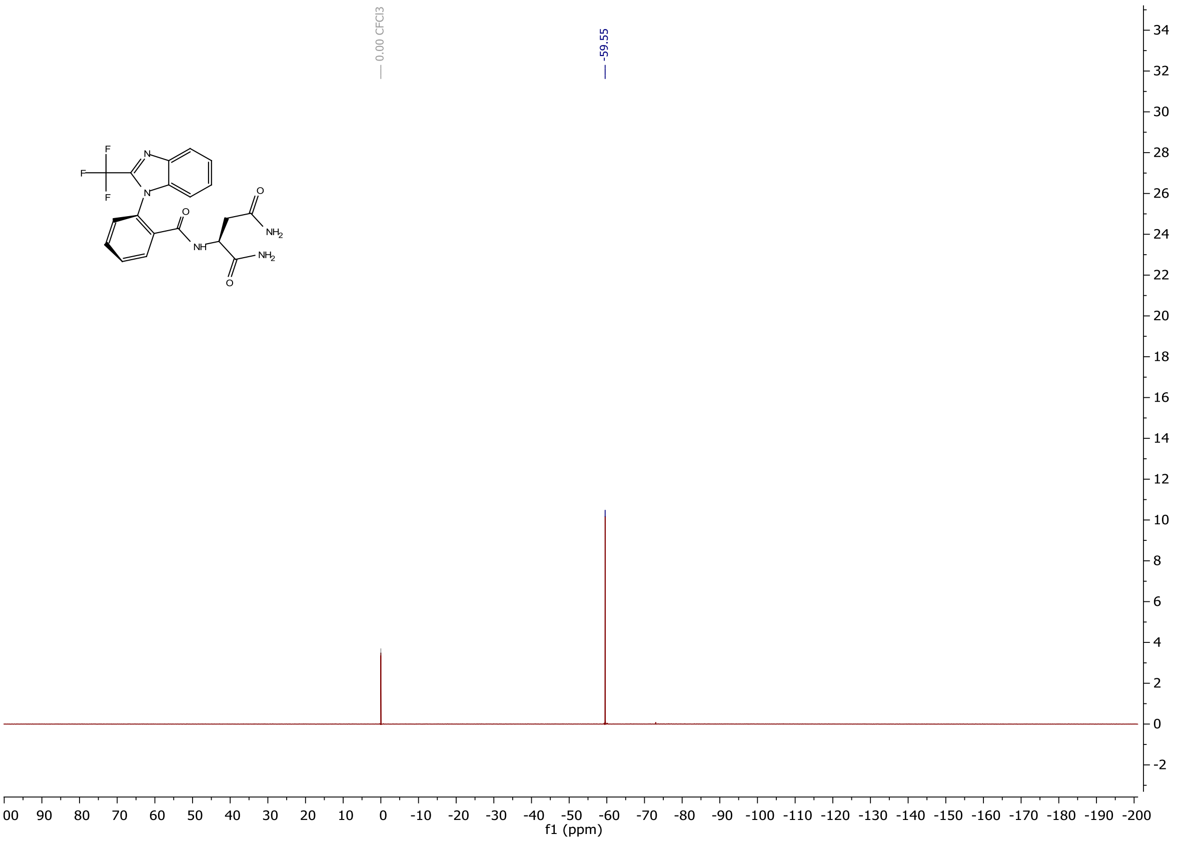

^{19}**F NMR** (76 MHz, THF)

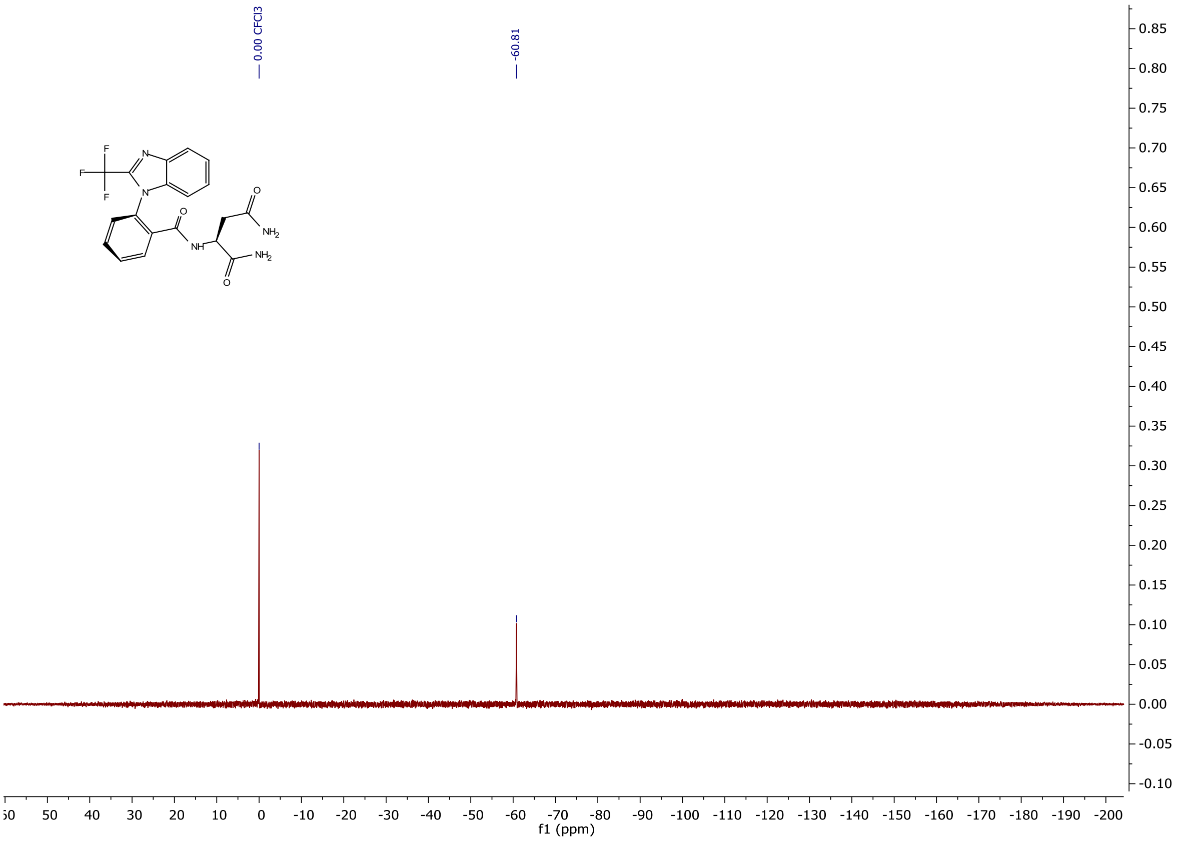

(*M*)-TBBA-(*L*)-Asn-NH_2 **(*M*)-19**

^{1}H NMR (400 MHz, DMSO-d_6)

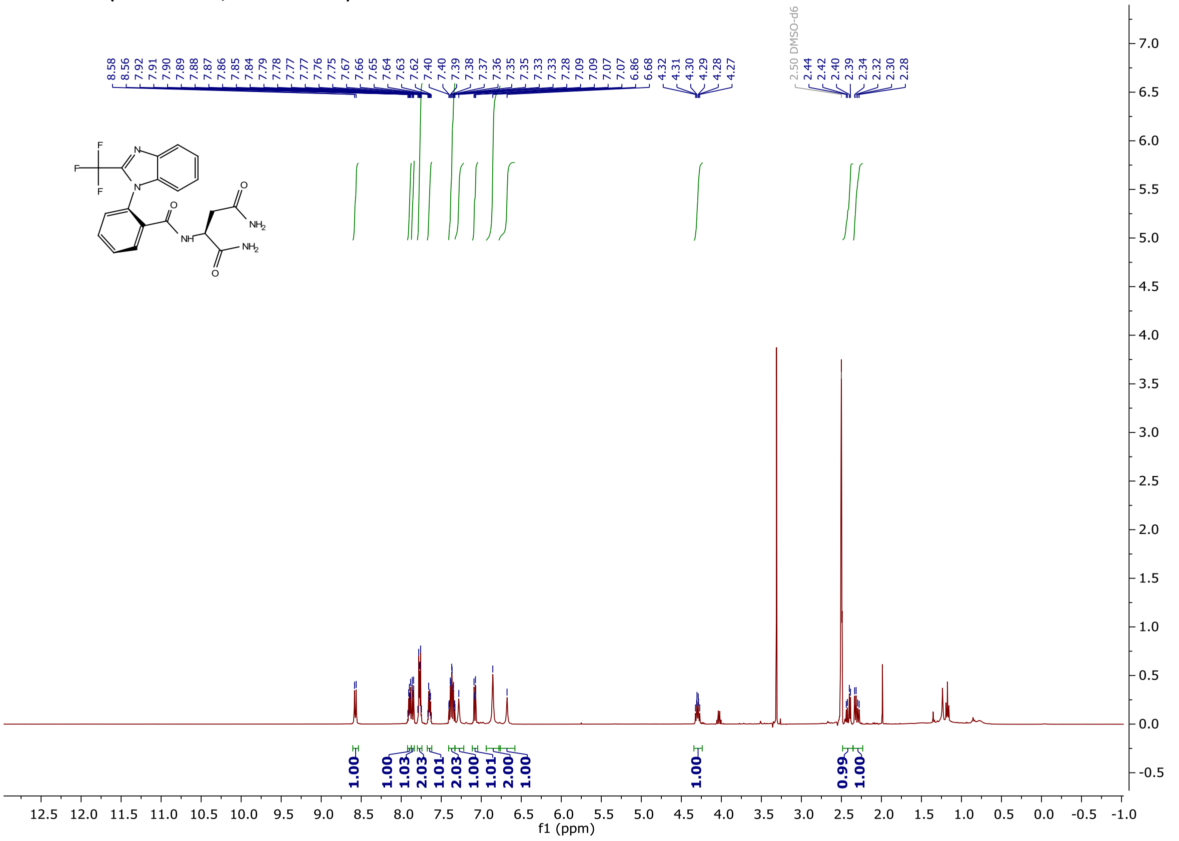

$^{13}C\{^{1}H\}$ NMR (101 MHz, DMSO-d_6)

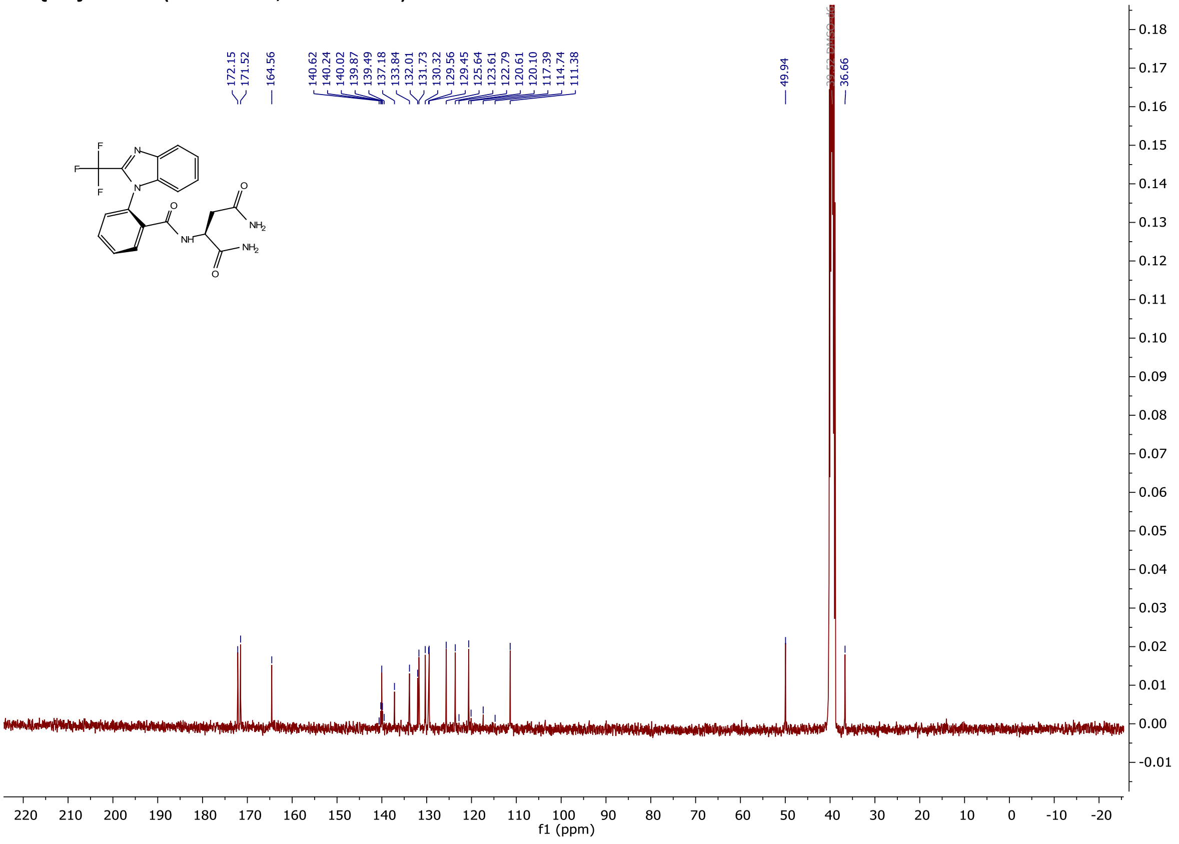

^{19}**F NMR** (376 MHz, DMSO-d_6)

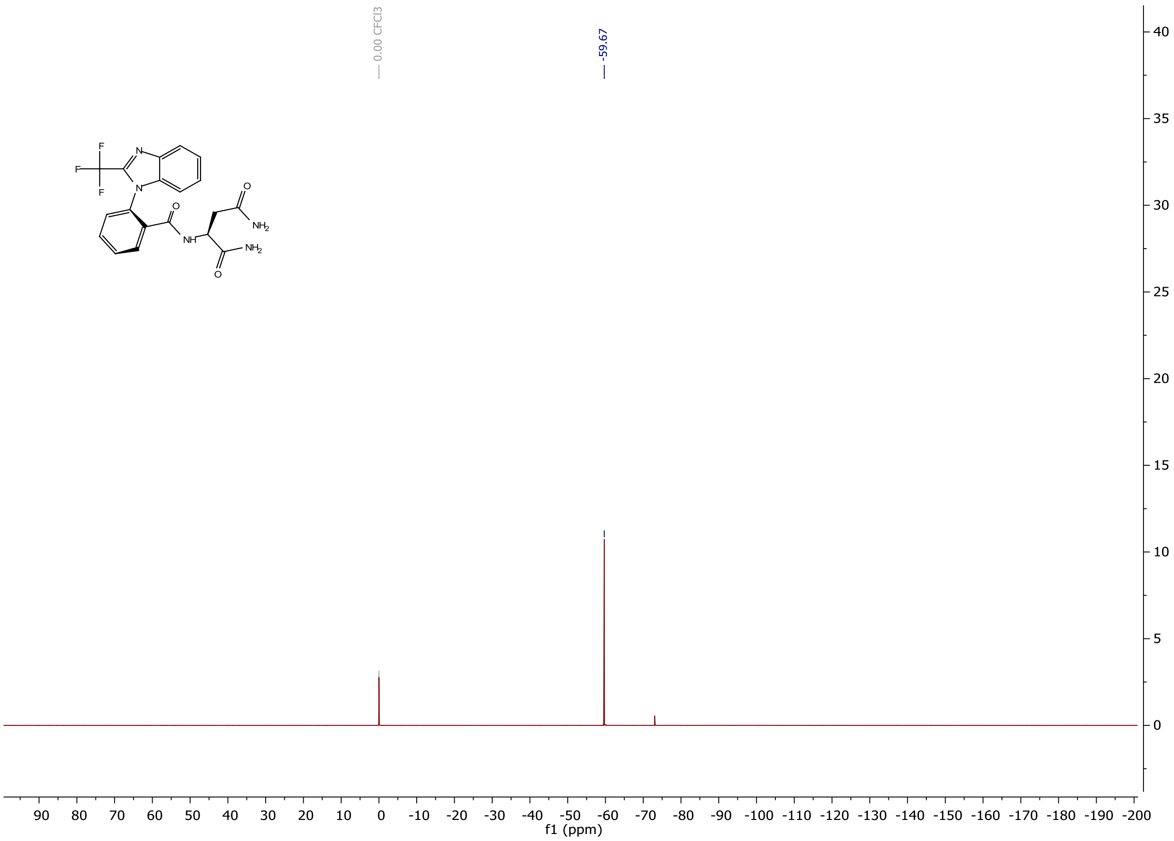

^{19}**F NMR** (76 MHz, THF)

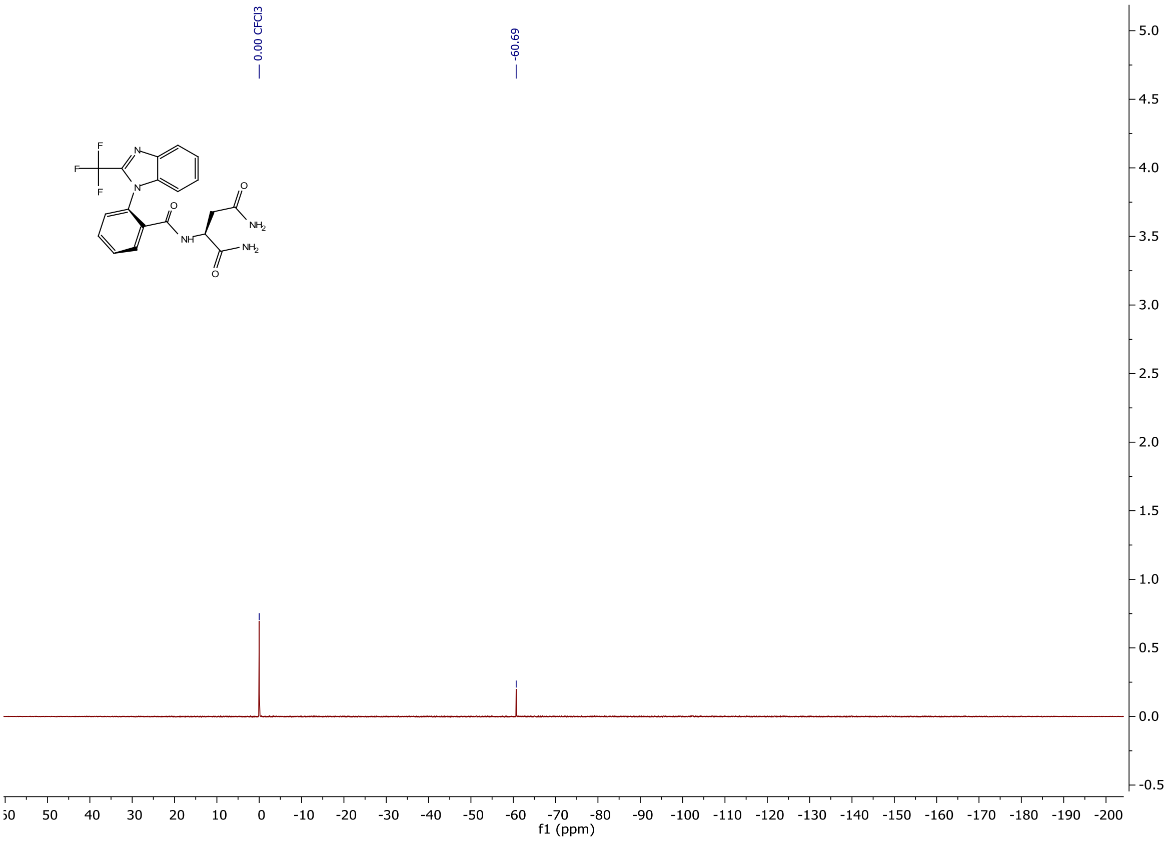

(*P*)-TBBA-(L)-Ala-(L)-Ala-NH_2 **(*P*)-20**

^{1}H NMR (400 MHz, DMSO-d_6)

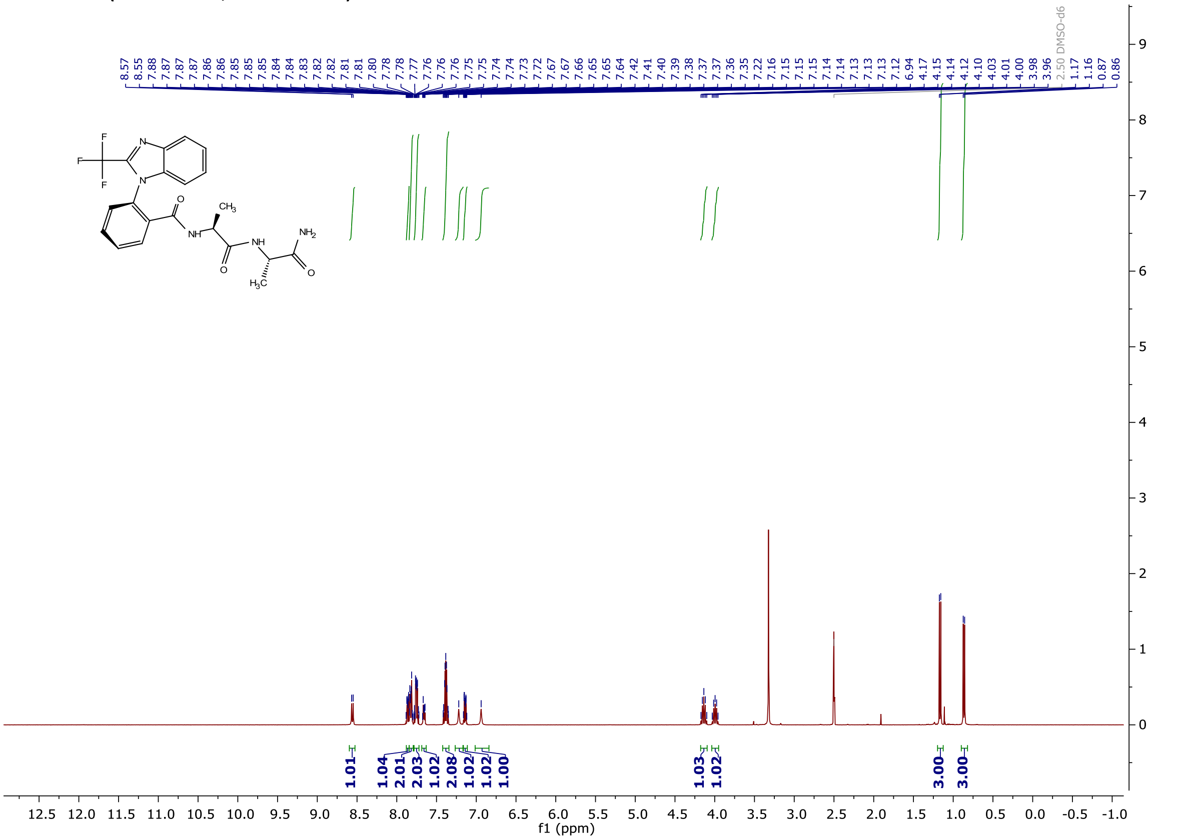

^{13}C{^{1}H} NMR (101 MHz, DMSO-d_6)

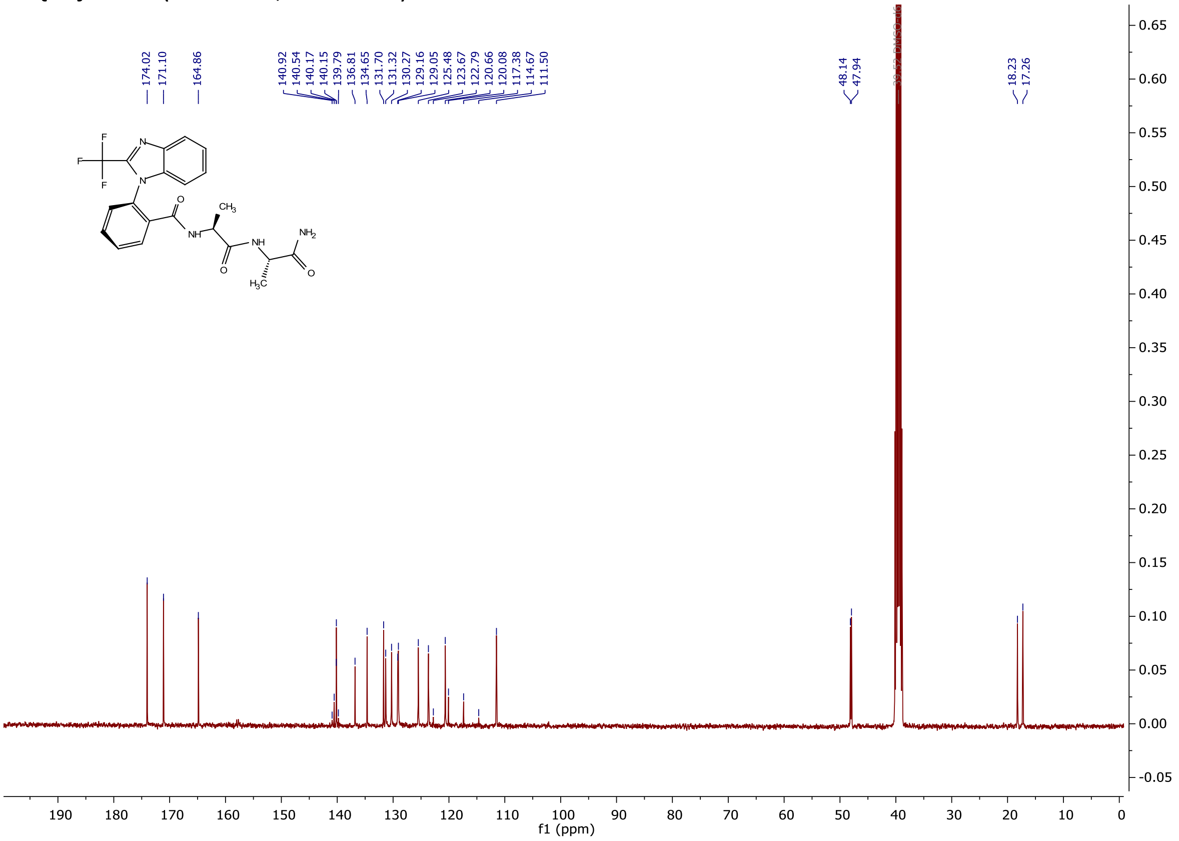

^{19}F NMR (376 MHz, DMSO-*d*$_6$)

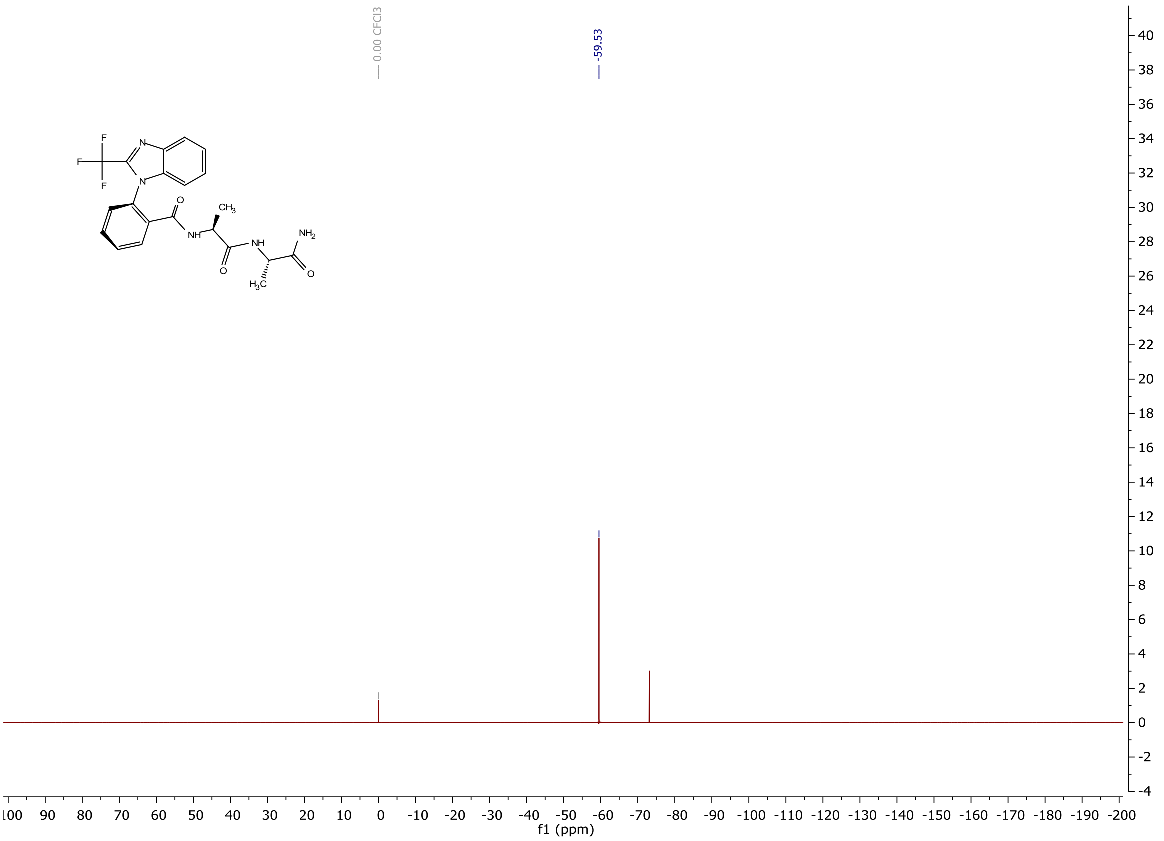

^{19}F NMR (76 MHz, THF)

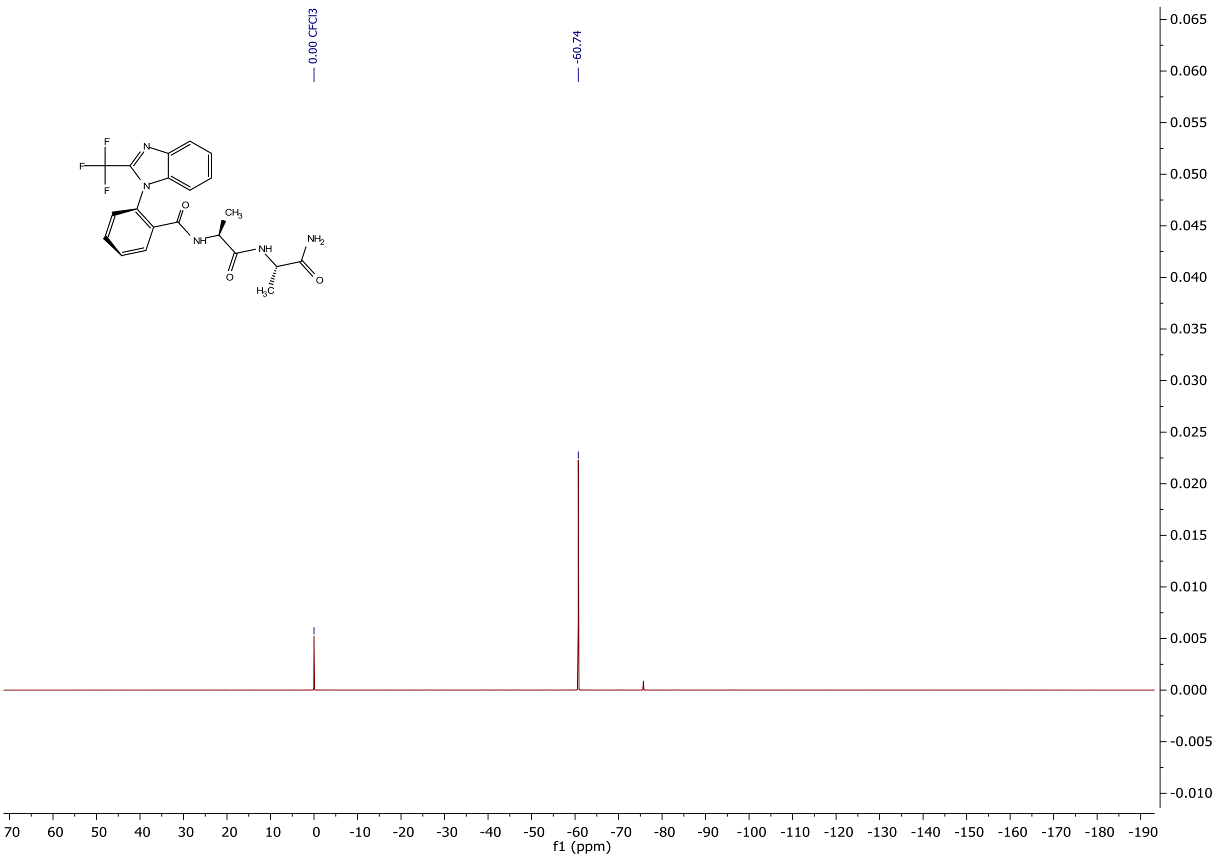

(*M*)-TBBA-(*L*)-Ala-(*L*)-Ala-NH_2 **(*M*)-20**

^{1}**H NMR** (400 MHz, DMSO-d_6)

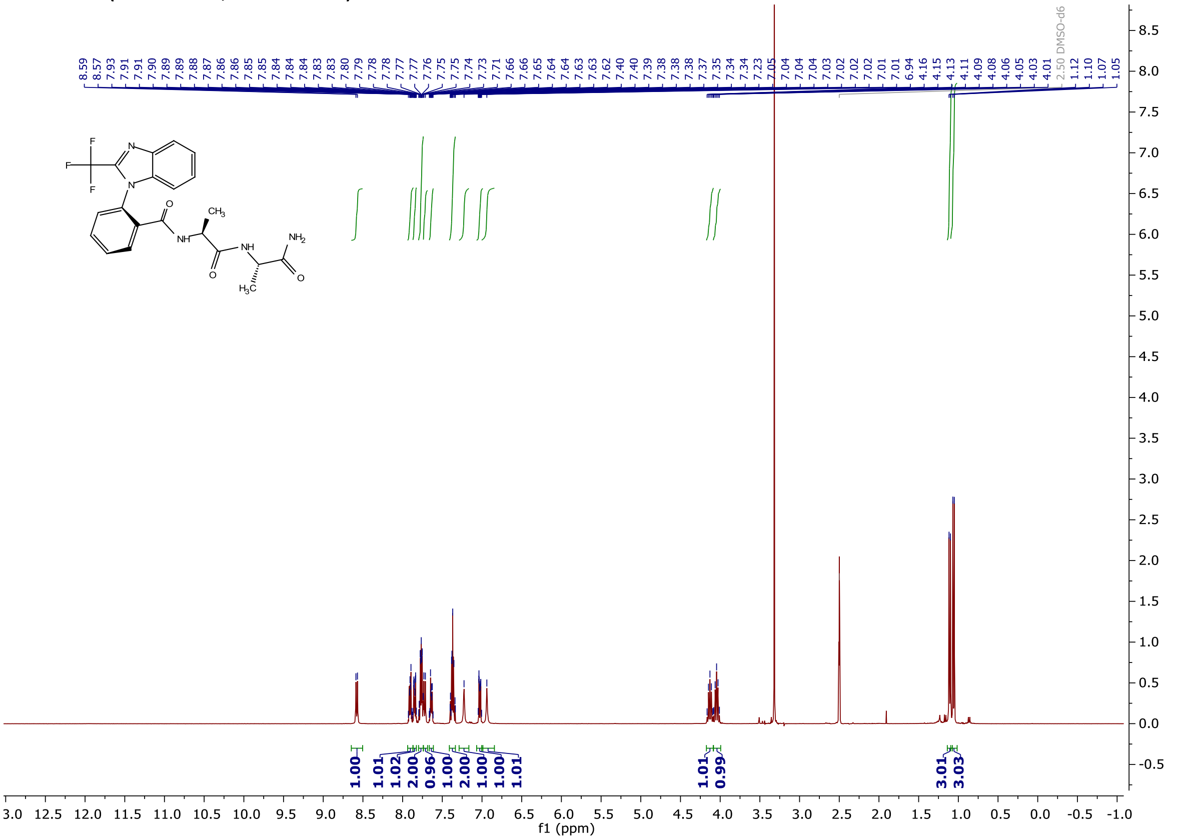

^{13}**C{^{1}H} NMR** (101 MHz, DMSO-d_6)

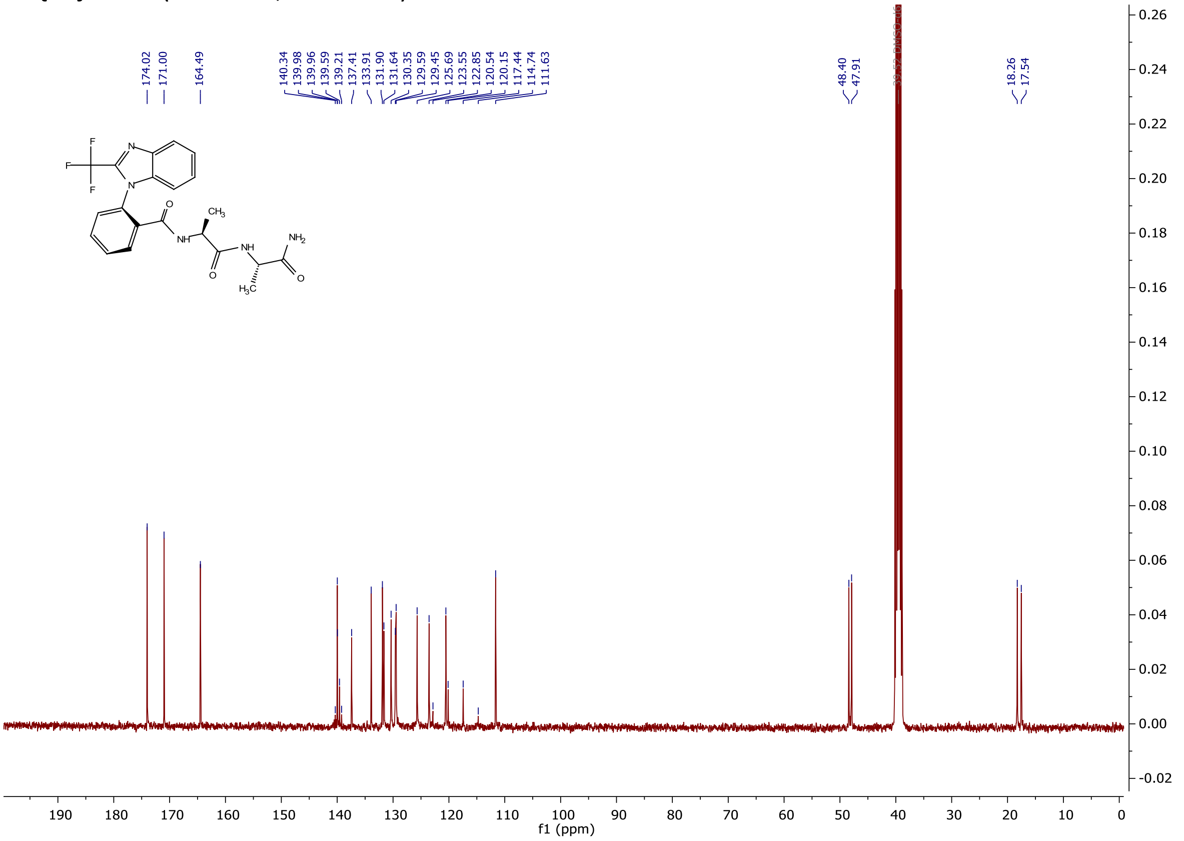

^{19}**F NMR** (376 MHz, DMSO-d_6)

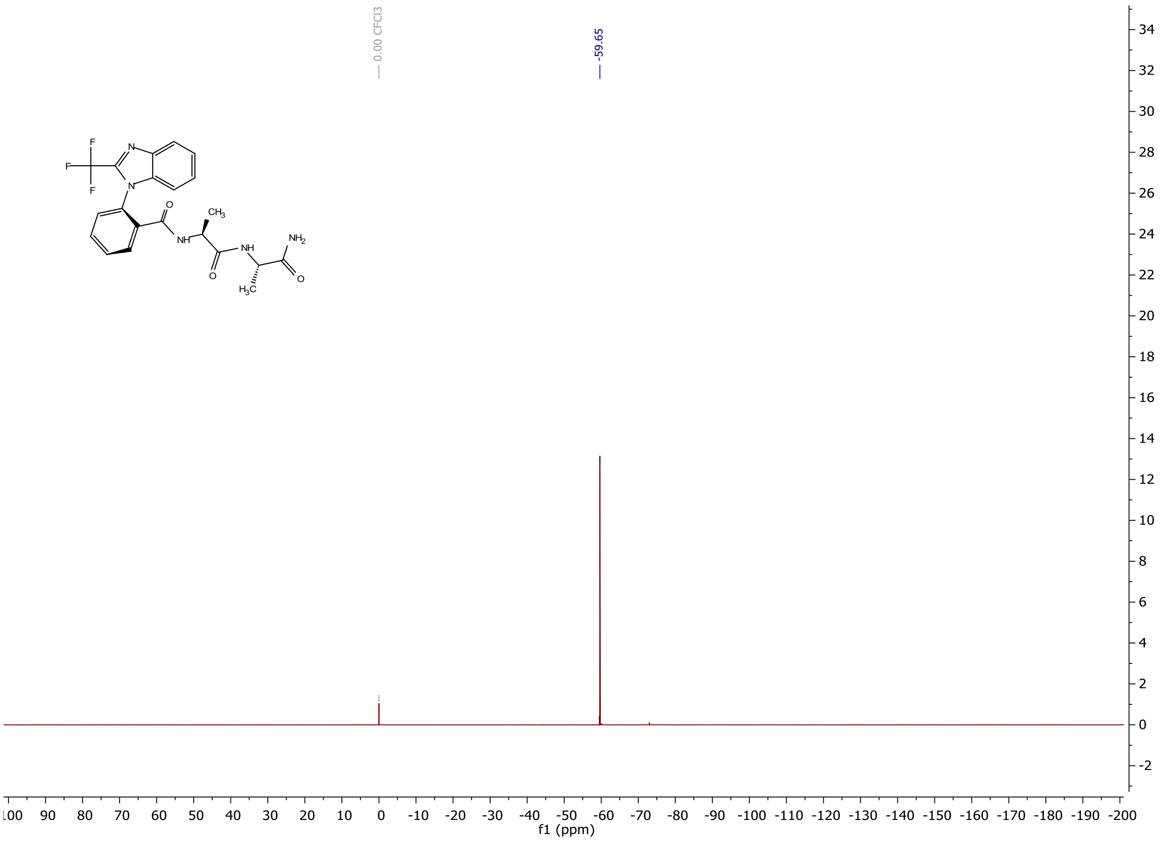

^{19}**F NMR** (76 MHz, THF)

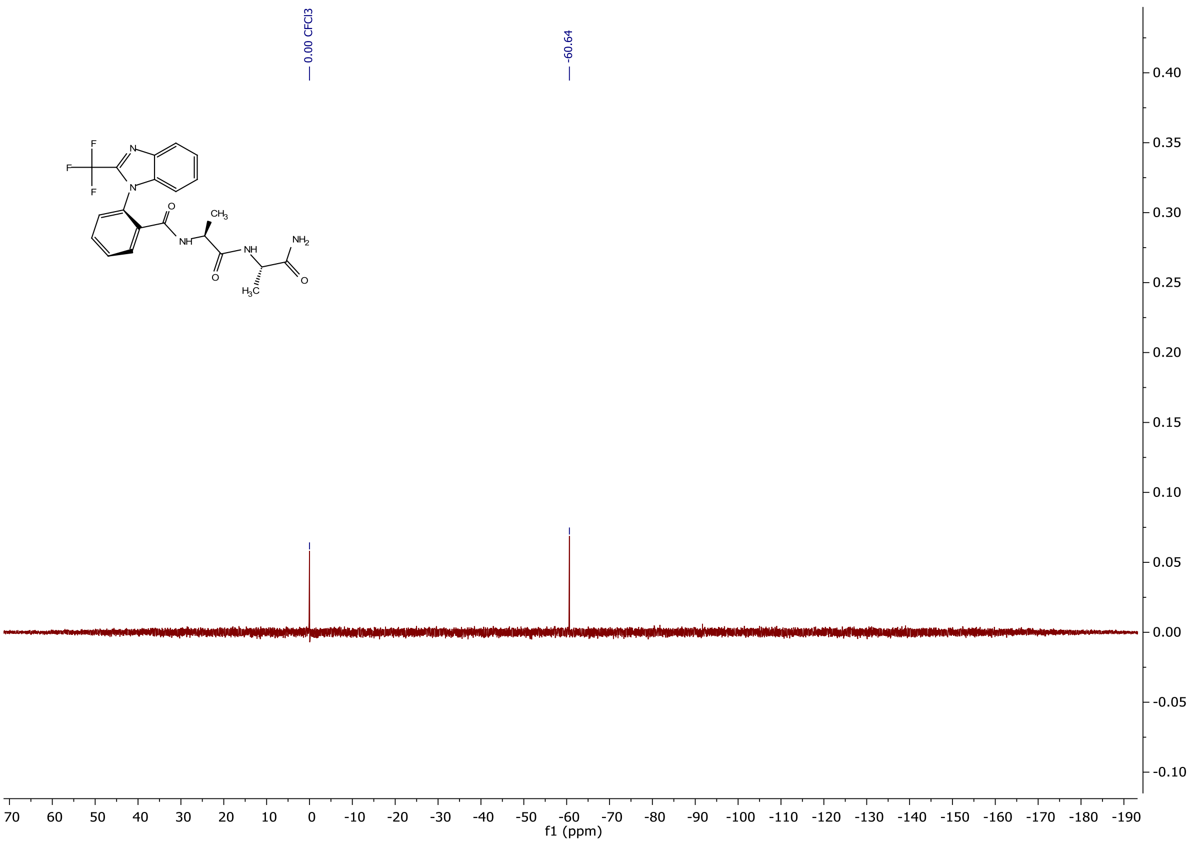

(P)-TBBA-(*L*)-Ala-(*L*)-Phe-NH_2 ***(P)*-21**

^{1}H NMR (400 MHz, DMSO-*d*$_6$)

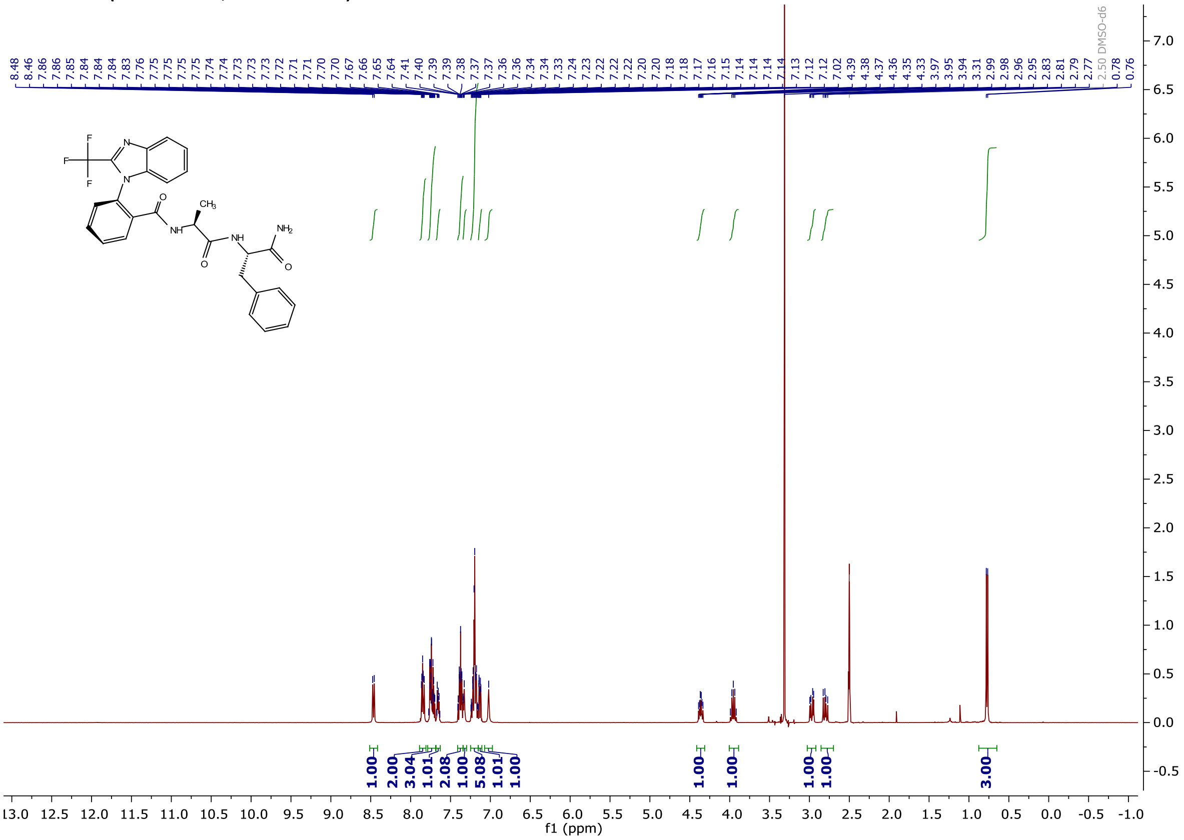

^{13}C{^{1}H} NMR (101 MHz, DMSO-*d*$_6$)

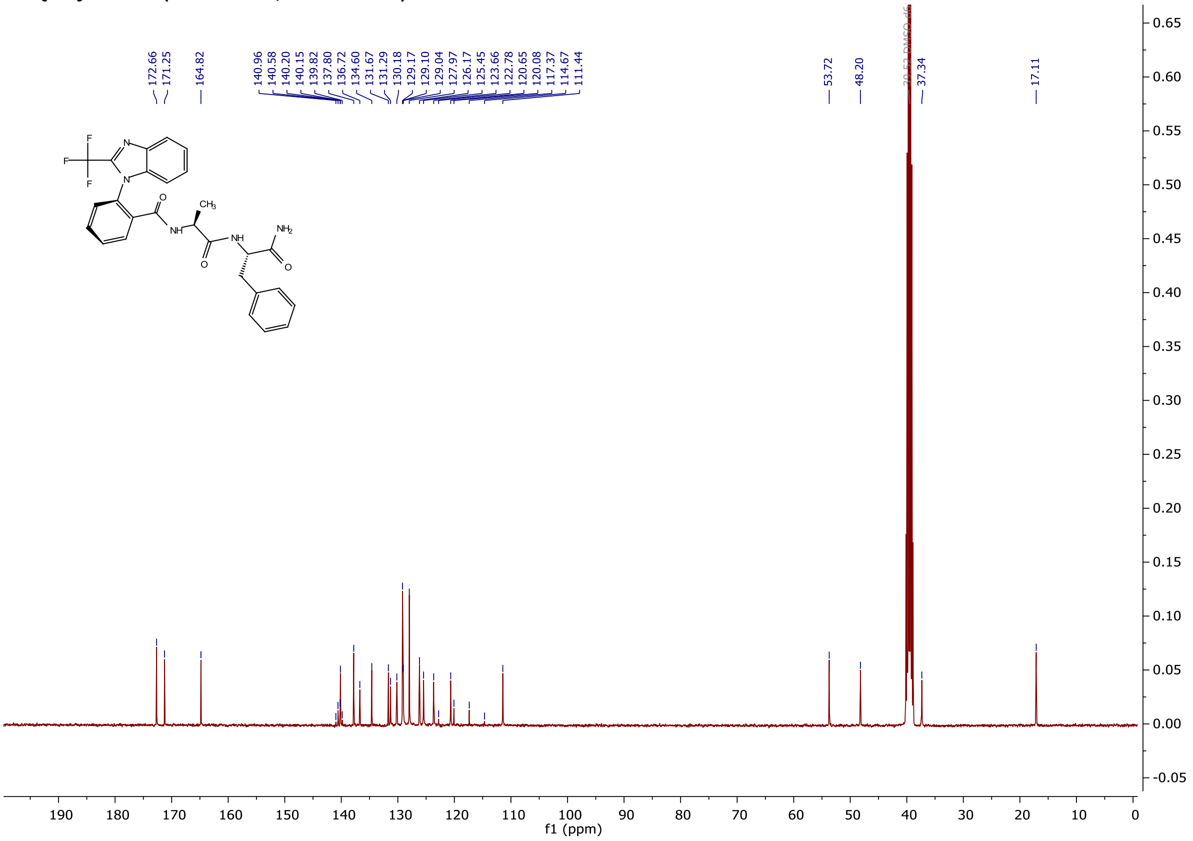

^{19}F NMR (376 MHz, DMSO-d_6)

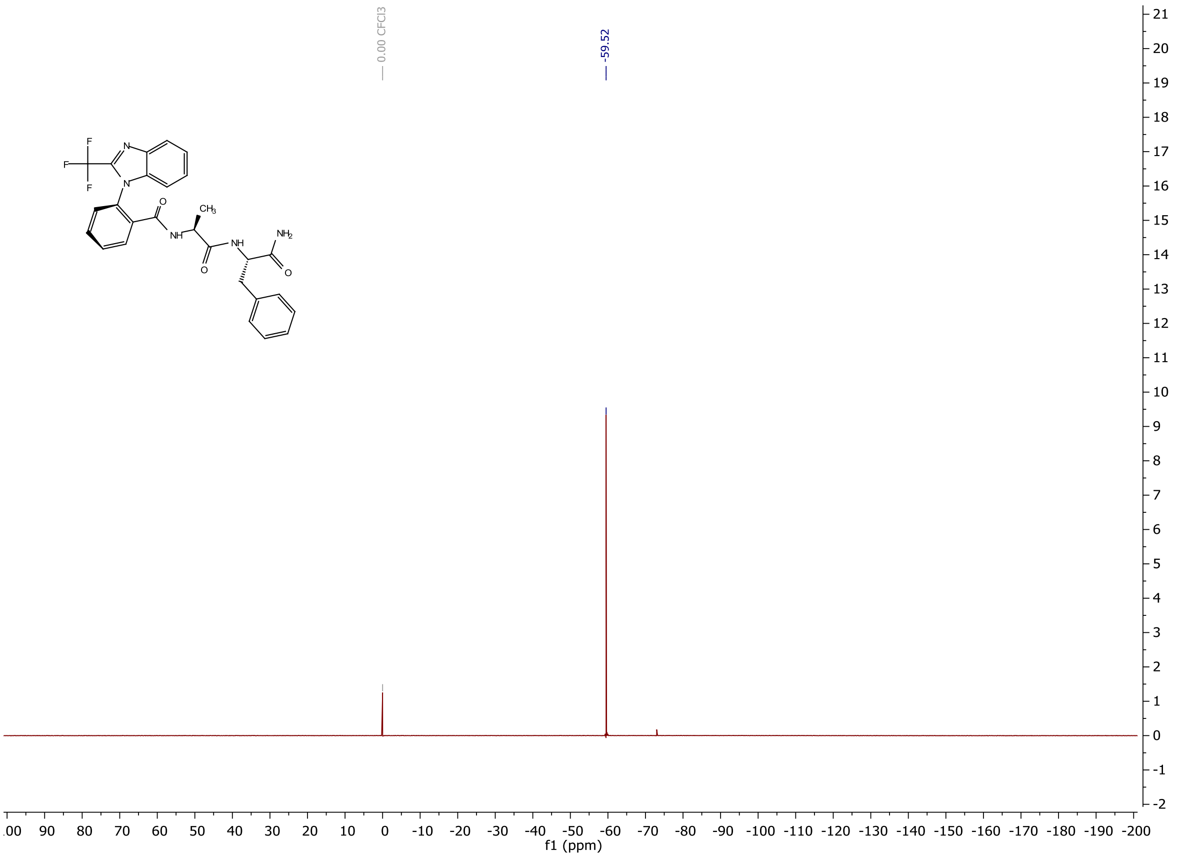

^{19}F NMR (76 MHz, THF)

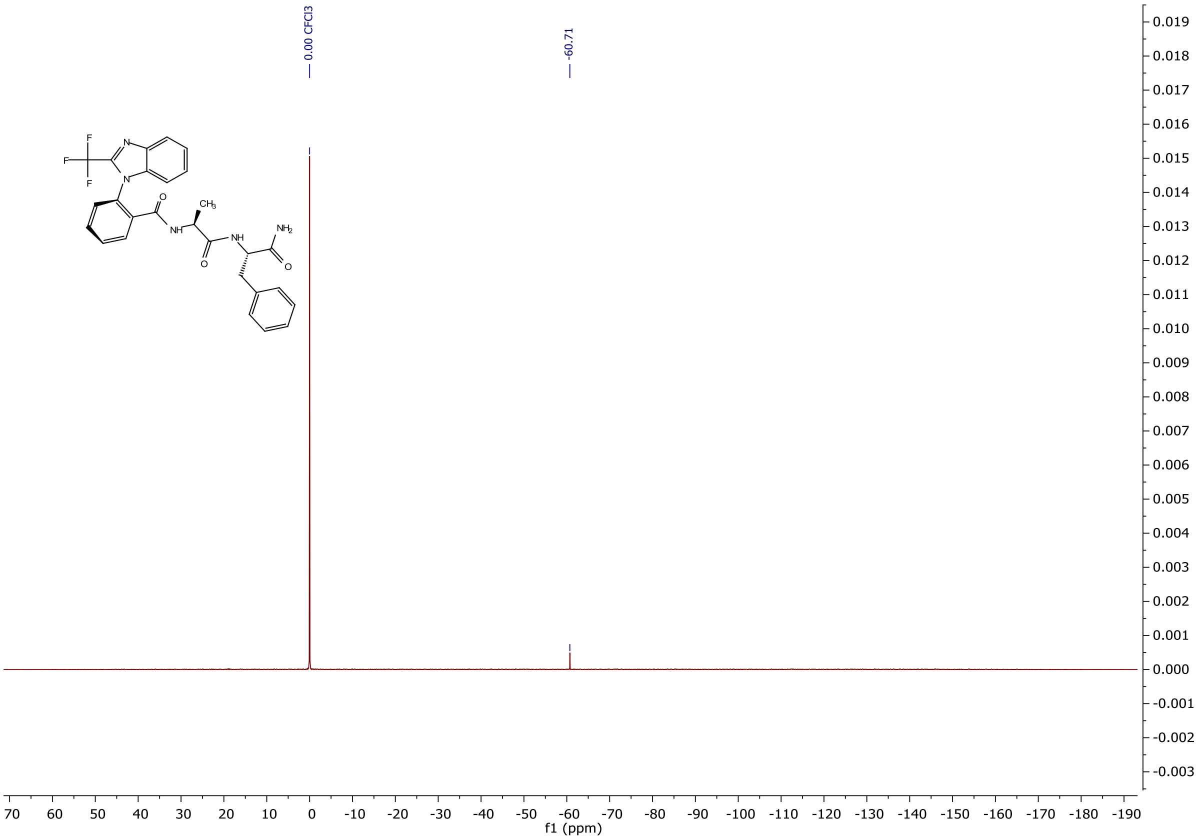

(*M*)-TBBA-(L)-Ala-(L)-Phe-NH_2 **(*M*)-21**

^{1}H NMR (400 MHz, DMSO-d_6)

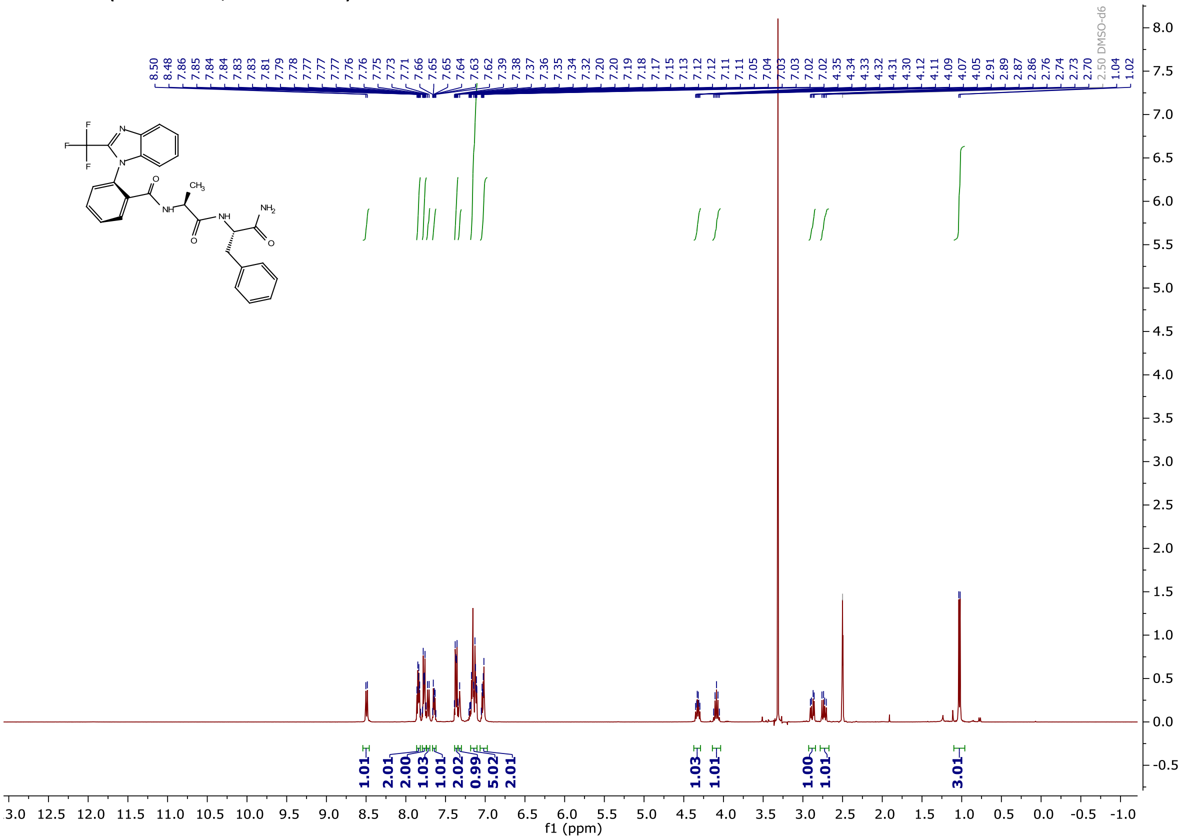

$^{13}C\{^{1}H\}$ NMR (101 MHz, DMSO-d_6)

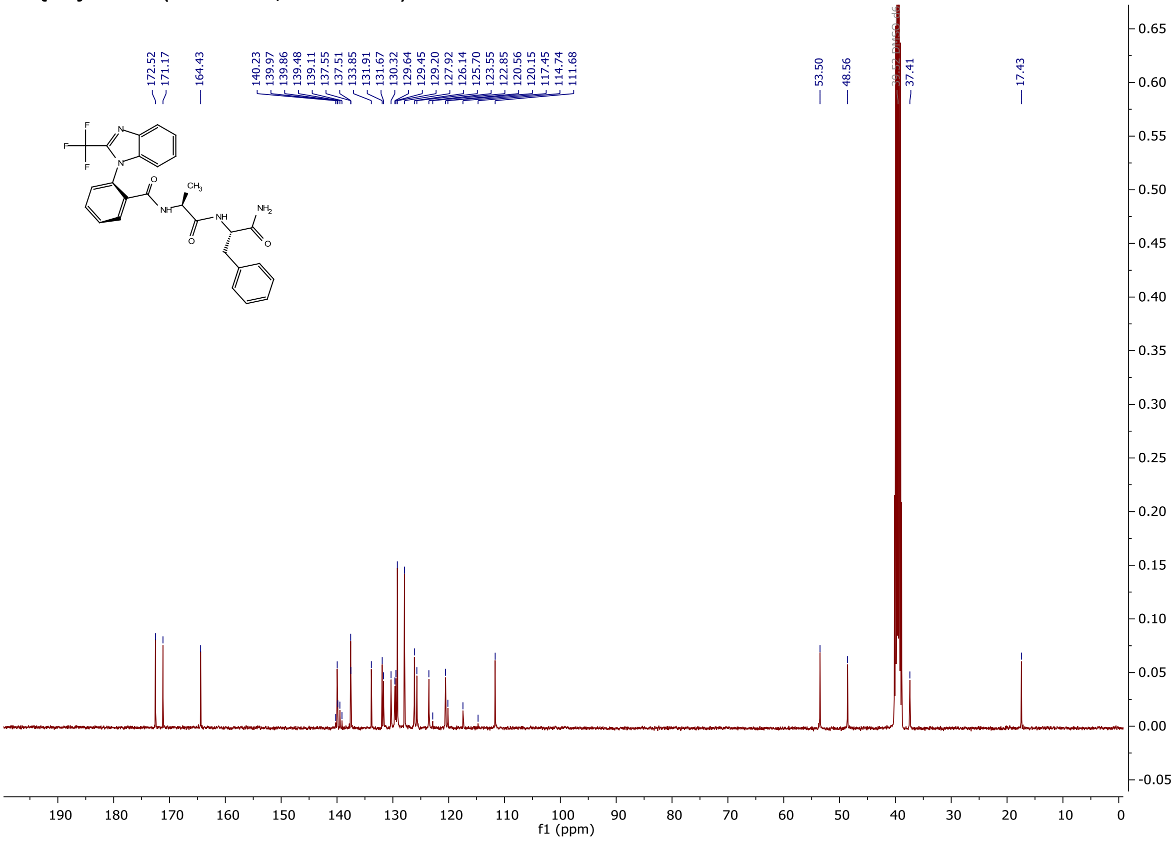

^{19}F NMR (376 MHz, DMSO-d_6)

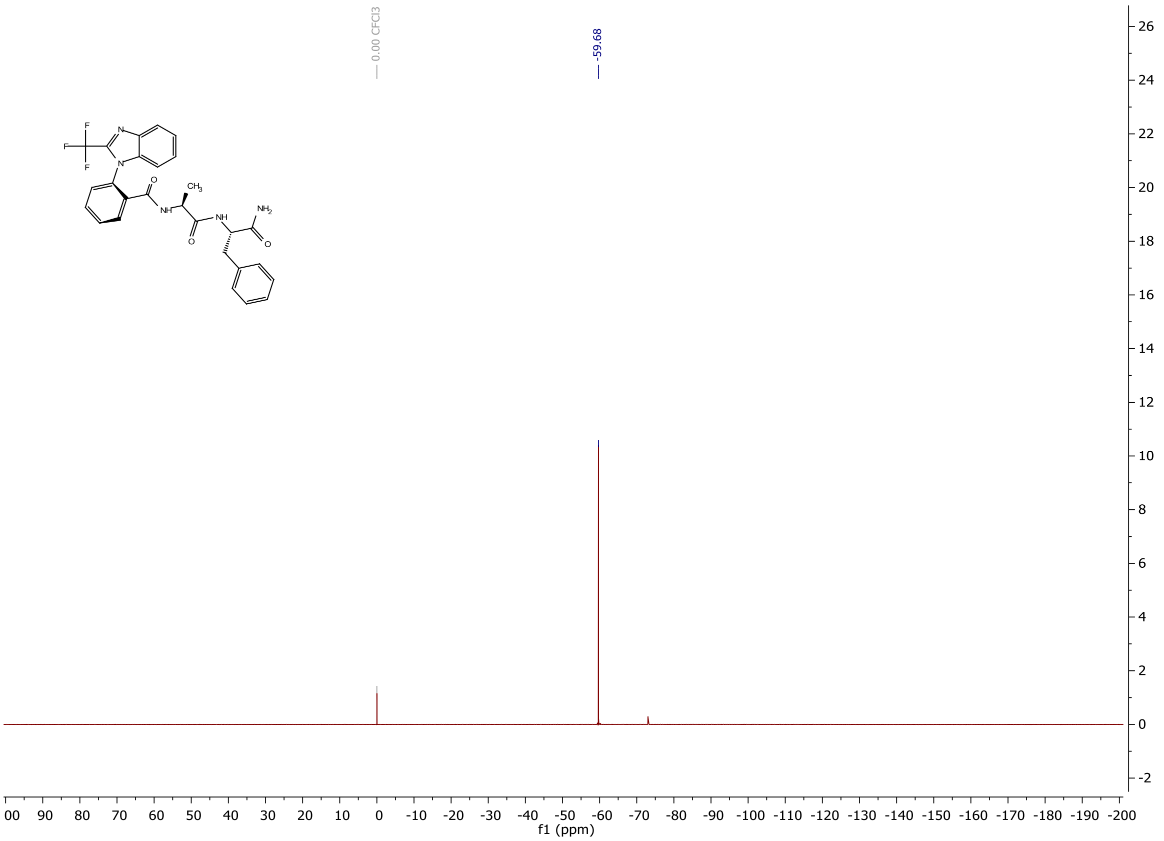

^{19}F NMR (76 MHz, THF)

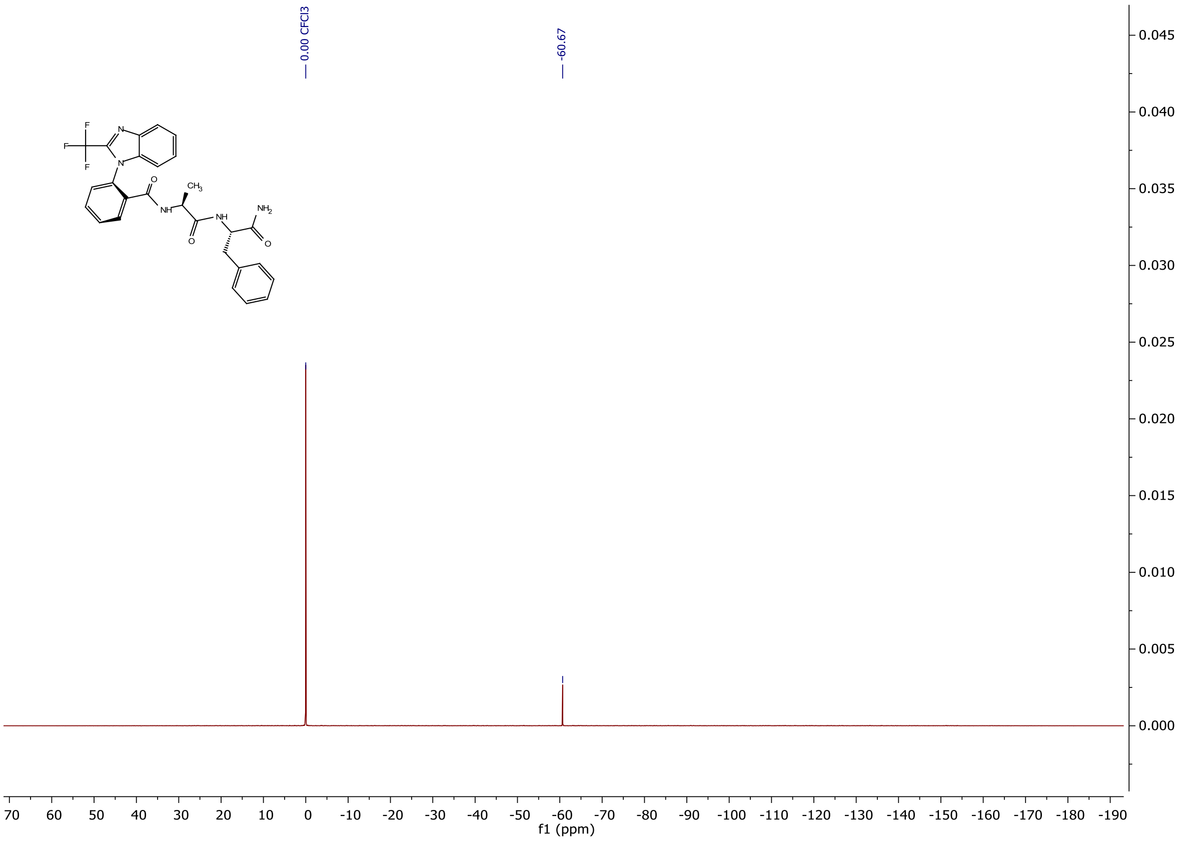

(*P*)-TBBA-(*L*)-Ala-(*D*)-Phe-NH2 **(*P*)-22**

^{1}H NMR (400 MHz, DMSO-d_6)

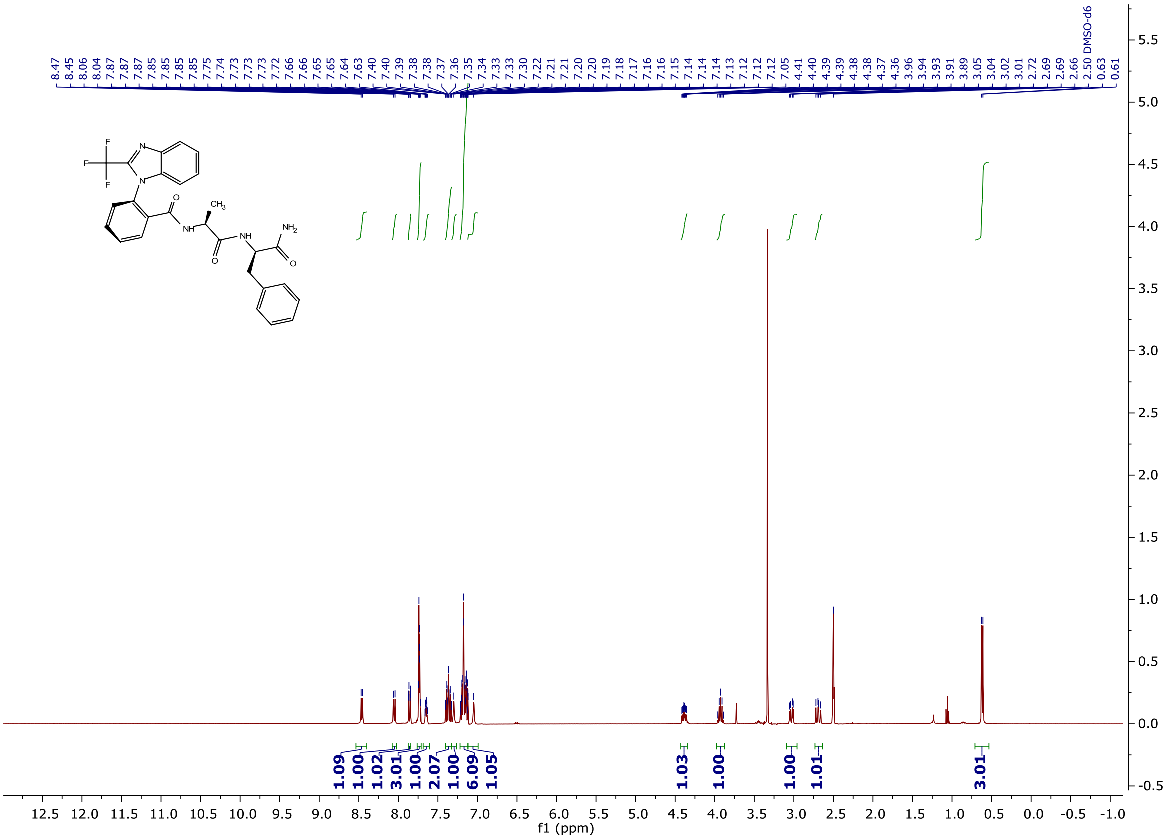

^{13}C{^{1}H} NMR (101 MHz, DMSO-d_6)

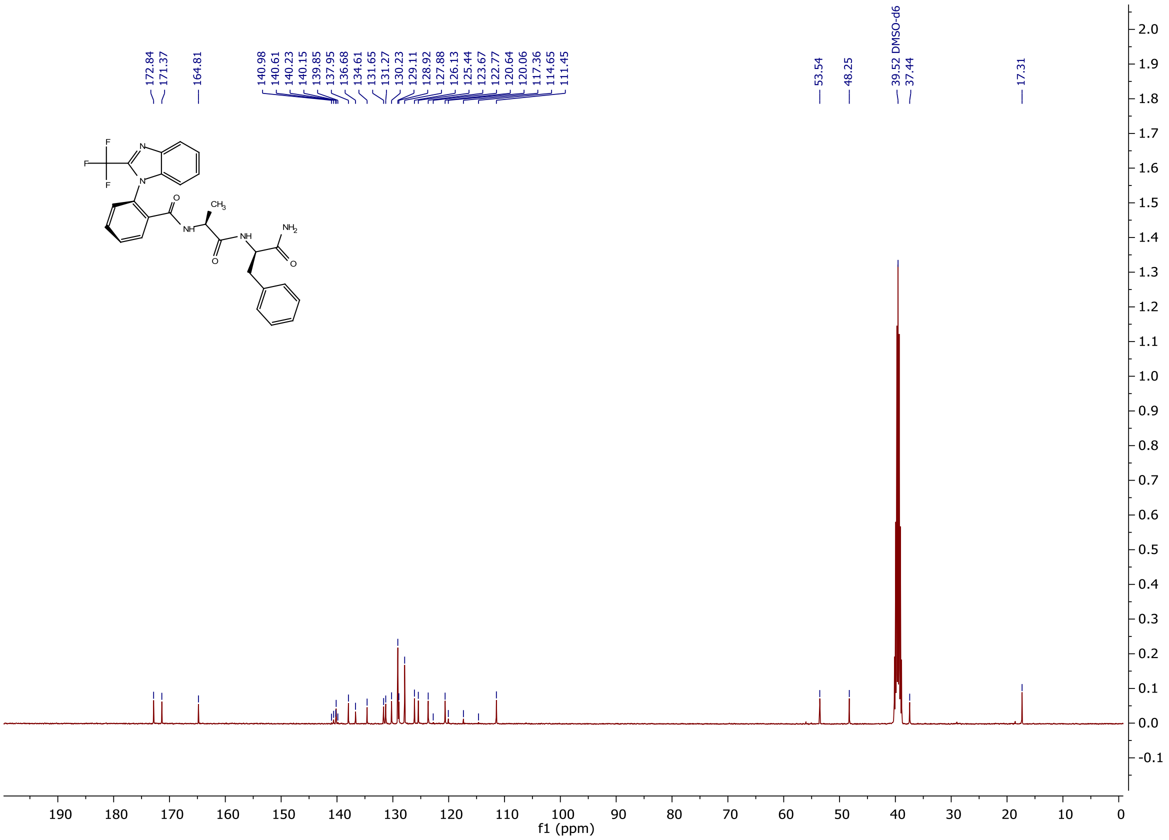

^{19}**F NMR** (376 MHz, DMSO-d_6)

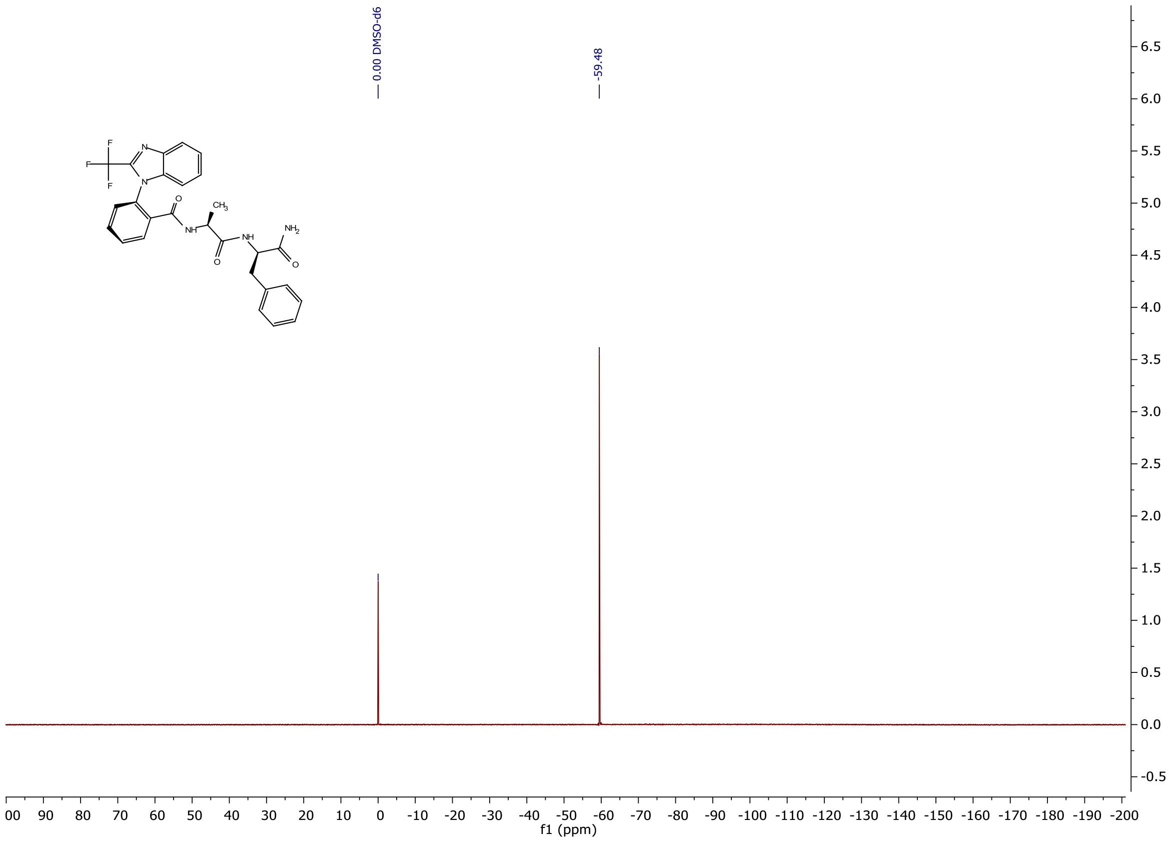

^{19}**F NMR** (76 MHz, THF)

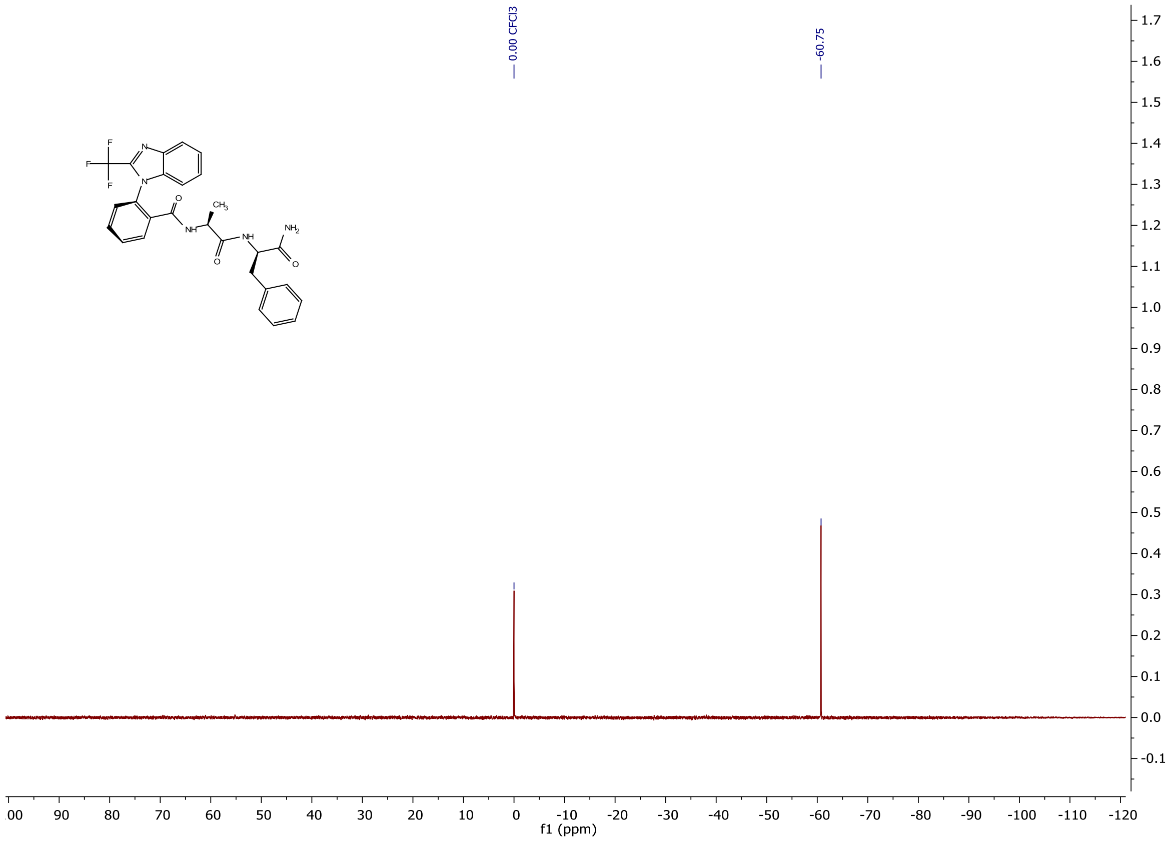

(*M*)-TBBA-(L)-Ala-(D)-Phe-NH_2 **(*M*)-22**

^{1}H NMR (400 MHz, DMSO-d_6)

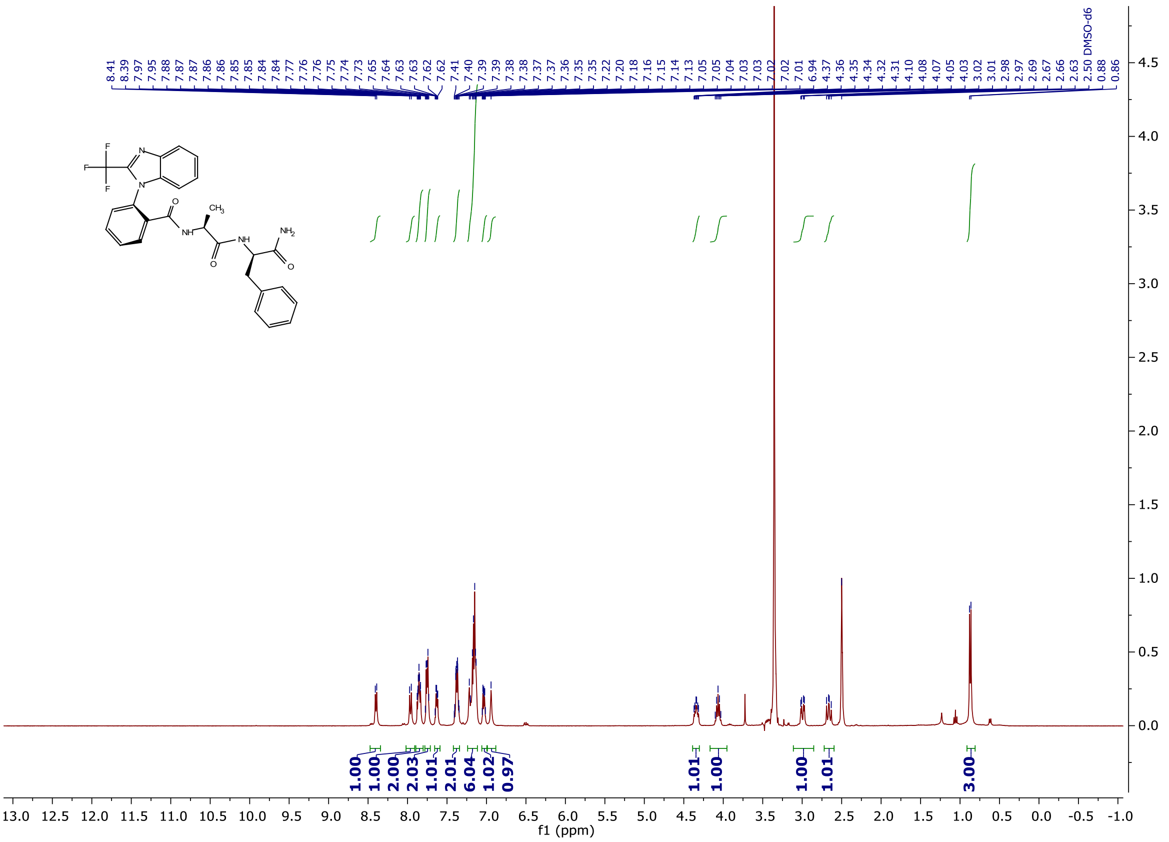

^{13}C{^{1}H} NMR (101 MHz, DMSO-d_6)

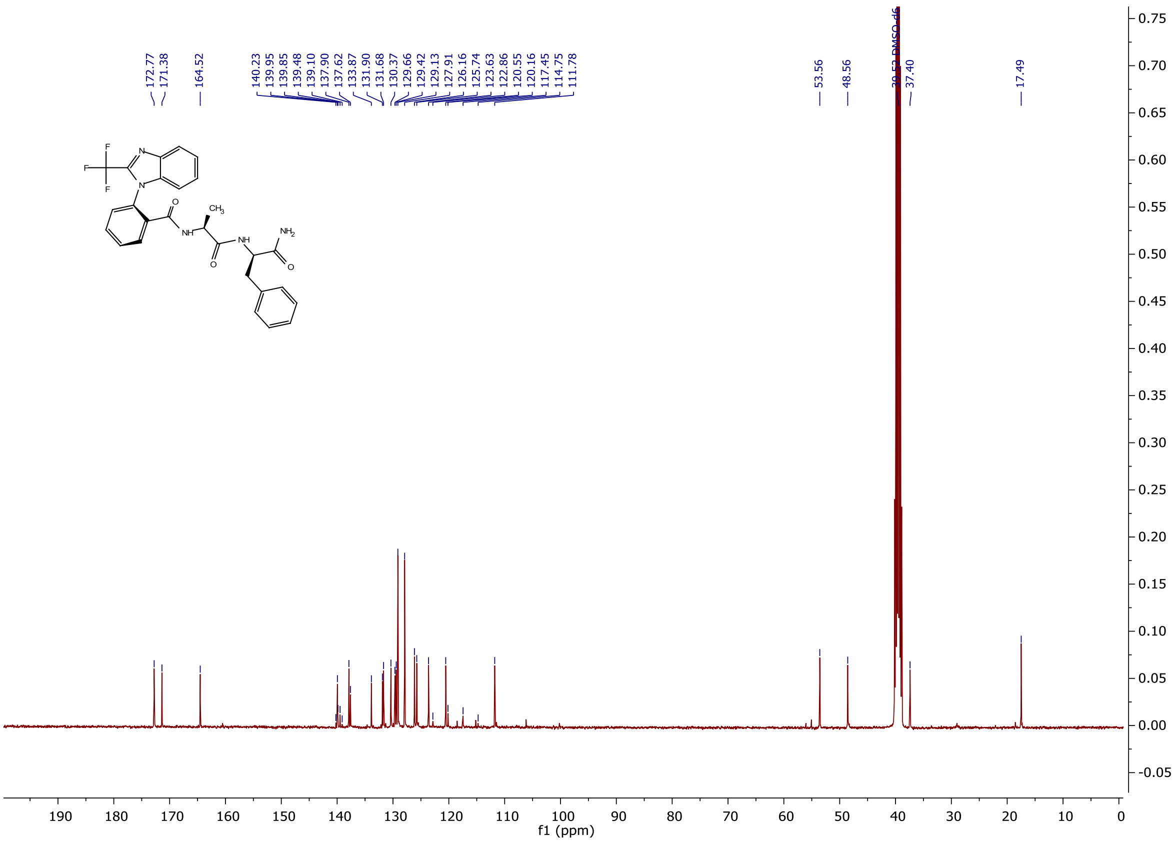

^{19}F NMR (376 MHz, DMSO-d_6)

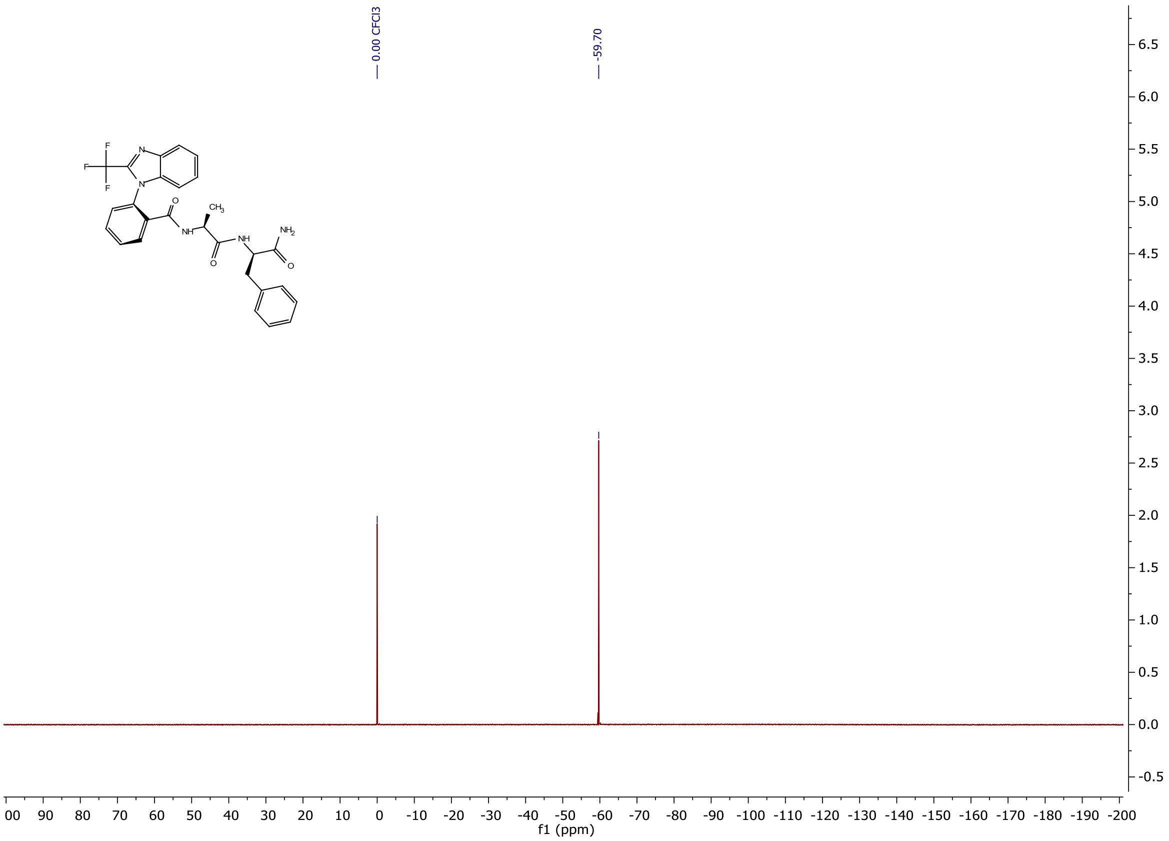

^{19}F NMR (76 MHz, THF)

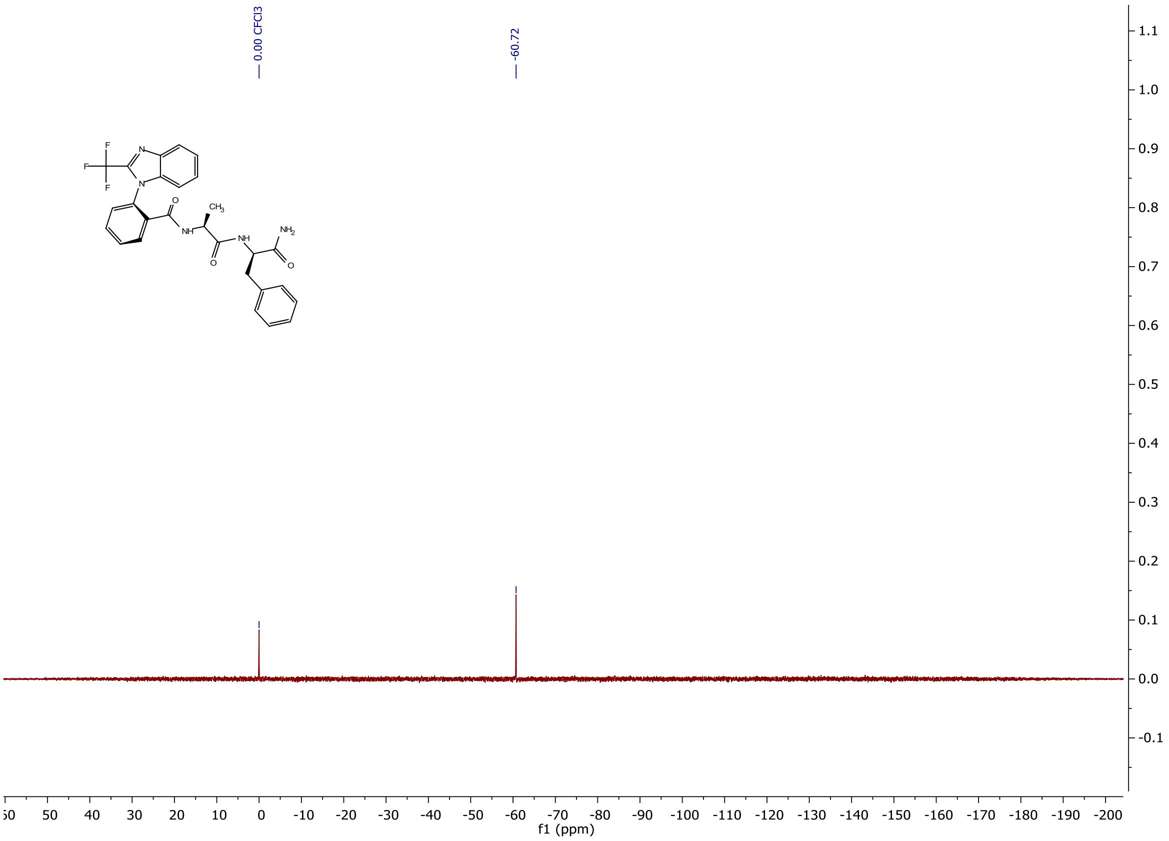

(P)-TBBA-(*D*)-Ala-(*L*)-Phe-NH2 **(*P*)-23**

^{1}H NMR (400 MHz, DMSO-*d*6)

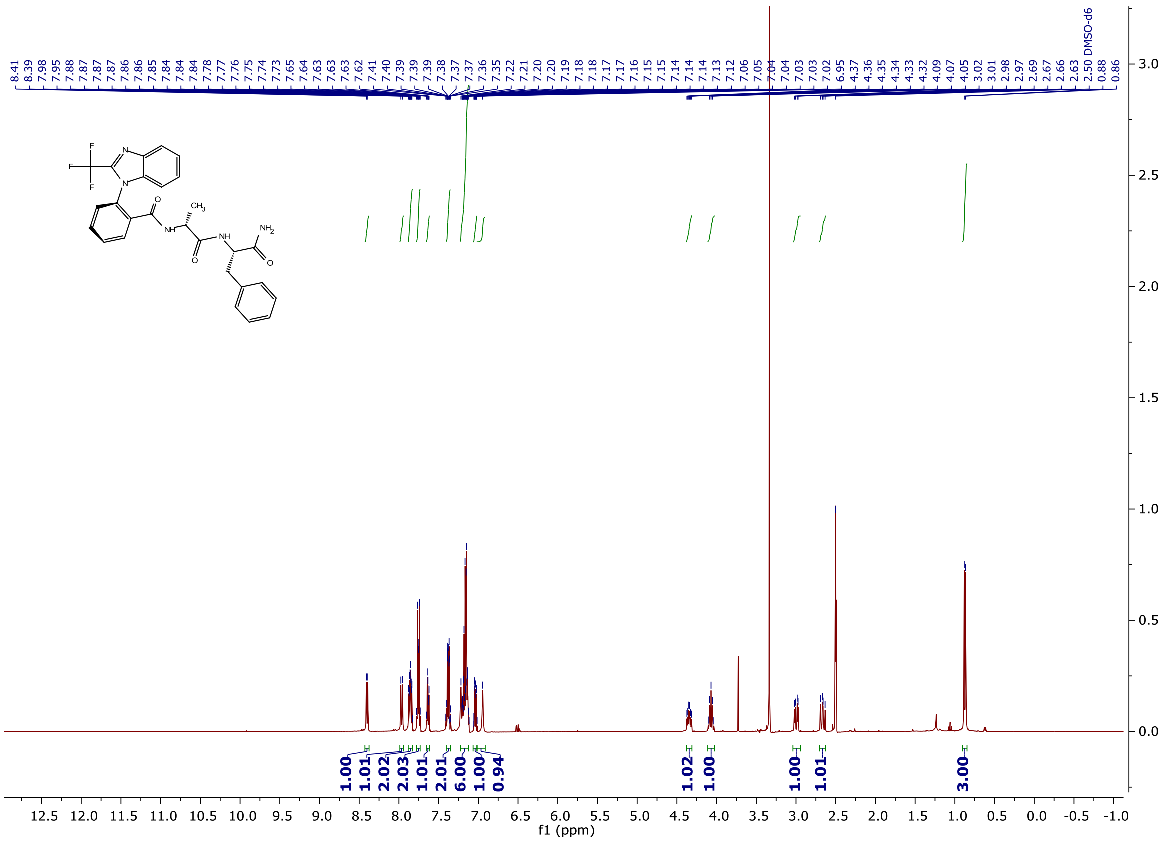

^{13}C{^{1}H} NMR (101 MHz, DMSO-*d*6)

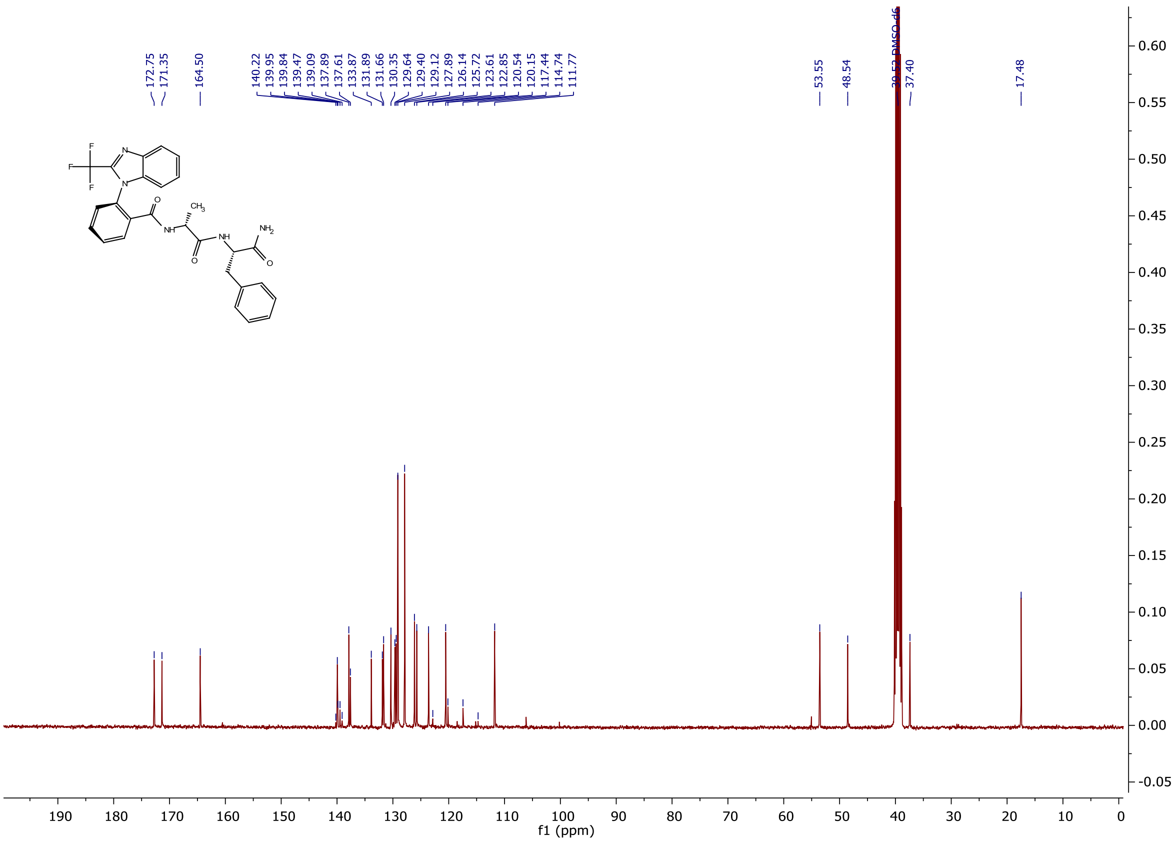

^{19}F NMR (376 MHz, DMSO-d_6)

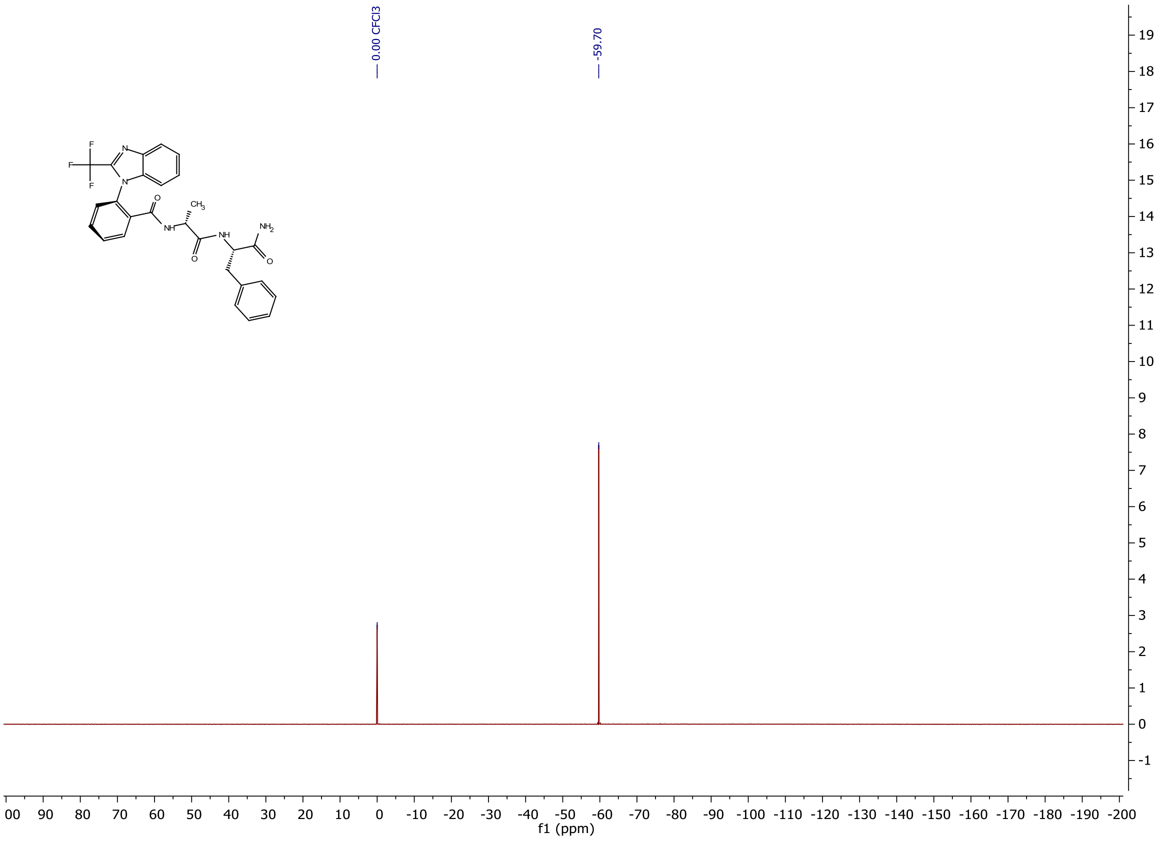

^{19}F NMR (76 MHz, THF)

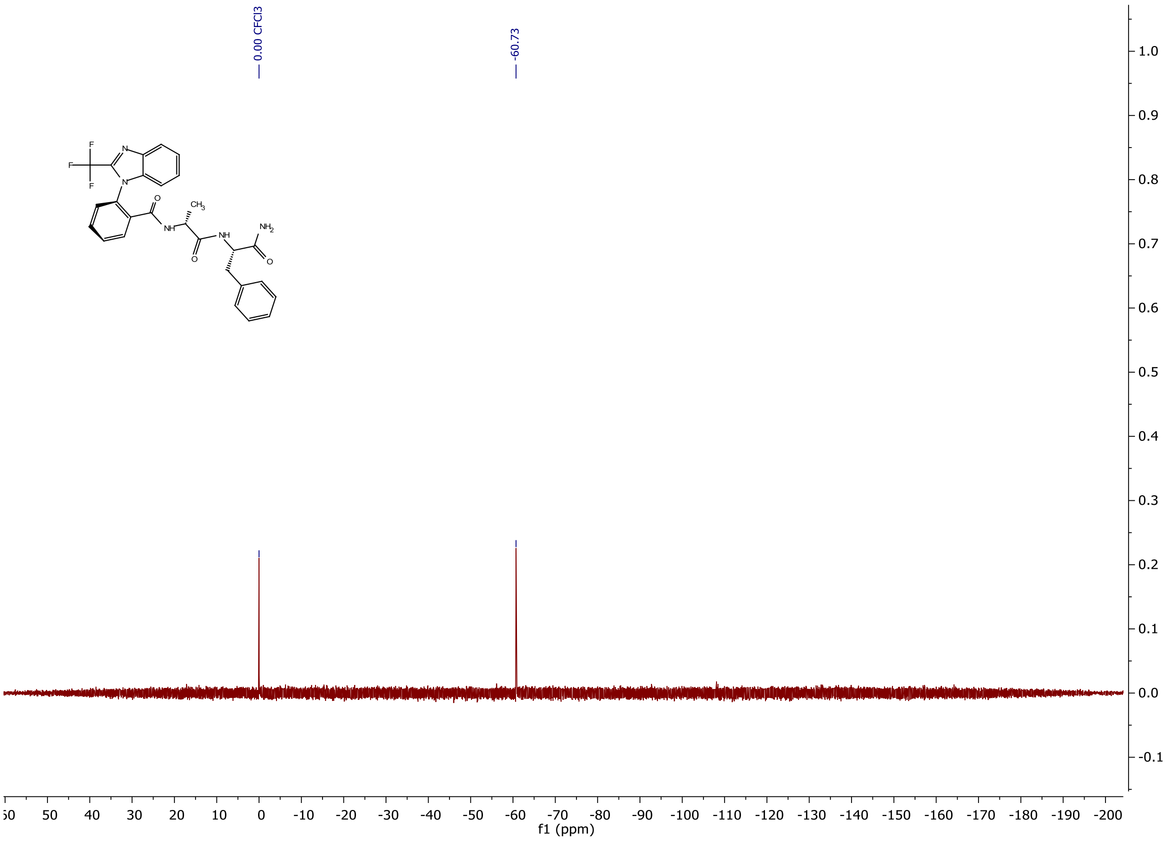

(*M*)-TBBA-(*D*)-Ala-(*L*)-Phe-NH_2 **(*M*)-23**

^{1}**H NMR** (400 MHz, DMSO-d_6)

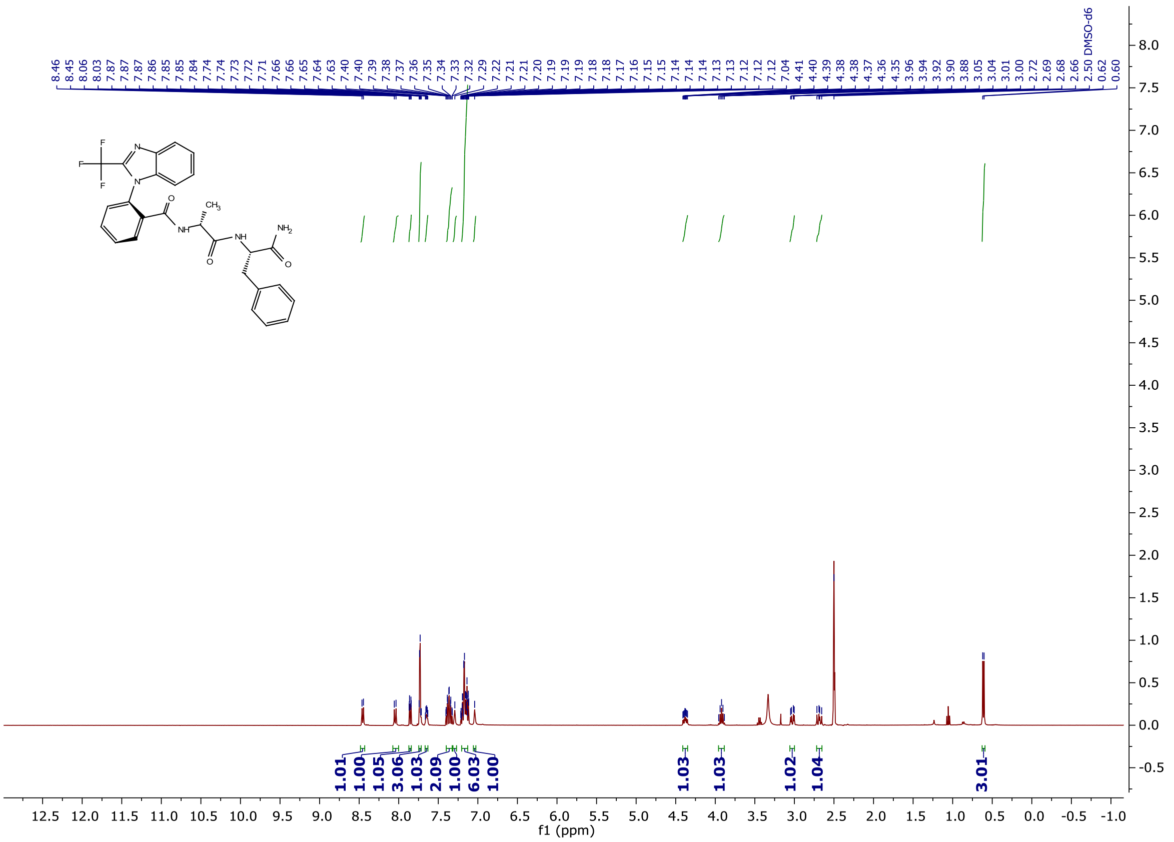

^{13}**C{^{1}H} NMR** (101 MHz, DMSO-d_6)

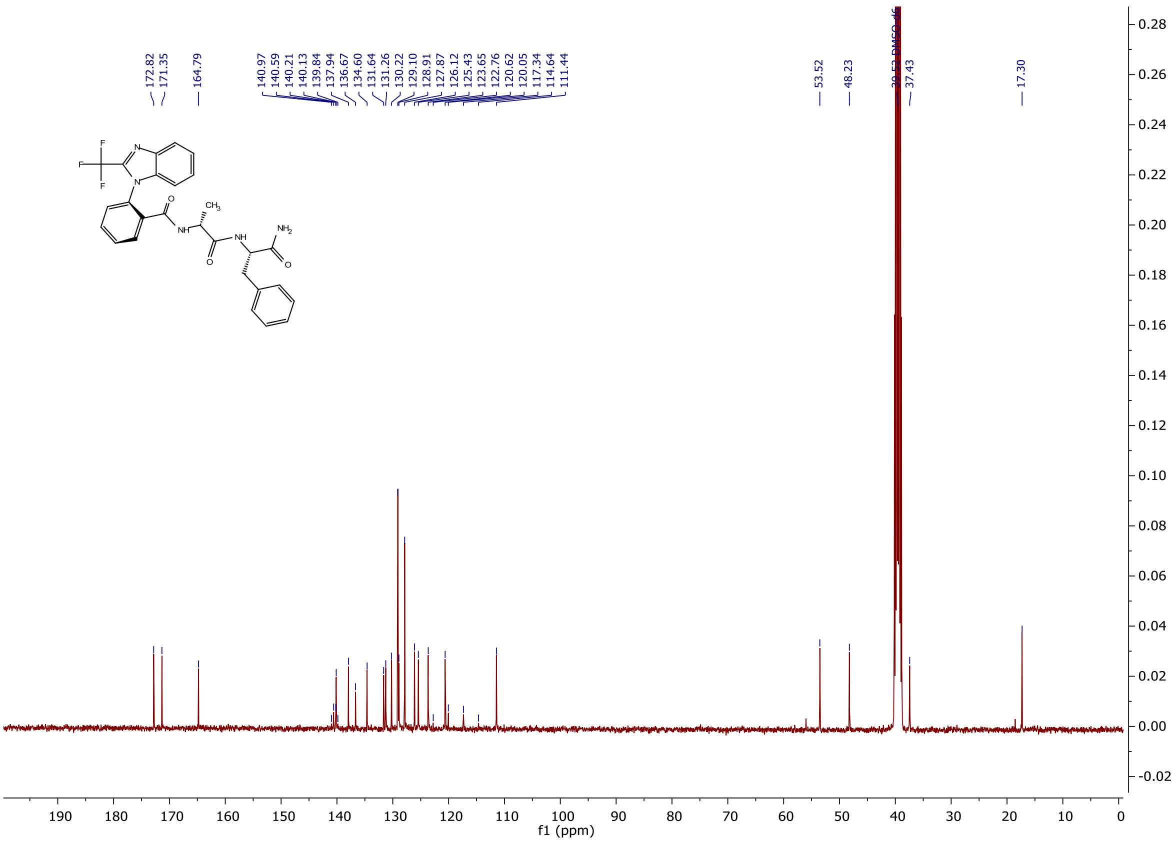

^{19}F NMR (376 MHz, DMSO-d_6)

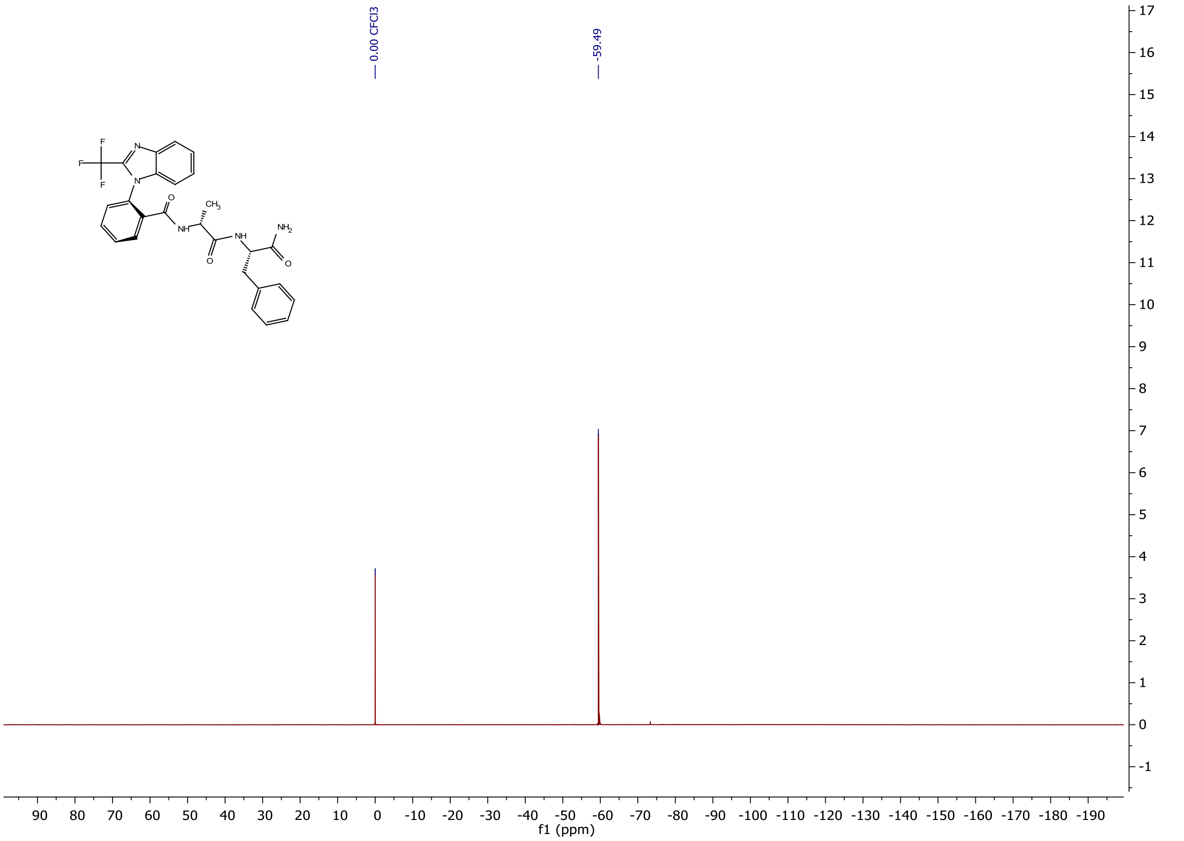

^{19}F NMR (76 MHz, THF)

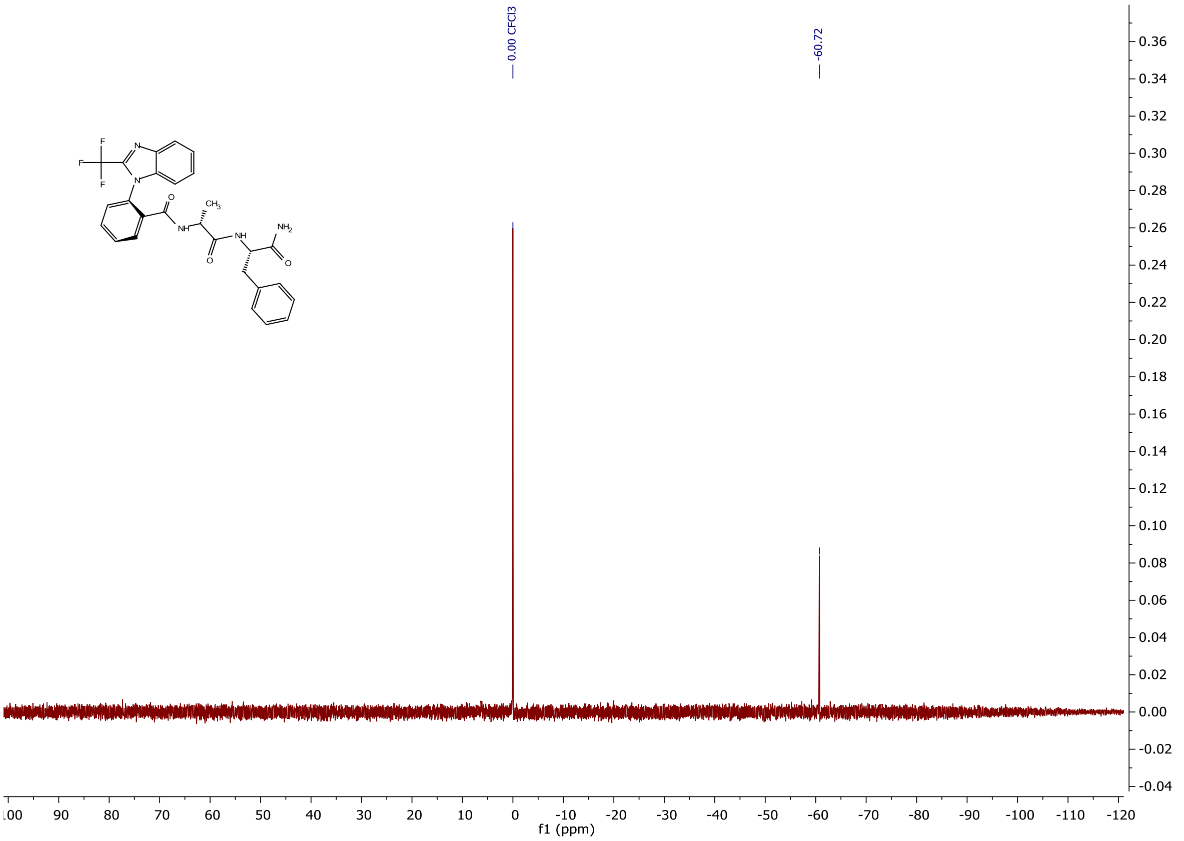

(*P*)-TBBA-(L)-Phe-(L)-Ala-NH_2 **(*P*)-24**

^{1}H NMR (400 MHz, DMSO-d_6)

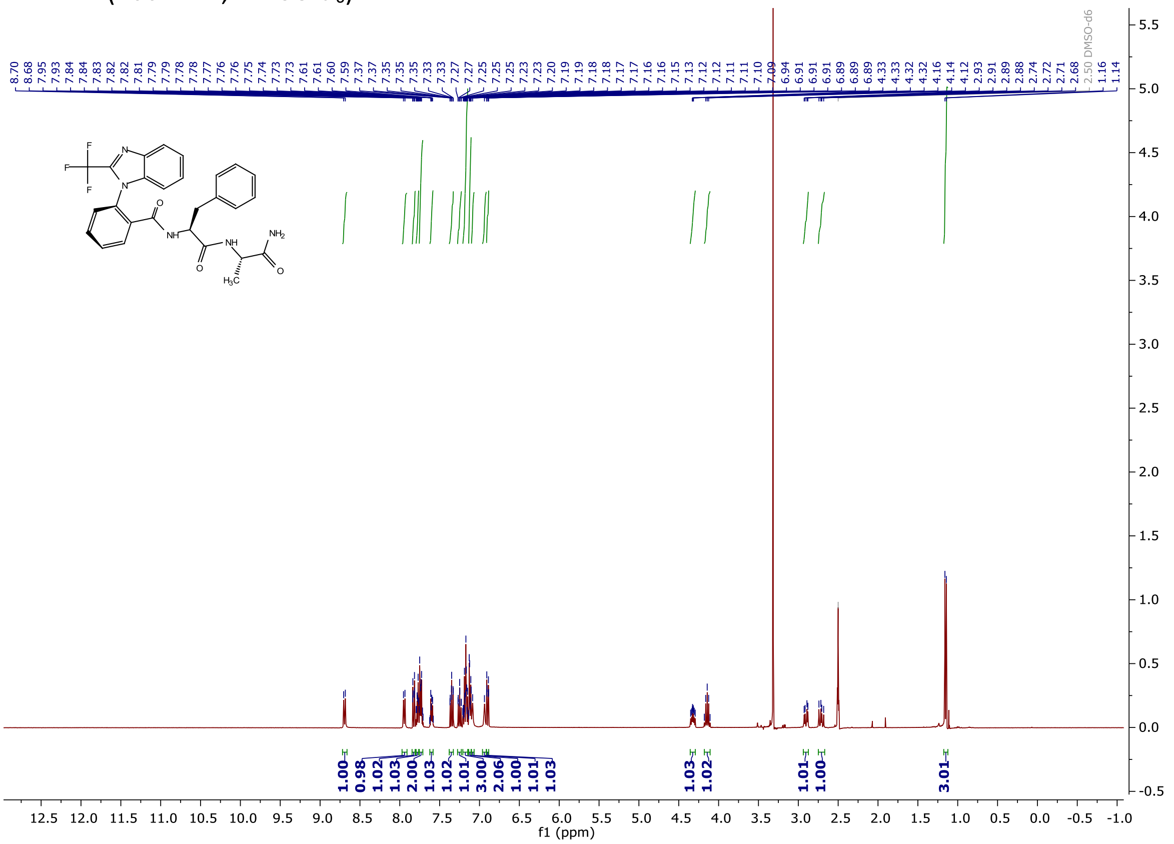

^{13}C{^{1}H} NMR (101 MHz, DMSO-d_6)

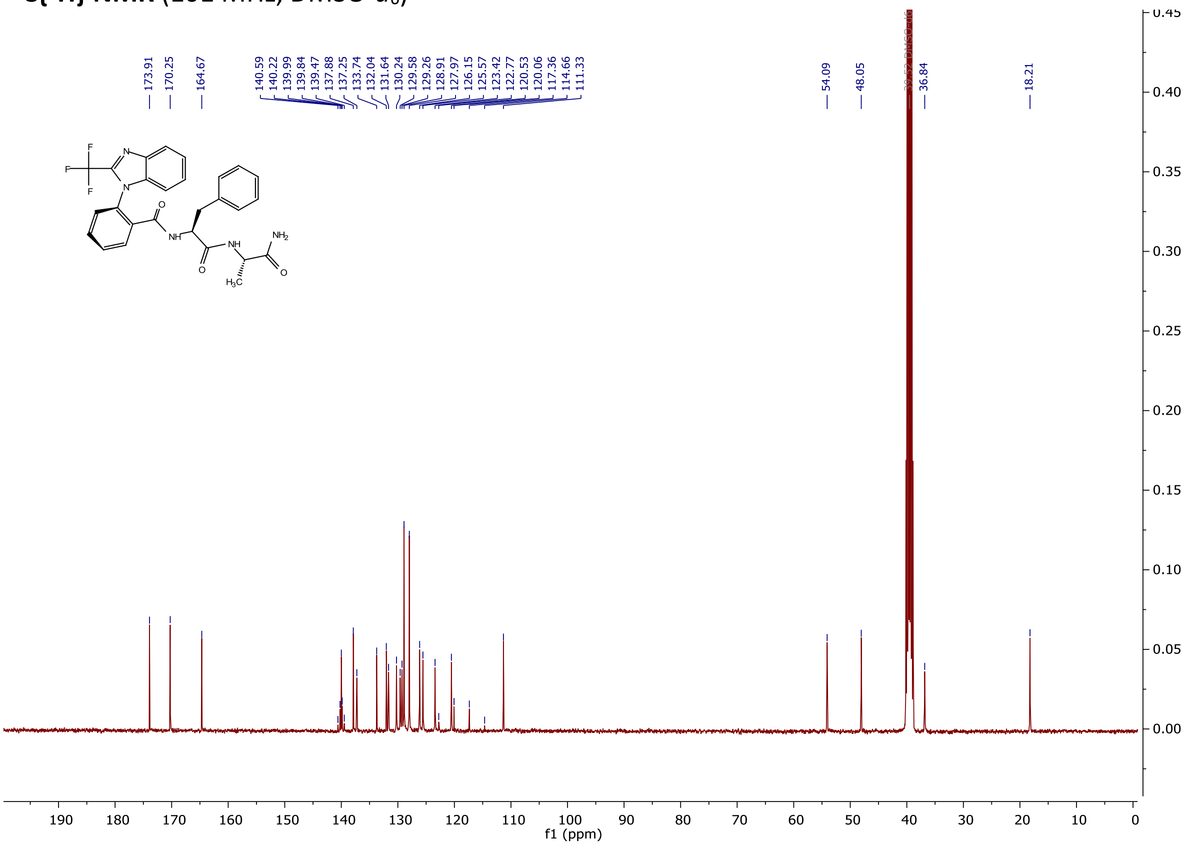

^{19}**F NMR** (376 MHz, DMSO-d_6)

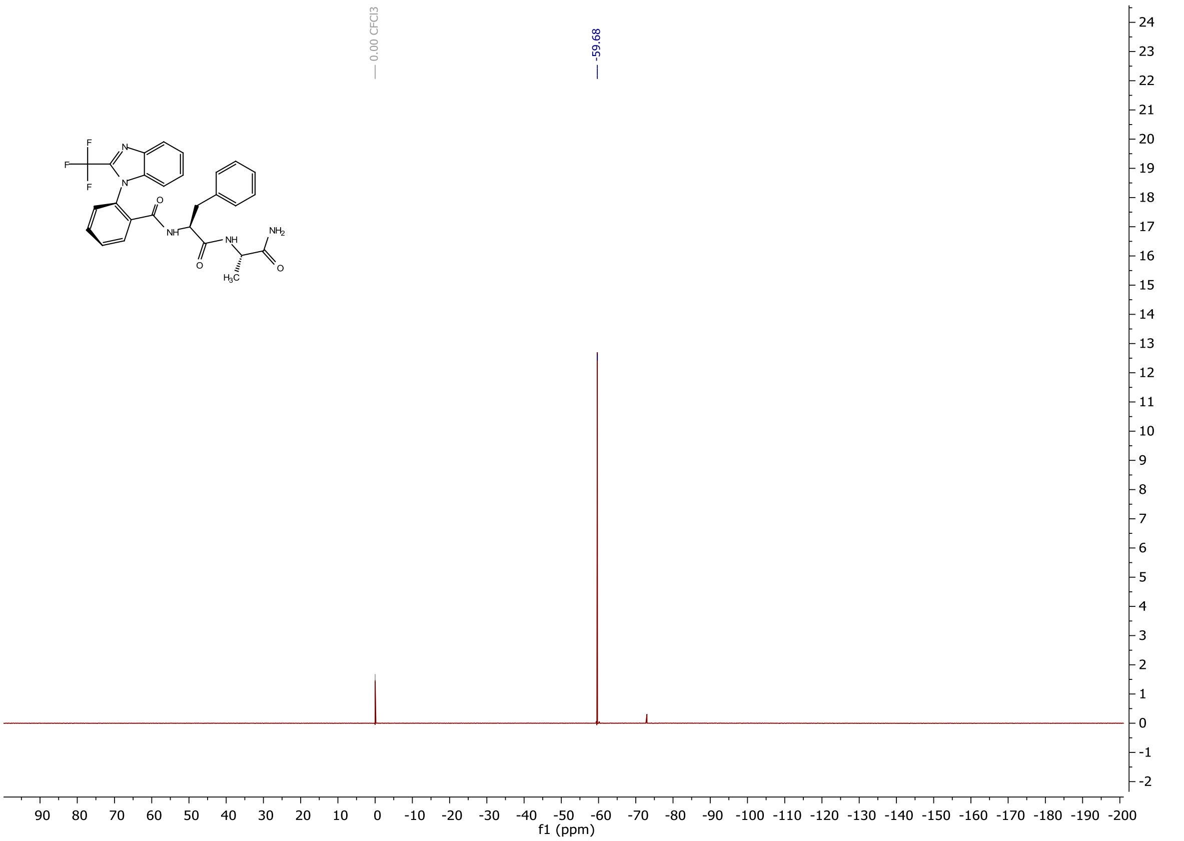

^{19}**F NMR** (76 MHz, THF)

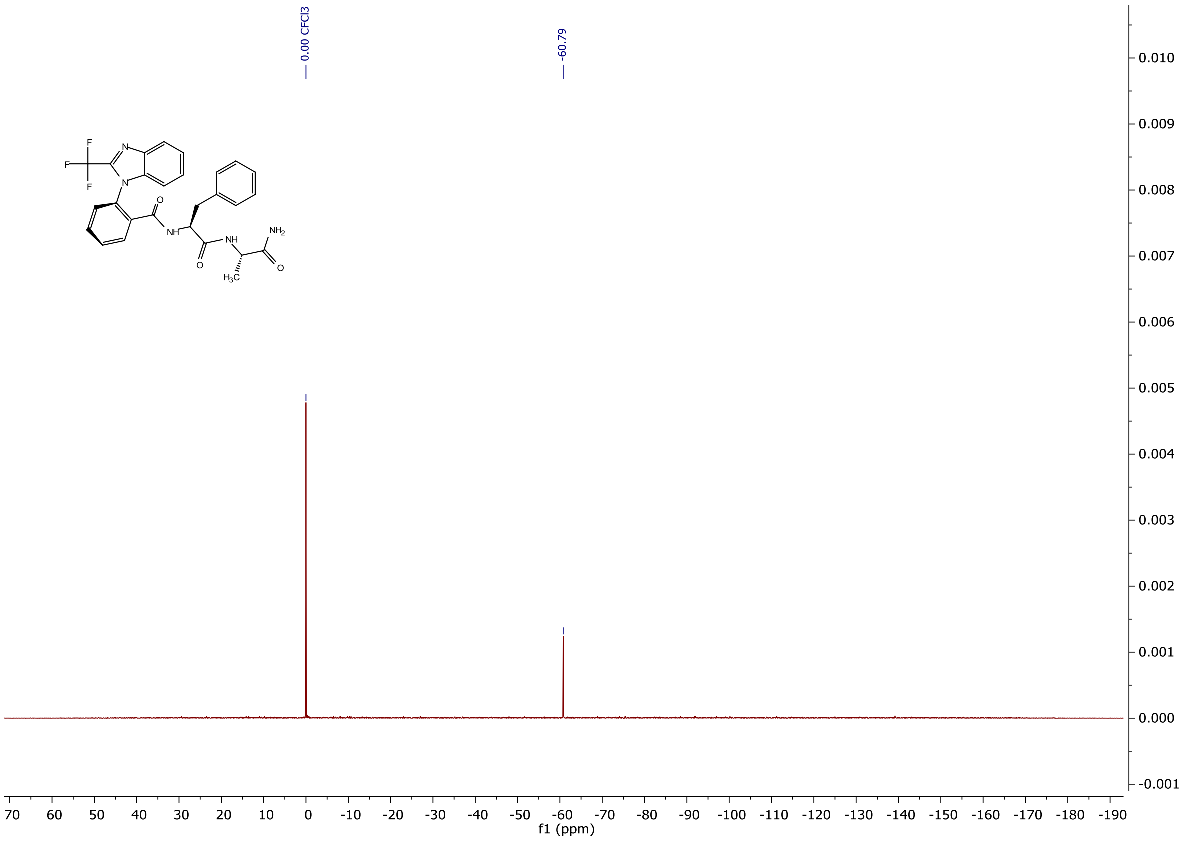

(M)-TBBA-(L)-Phe-(L)-Ala-NH2 ***(M)*-24**

^{1}H NMR (400 MHz, DMSO-d_6)

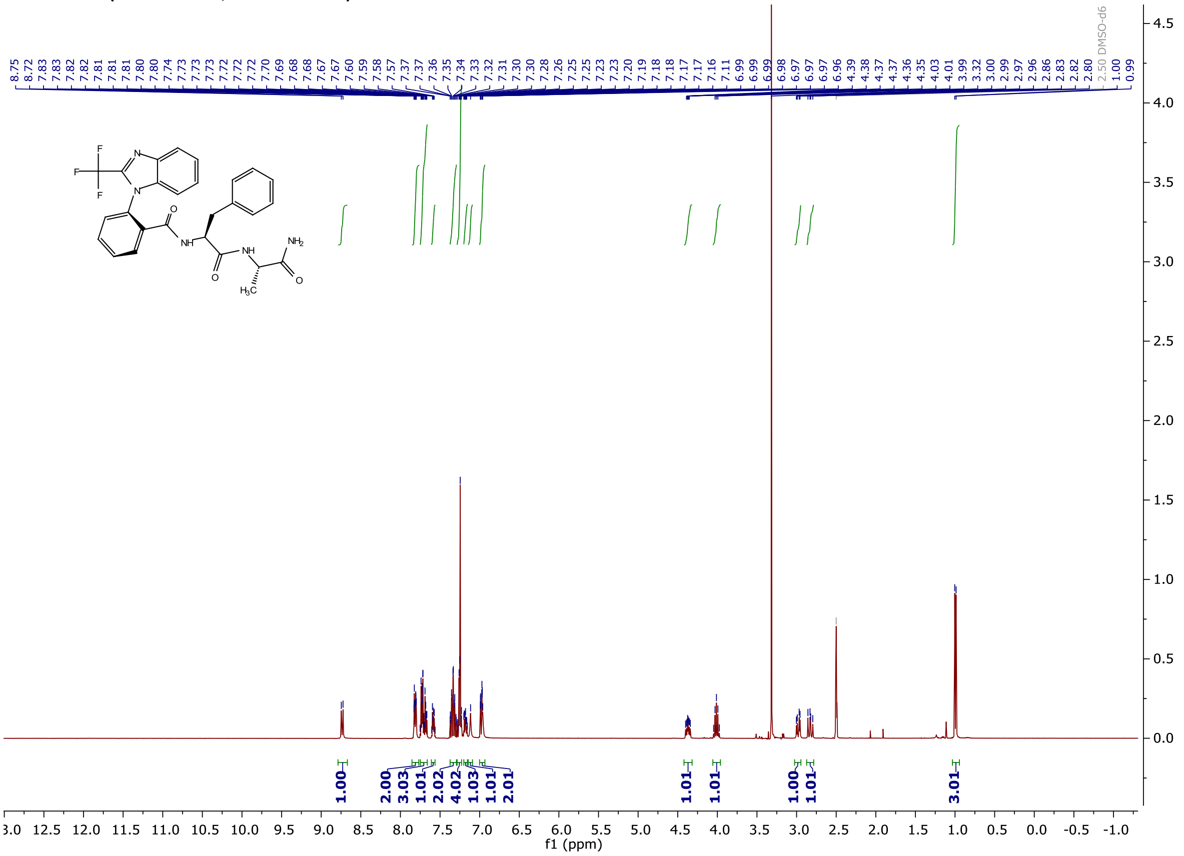

^{13}C{^{1}H} NMR (101 MHz, DMSO-d_6)

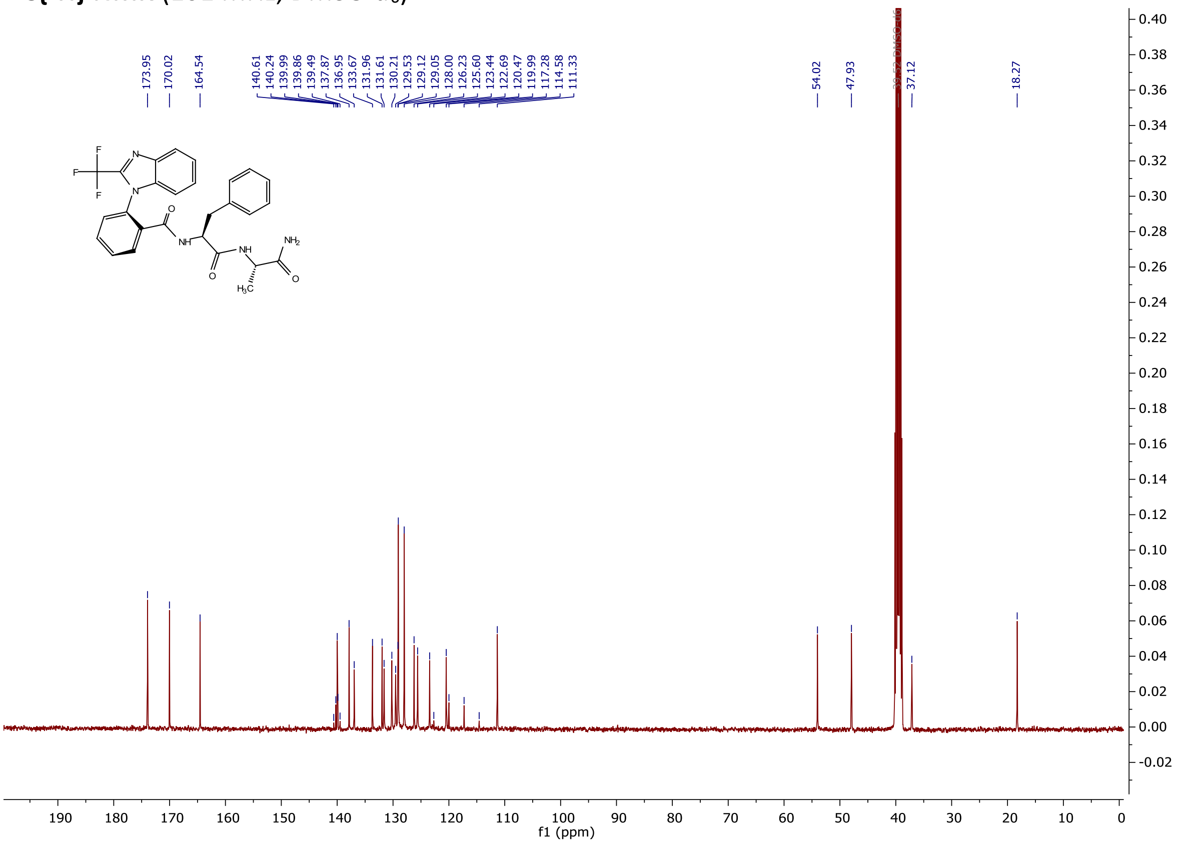

^{19}**F NMR** (376 MHz, DMSO-d_6)

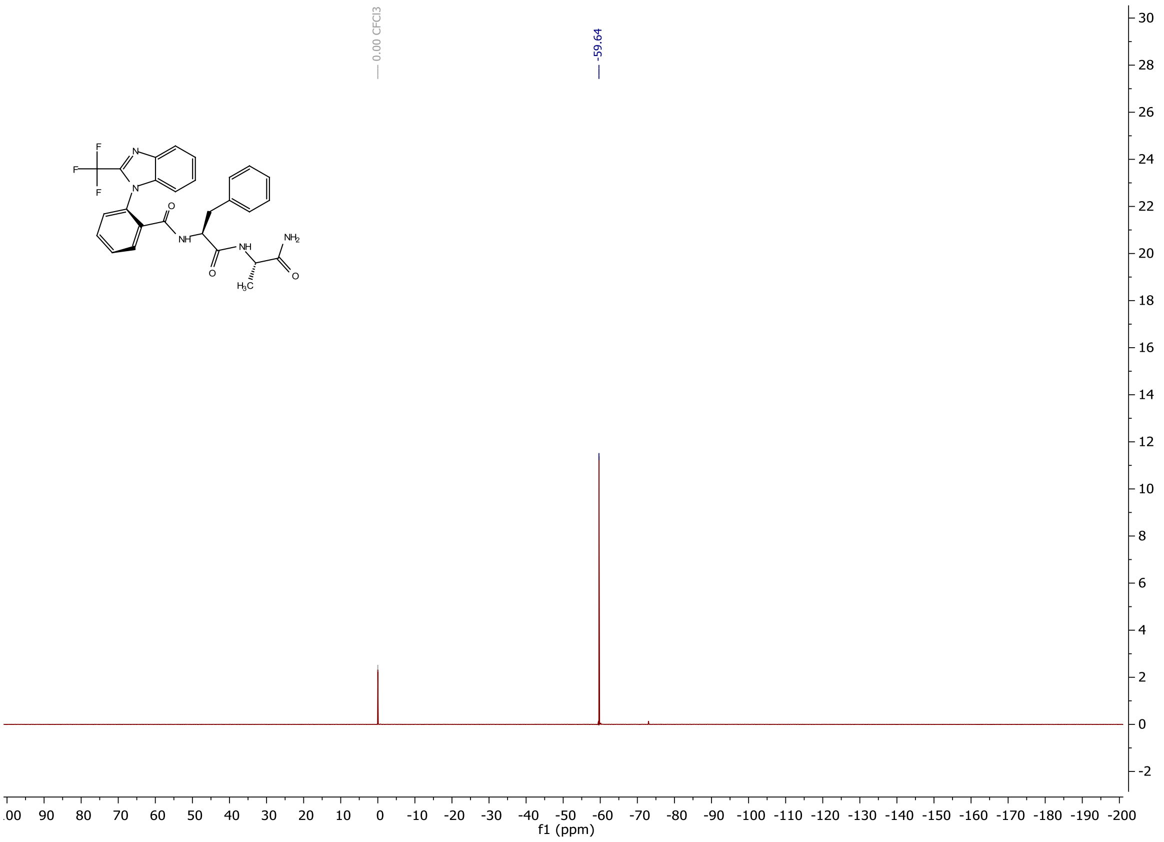

^{19}**F NMR** (76 MHz, THF)

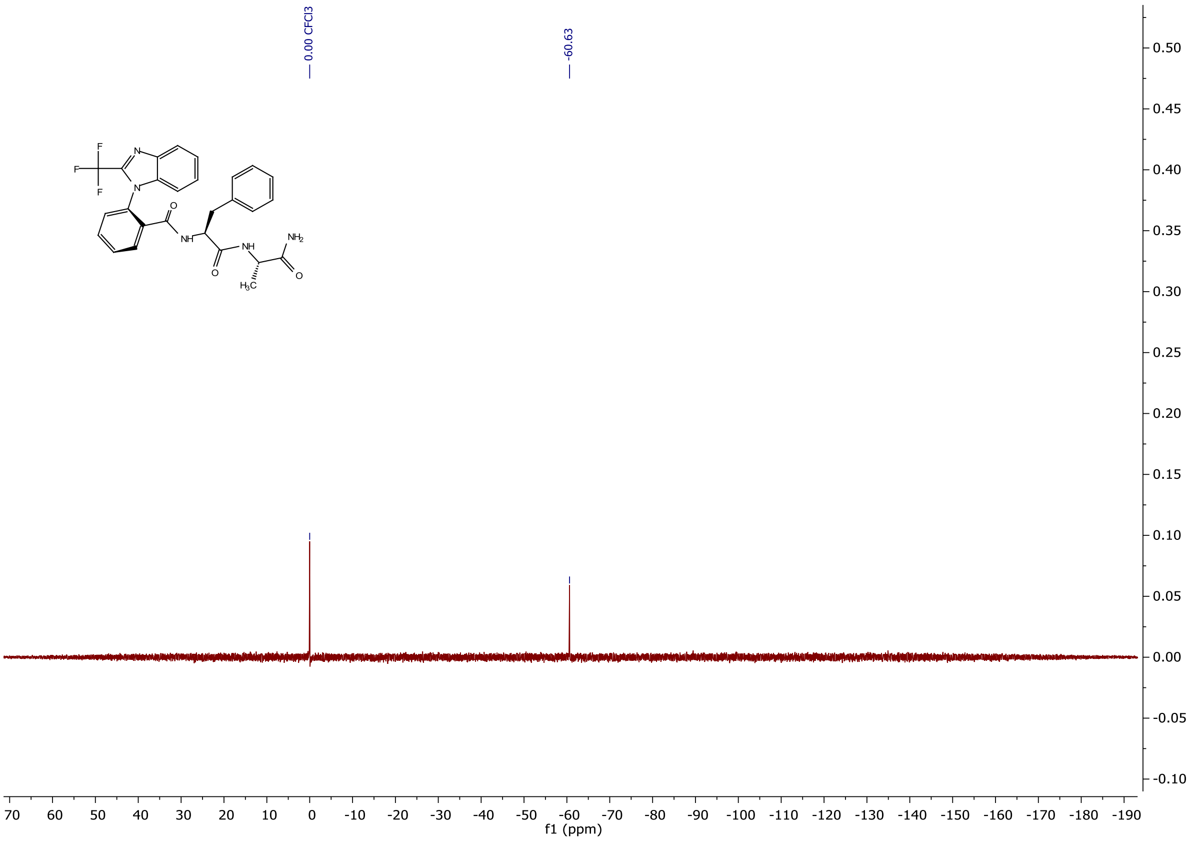

(P)-TBBA-(L)-Ser-(L)-Ala-NH_2 ***(P)*-25**

^{1}H NMR (400 MHz, DMSO-d_6)

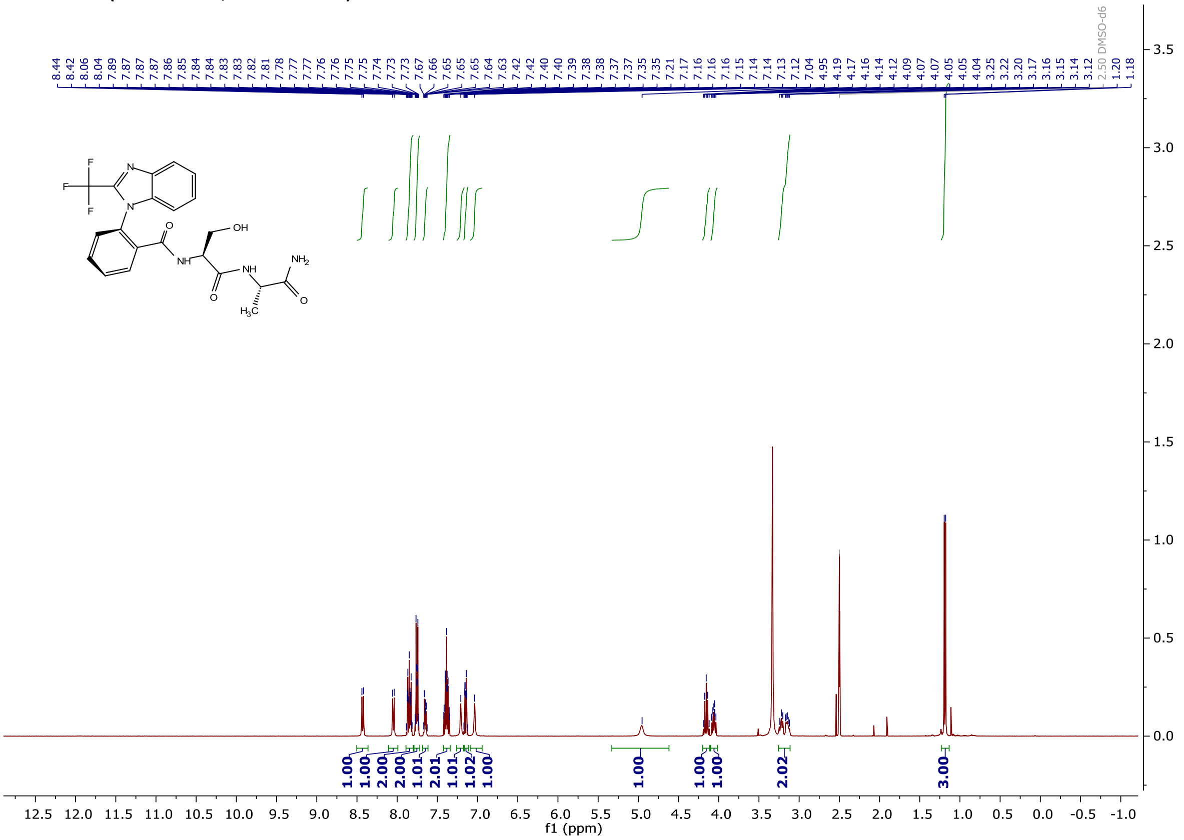

^{13}C{^{1}H} NMR (101 MHz, DMSO-d_6)

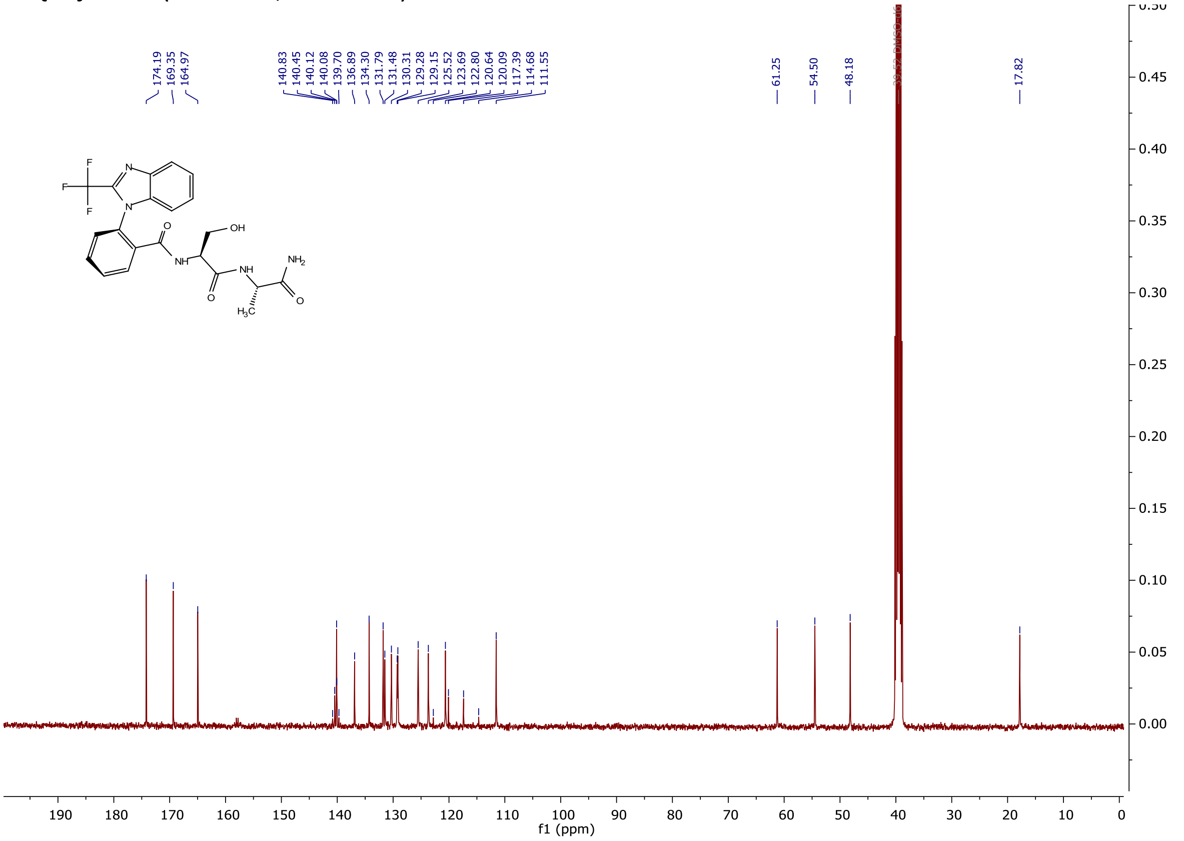

^{19}F NMR (376 MHz, DMSO-d_6)

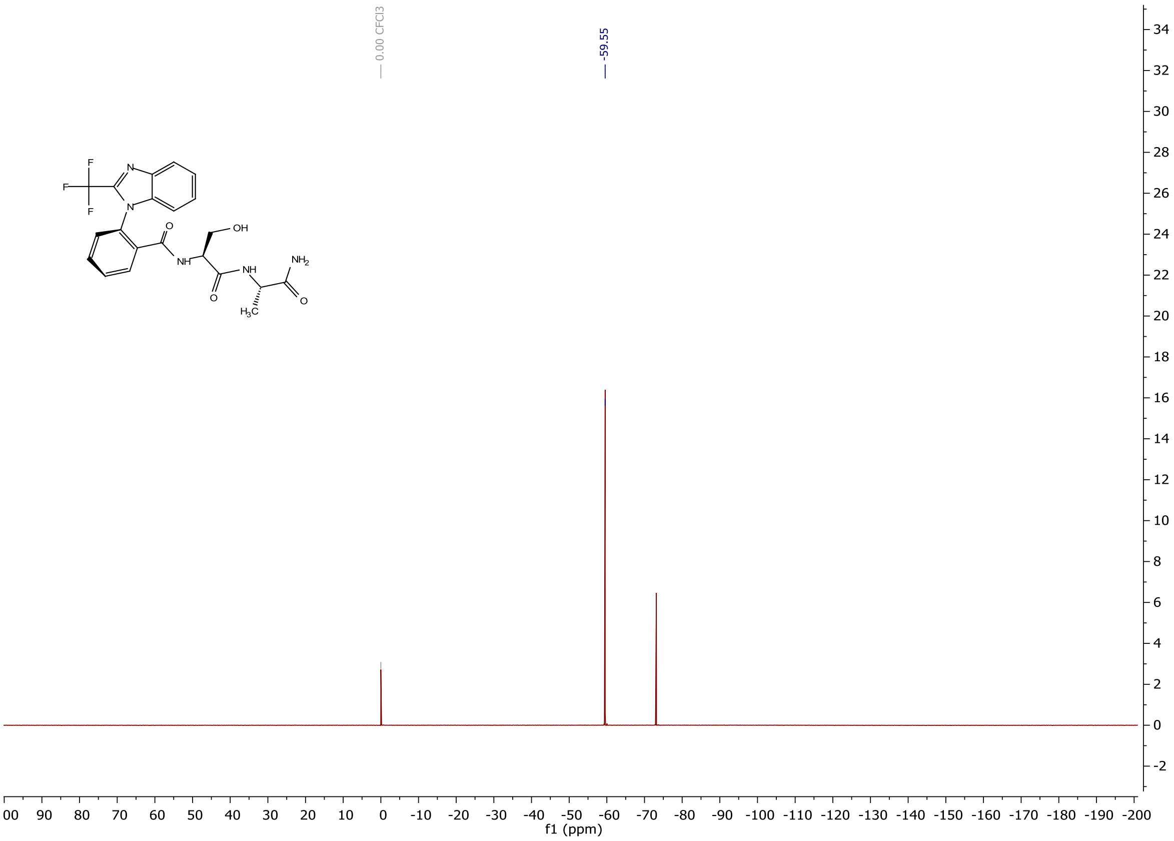

^{19}F NMR (76 MHz, THF)

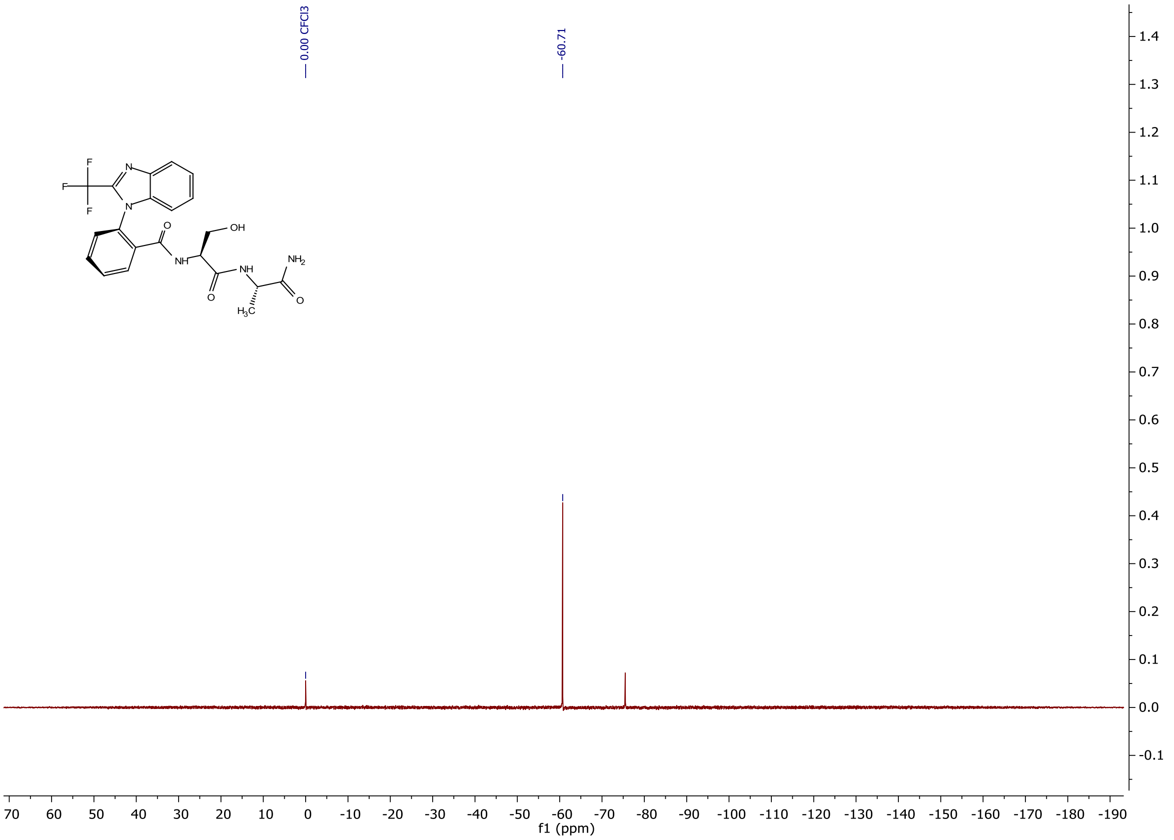

(*M*)-TBBA-(L)-Ser-(L)-Ala-NH2 **(*M*)-25**

^{1}H NMR (400 MHz, DMSO-d_6)

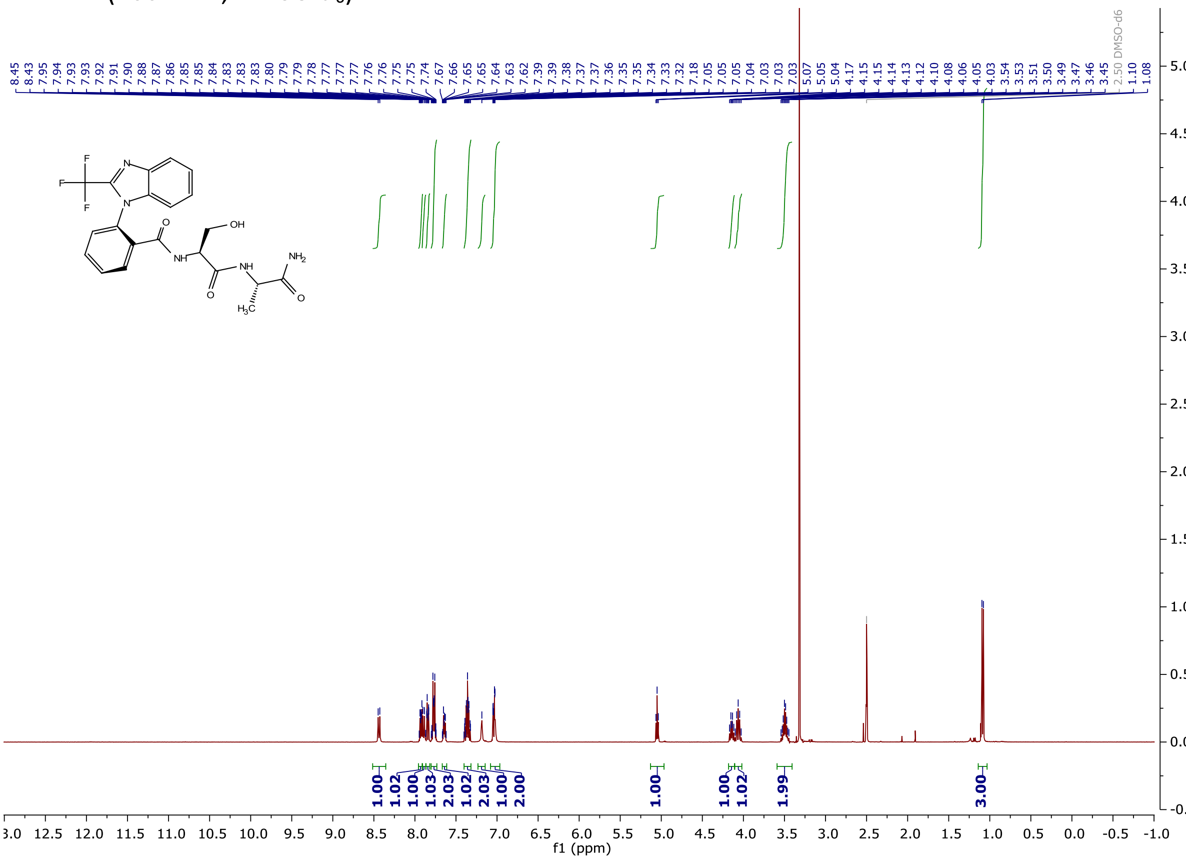

^{13}C{^{1}H} NMR (101 MHz, DMSO-d_6)

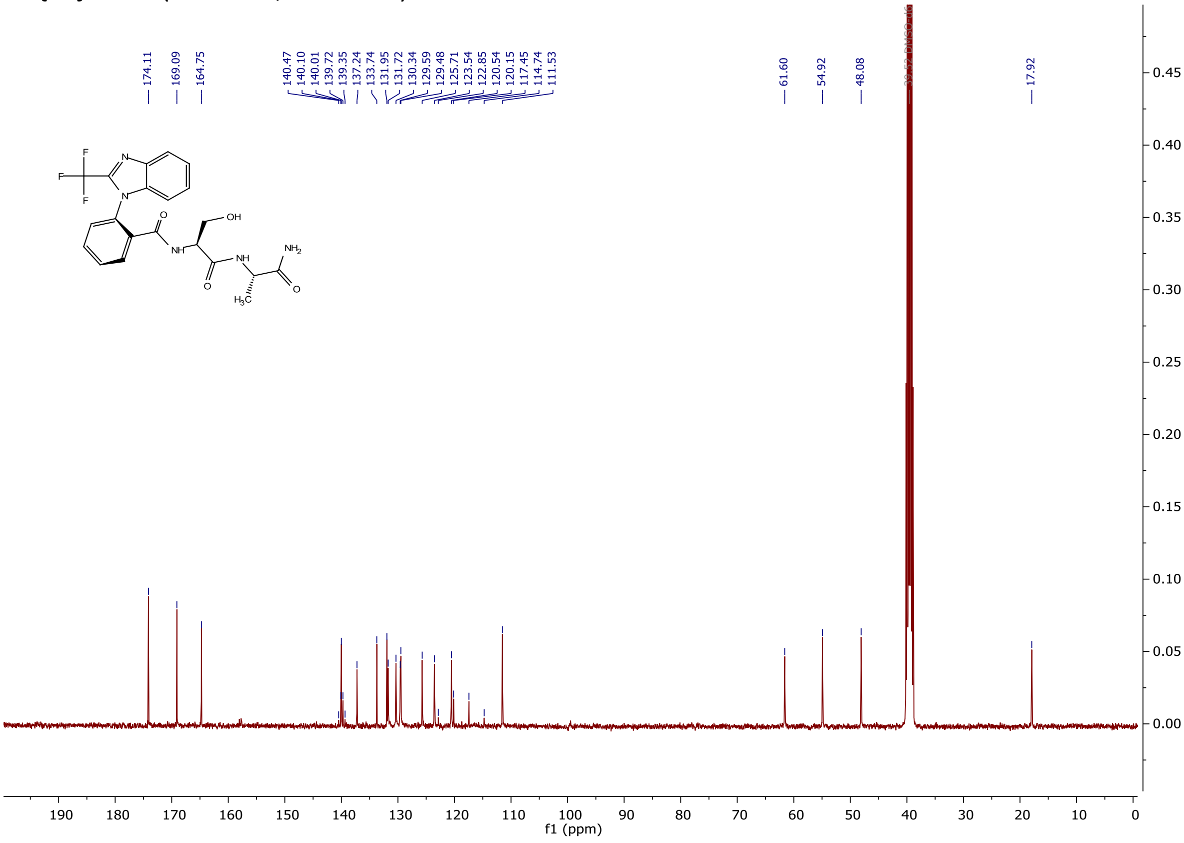

^{19}**F NMR** (376 MHz, DMSO-d_6)

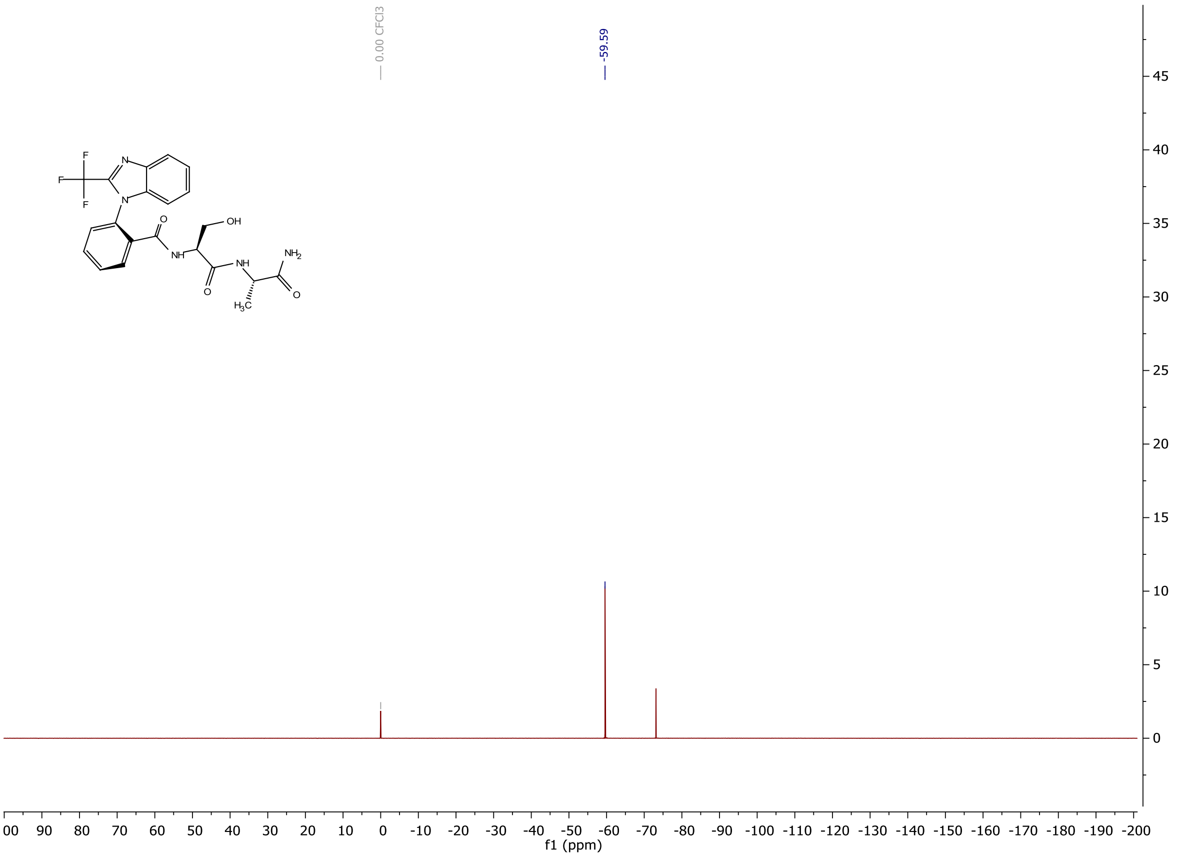

^{19}**F NMR** (76 MHz, THF)

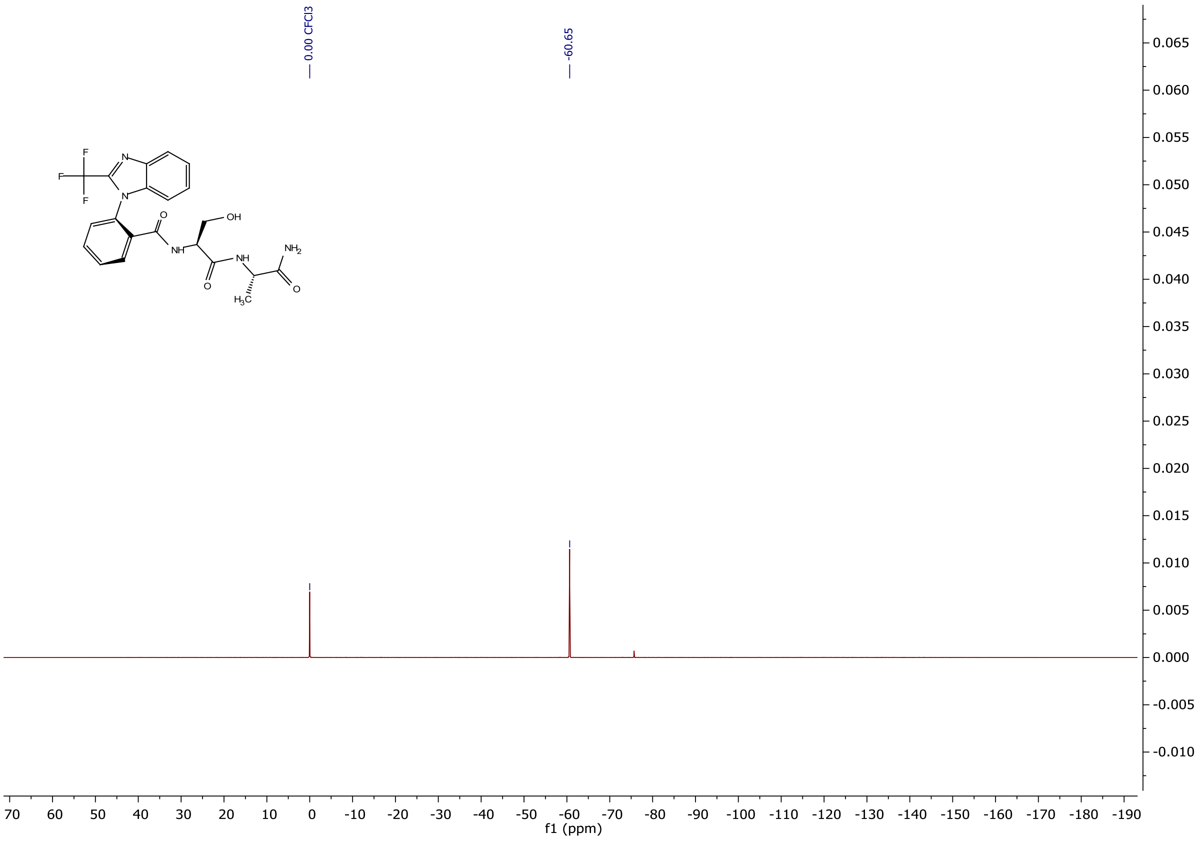

(*P*)-TBBA-(*L*)-Ala-(*L*)-Ala-(L)-Ala-NH_2 **(*P*)-26**

^{1}H NMR (400 MHz, DMSO-d_6)

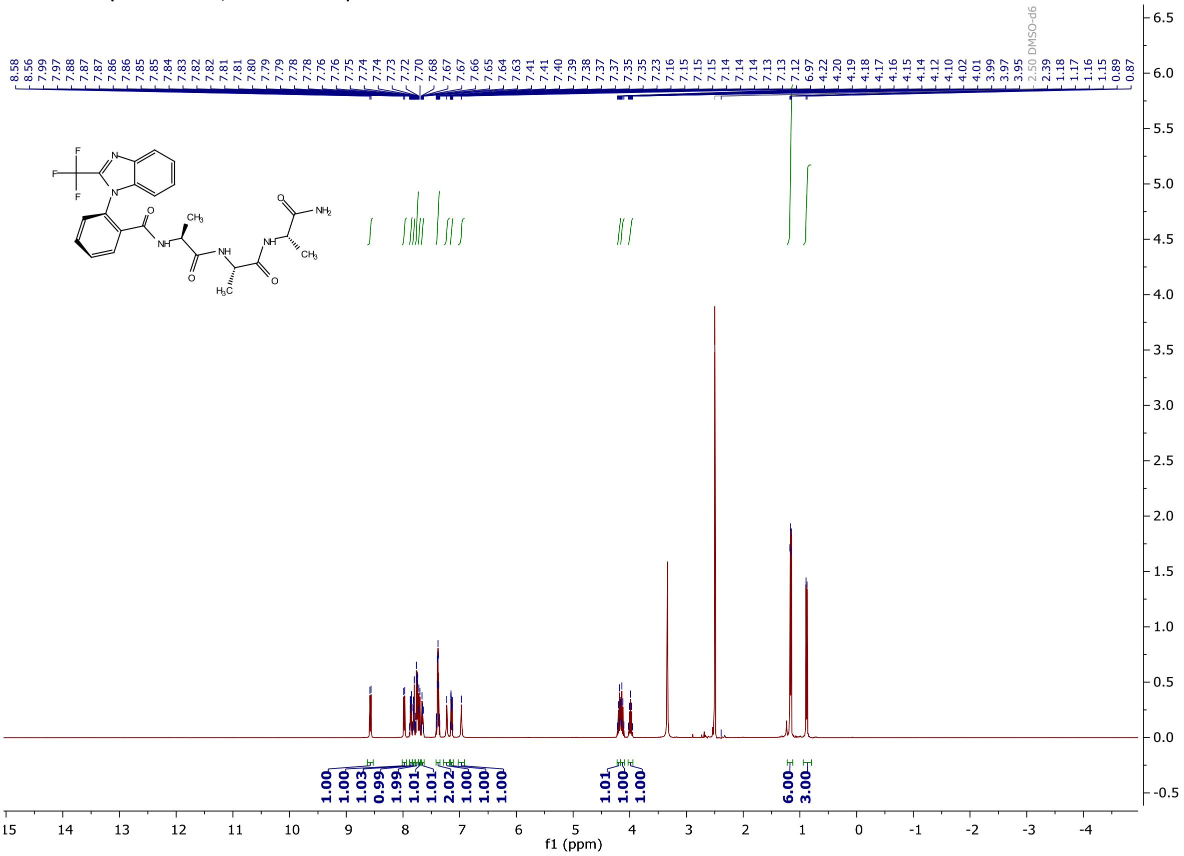

^{13}C{^{1}H} NMR (101 MHz, DMSO-d_6)

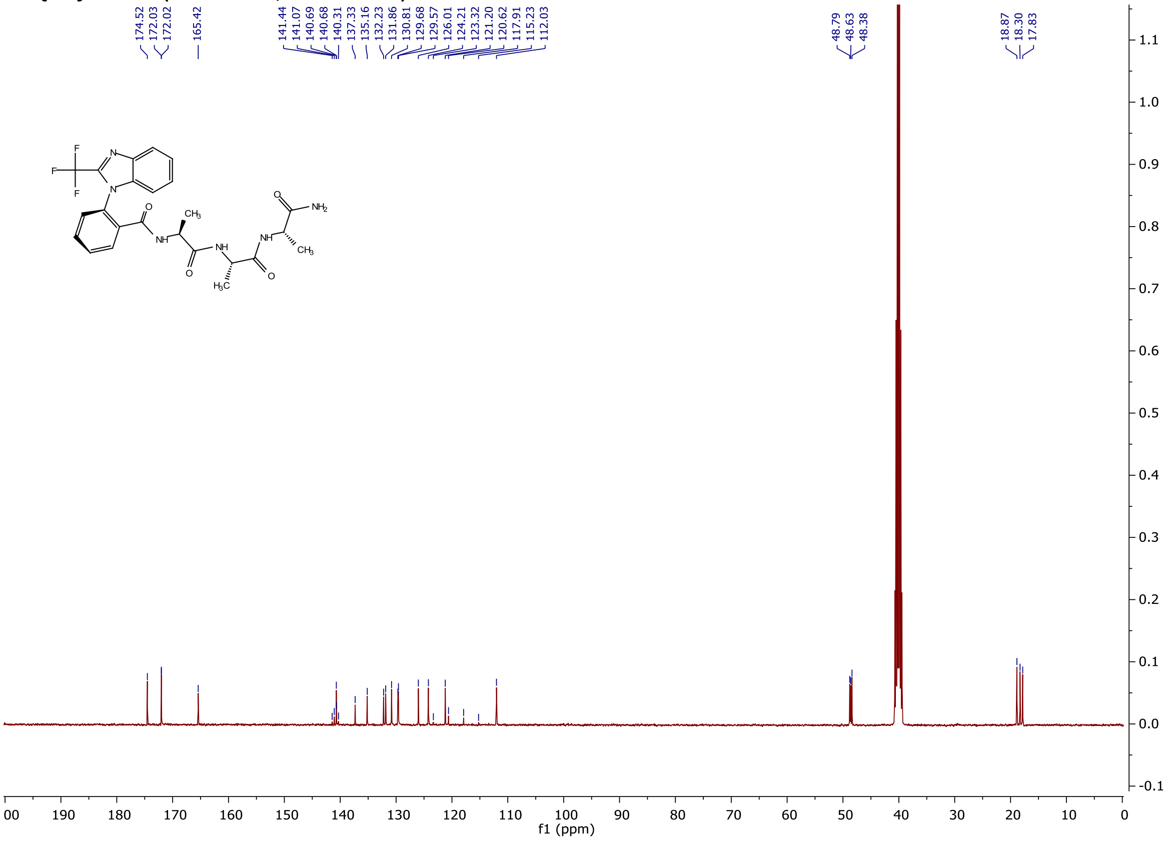

^{19}F NMR (376 MHz, DMSO-d_6)

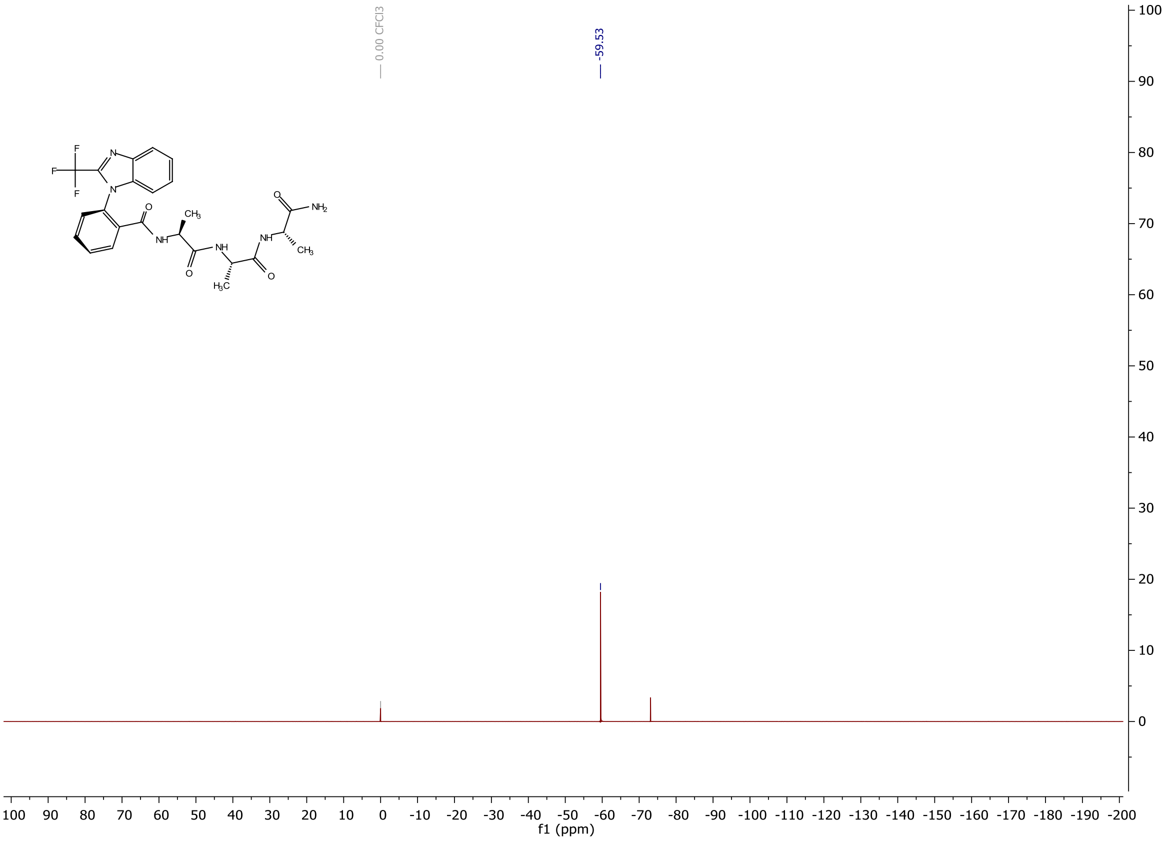

^{19}F NMR (76 MHz, THF)

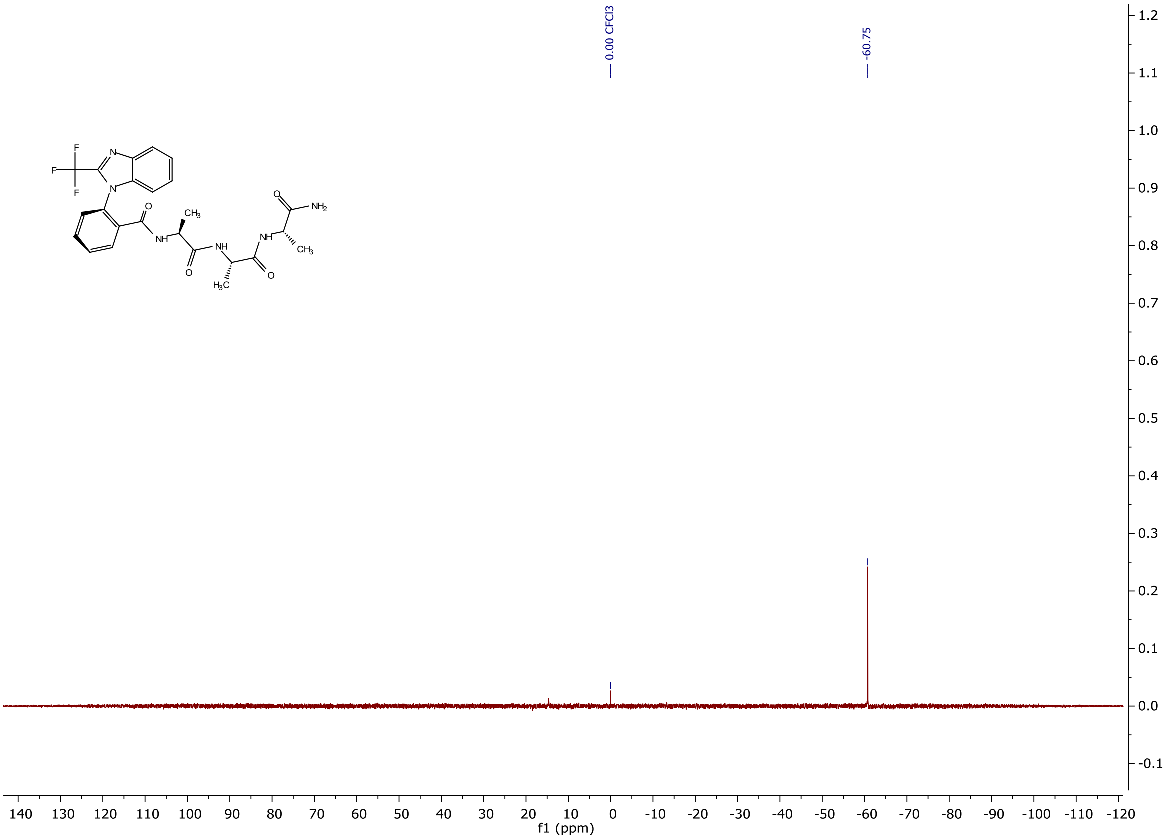

(*M*)-TBBA-(L)-Ala-(L)-Ala-(L)-Ala-NH2 **(*M*)-26**

^{1}H NMR (400 MHz, DMSO-*d*$_6$)

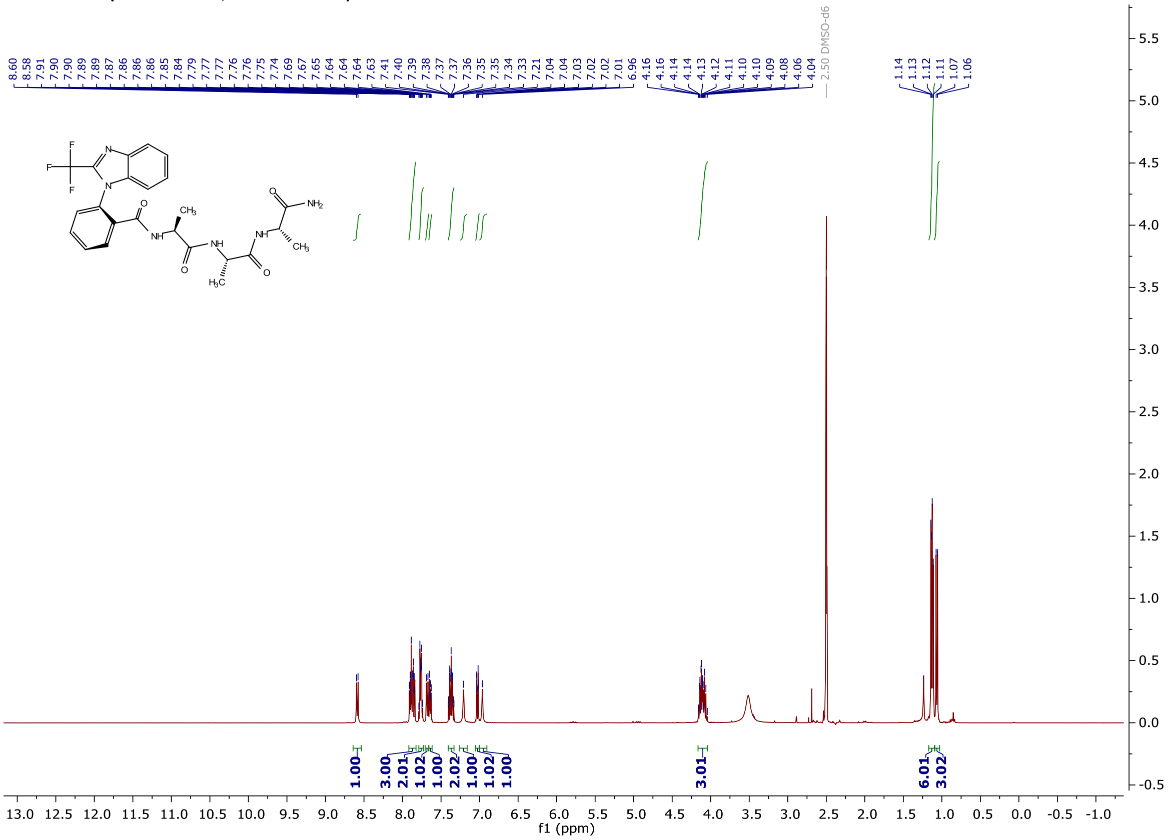

^{13}C{^{1}H} NMR (101 MHz, DMSO-*d*$_6$)

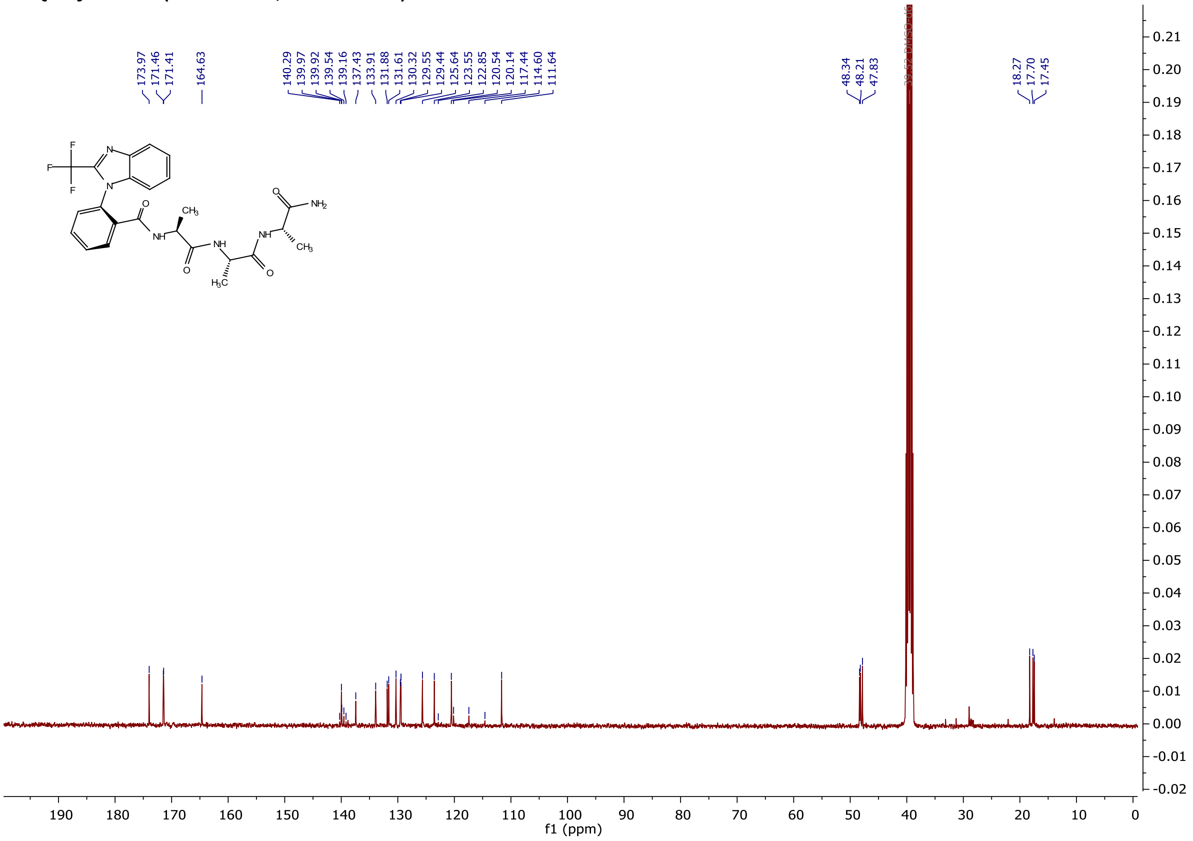

^{19}**F NMR** (376 MHz, DMSO-d_6)

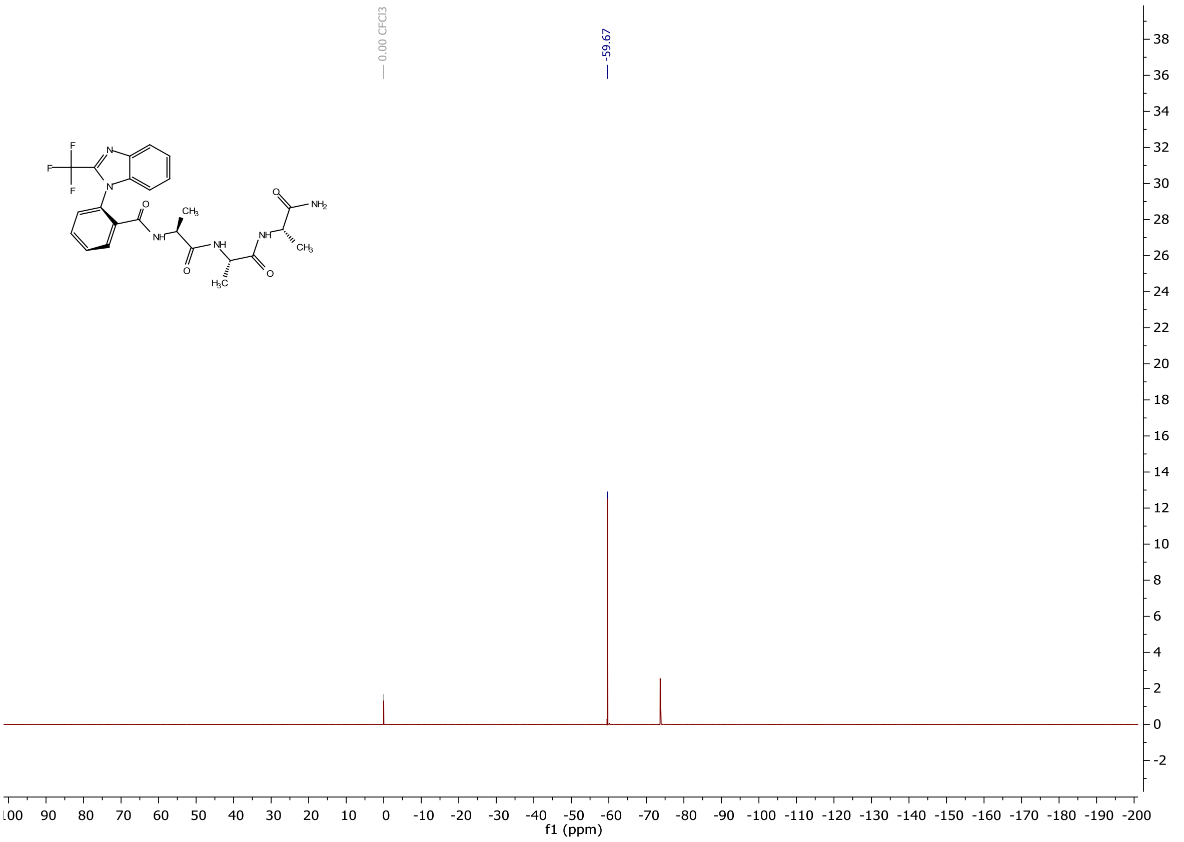

^{19}**F NMR** (76 MHz, THF)

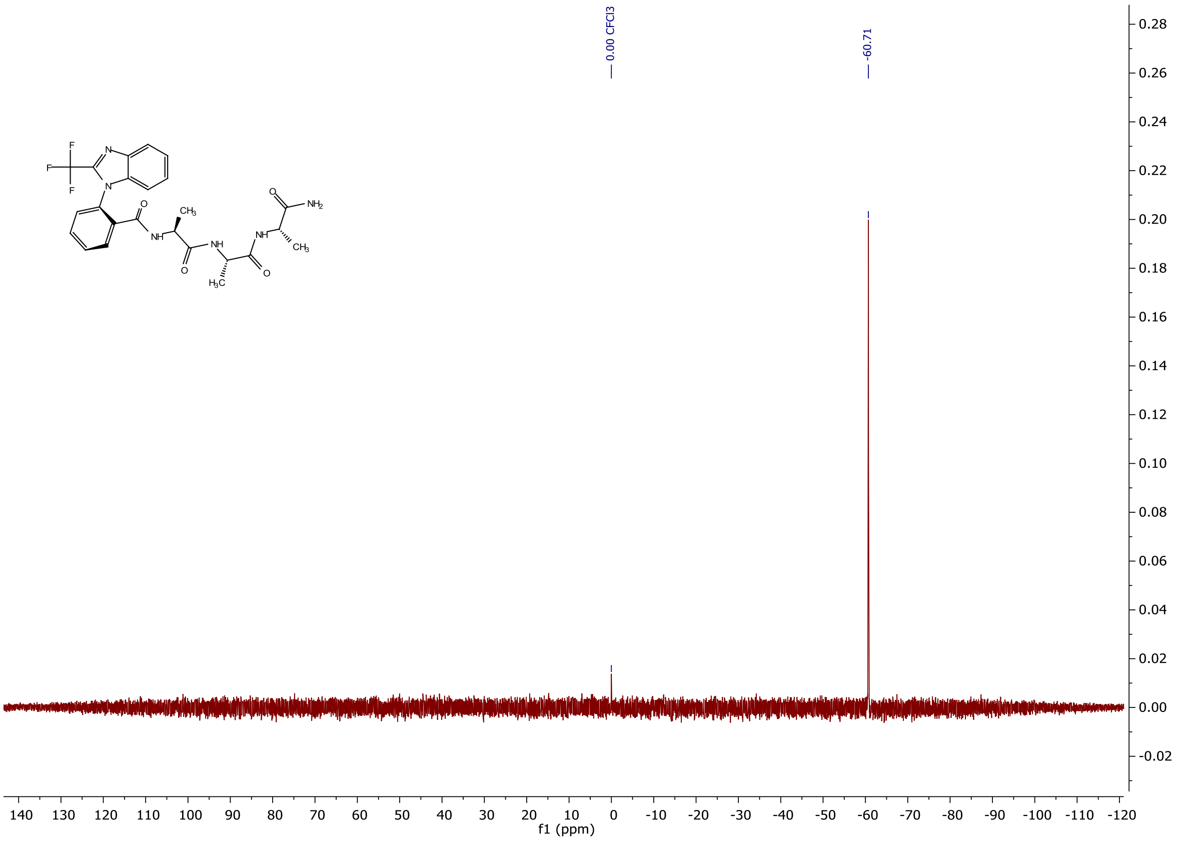

Conformational Sampling and DFT Calculations

Conformations of compound **9** were sampled using Spartan'24 software[2] with the ML-corrected MMFF force field. The fourteen and thirteen lowest-energy conformers of the (*P*)- and (*M*)-atropisomer, respectively, were then reoptimized using the def2-TZVP basis set[3] and the B97 DFT functional with the D3 empirical dispersion term and BJ-damping,[4,5] using the TurboMole 7.2 program.[6,7] During geometry optimization, solvation effects were modeled with the conductor-like screening model COSMO,[8] employing relative permittivities of 4.81 (chloroform) and 46.7 (DMSO). Final solvation energies were calculated for the optimized geometries using the more advanced COSMO-RS model ("RS" stands for *Real Solvent*), originally developed by Klamt,[9] using its open-source OpenCOSMO-RS 24a implementation[10,11] in ORCA software package,[12,13,14] at the BP86/def2-TZVPD level of theory.

We note that the chosen method represents a compromise between accurately describing intramolecular conformational energies and solute–solvent interactions. The continuum solvent model used in the QM calculations tends to underestimate hydrogen-bonding energies with solvent molecules, although COSMO-RS improves on classical COSMO in this respect. While explicit-solvent molecular dynamics with an empirical force field may better capture solvation, available force fields often describe intramolecular conformational energetics poorly. QM is generally more reliable for this purpose. We therefore optimized and ranked conformers using QM rather than relying on force-field energetics. Nevertheless, the balance between intramolecular hydrogen bonding (treated explicitly in QM) and hydrogen bonding to the solvent (treated implicitly) is likely biased toward intramolecular hydrogen bonds. This may particularly favor more compact conformers in DMSO.

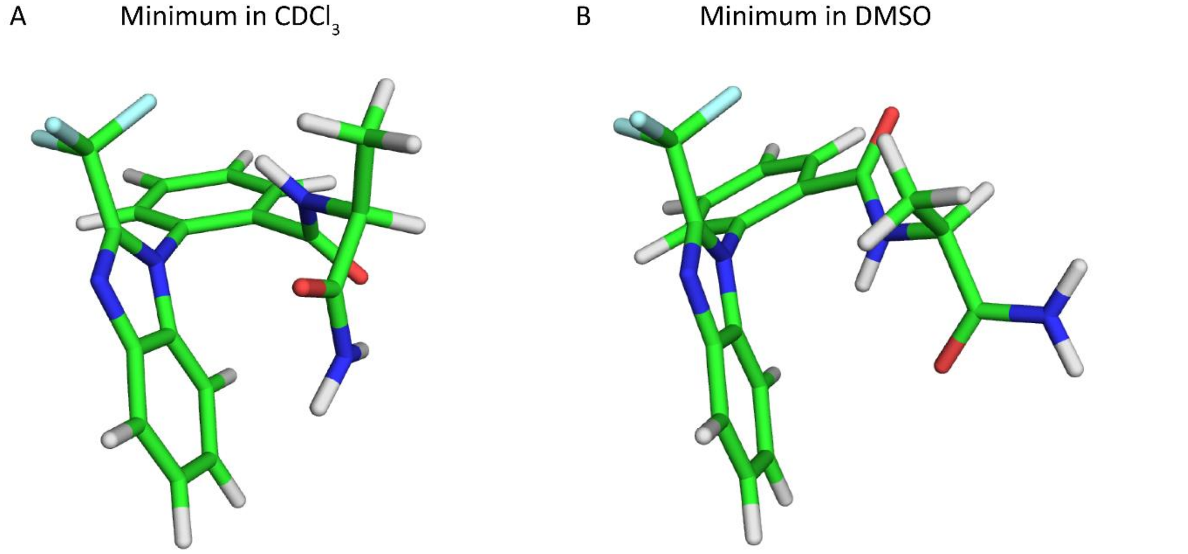

Figure S1. Most stable conformations of the (*P*)-atropisomer of compound **9** in $CDCl_3$ (A) and DMSO (B) solvents. In $CDCl_3$, the amide NH group is oriented toward the CF_3 group, whereas in DMSO, the amide carbonyl oxygen is positioned closer to the CF_3 group.

Optimized geometries of the (*P*)- and (*M*)-atropisomer of compound **9** in XYZ format

Ordering:
(*P*)-atropisomer in CDCl3 (14 structures)
(*P*)-atropisomer in DMSO (14 structures)
(*M*)-atropisomer in CDCl3 (13 structures)
(*M*)-atropisomer in DMSO (13 structures)

Comment line contains total absolute energy of the DFT calculation + COSMO-RS solvation (in Eh units), number of imaginary frequencies in COSMO and number of imaginary frequencies in vacuum. Frequencies were calculated only for the most stable conformers (according to COSMO ranking).

Note that whereas no imaginary frequences were found in vacuum optimizations, they were sometimes found in COSMO calculations. This is likely a consequence of numerical errors on the very flat COSMO surfaces.

(*P*)-atropisomer in CDCl3 (14 structures):

42
Energy+COSMO-RS = -1363.75820 ; # imag. freq. COSMO = 0 ; # imag. freq. vacuum = 0
C 1.5699574 0.3456725 3.6604456
C 1.6847042 1.0681280 2.3283446
C 2.7626656 0.4336407 1.4077677
N 3.1044281 1.2122438 0.3545082
O 3.2473227 -0.6725392 1.6263747
N 0.3934346 1.0468040 1.6336880
C -0.0111154 1.9888930 0.7535066
C -1.3560341 1.7497766 0.1209192
C -1.6417024 0.5972828 -0.6288583
N -0.6168106 -0.3809751 -0.8427105
C -0.4766277 -1.6521175 -0.3013996
C -1.4990479 -2.2614937 0.6196050
F -2.6771075 -2.5117987 -0.0082303
F -1.0607453 -3.4118569 1.1457297
F -1.7894858 -1.4144238 1.6600044
N 0.6363251 -2.2535148 -0.6530800
C 1.2971942 -1.3501661 -1.4742593
C 0.5270539 -0.1673404 -1.6071188
C 0.9383041 0.9203437 -2.3774299
C 2.1677374 0.7919589 -3.0179090

C	2.9540502	-0.3746948	-2.8903762
C	2.5344162	-1.4566464	-2.1229458
C	-2.8851963	0.4310277	-1.2382038
C	-3.8688009	1.4053951	-1.0783544
C	-3.5952054	2.5589027	-0.3404127
C	-2.3415082	2.7353335	0.2414163
O	0.6556611	2.9934226	0.4763986
H	0.8114663	0.8267834	4.2837552
H	1.3032855	-0.7047681	3.5118783
H	2.5278784	0.3734382	4.1838675
H	1.9441754	2.1200820	2.4805154
H	2.5674543	2.0465682	0.1525170
H	3.6454595	0.8003292	-0.3926853
H	-0.2244645	0.2731146	1.8290351
H	0.3421251	1.8220358	-2.4622613
H	2.5343122	1.6129980	-3.6264810
H	3.9083583	-0.4245590	-3.4059564
H	3.1378321	-2.3526095	-2.0206260
H	-3.0681206	-0.4524073	-1.8396492
H	-4.8396098	1.2704562	-1.5446153
H	-4.3561085	3.3243535	-0.2237925
H	-2.1167185	3.6356464	0.8042678

42

Energy+COSMO-RS = -1363.75585 ; # imag. freq. COSMO = 0 ; # imag. freq. vacuum = 0

C	1.2796810	2.7585020	-1.2589102
C	1.0898430	2.6453844	0.2459481
C	0.5593919	3.9615128	0.8759845
N	0.6547860	3.9900479	2.2242897
O	0.0986822	4.8798636	0.2007752
N	0.1690204	1.5478760	0.5481450
C	0.2118150	0.8136701	1.6825675
C	-0.7623341	-0.3305980	1.7856917
C	-1.0571547	-1.2325314	0.7478395
N	-0.4516036	-1.1017091	-0.5463021
C	0.7917826	-1.5309791	-0.9839812
C	1.7191626	-2.3385884	-0.1119469
F	1.1104113	-3.4634600	0.3523208
F	2.8177254	-2.7118693	-0.7863467
F	2.1208784	-1.6321077	0.9788474
N	1.0654926	-1.1761695	-2.2185662

C -0.0438712 -0.4589418 -2.6481633
C -1.0121137 -0.4079368 -1.6133623
C -2.2189406 0.2822488 -1.7378006
C -2.4367335 0.9277465 -2.9541573
C -1.4885836 0.8814548 -3.9979480
C -0.2872148 0.1915807 -3.8640536
C -1.9532210 -2.2835679 0.9429939
C -2.5719848 -2.4515522 2.1795347
C -2.2792035 -1.5737784 3.2251269
C -1.3753875 -0.5333683 3.0296925
O 0.9889666 1.0619519 2.6138634
H 1.6973477 1.8287401 -1.6524270
H 0.3291304 2.9618350 -1.7604403
H 1.9616409 3.5796065 -1.4891762
H 2.0378807 2.3995093 0.7335025
H 0.9434506 3.1580198 2.7271755
H 0.2453967 4.7605295 2.7321354
H -0.5764023 1.3874436 -0.1148750
H -2.9480306 0.3141605 -0.9346078
H -3.3595278 1.4803616 -3.1009519
H -1.7035414 1.4020674 -4.9260469
H 0.4459748 0.1639188 -4.6637046
H -2.1468125 -2.9674860 0.1232732
H -3.2700063 -3.2696077 2.3267032
H -2.7517189 -1.7025228 4.1939508
H -1.1312170 0.1441016 3.8409026

42
Energy+COSMO-RS = -1363.75493 ; # imag. freq. COSMO = 0 ; # imag. freq. vacuum = 0
C 1.3198505 -0.5102328 -2.6680575
C 0.9012599 0.8951336 -2.2292546
C -0.0131457 1.5270640 -3.3002103
N 0.5804899 2.4552428 -4.0829796
O -1.1776694 1.1587234 -3.4424507
N 0.2057413 0.8273356 -0.9563030
C 0.1456692 1.8794249 -0.1035049
C -0.6273673 1.6671631 1.1742034
C -0.5997209 0.4945121 1.9500088
N 0.1606555 -0.6520502 1.5409157
C 1.5167909 -0.9046713 1.6744230
C 2.4380591 0.0130962 2.4367031

F 1.9789251 0.2434831 3.6967446
F 3.6710062 -0.5092400 2.5359198
F 2.5497319 1.2266642 1.8325896
N 1.9057182 -2.0123988 1.0856338
C 0.7591682 -2.5427721 0.5089538
C -0.3499860 -1.7050973 0.7894194
C -1.6331643 -1.9579368 0.3023609
C -1.7808670 -3.1020761 -0.4799917
C -0.6915043 -3.9541005 -0.7602057
C 0.5857944 -3.6911022 -0.2736534
C -1.3231814 0.4022541 3.1394040
C -2.0923525 1.4793427 3.5727356
C -2.1220825 2.6553703 2.8205056
C -1.3865896 2.7480452 1.6425546
O 0.6891037 2.9616979 -0.3370254
H 1.9847009 -0.9531541 -1.9223522
H 0.4408063 -1.1516027 -2.7845961
H 1.8434683 -0.4665259 -3.6267439
H 1.7824898 1.5225363 -2.0792702
H 1.4919481 2.8235232 -3.8620444
H 0.0414479 2.9169922 -4.8011583
H -0.4281043 0.0525439 -0.8246649
H -2.4696126 -1.3008952 0.5180051
H -2.7592954 -3.3418453 -0.8849431
H -0.8568641 -4.8331774 -1.3756506
H 1.4258228 -4.3407688 -0.4975572
H -1.2667343 -0.5134315 3.7188438
H -2.6566659 1.4016455 4.4966558
H -2.7149537 3.5012873 3.1540857
H -1.3911081 3.6626880 1.0594765

42
Energy+COSMO-RS = -1363.75451 ; # imag. freq. COSMO = 0 ; # imag. freq. vacuum = 0
C 2.2147176 1.2902114 -1.9785850
C 0.8250796 1.9473121 -2.0631734
C 0.1242024 1.5811412 -3.3809703
N 0.6257751 2.1861741 -4.4793364
O -0.8030774 0.7724405 -3.4173416
N -0.0316245 1.5079071 -0.9781038
C -0.0125573 2.0771763 0.2469533
C -0.9164484 1.4665726 1.2901328

C -1.0504288 0.0874575 1.5241693
N -0.3636417 -0.8752145 0.7123798
C 0.9547642 -1.2986077 0.7653478
C 1.9016228 -0.8633899 1.8537154
F 1.3946323 -1.1377609 3.0877579
F 3.0851335 -1.4895406 1.7466004
F 2.1379907 0.4750389 1.8192318
N 1.2744648 -2.1411871 -0.1911240
C 0.1160893 -2.3006035 -0.9413119
C -0.9282188 -1.5211085 -0.3829470
C -2.2113611 -1.4710539 -0.9277485
C -2.4270868 -2.2429148 -2.0664280
C -1.4018818 -3.0270438 -2.6360035
C -0.1234811 -3.0680618 -2.0885014
C -1.8711719 -0.3922235 2.5459324
C -2.5771001 0.4997325 3.3491462
C -2.4456653 1.8740514 3.1378270
C -1.6133641 2.3479087 2.1278364
O 0.6883268 3.0546203 0.5256241
H 2.6737307 1.5384109 -1.0191653
H 2.1242840 0.2031206 -2.0564175
H 2.8667223 1.6494653 -2.7794643
H 0.9350714 3.0344219 -1.9980161
H 1.3739969 2.8583325 -4.4244541
H 0.2524168 1.9561363 -5.3880298
H -0.7104227 0.7973477 -1.2247993
H -2.9965568 -0.8624488 -0.4913919
H -3.4083009 -2.2363000 -2.5310280
H -1.6189604 -3.6086112 -3.5269074
H 0.6662960 -3.6686458 -2.5286850
H -1.9395294 -1.4633764 2.7052206
H -3.2167657 0.1232237 4.1412092
H -2.9874611 2.5762589 3.7640284
H -1.4904410 3.4140993 1.9694953

42

Energy+COSMO-RS = -1363.75416 ; # imag. freq. COSMO = 1 ; # imag. freq. vacuum = 0

C 0.0282225 4.9299404 -0.4955844
C 0.6054420 3.5454934 -0.7997461
C 1.8494547 3.2966215 0.0793184
N 1.6773146 2.5036567 1.1579310

O	2.9099282	3.8560949	-0.1936998
N	-0.4357693	2.5337238	-0.6613036
C	-0.3170893	1.2928756	-1.2080786
C	-1.5459348	0.4292722	-1.1025868
C	-1.4782034	-0.8641410	-0.5622640
N	-0.2517049	-1.3532662	-0.0058743
C	0.2079915	-1.2487933	1.2979224
C	-0.5993566	-0.6080199	2.3926955
F	-1.7651660	-1.2683034	2.6191335
F	0.0828305	-0.5659086	3.5458365
F	-0.9489095	0.6817068	2.0774524
N	1.3813181	-1.8089855	1.4890825
C	1.7419072	-2.3267423	0.2516710
C	0.7269406	-2.0542228	-0.6998309
C	0.8135028	-2.4534993	-2.0326963
C	1.9626056	-3.1503437	-2.3938528
C	2.9861233	-3.4313596	-1.4612261
C	2.8941946	-3.0260274	-0.1343929
C	-2.5998431	-1.6899479	-0.5407598
C	-3.8108901	-1.2313600	-1.0596470
C	-3.8963095	0.0560588	-1.5893585
C	-2.7717885	0.8816631	-1.6043237
O	0.7070942	0.9054505	-1.7660172
H	-0.7834137	5.1630630	-1.1906596
H	-0.3575284	4.9743157	0.5286111
H	0.8100146	5.6838900	-0.6035099
H	0.9758874	3.5137960	-1.8281543
H	0.8070418	2.0263245	1.3232710
H	2.4638053	2.3042562	1.7573913
H	-1.3033801	2.7874278	-0.2119976
H	0.0347108	-2.2209605	-2.7505631
H	2.0793495	-3.4815910	-3.4214017
H	3.8653909	-3.9749678	-1.7937369
H	3.6805859	-3.2380543	0.5831010
H	-2.5173156	-2.6814599	-0.1081370
H	-4.6828084	-1.8775380	-1.0440463
H	-4.8360982	0.4184906	-1.9943842
H	-2.8362893	1.8783718	-2.0304653

42

Energy+COSMO-RS = -1363.75317

C	2.4328131	0.9230965	-1.8893378
C	1.1684602	1.7807025	-2.0385039
C	0.4869349	1.5893958	-3.4032990
N	0.0069487	0.3448350	-3.6476296
O	0.3808989	2.5142727	-4.2050010
N	0.2025747	1.5038399	-0.9716259
C	0.3070282	2.0466413	0.2670829
C	-0.7087103	1.5878700	1.2857855
C	-1.0647294	0.2463209	1.5070365
N	-0.5158172	-0.8075117	0.6999096
C	0.7136885	-1.4403455	0.7916276
C	1.6667563	-1.1959708	1.9357657
F	1.0721621	-1.4550456	3.1318073
F	2.7522983	-1.9787143	1.8351470
F	2.0861533	0.0947421	1.9760939
N	0.9427247	-2.2898258	-0.1840031
C	-0.1861300	-2.2322627	-0.9914808
C	-1.1195802	-1.3131959	-0.4458228
C	-2.3439457	-1.0281302	-1.0517274
C	-2.6186507	-1.7060564	-2.2380326
C	-1.7065021	-2.6280354	-2.7933702
C	-0.4844899	-2.9044913	-2.1848593
C	-1.9711805	-0.1032573	2.5081435
C	-2.5418890	0.8866647	3.3046518
C	-2.1902273	2.2235696	3.1062726
C	-1.2733097	2.5650693	2.1159536
O	1.1657077	2.8760181	0.5681382
H	2.9239333	1.1616245	-0.9433034
H	2.1945863	-0.1446465	-1.8937987
H	3.1283465	1.1313894	-2.7074460
H	1.4367719	2.8363963	-1.9794215
H	0.1983339	-0.4445428	-3.0491628
H	-0.4291548	0.1559769	-4.5378810
H	-0.5898018	0.9164163	-1.1845244
H	-3.0438607	-0.3179953	-0.6236345
H	-3.5577638	-1.5175429	-2.7485995
H	-1.9651705	-3.1303971	-3.7203181
H	0.2184278	-3.6116943	-2.6133554
H	-2.2117158	-1.1505235	2.6585066
H	-3.2486821	0.6131126	4.0816215
H	-2.6259566	2.9996938	3.7278367

H -0.9799653 3.5990853 1.9685433

42

Energy+COSMO-RS = -1363.75272 ; # imag. freq. COSMO = 0 ; # imag. freq. vacuum = 0

C -0.5705614 -2.4734159 -3.5028720
C -0.4646819 -2.2624683 -1.9875805
C 0.9239506 -2.6918452 -1.4555570
N 1.9325805 -1.8203672 -1.6594195
O 1.0620740 -3.7866812 -0.9102849
N -0.8691412 -0.9180546 -1.5515634
C -0.3696314 0.2685444 -1.9592611
C -0.9157132 1.4849451 -1.2531686
C -0.8607099 1.6548374 0.1405291
N -0.3301101 0.6184707 0.9795973
C 0.9896301 0.3474838 1.3141776
C 2.1178093 1.2851759 0.9676541
F 1.9420845 2.5022319 1.5549360
F 3.2986755 0.7970432 1.3791465
F 2.2026686 1.5074787 -0.3699390
N 1.1397924 -0.7578741 2.0055454
C -0.1394302 -1.2822531 2.1410810
C -1.0784371 -0.4229397 1.5162591
C -2.4456507 -0.7007453 1.4692464
C -2.8548263 -1.8877483 2.0760491
C -1.9349423 -2.7548350 2.7026383
C -0.5738096 -2.4675590 2.7471162
C -1.3184574 2.8285306 0.7388342
C -1.8414594 3.8507552 -0.0505698
C -1.8903169 3.7009598 -1.4382062
C -1.4190587 2.5328466 -2.0328861
O 0.4813885 0.3969330 -2.8487708
H -1.5877997 -2.2504240 -3.8370594
H 0.1268674 -1.8346460 -4.0451132
H -0.3489735 -3.5194038 -3.7326083
H -1.1632927 -2.9380959 -1.4917302
H 1.7821926 -0.9616349 -2.1770993
H 2.8616514 -2.0652163 -1.3508218
H -1.5611637 -0.8983159 -0.8167943
H -3.1530469 -0.0282851 0.9939288
H -3.9084766 -2.1495031 2.0674571
H -2.3017068 -3.6695235 3.1580843

H	0.1344938	-3.1384904	3.2221784
H	-1.2502102	2.9321384	1.8166774
H	-2.1992706	4.7630453	0.4160690
H	-2.2898922	4.4975183	-2.0582416
H	-1.4365975	2.4189520	-3.1119841

42

Energy+COSMO-RS = -1363.75256

C	-1.5593114	1.8379772	-3.5657350
C	-0.6832413	0.7902969	-2.8819902
C	0.8172750	0.9656722	-3.2213412
N	1.6652670	0.2043353	-2.4923487
O	1.1890690	1.6856905	-4.1434959
N	-0.9173928	0.7067517	-1.4373267
C	-0.6012477	1.7109330	-0.5760283
C	-1.0461027	1.5329952	0.8573341
C	-0.8296136	0.3760256	1.6243186
N	-0.1889069	-0.7700477	1.0443605
C	1.1607533	-0.9968748	0.8346416
C	2.2341308	-0.1310239	1.4489813
F	2.1582112	-0.1527771	2.8075969
F	3.4571757	-0.5584157	1.0958873
F	2.1206197	1.1659533	1.0686916
N	1.4064516	-2.0658740	0.1074786
C	0.1575630	-2.5939138	-0.2010997
C	-0.8568222	-1.7982139	0.3888275
C	-2.2176752	-2.0608467	0.2235378
C	-2.5402297	-3.1695554	-0.5574027
C	-1.5441359	-3.9771814	-1.1469107
C	-0.1894768	-3.7055729	-0.9796392
C	-1.2262325	0.3081806	2.9591942
C	-1.8553420	1.4015997	3.5508994
C	-2.0690033	2.5637577	2.8069894
C	-1.6559615	2.6303584	1.4785412
O	0.0028324	2.7239321	-0.9225691
H	-2.6106470	1.6303648	-3.3471681
H	-1.3092307	2.8397139	-3.2136713
H	-1.4084668	1.8015059	-4.6462500
H	-0.9495179	-0.2005699	-3.2691928
H	1.3506834	-0.3947409	-1.7464092
H	2.6541327	0.2491734	-2.6849560

H	-1.5433221	-0.0181411	-1.1181976
H	-2.9816867	-1.4401945	0.6804890
H	-3.5850890	-3.4190182	-0.7143368
H	-1.8465520	-4.8301402	-1.7465537
H	0.5765722	-4.3234131	-1.4370242
H	-1.0276783	-0.5971612	3.5231555
H	-2.1662251	1.3483697	4.5894266
H	-2.5516453	3.4220430	3.2641384
H	-1.8009833	3.5376486	0.9015287

42

Energy+COSMO-RS = -1363.75251

C	0.7407563	3.7549464	-2.6431434
C	0.8957503	3.1927767	-1.2190987
C	1.0253584	4.3349527	-0.1989167
N	2.2382760	4.9231478	-0.1532089
O	0.0695058	4.6979139	0.4888448
N	-0.2467034	2.3892952	-0.8273285
C	-0.3456612	1.0719489	-1.1203115
C	-1.5777096	0.3998372	-0.5733918
C	-1.5056161	-0.8612424	0.0396311
N	-0.2476989	-1.5243308	0.2111966
C	0.7064921	-1.3539320	1.2002625
C	0.5195589	-0.4011783	2.3507807
F	-0.6365699	-0.6530989	3.0256728
F	1.5335709	-0.4883229	3.2267624
F	0.4475687	0.8947118	1.9336956
N	1.7623099	-2.1226118	1.0512316
C	1.5171351	-2.8554733	-0.1029265
C	0.2577700	-2.4942000	-0.6438751
C	-0.2543252	-3.0606362	-1.8102698
C	0.5385092	-4.0235170	-2.4290461
C	1.7944115	-4.4004002	-1.9039986
C	2.3006771	-3.8258774	-0.7425926
C	-2.6561368	-1.5066519	0.4889722
C	-3.9021864	-0.9011241	0.3316840
C	-3.9903667	0.3550477	-0.2685773
C	-2.8360440	1.0001304	-0.7108793
O	0.4862259	0.4626406	-1.7967887
H	0.6113968	2.9277701	-3.3448222
H	-0.1337333	4.4098994	-2.6982123

H	1.6277621	4.3247504	-2.9340089
H	1.7880603	2.5591480	-1.1802118
H	3.0182236	4.5779734	-0.6893165
H	2.3889036	5.7070471	0.4642564
H	-0.8823217	2.8237112	-0.1696591
H	-1.2142722	-2.7614352	-2.2174620
H	0.1842907	-4.4942830	-3.3413525
H	2.3767651	-5.1552879	-2.4239849
H	3.2674064	-4.1115306	-0.3397702
H	-2.5623311	-2.4763363	0.9668064
H	-4.7964394	-1.4078874	0.6805422
H	-4.9569373	0.8326900	-0.3964639
H	-2.9103675	1.9701732	-1.1924372

42

Energy+COSMO-RS = -1363.75050

C	-3.6049824	-1.0129052	-0.5034892
C	-2.0836901	-1.0210569	-0.7213442
C	-1.6808449	-2.2806083	-1.5070665
N	-1.2733623	-3.3262823	-0.7591990
O	-1.7928463	-2.3185397	-2.7321208
N	-1.6348239	0.1283220	-1.4972424
C	-1.4405904	1.3940208	-1.0545136
C	-1.2651531	1.6255529	0.4276135
C	-0.2057219	1.1201284	1.1965930
N	0.7400801	0.1806613	0.6655656
C	1.7287959	0.3112663	-0.3027612
C	2.0754204	1.5919978	-1.0288082
F	1.5926552	2.6852884	-0.3969917
F	3.4174733	1.7319455	-1.1173829
F	1.5903687	1.5940226	-2.2932035
N	2.3969046	-0.7985518	-0.5207939
C	1.8548967	-1.7361381	0.3441666
C	0.8266915	-1.1373362	1.1131705
C	0.1065316	-1.8401821	2.0817465
C	0.4474119	-3.1808128	2.2593977
C	1.4662779	-3.7935465	1.4973629
C	2.1804251	-3.0856029	0.5361573
C	-0.0613492	1.4807104	2.5386523
C	-0.9823905	2.3363333	3.1361120
C	-2.0409929	2.8485362	2.3833862

C	-2.1658080	2.5079263	1.0395680
O	-1.3962244	2.3592451	-1.8196565
H	-3.8916598	-0.1125521	0.0472653
H	-4.1242616	-1.0200123	-1.4656547
H	-3.9142634	-1.8900593	0.0727591
H	-1.5857482	-1.0112886	0.2463955
H	-1.0485867	-3.2241500	0.2200827
H	-0.9880015	-4.1761594	-1.2238164
H	-1.7640740	0.0355947	-2.4998529
H	-0.6735987	-1.3709054	2.6714379
H	-0.0832479	-3.7656641	3.0043907
H	1.6949063	-4.8406465	1.6702098
H	2.9640298	-3.5538435	-0.0507496
H	0.7795977	1.0883755	3.1009540
H	-0.8658472	2.6096051	4.1801079
H	-2.7613480	3.5208305	2.8389549
H	-2.9701050	2.9244545	0.4418648

42

Energy+COSMO-RS = -1363.75038

C	1.0872801	2.3517621	-3.5880621
C	0.7454466	1.2205841	-2.6137717
C	0.7120974	-0.1413533	-3.3429847
N	1.4102566	-1.1505780	-2.7741656
O	0.0830053	-0.2606839	-4.3917833
N	1.6332608	1.2252153	-1.4583982
C	1.3230458	1.5000395	-0.1645179
C	-0.1030362	1.8859230	0.1457530
C	-0.9985574	0.9705853	0.7109132
N	-0.6179254	-0.4068944	0.8232974
C	0.1845923	-1.0572227	1.7504673
C	0.6284259	-0.4729159	3.0717408
F	0.3136655	0.8322694	3.2015203
F	0.0215320	-1.1318254	4.0990786
F	1.9591818	-0.6075360	3.2505661
N	0.4381425	-2.3072515	1.4345449
C	-0.2165746	-2.5271871	0.2303144
C	-0.8980436	-1.3486238	-0.1644784
C	-1.6653979	-1.2705636	-1.3262797
C	-1.7270809	-2.4225388	-2.1052337
C	-1.0454193	-3.6018926	-1.7369761

C	-0.2862727	-3.6741851	-0.5721224
C	-2.2837327	1.3574263	1.0834001
C	-2.6830834	2.6816616	0.9052050
C	-1.7982227	3.6067235	0.3485095
C	-0.5159549	3.2104054	-0.0312569
O	2.1653097	1.4689809	0.7323480
H	0.9775685	3.3171005	-3.0874585
H	2.1194105	2.2548527	-3.9400061
H	0.4206248	2.3154268	-4.4510050
H	-0.2710141	1.3678362	-2.2379257
H	1.7589159	-1.0666581	-1.8324650
H	1.2775167	-2.0826367	-3.1385910
H	2.6249450	1.1090813	-1.6342580
H	-2.1845742	-0.3621512	-1.6120455
H	-2.3031831	-2.4078532	-3.0247679
H	-1.1201870	-4.4742467	-2.3792885
H	0.2319789	-4.5837846	-0.2852682
H	-2.9579663	0.6201156	1.5068887
H	-3.6820229	2.9875921	1.1992496
H	-2.1057029	4.6383228	0.2077187
H	0.1733994	3.9299712	-0.4622463

42

Energy+COSMO-RS = -1363.75010

C	2.0423734	2.7755209	-1.0111403
C	0.9166215	3.4600416	-0.2230126
C	0.1319065	4.4473887	-1.1188825
N	-0.7501829	3.8974491	-1.9756567
O	0.3607305	5.6566374	-1.0490510
N	0.0688636	2.5076233	0.5117783
C	-0.5805905	1.4340255	0.0075655
C	-1.2804047	0.5856475	1.0358472
C	-1.1145501	-0.8081312	1.0546867
N	-0.2216754	-1.4485111	0.1361546
C	1.1445991	-1.6546723	0.2455642
C	1.9284230	-1.2493570	1.4650611
F	1.4481396	-1.8494301	2.5900436
F	3.2238723	-1.5779421	1.3453866
F	1.8636591	0.0947919	1.6903049
N	1.6669527	-2.2748211	-0.7882964
C	0.6023476	-2.5044214	-1.6505387

C	-0.5936600	-1.9950308	-1.0855659
C	-1.8281540	-2.0771314	-1.7280310
C	-1.8357343	-2.7005314	-2.9727728
C	-0.6554708	-3.2166059	-3.5522779
C	0.5731391	-3.1264142	-2.9064675
C	-1.8153699	-1.5994203	1.9624520
C	-2.6956845	-1.0076268	2.8678169
C	-2.8647588	0.3769957	2.8663523
C	-2.1575907	1.1667044	1.9599427
O	-0.6421834	1.1588412	-1.1972774
H	2.6572785	2.1772373	-0.3338037
H	1.6450852	2.1269303	-1.7925610
H	2.6732720	3.5409881	-1.4715559
H	1.3619445	4.0905360	0.5474329
H	-0.8571388	2.8878827	-2.0239339
H	-1.2531223	4.4951893	-2.6147392
H	0.0743746	2.6031561	1.5159048
H	-2.7309122	-1.6689529	-1.2864203
H	-2.7727252	-2.7893768	-3.5145045
H	-0.7129112	-3.6927745	-4.5265394
H	1.4816627	-3.5196837	-3.3515131
H	-1.6572388	-2.6728331	1.9567065
H	-3.2420277	-1.6269388	3.5720654
H	-3.5503364	0.8441343	3.5666546
H	-2.3056027	2.2422667	1.9492778

42

Energy+COSMO-RS = -1363.74970

C	-3.6442718	-1.1071240	-1.2592493
C	-2.1392829	-1.0044900	-0.9929879
C	-1.4236260	-2.3277566	-1.3314374
N	-0.2637897	-2.2227064	-2.0177675
O	-1.9106515	-3.4014148	-0.9857067
N	-1.5540008	0.1447810	-1.6832947
C	-1.4044255	1.4010681	-1.1697383
C	-1.3635799	1.5666596	0.3281582
C	-0.3746385	1.0309575	1.1659313
N	0.6469274	0.1550871	0.6712799
C	1.7554538	0.4029934	-0.1306745
C	2.1110182	1.7388137	-0.7386748
F	1.5230983	2.7716019	-0.0939636

F	3.4463774	1.9380033	-0.6985373
F	1.7392733	1.8036479	-2.0440731
N	2.5100057	-0.6569547	-0.3185930
C	1.9066854	-1.6777346	0.4004753
C	0.7426196	-1.1873416	1.0412133
C	-0.0762712	-1.9935450	1.8322916
C	0.3036832	-3.3263642	1.9595340
C	1.4575837	-3.8330669	1.3248192
C	2.2731774	-3.0225245	0.5424878
C	-0.3734695	1.3089995	2.5347478
C	-1.3655765	2.1150874	3.0856870
C	-2.3521546	2.6609730	2.2620607
C	-2.3378230	2.3998207	0.8949482
O	-1.3105040	2.3900096	-1.8946739
H	-4.1437970	-0.2032513	-0.9001835
H	-3.8375958	-1.2125983	-2.3315513
H	-4.0572241	-1.9758500	-0.7441609
H	-1.9823473	-0.8555760	0.0780995
H	0.1676614	-1.3257702	-2.1751102
H	0.2974922	-3.0512141	-2.1466998
H	-1.6761281	0.1550361	-2.6906557
H	-0.9655498	-1.6085821	2.3194101
H	-0.3085502	-3.9944004	2.5571150
H	1.7099923	-4.8814168	1.4518160
H	3.1605049	-3.4095350	0.0515554
H	0.4134969	0.8924748	3.1547315
H	-1.3600939	2.3242162	4.1507453
H	-3.1258645	3.2960909	2.6819266
H	-3.0890183	2.8379826	0.2458156

42

Energy+COSMO-RS = -1363.74912

C	1.4722946	3.3090190	-1.9664653
C	0.3151336	3.0271951	-1.0004131
C	-0.3823412	4.3562354	-0.6403424
N	-1.6320402	4.5307912	-1.1223420
O	0.2111273	5.1980540	0.0318595
N	-0.5742404	2.0179600	-1.5671230
C	-1.1447574	0.9406756	-0.9550364
C	-1.0266726	0.8456141	0.5432206
C	-0.5003085	-0.3035622	1.1548766

N -0.0102198 -1.3987024 0.3702704
C 1.2871645 -1.6811824 -0.0289682
C 2.4497658 -0.7839334 0.2969846
F 2.6686000 -0.7130118 1.6401946
F 3.5812481 -1.2191258 -0.2779664
F 2.2365128 0.4952491 -0.1250215
N 1.4042130 -2.8210418 -0.6714835
C 0.1215961 -3.3531217 -0.7046352
C -0.7802400 -2.4779062 -0.0497353
C -2.1397289 -2.7559372 0.0838702
C -2.5811802 -3.9559913 -0.4660166
C -1.6992953 -4.8398055 -1.1269907
C -0.3445182 -4.5541827 -1.2575126
C -0.4646425 -0.4208351 2.5434816
C -0.9617609 0.6061935 3.3445227
C -1.4983540 1.7482531 2.7507692
C -1.5249609 1.8661686 1.3620625
O -1.7650554 0.0909863 -1.5950168
H 2.0378445 2.3931621 -2.1510255
H 1.0910525 3.6874753 -2.9210499
H 2.1371028 4.0601843 -1.5366463
H 0.7159118 2.6439009 -0.0606190
H -2.1063907 3.7996630 -1.6278855
H -2.1296838 5.3840649 -0.9165511
H -0.6656903 2.0033029 -2.5762454
H -2.8175171 -2.0678545 0.5766629
H -3.6324029 -4.2176592 -0.3898968
H -2.0929868 -5.7627405 -1.5423142
H 0.3355059 -5.2313411 -1.7647623
H -0.0452742 -1.3189026 2.9844912
H -0.9319353 0.5101958 4.4252284
H -1.8959821 2.5496339 3.3656913
H -1.9536664 2.7523752 0.9074389

(*P*)-atropisomer in DMSO (14 structures)

42
Energy+COSMO-RS = -1363.75915 ; # imag. freq. COSMO = 1 ; # imag. freq. cacuum = 0
C 2.2147176 1.2902114 -1.9785850

C	0.8250796	1.9473121	-2.0631734
C	0.1242024	1.5811412	-3.3809703
N	0.6257751	2.1861741	-4.4793364
O	-0.8030774	0.7724405	-3.4173416
N	-0.0316245	1.5079071	-0.9781038
C	-0.0125573	2.0771763	0.2469533
C	-0.9164484	1.4665726	1.2901328
C	-1.0504288	0.0874575	1.5241693
N	-0.3636417	-0.8752145	0.7123798
C	0.9547642	-1.2986077	0.7653478
C	1.9016228	-0.8633899	1.8537154
F	1.3946323	-1.1377609	3.0877579
F	3.0851335	-1.4895406	1.7466004
F	2.1379907	0.4750389	1.8192318
N	1.2744648	-2.1411871	-0.1911240
C	0.1160893	-2.3006035	-0.9413119
C	-0.9282188	-1.5211085	-0.3829470
C	-2.2113611	-1.4710539	-0.9277485
C	-2.4270868	-2.2429148	-2.0664280
C	-1.4018818	-3.0270438	-2.6360035
C	-0.1234811	-3.0680618	-2.0885014
C	-1.8711719	-0.3922235	2.5459324
C	-2.5771001	0.4997325	3.3491462
C	-2.4456653	1.8740514	3.1378270
C	-1.6133641	2.3479087	2.1278364
O	0.6883268	3.0546203	0.5256241
H	2.6737307	1.5384109	-1.0191653
H	2.1242840	0.2031206	-2.0564175
H	2.8667223	1.6494653	-2.7794643
H	0.9350714	3.0344219	-1.9980161
H	1.3739969	2.8583325	-4.4244541
H	0.2524168	1.9561363	-5.3880298
H	-0.7104227	0.7973477	-1.2247993
H	-2.9965568	-0.8624488	-0.4913919
H	-3.4083009	-2.2363000	-2.5310280
H	-1.6189604	-3.6086112	-3.5269074
H	0.6662960	-3.6686458	-2.5286850
H	-1.9395294	-1.4633764	2.7052206
H	-3.2167657	0.1232237	4.1412092
H	-2.9874611	2.5762589	3.7640284
H	-1.4904410	3.4140993	1.9694953

42

Energy+COSMO-RS = -1363.75750 ; # imag. freq. COSMO = 1 ; # imag. freq. cacuum = 0

C 1.3198505 -0.5102328 -2.6680575
C 0.9012599 0.8951336 -2.2292546
C -0.0131457 1.5270640 -3.3002103
N 0.5804899 2.4552428 -4.0829796
O -1.1776694 1.1587234 -3.4424507
N 0.2057413 0.8273356 -0.9563030
C 0.1456692 1.8794249 -0.1035049
C -0.6273673 1.6671631 1.1742034
C -0.5997209 0.4945121 1.9500088
N 0.1606555 -0.6520502 1.5409157
C 1.5167909 -0.9046713 1.6744230
C 2.4380591 0.0130962 2.4367031
F 1.9789251 0.2434831 3.6967446
F 3.6710062 -0.5092400 2.5359198
F 2.5497319 1.2266642 1.8325896
N 1.9057182 -2.0123988 1.0856338
C 0.7591682 -2.5427721 0.5089538
C -0.3499860 -1.7050973 0.7894194
C -1.6331643 -1.9579368 0.3023609
C -1.7808670 -3.1020761 -0.4799917
C -0.6915043 -3.9541005 -0.7602057
C 0.5857944 -3.6911022 -0.2736534
C -1.3231814 0.4022541 3.1394040
C -2.0923525 1.4793427 3.5727356
C -2.1220825 2.6553703 2.8205056
C -1.3865896 2.7480452 1.6425546
O 0.6891037 2.9616979 -0.3370254
H 1.9847009 -0.9531541 -1.9223522
H 0.4408063 -1.1516027 -2.7845961
H 1.8434683 -0.4665259 -3.6267439
H 1.7824898 1.5225363 -2.0792702
H 1.4919481 2.8235232 -3.8620444
H 0.0414479 2.9169922 -4.8011583
H -0.4281043 0.0525439 -0.8246649
H -2.4696126 -1.3008952 0.5180051
H -2.7592954 -3.3418453 -0.8849431
H -0.8568641 -4.8331774 -1.3756506
H 1.4258228 -4.3407688 -0.4975572

H	-1.2667343	-0.5134315	3.7188438
H	-2.6566659	1.4016455	4.4966558
H	-2.7149537	3.5012873	3.1540857
H	-1.3911081	3.6626880	1.0594765

42

Energy+COSMO-RS = -1363.75745

C	0.7407563	3.7549464	-2.6431434
C	0.8957503	3.1927767	-1.2190987
C	1.0253584	4.3349527	-0.1989167
N	2.2382760	4.9231478	-0.1532089
O	0.0695058	4.6979139	0.4888448
N	-0.2467034	2.3892952	-0.8273285
C	-0.3456612	1.0719489	-1.1203115
C	-1.5777096	0.3998372	-0.5733918
C	-1.5056161	-0.8612424	0.0396311
N	-0.2476989	-1.5243308	0.2111966
C	0.7064921	-1.3539320	1.2002625
C	0.5195589	-0.4011783	2.3507807
F	-0.6365699	-0.6530989	3.0256728
F	1.5335709	-0.4883229	3.2267624
F	0.4475687	0.8947118	1.9336956
N	1.7623099	-2.1226118	1.0512316
C	1.5171351	-2.8554733	-0.1029265
C	0.2577700	-2.4942000	-0.6438751
C	-0.2543252	-3.0606362	-1.8102698
C	0.5385092	-4.0235170	-2.4290461
C	1.7944115	-4.4004002	-1.9039986
C	2.3006771	-3.8258774	-0.7425926
C	-2.6561368	-1.5066519	0.4889722
C	-3.9021864	-0.9011241	0.3316840
C	-3.9903667	0.3550477	-0.2685773
C	-2.8360440	1.0001304	-0.7108793
O	0.4862259	0.4626406	-1.7967887
H	0.6113968	2.9277701	-3.3448222
H	-0.1337333	4.4098994	-2.6982123
H	1.6277621	4.3247504	-2.9340089
H	1.7880603	2.5591480	-1.1802118
H	3.0182236	4.5779734	-0.6893165
H	2.3889036	5.7070471	0.4642564
H	-0.8823217	2.8237112	-0.1696591

H	-1.2142722	-2.7614352	-2.2174620
H	0.1842907	-4.4942830	-3.3413525
H	2.3767651	-5.1552879	-2.4239849
H	3.2674064	-4.1115306	-0.3397702
H	-2.5623311	-2.4763363	0.9668064
H	-4.7964394	-1.4078874	0.6805422
H	-4.9569373	0.8326900	-0.3964639
H	-2.9103675	1.9701732	-1.1924372

42

Energy+COSMO-RS = -1363.75528 ; # imag. freq. COSMO = 1 ; # imag. freq. cacuum = 0

C	0.0282225	4.9299404	-0.4955844
C	0.6054420	3.5454934	-0.7997461
C	1.8494547	3.2966215	0.0793184
N	1.6773146	2.5036567	1.1579310
O	2.9099282	3.8560949	-0.1936998
N	-0.4357693	2.5337238	-0.6613036
C	-0.3170893	1.2928756	-1.2080786
C	-1.5459348	0.4292722	-1.1025868
C	-1.4782034	-0.8641410	-0.5622640
N	-0.2517049	-1.3532662	-0.0058743
C	0.2079915	-1.2487933	1.2979224
C	-0.5993566	-0.6080199	2.3926955
F	-1.7651660	-1.2683034	2.6191335
F	0.0828305	-0.5659086	3.5458365
F	-0.9489095	0.6817068	2.0774524
N	1.3813181	-1.8089855	1.4890825
C	1.7419072	-2.3267423	0.2516710
C	0.7269406	-2.0542228	-0.6998309
C	0.8135028	-2.4534993	-2.0326963
C	1.9626056	-3.1503437	-2.3938528
C	2.9861233	-3.4313596	-1.4612261
C	2.8941946	-3.0260274	-0.1343929
C	-2.5998431	-1.6899479	-0.5407598
C	-3.8108901	-1.2313600	-1.0596470
C	-3.8963095	0.0560588	-1.5893585
C	-2.7717885	0.8816631	-1.6043237
O	0.7070942	0.9054505	-1.7660172
H	-0.7834137	5.1630630	-1.1906596
H	-0.3575284	4.9743157	0.5286111
H	0.8100146	5.6838900	-0.6035099

H	0.9758874	3.5137960	-1.8281543
H	0.8070418	2.0263245	1.3232710
H	2.4638053	2.3042562	1.7573913
H	-1.3033801	2.7874278	-0.2119976
H	0.0347108	-2.2209605	-2.7505631
H	2.0793495	-3.4815910	-3.4214017
H	3.8653909	-3.9749678	-1.7937369
H	3.6805859	-3.2380543	0.5831010
H	-2.5173156	-2.6814599	-0.1081370
H	-4.6828084	-1.8775380	-1.0440463
H	-4.8360982	0.4184906	-1.9943842
H	-2.8362893	1.8783718	-2.0304653

42

Energy+COSMO-RS = -1363.75508 ; # imag. freq. COSMO = 0 ; # imag. freq. cacuum = 0

C	1.5699574	0.3456725	3.6604456
C	1.6847042	1.0681280	2.3283446
C	2.7626656	0.4336407	1.4077677
N	3.1044281	1.2122438	0.3545082
O	3.2473227	-0.6725392	1.6263747
N	0.3934346	1.0468040	1.6336880
C	-0.0111154	1.9888930	0.7535066
C	-1.3560341	1.7497766	0.1209192
C	-1.6417024	0.5972828	-0.6288583
N	-0.6168106	-0.3809751	-0.8427105
C	-0.4766277	-1.6521175	-0.3013996
C	-1.4990479	-2.2614937	0.6196050
F	-2.6771075	-2.5117987	-0.0082303
F	-1.0607453	-3.4118569	1.1457297
F	-1.7894858	-1.4144238	1.6600044
N	0.6363251	-2.2535148	-0.6530800
C	1.2971942	-1.3501661	-1.4742593
C	0.5270539	-0.1673404	-1.6071188
C	0.9383041	0.9203437	-2.3774299
C	2.1677374	0.7919589	-3.0179090
C	2.9540502	-0.3746948	-2.8903762
C	2.5344162	-1.4566464	-2.1229458
C	-2.8851963	0.4310277	-1.2382038
C	-3.8688009	1.4053951	-1.0783544
C	-3.5952054	2.5589027	-0.3404127
C	-2.3415082	2.7353335	0.2414163

O 0.6556611 2.9934226 0.4763986
H 0.8114663 0.8267834 4.2837552
H 1.3032855 -0.7047681 3.5118783
H 2.5278784 0.3734382 4.1838675
H 1.9441754 2.1200820 2.4805154
H 2.5674543 2.0465682 0.1525170
H 3.6454595 0.8003292 -0.3926853
H -0.2244645 0.2731146 1.8290351
H 0.3421251 1.8220358 -2.4622613
H 2.5343122 1.6129980 -3.6264810
H 3.9083583 -0.4245590 -3.4059564
H 3.1378321 -2.3526095 -2.0206260
H -3.0681206 -0.4524073 -1.8396492
H -4.8396098 1.2704562 -1.5446153
H -4.3561085 3.3243535 -0.2237925
H -2.1167185 3.6356464 0.8042678

42
Energy+COSMO-RS = -1363.75368 ; # imag. freq. COSMO = 0 ; # imag. freq. cacuum = 0
C 1.2796810 2.7585020 -1.2589102
C 1.0898430 2.6453844 0.2459481
C 0.5593919 3.9615128 0.8759845
N 0.6547860 3.9900479 2.2242897
O 0.0986822 4.8798636 0.2007752
N 0.1690204 1.5478760 0.5481450
C 0.2118150 0.8136701 1.6825675
C -0.7623341 -0.3305980 1.7856917
C -1.0571547 -1.2325314 0.7478395
N -0.4516036 -1.1017091 -0.5463021
C 0.7917826 -1.5309791 -0.9839812
C 1.7191626 -2.3385884 -0.1119469
F 1.1104113 -3.4634600 0.3523208
F 2.8177254 -2.7118693 -0.7863467
F 2.1208784 -1.6321077 0.9788474
N 1.0654926 -1.1761695 -2.2185662
C -0.0438712 -0.4589418 -2.6481633
C -1.0121137 -0.4079368 -1.6133623
C -2.2189406 0.2822488 -1.7378006
C -2.4367335 0.9277465 -2.9541573
C -1.4885836 0.8814548 -3.9979480
C -0.2872148 0.1915807 -3.8640536

C -1.9532210 -2.2835679 0.9429939
C -2.5719848 -2.4515522 2.1795347
C -2.2792035 -1.5737784 3.2251269
C -1.3753875 -0.5333683 3.0296925
O 0.9889666 1.0619519 2.6138634
H 1.6973477 1.8287401 -1.6524270
H 0.3291304 2.9618350 -1.7604403
H 1.9616409 3.5796065 -1.4891762
H 2.0378807 2.3995093 0.7335025
H 0.9434506 3.1580198 2.7271755
H 0.2453967 4.7605295 2.7321354
H -0.5764023 1.3874436 -0.1148750
H -2.9480306 0.3141605 -0.9346078
H -3.3595278 1.4803616 -3.1009519
H -1.7035414 1.4020674 -4.9260469
H 0.4459748 0.1639188 -4.6637046
H -2.1468125 -2.9674860 0.1232732
H -3.2700063 -3.2696077 2.3267032
H -2.7517189 -1.7025228 4.1939508
H -1.1312170 0.1441016 3.8409026

42
Energy+COSMO-RS = -1363.75361
C 1.0872801 2.3517621 -3.5880621
C 0.7454466 1.2205841 -2.6137717
C 0.7120974 -0.1413533 -3.3429847
N 1.4102566 -1.1505780 -2.7741656
O 0.0830053 -0.2606839 -4.3917833
N 1.6332608 1.2252153 -1.4583982
C 1.3230458 1.5000395 -0.1645179
C -0.1030362 1.8859230 0.1457530
C -0.9985574 0.9705853 0.7109132
N -0.6179254 -0.4068944 0.8232974
C 0.1845923 -1.0572227 1.7504673
C 0.6284259 -0.4729159 3.0717408
F 0.3136655 0.8322694 3.2015203
F 0.0215320 -1.1318254 4.0990786
F 1.9591818 -0.6075360 3.2505661
N 0.4381425 -2.3072515 1.4345449
C -0.2165746 -2.5271871 0.2303144
C -0.8980436 -1.3486238 -0.1644784

C	-1.6653979	-1.2705636	-1.3262797
C	-1.7270809	-2.4225388	-2.1052337
C	-1.0454193	-3.6018926	-1.7369761
C	-0.2862727	-3.6741851	-0.5721224
C	-2.2837327	1.3574263	1.0834001
C	-2.6830834	2.6816616	0.9052050
C	-1.7982227	3.6067235	0.3485095
C	-0.5159549	3.2104054	-0.0312569
O	2.1653097	1.4689809	0.7323480
H	0.9775685	3.3171005	-3.0874585
H	2.1194105	2.2548527	-3.9400061
H	0.4206248	2.3154268	-4.4510050
H	-0.2710141	1.3678362	-2.2379257
H	1.7589159	-1.0666581	-1.8324650
H	1.2775167	-2.0826367	-3.1385910
H	2.6249450	1.1090813	-1.6342580
H	-2.1845742	-0.3621512	-1.6120455
H	-2.3031831	-2.4078532	-3.0247679
H	-1.1201870	-4.4742467	-2.3792885
H	0.2319789	-4.5837846	-0.2852682
H	-2.9579663	0.6201156	1.5068887
H	-3.6820229	2.9875921	1.1992496
H	-2.1057029	4.6383228	0.2077187
H	0.1733994	3.9299712	-0.4622463

42

Energy+COSMO-RS = -1363.75355

C	1.4722946	3.3090190	-1.9664653
C	0.3151336	3.0271951	-1.0004131
C	-0.3823412	4.3562354	-0.6403424
N	-1.6320402	4.5307912	-1.1223420
O	0.2111273	5.1980540	0.0318595
N	-0.5742404	2.0179600	-1.5671230
C	-1.1447574	0.9406756	-0.9550364
C	-1.0266726	0.8456141	0.5432206
C	-0.5003085	-0.3035622	1.1548766
N	-0.0102198	-1.3987024	0.3702704
C	1.2871645	-1.6811824	-0.0289682
C	2.4497658	-0.7839334	0.2969846
F	2.6686000	-0.7130118	1.6401946
F	3.5812481	-1.2191258	-0.2779664

F	2.2365128	0.4952491	-0.1250215
N	1.4042130	-2.8210418	-0.6714835
C	0.1215961	-3.3531217	-0.7046352
C	-0.7802400	-2.4779062	-0.0497353
C	-2.1397289	-2.7559372	0.0838702
C	-2.5811802	-3.9559913	-0.4660166
C	-1.6992953	-4.8398055	-1.1269907
C	-0.3445182	-4.5541827	-1.2575126
C	-0.4646425	-0.4208351	2.5434816
C	-0.9617609	0.6061935	3.3445227
C	-1.4983540	1.7482531	2.7507692
C	-1.5249609	1.8661686	1.3620625
O	-1.7650554	0.0909863	-1.5950168
H	2.0378445	2.3931621	-2.1510255
H	1.0910525	3.6874753	-2.9210499
H	2.1371028	4.0601843	-1.5366463
H	0.7159118	2.6439009	-0.0606190
H	-2.1063907	3.7996630	-1.6278855
H	-2.1296838	5.3840649	-0.9165511
H	-0.6656903	2.0033029	-2.5762454
H	-2.8175171	-2.0678545	0.5766629
H	-3.6324029	-4.2176592	-0.3898968
H	-2.0929868	-5.7627405	-1.5423142
H	0.3355059	-5.2313411	-1.7647623
H	-0.0452742	-1.3189026	2.9844912
H	-0.9319353	0.5101958	4.4252284
H	-1.8959821	2.5496339	3.3656913
H	-1.9536664	2.7523752	0.9074389

42

Energy+COSMO-RS = -1363.75354

C	-3.6442718	-1.1071240	-1.2592493
C	-2.1392829	-1.0044900	-0.9929879
C	-1.4236260	-2.3277566	-1.3314374
N	-0.2637897	-2.2227064	-2.0177675
O	-1.9106515	-3.4014148	-0.9857067
N	-1.5540008	0.1447810	-1.6832947
C	-1.4044255	1.4010681	-1.1697383
C	-1.3635799	1.5666596	0.3281582
C	-0.3746385	1.0309575	1.1659313
N	0.6469274	0.1550871	0.6712799

C	1.7554538	0.4029934	-0.1306745
C	2.1110182	1.7388137	-0.7386748
F	1.5230983	2.7716019	-0.0939636
F	3.4463774	1.9380033	-0.6985373
F	1.7392733	1.8036479	-2.0440731
N	2.5100057	-0.6569547	-0.3185930
C	1.9066854	-1.6777346	0.4004753
C	0.7426196	-1.1873416	1.0412133
C	-0.0762712	-1.9935450	1.8322916
C	0.3036832	-3.3263642	1.9595340
C	1.4575837	-3.8330669	1.3248192
C	2.2731774	-3.0225245	0.5424878
C	-0.3734695	1.3089995	2.5347478
C	-1.3655765	2.1150874	3.0856870
C	-2.3521546	2.6609730	2.2620607
C	-2.3378230	2.3998207	0.8949482
O	-1.3105040	2.3900096	-1.8946739
H	-4.1437970	-0.2032513	-0.9001835
H	-3.8375958	-1.2125983	-2.3315513
H	-4.0572241	-1.9758500	-0.7441609
H	-1.9823473	-0.8555760	0.0780995
H	0.1676614	-1.3257702	-2.1751102
H	0.2974922	-3.0512141	-2.1466998
H	-1.6761281	0.1550361	-2.6906557
H	-0.9655498	-1.6085821	2.3194101
H	-0.3085502	-3.9944004	2.5571150
H	1.7099923	-4.8814168	1.4518160
H	3.1605049	-3.4095350	0.0515554
H	0.4134969	0.8924748	3.1547315
H	-1.3600939	2.3242162	4.1507453
H	-3.1258645	3.2960909	2.6819266
H	-3.0890183	2.8379826	0.2458156

42

Energy+COSMO-RS = -1363.75212

C	2.0423734	2.7755209	-1.0111403
C	0.9166215	3.4600416	-0.2230126
C	0.1319065	4.4473887	-1.1188825
N	-0.7501829	3.8974491	-1.9756567
O	0.3607305	5.6566374	-1.0490510
N	0.0688636	2.5076233	0.5117783

C	-0.5805905	1.4340255	0.0075655
C	-1.2804047	0.5856475	1.0358472
C	-1.1145501	-0.8081312	1.0546867
N	-0.2216754	-1.4485111	0.1361546
C	1.1445991	-1.6546723	0.2455642
C	1.9284230	-1.2493570	1.4650611
F	1.4481396	-1.8494301	2.5900436
F	3.2238723	-1.5779421	1.3453866
F	1.8636591	0.0947919	1.6903049
N	1.6669527	-2.2748211	-0.7882964
C	0.6023476	-2.5044214	-1.6505387
C	-0.5936600	-1.9950308	-1.0855659
C	-1.8281540	-2.0771314	-1.7280310
C	-1.8357343	-2.7005314	-2.9727728
C	-0.6554708	-3.2166059	-3.5522779
C	0.5731391	-3.1264142	-2.9064675
C	-1.8153699	-1.5994203	1.9624520
C	-2.6956845	-1.0076268	2.8678169
C	-2.8647588	0.3769957	2.8663523
C	-2.1575907	1.1667044	1.9599427
O	-0.6421834	1.1588412	-1.1972774
H	2.6572785	2.1772373	-0.3338037
H	1.6450852	2.1269303	-1.7925610
H	2.6732720	3.5409881	-1.4715559
H	1.3619445	4.0905360	0.5474329
H	-0.8571388	2.8878827	-2.0239339
H	-1.2531223	4.4951893	-2.6147392
H	0.0743746	2.6031561	1.5159048
H	-2.7309122	-1.6689529	-1.2864203
H	-2.7727252	-2.7893768	-3.5145045
H	-0.7129112	-3.6927745	-4.5265394
H	1.4816627	-3.5196837	-3.3515131
H	-1.6572388	-2.6728331	1.9567065
H	-3.2420277	-1.6269388	3.5720654
H	-3.5503364	0.8441343	3.5666546
H	-2.3056027	2.2422667	1.9492778

42

Energy+COSMO-RS = -1363.75130

C	-1.5593114	1.8379772	-3.5657350
C	-0.6832413	0.7902969	-2.8819902

C	0.8172750	0.9656722	-3.2213412
N	1.6652670	0.2043353	-2.4923487
O	1.1890690	1.6856905	-4.1434959
N	-0.9173928	0.7067517	-1.4373267
C	-0.6012477	1.7109330	-0.5760283
C	-1.0461027	1.5329952	0.8573341
C	-0.8296136	0.3760256	1.6243186
N	-0.1889069	-0.7700477	1.0443605
C	1.1607533	-0.9968748	0.8346416
C	2.2341308	-0.1310239	1.4489813
F	2.1582112	-0.1527771	2.8075969
F	3.4571757	-0.5584157	1.0958873
F	2.1206197	1.1659533	1.0686916
N	1.4064516	-2.0658740	0.1074786
C	0.1575630	-2.5939138	-0.2010997
C	-0.8568222	-1.7982139	0.3888275
C	-2.2176752	-2.0608467	0.2235378
C	-2.5402297	-3.1695554	-0.5574027
C	-1.5441359	-3.9771814	-1.1469107
C	-0.1894768	-3.7055729	-0.9796392
C	-1.2262325	0.3081806	2.9591942
C	-1.8553420	1.4015997	3.5508994
C	-2.0690033	2.5637577	2.8069894
C	-1.6559615	2.6303584	1.4785412
O	0.0028324	2.7239321	-0.9225691
H	-2.6106470	1.6303648	-3.3471681
H	-1.3092307	2.8397139	-3.2136713
H	-1.4084668	1.8015059	-4.6462500
H	-0.9495179	-0.2005699	-3.2691928
H	1.3506834	-0.3947409	-1.7464092
H	2.6541327	0.2491734	-2.6849560
H	-1.5433221	-0.0181411	-1.1181976
H	-2.9816867	-1.4401945	0.6804890
H	-3.5850890	-3.4190182	-0.7143368
H	-1.8465520	-4.8301402	-1.7465537
H	0.5765722	-4.3234131	-1.4370242
H	-1.0276783	-0.5971612	3.5231555
H	-2.1662251	1.3483697	4.5894266
H	-2.5516453	3.4220430	3.2641384
H	-1.8009833	3.5376486	0.9015287

42

Energy+COSMO-RS = -1363.75124

C	2.4328131	0.9230965	-1.8893378
C	1.1684602	1.7807025	-2.0385039
C	0.4869349	1.5893958	-3.4032990
N	0.0069487	0.3448350	-3.6476296
O	0.3808989	2.5142727	-4.2050010
N	0.2025747	1.5038399	-0.9716259
C	0.3070282	2.0466413	0.2670829
C	-0.7087103	1.5878700	1.2857855
C	-1.0647294	0.2463209	1.5070365
N	-0.5158172	-0.8075117	0.6999096
C	0.7136885	-1.4403455	0.7916276
C	1.6667563	-1.1959708	1.9357657
F	1.0721621	-1.4550456	3.1318073
F	2.7522983	-1.9787143	1.8351470
F	2.0861533	0.0947421	1.9760939
N	0.9427247	-2.2898258	-0.1840031
C	-0.1861300	-2.2322627	-0.9914808
C	-1.1195802	-1.3131959	-0.4458228
C	-2.3439457	-1.0281302	-1.0517274
C	-2.6186507	-1.7060564	-2.2380326
C	-1.7065021	-2.6280354	-2.7933702
C	-0.4844899	-2.9044913	-2.1848593
C	-1.9711805	-0.1032573	2.5081435
C	-2.5418890	0.8866647	3.3046518
C	-2.1902273	2.2235696	3.1062726
C	-1.2733097	2.5650693	2.1159536
O	1.1657077	2.8760181	0.5681382
H	2.9239333	1.1616245	-0.9433034
H	2.1945863	-0.1446465	-1.8937987
H	3.1283465	1.1313894	-2.7074460
H	1.4367719	2.8363963	-1.9794215
H	0.1983339	-0.4445428	-3.0491628
H	-0.4291548	0.1559769	-4.5378810
H	-0.5898018	0.9164163	-1.1845244
H	-3.0438607	-0.3179953	-0.6236345
H	-3.5577638	-1.5175429	-2.7485995
H	-1.9651705	-3.1303971	-3.7203181
H	0.2184278	-3.6116943	-2.6133554
H	-2.2117158	-1.1505235	2.6585066

H -3.2486821 0.6131126 4.0816215
H -2.6259566 2.9996938 3.7278367
H -0.9799653 3.5990853 1.9685433

42
Energy+COSMO-RS = -1363.75074 ; # imag. freq. COSMO = 20 ; # imag. freq. cacuum = 0
C -0.5705614 -2.4734159 -3.5028720
C -0.4646819 -2.2624683 -1.9875805
C 0.9239506 -2.6918452 -1.4555570
N 1.9325805 -1.8203672 -1.6594195
O 1.0620740 -3.7866812 -0.9102849
N -0.8691412 -0.9180546 -1.5515634
C -0.3696314 0.2685444 -1.9592611
C -0.9157132 1.4849451 -1.2531686
C -0.8607099 1.6548374 0.1405291
N -0.3301101 0.6184707 0.9795973
C 0.9896301 0.3474838 1.3141776
C 2.1178093 1.2851759 0.9676541
F 1.9420845 2.5022319 1.5549360
F 3.2986755 0.7970432 1.3791465
F 2.2026686 1.5074787 -0.3699390
N 1.1397924 -0.7578741 2.0055454
C -0.1394302 -1.2822531 2.1410810
C -1.0784371 -0.4229397 1.5162591
C -2.4456507 -0.7007453 1.4692464
C -2.8548263 -1.8877483 2.0760491
C -1.9349423 -2.7548350 2.7026383
C -0.5738096 -2.4675590 2.7471162
C -1.3184574 2.8285306 0.7388342
C -1.8414594 3.8507552 -0.0505698
C -1.8903169 3.7009598 -1.4382062
C -1.4190587 2.5328466 -2.0328861
O 0.4813885 0.3969330 -2.8487708
H -1.5877997 -2.2504240 -3.8370594
H 0.1268674 -1.8346460 -4.0451132
H -0.3489735 -3.5194038 -3.7326083
H -1.1632927 -2.9380959 -1.4917302
H 1.7821926 -0.9616349 -2.1770993
H 2.8616514 -2.0652163 -1.3508218
H -1.5611637 -0.8983159 -0.8167943
H -3.1530469 -0.0282851 0.9939288

H -3.9084766 -2.1495031 2.0674571
H -2.3017068 -3.6695235 3.1580843
H 0.1344938 -3.1384904 3.2221784
H -1.2502102 2.9321384 1.8166774
H -2.1992706 4.7630453 0.4160690
H -2.2898922 4.4975183 -2.0582416
H -1.4365975 2.4189520 -3.1119841

42
Energy+COSMO-RS = -1363.74917
C -3.6049824 -1.0129052 -0.5034892
C -2.0836901 -1.0210569 -0.7213442
C -1.6808449 -2.2806083 -1.5070665
N -1.2733623 -3.3262823 -0.7591990
O -1.7928463 -2.3185397 -2.7321208
N -1.6348239 0.1283220 -1.4972424
C -1.4405904 1.3940208 -1.0545136
C -1.2651531 1.6255529 0.4276135
C -0.2057219 1.1201284 1.1965930
N 0.7400801 0.1806613 0.6655656
C 1.7287959 0.3112663 -0.3027612
C 2.0754204 1.5919978 -1.0288082
F 1.5926552 2.6852884 -0.3969917
F 3.4174733 1.7319455 -1.1173829
F 1.5903687 1.5940226 -2.2932035
N 2.3969046 -0.7985518 -0.5207939
C 1.8548967 -1.7361381 0.3441666
C 0.8266915 -1.1373362 1.1131705
C 0.1065316 -1.8401821 2.0817465
C 0.4474119 -3.1808128 2.2593977
C 1.4662779 -3.7935465 1.4973629
C 2.1804251 -3.0856029 0.5361573
C -0.0613492 1.4807104 2.5386523
C -0.9823905 2.3363333 3.1361120
C -2.0409929 2.8485362 2.3833862
C -2.1658080 2.5079263 1.0395680
O -1.3962244 2.3592451 -1.8196565
H -3.8916598 -0.1125521 0.0472653
H -4.1242616 -1.0200123 -1.4656547
H -3.9142634 -1.8900593 0.0727591
H -1.5857482 -1.0112886 0.2463955

H -1.0485867 -3.2241500 0.2200827
H -0.9880015 -4.1761594 -1.2238164
H -1.7640740 0.0355947 -2.4998529
H -0.6735987 -1.3709054 2.6714379
H -0.0832479 -3.7656641 3.0043907
H 1.6949063 -4.8406465 1.6702098
H 2.9640298 -3.5538435 -0.0507496
H 0.7795977 1.0883755 3.1009540
H -0.8658472 2.6096051 4.1801079
H -2.7613480 3.5208305 2.8389549
H -2.9701050 2.9244545 0.4418648

(*M*)-atropisomer in CDCl3 (14 structures)

42
Energy+COSMO-RS = -1363.75736 ; # imag. freq. COSMO = 0 ; # imag. freq. vacuum = 0
C 4.4420298 2.1278433 -1.0429719
C 3.2723903 1.1699351 -1.1984707
C 3.6187616 -0.2664173 -0.7203974
N 2.7886899 -1.2166961 -1.2099035
O 4.5572616 -0.4989780 0.0379927
N 2.1171189 1.6511556 -0.4344907
C 0.8369319 1.3328516 -0.7388620
C -0.1952356 1.9005204 0.1958223
C -1.1601978 1.0622750 0.7780202
N -1.0777288 -0.3493968 0.5646619
C -1.8467856 -1.1570670 -0.2585259
C -3.0932052 -0.6634052 -0.9476243
F -2.8854834 0.5046747 -1.6040732
F -3.5461143 -1.5637792 -1.8352259
F -4.0968477 -0.4338068 -0.0493874
N -1.4042390 -2.3899955 -0.3475349
C -0.2609242 -2.4262640 0.4414240
C -0.0371100 -1.1523757 1.0212319
C 1.0437469 -0.8851805 1.8613152
C 1.9147990 -1.9429412 2.1069737
C 1.7098122 -3.2180784 1.5376775
C 0.6256211 -3.4792328 0.7039350
C -2.1506602 1.5808539 1.6075416

C -2.2063004 2.9554300 1.8429708
C -1.2545618 3.8001772 1.2722284
C -0.2476627 3.2724593 0.4626155
O 0.5219358 0.6370935 -1.7110841
H 4.1601454 3.1238785 -1.3950651
H 4.7574174 2.1894101 0.0023628
H 5.2938312 1.7747099 -1.6287804
H 2.9547107 1.1169046 -2.2433685
H 1.9321464 -0.9286159 -1.6700441
H 2.8127094 -2.1342873 -0.7864168
H 2.3005707 2.1212809 0.4404136
H 1.2035707 0.0953543 2.2965023
H 2.7790105 -1.7798509 2.7425780
H 2.4185019 -4.0110798 1.7564794
H 0.4664998 -4.4598880 0.2666917
H -2.8654578 0.9074637 2.0674281
H -2.9859570 3.3599565 2.4806042
H -1.2936333 4.8694143 1.4557745
H 0.4875186 3.9305882 0.0095247

42
Energy+COSMO-RS = -1363.75708 ; # imag. freq. COSMO = 0 ; # imag. freq. vacuum = 0
C -1.9644245 4.5954376 -0.3749941
C -1.2305950 3.3725775 -0.9308664
C 0.2707627 3.6942553 -1.0809717
N 1.0997488 3.1864908 -0.1462505
O 0.6405972 4.4352227 -1.9911308
N -1.5247479 2.2066351 -0.1034044
C -1.3042171 0.9350167 -0.5347575
C -1.8733377 -0.1496273 0.3429430
C -1.0704385 -1.2153056 0.7792339
N 0.3387997 -1.1641041 0.5388632
C 1.1016655 -1.8144829 -0.4177389
C 0.5527251 -2.9278419 -1.2743758
F -0.6371991 -2.6080927 -1.8349426
F 1.4065538 -3.2478268 -2.2596976
F 0.3442996 -4.0548265 -0.5291806
N 2.3519479 -1.4154639 -0.4529752
C 2.4484713 -0.4281507 0.5192908
C 1.1928558 -0.2576231 1.1548218
C 0.9863779 0.6564127 2.1893398

C	2.0909781	1.4157511	2.5745512
C	3.3455365	1.2684386	1.9452275
C	3.5430479	0.3500927	0.9174891
C	-1.6101905	-2.2705710	1.5084110
C	-2.9766764	-2.2850850	1.7933504
C	-3.7873351	-1.2310769	1.3727127
C	-3.2357940	-0.1653755	0.6595797
O	-0.7023441	0.6795885	-1.5756178
H	-3.0443743	4.4213772	-0.3724554
H	-1.6369951	4.8127978	0.6473870
H	-1.7510811	5.4646930	-0.9992933
H	-1.5748269	3.1635413	-1.9481335
H	0.7887787	2.4992971	0.5232277
H	2.0930651	3.3359208	-0.2401902
H	-2.0236417	2.3448437	0.7630956
H	0.0219257	0.7656990	2.6742684
H	1.9809662	2.1410572	3.3748501
H	4.1750194	1.8861250	2.2755736
H	4.5092237	0.2337254	0.4369462
H	-0.9592306	-3.0664530	1.8537184
H	-3.4005316	-3.1130385	2.3526934
H	-4.8496621	-1.2371147	1.5962663
H	-3.8716673	0.6460070	0.3180558

42

Energy+COSMO-RS = -1363.75609 ; # imag. freq. COSMO = 1 ; # imag. freq. vacuum = 0

C	3.2011096	-0.0730894	-0.8876906
C	2.4526408	-1.4181168	-0.8829935
C	2.7962331	-2.2239112	0.3806069
N	4.0274308	-2.7751047	0.3899147
O	2.0029384	-2.3196651	1.3181156
N	1.0138313	-1.2318305	-0.8813423
C	0.3066481	-0.8779732	-1.9740172
C	-1.1700356	-0.6577116	-1.7607114
C	-1.7005577	0.1433887	-0.7355815
N	-0.8264025	0.8193584	0.1760801
C	-0.6269901	0.6030155	1.5330142
C	-1.3990709	-0.4233904	2.3210846
F	-1.4591283	-1.6147128	1.6640421
F	-0.8372236	-0.6402892	3.5192407
F	-2.6874051	-0.0329616	2.5374469

N 0.2972040 1.3754090 2.0573187
C 0.7607238 2.1567913 1.0072862
C 0.0705119 1.8207911 -0.1843395
C 0.3367489 2.4326668 -1.4089711
C 1.3326610 3.4056755 -1.4090577
C 2.0311739 3.7542280 -0.2327954
C 1.7576289 3.1413829 0.9855901
C -3.0757778 0.3554228 -0.6318170
C -3.9430510 -0.2625806 -1.5300587
C -3.4308231 -1.0619103 -2.5539800
C -2.0550407 -1.2404193 -2.6756135
O 0.8067108 -0.7471278 -3.0958336
H 2.8886795 0.5134462 -1.7533633
H 2.9718530 0.4919972 0.0191690
H 4.2814630 -0.2322172 -0.9429894
H 2.7335899 -1.9919041 -1.7725733
H 4.6535944 -2.6952151 -0.3950948
H 4.3457293 -3.2682928 1.2106732
H 0.5580426 -1.4362692 -0.0004305
H -0.1900901 2.1571316 -2.3158117
H 1.5804879 3.9068086 -2.3398390
H 2.8007440 4.5185131 -0.2850554
H 2.2945982 3.4057027 1.8910651
H -3.4540935 1.0103271 0.1449850
H -5.0131180 -0.1042531 -1.4396550
H -4.1019730 -1.5377319 -3.2623555
H -1.6456445 -1.8420085 -3.4805675

42
Energy+COSMO-RS = -1363.75598 ; # imag. freq. COSMO = 0 ; # imag. freq. vacuum = 0
C -1.6318501 -2.2451535 -1.6158534
C -0.5833431 -2.6969880 -0.6116704
C 0.2170711 -3.9324935 -1.1053910
N 0.9566022 -4.5171734 -0.1363933
O 0.1702896 -4.3302023 -2.2675424
N 0.3320867 -1.5924385 -0.3166907
C 0.9509998 -1.4215157 0.8720275
C 1.7229408 -0.1410561 1.0331644
C 1.1693411 1.1268228 0.7856135
N -0.2012154 1.2445841 0.3861881
C -0.7358106 1.6461218 -0.8305594

C	0.1157816	2.0677287	-1.9983633
F	1.0883179	1.1383072	-2.2531782
F	-0.6175891	2.2045149	-3.1116548
F	0.7558148	3.2457557	-1.7770282
N	-2.0469353	1.5786637	-0.8764046
C	-2.4332682	1.1028640	0.3696726
C	-1.2889657	0.8817123	1.1766023
C	-1.3678468	0.3894665	2.4793244
C	-2.6464808	0.1178032	2.9591061
C	-3.7980025	0.3313305	2.1704486
C	-3.7112597	0.8248097	0.8730377
C	1.9153837	2.2852400	1.0025305
C	3.2365488	2.1880825	1.4351611
C	3.7984960	0.9349137	1.6864302
C	3.0390578	-0.2181320	1.5025866
O	0.9050568	-2.2539119	1.7874516
H	-2.1953290	-1.4000064	-1.2127666
H	-1.1661448	-1.9487881	-2.5599111
H	-2.3237127	-3.0629991	-1.8274444
H	-1.0555404	-2.9654340	0.3385097
H	1.0292493	-4.0812427	0.7764313
H	1.5838059	-5.2697688	-0.3798045
H	0.5334763	-0.9447823	-1.0642581
H	-0.4842726	0.2153675	3.0830635
H	-2.7599534	-0.2713089	3.9663529
H	-4.7727622	0.1022354	2.5903773
H	-4.5943371	0.9886949	0.2637464
H	1.4528729	3.2524797	0.8421738
H	3.8176488	3.0912817	1.5924129
H	4.8240858	0.8565163	2.0334079
H	3.4607934	-1.1958221	1.7121393

42

Energy+COSMO-RS = -1363.75506 ; # imag. freq. COSMO = 1 ; # imag. freq. vacuum = 0

C	0.9273665	-0.5631756	-2.7912134
C	0.5719252	0.8546914	-2.3311663
C	-0.1725943	1.5982197	-3.4581255
N	0.5860277	2.4523443	-4.1798979
O	-1.3600721	1.3778539	-3.6906639
N	-0.2598374	0.8052272	-1.1410886
C	-0.1186737	1.6620530	-0.1002067

C	-1.0509612	1.4294098	1.0607747
C	-1.2020424	0.1834893	1.6923455
N	-0.4375643	-0.9433189	1.2464804
C	-0.8596542	-2.0889596	0.5867456
C	-2.3047915	-2.3547877	0.2573259
F	-2.8702492	-1.2783282	-0.3689604
F	-2.4319424	-3.4126401	-0.5567245
F	-3.0537943	-2.5945124	1.3661070
N	0.1227113	-2.8930741	0.2481573
C	1.2788867	-2.2651955	0.6922146
C	0.9493645	-1.0363154	1.3174249
C	1.9113737	-0.1766549	1.8472730
C	3.2367180	-0.5868020	1.7290323
C	3.5851535	-1.8066202	1.1090052
C	2.6193854	-2.6602012	0.5858136
C	-2.0349865	0.0409434	2.8023048
C	-2.7586854	1.1357218	3.2705807
C	-2.6195050	2.3796068	2.6518202
C	-1.7574118	2.5259654	1.5676338
O	0.7127837	2.5715708	-0.0695304
H	1.4715886	-1.0861867	-2.0012540
H	0.0197741	-1.1265184	-3.0283825
H	1.5530272	-0.5256652	-3.6872786
H	1.4756861	1.4010265	-2.0546129
H	1.5342216	2.6701034	-3.9186234
H	0.1722877	2.9596514	-4.9486149
H	-1.0824569	0.2218281	-1.1784556
H	1.6458462	0.7660082	2.3126250
H	4.0230624	0.0508763	2.1215802
H	4.6337158	-2.0804392	1.0402220
H	2.8844603	-3.5977456	0.1075663
H	-2.1026759	-0.9224788	3.2948572
H	-3.4154596	1.0188285	4.1268371
H	-3.1751610	3.2368755	3.0189486
H	-1.6276590	3.4929874	1.0926354

42

Energy+COSMO-RS = -1363.75445 ; # imag. freq. COSMO = 0 ; # imag. freq. vacuum = 0

C	2.3963692	2.5697551	-2.4783412
C	1.8483828	1.2037143	-2.0513294
C	2.9001832	0.3682374	-1.2807993

N	3.2029785	0.8035594	-0.0410461
O	3.4326887	-0.6044026	-1.8151069
N	0.5603553	1.2841302	-1.3418399
C	0.2747202	1.9927640	-0.2279342
C	-1.1300498	1.8209568	0.2920339
C	-1.6158469	0.5765165	0.7245073
N	-0.7526824	-0.5669135	0.6896200
C	-0.7552611	-1.6496985	-0.1797704
C	-1.8216667	-1.8532790	-1.2218077
F	-1.9690841	-0.7344200	-2.0019740
F	-1.5250698	-2.8755410	-2.0341015
F	-3.0395538	-2.0973906	-0.6712659
N	0.2604273	-2.4641739	-0.0085805
C	1.0029124	-1.9114997	1.0265588
C	0.3838633	-0.7197179	1.4801067
C	0.9038339	0.0546941	2.5169824
C	2.0856297	-0.4017713	3.0938877
C	2.7214128	-1.5826252	2.6509642
C	2.1940980	-2.3513670	1.6186172
C	-2.8995706	0.4555705	1.2554311
C	-3.7230198	1.5776107	1.3318900
C	-3.2499175	2.8215749	0.9091619
C	-1.9556574	2.9437220	0.4068715
O	1.0754716	2.7314971	0.3589435
H	1.6566650	3.0887027	-3.0946928
H	2.6378920	3.1931404	-1.6173379
H	3.3019805	2.4196786	-3.0729737
H	1.6382535	0.6149865	-2.9449565
H	2.6817355	1.5664829	0.3770595
H	3.7973206	0.2330005	0.5419570
H	-0.1956527	0.7516441	-1.7443937
H	0.4243943	0.9697702	2.8459831
H	2.5328268	0.1683802	3.9025228
H	3.6442617	-1.8946228	3.1306482
H	2.6821469	-3.2572846	1.2742734
H	-3.2388066	-0.5106817	1.6117891
H	-4.7257119	1.4815688	1.7361721
H	-3.8864985	3.6982764	0.9773312
H	-1.5764334	3.9103409	0.0904897

42

Energy+COSMO-RS = -1363.75358

C	2.7641110	2.5400808	-2.0860879
C	2.3330071	1.1302508	-1.6829214
C	3.4647057	0.3521347	-0.9725032
N	3.0771578	-0.7503385	-0.2912154
O	4.6420487	0.6692810	-1.1211808
N	1.0733802	1.1199393	-0.9284670
C	0.9539585	1.6712187	0.3117525
C	-0.4526646	1.8048561	0.8410276
C	-1.3788422	0.7511418	0.8999579
N	-0.9933968	-0.5653896	0.4858832
C	-1.3820790	-1.2934601	-0.6286434
C	-2.3717994	-0.7708389	-1.6368251
F	-2.0211419	0.4842915	-2.0583585
F	-2.4319532	-1.5607556	-2.7169728
F	-3.6251855	-0.6696200	-1.1250947
N	-0.7844962	-2.4589632	-0.7314052
C	0.0579790	-2.5363826	0.3702256
C	-0.0566622	-1.3544845	1.1472789
C	0.6816679	-1.1403564	2.3123216
C	1.5566001	-2.1582855	2.6824702
C	1.6860824	-3.3408513	1.9214944
C	0.9445865	-3.5488284	0.7631333
C	-2.6513393	0.9434984	1.4381774
C	-3.0272589	2.2054638	1.8935637
C	-2.1164233	3.2623797	1.8447678
C	-0.8354706	3.0566977	1.3379847
O	1.9168431	2.0560479	0.9718266
H	1.9606188	3.0182986	-2.6532738
H	2.9889210	3.1422369	-1.2040958
H	3.6576204	2.4907188	-2.7105609
H	2.1246624	0.5511475	-2.5901793
H	2.1086336	-0.9115182	-0.0656687
H	3.7718049	-1.2647506	0.2284329
H	0.2265986	1.0061520	-1.4657193
H	0.5937884	-0.2258197	2.8880881
H	2.1598929	-2.0356108	3.5764813
H	2.3857363	-4.1030357	2.2503780
H	1.0459103	-4.4553930	0.1754837
H	-3.3313746	0.1017734	1.5058783
H	-4.0216741	2.3556033	2.3017480

H -2.4006269 4.2446817 2.2090576
H -0.1160068 3.8686088 1.3129214

42
Energy+COSMO-RS = -1363.75274
C 3.0632364 1.2687142 -0.7054480
C 2.2352082 0.5855994 -1.8047931
C 2.7701295 -0.8295565 -2.0694819
N 2.4537963 -1.7677193 -1.1395612
O 3.4481010 -1.0761220 -3.0635890
N 0.8098773 0.5267697 -1.4618400
C 0.0189645 1.6285876 -1.4014591
C -1.4187297 1.3839647 -1.0125795
C -1.8046933 0.6134991 0.0978395
N -0.8029069 0.0070820 0.9261130
C -0.4136897 -1.3205254 0.9874427
C -1.1496048 -2.4238032 0.2719413
F -1.3073482 -2.1310900 -1.0540223
F -0.4830404 -3.5846313 0.3554670
F -2.3940866 -2.6203952 0.7774847
N 0.6690197 -1.5176454 1.7106449
C 1.0390716 -0.2574854 2.1674111
C 0.1323806 0.7146150 1.6767041
C 0.2715437 2.0769433 1.9396986
C 1.3594854 2.4437454 2.7267067
C 2.2689559 1.4887505 3.2312484
C 2.1250810 0.1318319 2.9630459
C -3.1470816 0.4804786 0.4496605
C -4.1292623 1.0887966 -0.3299431
C -3.7629730 1.8562918 -1.4372311
C -2.4177572 2.0152057 -1.7623607
O 0.4209564 2.7680290 -1.6374807
H 2.7240857 2.2962334 -0.5699061
H 2.9651437 0.7404513 0.2459843
H 4.1170830 1.2795110 -0.9971808
H 2.3535358 1.1361516 -2.7416441
H 2.0674245 -1.5333595 -0.2352429
H 2.8587626 -2.6878622 -1.2363441
H 0.3568539 -0.3742213 -1.4857524
H -0.4190034 2.8122675 1.5419899
H 1.5150209 3.4935831 2.9550007

H 3.1023584 1.8267877 3.8393753
H 2.8259402 -0.6037062 3.3444528
H -3.4107823 -0.0899707 1.3333063
H -5.1749226 0.9755911 -0.0618888
H -4.5246208 2.3386830 -2.0419186
H -2.1228977 2.6271959 -2.6085108

42
Energy+COSMO-RS = -1363.75166 ; # imag. freq. COSMO = 0 ; # imag. freq. vacuum = 0
C 1.5434214 3.1902034 -0.3666242
C 0.1179200 3.5717335 0.0566359
C -0.5998256 4.3660959 -1.0595930
N -1.0592557 3.6421134 -2.0984728
O -0.6897808 5.5931395 -0.9822698
N -0.6522149 2.4275805 0.5711148
C -0.8512179 1.2387499 -0.0409838
C -1.5665254 0.2124381 0.7979368
C -1.0074224 -1.0621291 0.9828370
N 0.3032348 -1.3240147 0.4745903
C 0.6804537 -2.0296528 -0.6572544
C -0.2868458 -2.8724521 -1.4483861
F -1.4209659 -2.1978424 -1.7588425
F 0.2674166 -3.3023992 -2.5937119
F -0.6710641 -3.9761943 -0.7398796
N 1.9586611 -1.9281733 -0.9387108
C 2.4835854 -1.0929803 0.0398469
C 1.4587055 -0.7016533 0.9368966
C 1.6902192 0.1328725 2.0299150
C 2.9980692 0.5808833 2.2009050
C 4.0304241 0.2088320 1.3135900
C 3.7921910 -0.6298829 0.2288255
C -1.6828795 -2.0425329 1.7040831
C -2.9458768 -1.7666964 2.2302073
C -3.5140574 -0.5051520 2.0545132
C -2.8228739 0.4819402 1.3499756
O -0.5036984 0.9844528 -1.2004408
H 2.0651715 2.7187169 0.4695774
H 1.5381190 2.4969018 -1.2080386
H 2.0836902 4.0961963 -0.6551042
H 0.1752154 4.2698816 0.8926143
H -0.9051187 2.6383583 -2.1326474

H	-1.4934646	4.1173927	-2.8757987
H	-0.9769385	2.5077729	1.5227410
H	0.8958352	0.4205830	2.7103125
H	3.2282229	1.2348866	3.0366415
H	5.0337370	0.5866701	1.4857964
H	4.5862756	-0.9199249	-0.4519652
H	-1.2149710	-3.0092719	1.8541442
H	-3.4768727	-2.5345533	2.7837967
H	-4.4956562	-0.2870299	2.4636862
H	-3.2720551	1.4595927	1.2023338

42

Energy+COSMO-RS = -1363.75101 ; # imag. freq. COSMO = 0 ; # imag. freq. vacuum = 0

C	2.0945989	3.6192006	-0.2435172
C	0.6248618	3.1833154	-0.2390626
C	-0.2830381	4.4288682	-0.3115888
N	-0.9817878	4.5967886	-1.4545421
O	-0.3216562	5.2149746	0.6337879
N	0.3902657	2.2055876	-1.2966661
C	-0.2878561	1.0238997	-1.2147189
C	-1.1194728	0.7846414	0.0187710
C	-0.9272106	-0.3741190	0.7886176
N	0.1476215	-1.2608864	0.4662763
C	0.1072193	-2.4753825	-0.2005218
C	-1.1834791	-3.1833796	-0.5260755
F	-2.0967463	-2.3583054	-1.0912503
F	-0.9749466	-4.2108267	-1.3659379
F	-1.7614195	-3.6905856	0.6043355
N	1.2943385	-2.9709984	-0.4626032
C	2.1972137	-2.0404168	0.0358249
C	1.4954139	-0.9581736	0.6240944
C	2.1413124	0.1213068	1.2257485
C	3.5340789	0.0979532	1.2099492
C	4.2501737	-0.9655711	0.6201319
C	3.5981309	-2.0454252	0.0318107
C	-1.7397456	-0.6502194	1.8851554
C	-2.7836484	0.2168975	2.2093548
C	-2.9966378	1.3642240	1.4457078
C	-2.1631642	1.6495327	0.3640926
O	-0.2501813	0.1939142	-2.1223584
H	2.7398685	2.7504942	-0.0941013

H	2.3521924	4.0900985	-1.1981685
H	2.2694725	4.3398068	0.5571826
H	0.3914152	2.7140083	0.7188890
H	-0.9635862	3.9033116	-2.1853989
H	-1.5916790	5.3945812	-1.5527876
H	0.9622302	2.2862575	-2.1291451
H	1.5915229	0.9302927	1.6937458
H	4.0821027	0.9155073	1.6686177
H	5.3356389	-0.9398647	0.6327639
H	4.1477278	-2.8678844	-0.4150467
H	-1.5491056	-1.5397475	2.4754796
H	-3.4219992	-0.0031381	3.0590931
H	-3.8083624	2.0416819	1.6916377
H	-2.3382399	2.5399255	-0.2301933

42

Energy+COSMO-RS = -1363.75051

C	-1.8606136	-0.1525018	-3.5048407
C	-0.9011197	-0.0362030	-2.3151036
C	0.3946855	-0.8286059	-2.5847658
N	1.5567589	-0.1567163	-2.4195438
O	0.3349007	-2.0016542	-2.9449220
N	-0.6545406	1.3603187	-1.9751152
C	-1.1483047	2.0543084	-0.9154737
C	-1.7673184	1.3016126	0.2374685
C	-1.0950024	0.4113031	1.0926048
N	0.2740387	0.0574840	0.8710132
C	0.8427828	-1.2101435	0.7559153
C	0.0680841	-2.5076631	0.7656372
F	-1.1363167	-2.3990554	0.1497832
F	0.7586672	-3.4823128	0.1554319
F	-0.1794715	-2.9283853	2.0408371
N	2.1520119	-1.1892645	0.6692726
C	2.5085348	0.1491277	0.7374690
C	1.3476208	0.9515698	0.8745130
C	1.4087240	2.3393715	1.0080329
C	2.6784370	2.9104415	0.9646222
C	3.8423541	2.1287862	0.8033943
C	3.7764142	0.7440411	0.6927458
C	-1.7401915	-0.1122637	2.2162676
C	-3.0736805	0.1942838	2.4682431

C	-3.7601289	1.0594951	1.6148954
C	-3.1012828	1.6230876	0.5266748
O	-1.1230572	3.2866105	-0.8854898
H	-2.8186794	0.3097413	-3.2532163
H	-1.4453933	0.3526244	-4.3830522
H	-2.0206659	-1.2033305	-3.7514018
H	-1.3638950	-0.5068427	-1.4456518
H	1.5716008	0.7502333	-1.9800962
H	2.4220505	-0.6738498	-2.4695918
H	-0.3580681	1.9609548	-2.7366358
H	0.5211631	2.9482642	1.1288234
H	2.7728928	3.9878736	1.0595808
H	4.8089692	2.6224836	0.7738620
H	4.6684492	0.1356041	0.5826389
H	-1.1870603	-0.7537062	2.8920576
H	-3.5667949	-0.2285965	3.3377198
H	-4.7988106	1.3097836	1.8064063
H	-3.6165528	2.3257263	-0.1200702

42

Energy+COSMO-RS = -1363.75026

C	1.5858750	3.3484180	-0.0636502
C	0.0840422	3.6053602	0.0912253
C	-0.6916641	4.0224280	-1.1845556
N	0.0488278	4.2951006	-2.2837797
O	-1.9107177	4.1745070	-1.1436075
N	-0.5977795	2.4734325	0.7119137
C	-0.8047391	1.3080539	0.0564351
C	-1.5408956	0.2580192	0.8462636
C	-1.0052620	-1.0321039	0.9842036
N	0.3037615	-1.2974348	0.4715768
C	0.6724449	-1.9758422	-0.6792548
C	-0.3049374	-2.7798611	-1.4980913
F	-1.4254404	-2.0780623	-1.7952210
F	0.2486988	-3.1869138	-2.6524817
F	-0.7107458	-3.8973027	-0.8236185
N	1.9522258	-1.8833663	-0.9580819
C	2.4870388	-1.0820100	0.0428890
C	1.4665509	-0.7019568	0.9498076
C	1.7080284	0.1030927	2.0625693
C	3.0204824	0.5324016	2.2436671

C	4.0488441	0.1702108	1.3477632
C	3.8010687	-0.6392522	0.2430524
C	-1.7029210	-2.0292173	1.6601838
C	-2.9664896	-1.7531309	2.1850966
C	-3.5134498	-0.4768368	2.0527897
C	-2.7984588	0.5260573	1.3958856
O	-0.4315156	1.1100486	-1.1005292
H	2.0118499	3.0877797	0.9079335
H	1.7767555	2.5173035	-0.7459886
H	2.0961856	4.2456174	-0.4260007
H	-0.0588305	4.4479480	0.7757146
H	1.0013059	3.9782103	-2.3639036
H	-0.4408582	4.5106766	-3.1401957
H	-0.9327253	2.5686821	1.6573281
H	0.9165709	0.3844283	2.7488270
H	3.2578639	1.1633736	3.0950366
H	5.0566337	0.5316852	1.5292946
H	4.5917557	-0.9219878	-0.4448458
H	-1.2529969	-3.0091140	1.7758074
H	-3.5153357	-2.5335559	2.7026806
H	-4.4963164	-0.2597134	2.4595699
H	-3.2285174	1.5165257	1.2797330

42

Energy+COSMO-RS = -1363.74985

C	-1.7972906	0.0133382	-3.3333716
C	-0.6644443	-0.0354372	-2.2928872
C	0.5278281	-0.8339809	-2.8499406
N	0.4210108	-2.1745805	-2.7345761
O	1.4735360	-0.2719781	-3.3987218
N	-0.1933988	1.2941673	-1.9496686
C	-0.6769151	2.1119355	-0.9894123
C	-1.5540927	1.5218697	0.0911739
C	-1.1142121	0.5966401	1.0551117
N	0.2073745	0.0488519	0.9957519
C	0.6017534	-1.2822467	0.8753285
C	-0.3378624	-2.4612110	0.8896543
F	-1.4659534	-2.2435450	0.1516310
F	0.2584785	-3.5588935	0.3929773
F	-0.7544346	-2.7638441	2.1495439
N	1.9045249	-1.4396018	0.8258887

C 2.4364491 -0.1625974 0.9261586
C 1.3914387 0.7871218 1.0454902
C 1.6339719 2.1504021 1.2167555
C 2.9692679 2.5429844 1.2345155
C 4.0223275 1.6131283 1.0943094
C 3.7744602 0.2538184 0.9424812
C -1.9490400 0.2228292 2.1108482
C -3.2472298 0.7199366 2.1874324
C -3.7039407 1.6220832 1.2253407
C -2.8533134 2.0331623 0.2024517
O -0.4234895 3.3197200 -0.9616254
H -2.6386489 0.5837507 -2.9303419
H -1.4487880 0.4982212 -4.2497867
H -2.1425452 -0.9950539 -3.5784851
H -1.0381531 -0.5125423 -1.3887130
H -0.3580969 -2.6138108 -2.2722617
H 1.1438529 -2.7663299 -3.1166468
H 0.4289431 1.7233914 -2.6257506
H 0.8314691 2.8708331 1.3155037
H 3.2058515 3.5951574 1.3605876
H 5.0472363 1.9713622 1.1122914
H 4.5795879 -0.4676925 0.8470842
H -1.5717984 -0.4505540 2.8714686
H -3.8914932 0.4152040 3.0060931
H -4.7128789 2.0186942 1.2814167
H -3.1883247 2.7630977 -0.5276418

(*M*)-atropisomer in DMSO (13 structures)

42
Energy+COSMO-RS = -1363.76052 ; # imag. freq. COSMO = 1 ; # imag. freq. vacuum = 0
C 3.2011096 -0.0730894 -0.8876906
C 2.4526408 -1.4181168 -0.8829935
C 2.7962331 -2.2239112 0.3806069
N 4.0274308 -2.7751047 0.3899147
O 2.0029384 -2.3196651 1.3181156
N 1.0138313 -1.2318305 -0.8813423
C 0.3066481 -0.8779732 -1.9740172
C -1.1700356 -0.6577116 -1.7607114

C	-1.7005577	0.1433887	-0.7355815
N	-0.8264025	0.8193584	0.1760801
C	-0.6269901	0.6030155	1.5330142
C	-1.3990709	-0.4233904	2.3210846
F	-1.4591283	-1.6147128	1.6640421
F	-0.8372236	-0.6402892	3.5192407
F	-2.6874051	-0.0329616	2.5374469
N	0.2972040	1.3754090	2.0573187
C	0.7607238	2.1567913	1.0072862
C	0.0705119	1.8207911	-0.1843395
C	0.3367489	2.4326668	-1.4089711
C	1.3326610	3.4056755	-1.4090577
C	2.0311739	3.7542280	-0.2327954
C	1.7576289	3.1413829	0.9855901
C	-3.0757778	0.3554228	-0.6318170
C	-3.9430510	-0.2625806	-1.5300587
C	-3.4308231	-1.0619103	-2.5539800
C	-2.0550407	-1.2404193	-2.6756135
O	0.8067108	-0.7471278	-3.0958336
H	2.8886795	0.5134462	-1.7533633
H	2.9718530	0.4919972	0.0191690
H	4.2814630	-0.2322172	-0.9429894
H	2.7335899	-1.9919041	-1.7725733
H	4.6535944	-2.6952151	-0.3950948
H	4.3457293	-3.2682928	1.2106732
H	0.5580426	-1.4362692	-0.0004305
H	-0.1900901	2.1571316	-2.3158117
H	1.5804879	3.9068086	-2.3398390
H	2.8007440	4.5185131	-0.2850554
H	2.2945982	3.4057027	1.8910651
H	-3.4540935	1.0103271	0.1449850
H	-5.0131180	-0.1042531	-1.4396550
H	-4.1019730	-1.5377319	-3.2623555
H	-1.6456445	-1.8420085	-3.4805675

42

Energy+COSMO-RS = -1363.75800 ; # imag. freq. COSMO = 1 ; # imag. freq. vacuum = 0

C	0.9273665	-0.5631756	-2.7912134
C	0.5719252	0.8546914	-2.3311663
C	-0.1725943	1.5982197	-3.4581255
N	0.5860277	2.4523443	-4.1798979

O	-1.3600721	1.3778539	-3.6906639
N	-0.2598374	0.8052272	-1.1410886
C	-0.1186737	1.6620530	-0.1002067
C	-1.0509612	1.4294098	1.0607747
C	-1.2020424	0.1834893	1.6923455
N	-0.4375643	-0.9433189	1.2464804
C	-0.8596542	-2.0889596	0.5867456
C	-2.3047915	-2.3547877	0.2573259
F	-2.8702492	-1.2783282	-0.3689604
F	-2.4319424	-3.4126401	-0.5567245
F	-3.0537943	-2.5945124	1.3661070
N	0.1227113	-2.8930741	0.2481573
C	1.2788867	-2.2651955	0.6922146
C	0.9493645	-1.0363154	1.3174249
C	1.9113737	-0.1766549	1.8472730
C	3.2367180	-0.5868020	1.7290323
C	3.5851535	-1.8066202	1.1090052
C	2.6193854	-2.6602012	0.5858136
C	-2.0349865	0.0409434	2.8023048
C	-2.7586854	1.1357218	3.2705807
C	-2.6195050	2.3796068	2.6518202
C	-1.7574118	2.5259654	1.5676338
O	0.7127837	2.5715708	-0.0695304
H	1.4715886	-1.0861867	-2.0012540
H	0.0197741	-1.1265184	-3.0283825
H	1.5530272	-0.5256652	-3.6872786
H	1.4756861	1.4010265	-2.0546129
H	1.5342216	2.6701034	-3.9186234
H	0.1722877	2.9596514	-4.9486149
H	-1.0824569	0.2218281	-1.1784556
H	1.6458462	0.7660082	2.3126250
H	4.0230624	0.0508763	2.1215802
H	4.6337158	-2.0804392	1.0402220
H	2.8844603	-3.5977456	0.1075663
H	-2.1026759	-0.9224788	3.2948572
H	-3.4154596	1.0188285	4.1268371
H	-3.1751610	3.2368755	3.0189486
H	-1.6276590	3.4929874	1.0926354

42

Energy+COSMO-RS = -1363.75682 ; # imag. freq. COSMO = 0 ; # imag. freq. vacuum = 0

C	4.4420298	2.1278433	-1.0429719
C	3.2723903	1.1699351	-1.1984707
C	3.6187616	-0.2664173	-0.7203974
N	2.7886899	-1.2166961	-1.2099035
O	4.5572616	-0.4989780	0.0379927
N	2.1171189	1.6511556	-0.4344907
C	0.8369319	1.3328516	-0.7388620
C	-0.1952356	1.9005204	0.1958223
C	-1.1601978	1.0622750	0.7780202
N	-1.0777288	-0.3493968	0.5646619
C	-1.8467856	-1.1570670	-0.2585259
C	-3.0932052	-0.6634052	-0.9476243
F	-2.8854834	0.5046747	-1.6040732
F	-3.5461143	-1.5637792	-1.8352259
F	-4.0968477	-0.4338068	-0.0493874
N	-1.4042390	-2.3899955	-0.3475349
C	-0.2609242	-2.4262640	0.4414240
C	-0.0371100	-1.1523757	1.0212319
C	1.0437469	-0.8851805	1.8613152
C	1.9147990	-1.9429412	2.1069737
C	1.7098122	-3.2180784	1.5376775
C	0.6256211	-3.4792328	0.7039350
C	-2.1506602	1.5808539	1.6075416
C	-2.2063004	2.9554300	1.8429708
C	-1.2545618	3.8001772	1.2722284
C	-0.2476627	3.2724593	0.4626155
O	0.5219358	0.6370935	-1.7110841
H	4.1601454	3.1238785	-1.3950651
H	4.7574174	2.1894101	0.0023628
H	5.2938312	1.7747099	-1.6287804
H	2.9547107	1.1169046	-2.2433685
H	1.9321464	-0.9286159	-1.6700441
H	2.8127094	-2.1342873	-0.7864168
H	2.3005707	2.1212809	0.4404136
H	1.2035707	0.0953543	2.2965023
H	2.7790105	-1.7798509	2.7425780
H	2.4185019	-4.0110798	1.7564794
H	0.4664998	-4.4598880	0.2666917
H	-2.8654578	0.9074637	2.0674281
H	-2.9859570	3.3599565	2.4806042
H	-1.2936333	4.8694143	1.4557745

H 0.4875186 3.9305882 0.0095247

42

Energy+COSMO-RS = -1363.75657 ; # imag. freq. COSMO = 0 ; # imag. freq. vacuum = 0

C 2.0945989 3.6192006 -0.2435172

C 0.6248618 3.1833154 -0.2390626

C -0.2830381 4.4288682 -0.3115888

N -0.9817878 4.5967886 -1.4545421

O -0.3216562 5.2149746 0.6337879

N 0.3902657 2.2055876 -1.2966661

C -0.2878561 1.0238997 -1.2147189

C -1.1194728 0.7846414 0.0187710

C -0.9272106 -0.3741190 0.7886176

N 0.1476215 -1.2608864 0.4662763

C 0.1072193 -2.4753825 -0.2005218

C -1.1834791 -3.1833796 -0.5260755

F -2.0967463 -2.3583054 -1.0912503

F -0.9749466 -4.2108267 -1.3659379

F -1.7614195 -3.6905856 0.6043355

N 1.2943385 -2.9709984 -0.4626032

C 2.1972137 -2.0404168 0.0358249

C 1.4954139 -0.9581736 0.6240944

C 2.1413124 0.1213068 1.2257485

C 3.5340789 0.0979532 1.2099492

C 4.2501737 -0.9655711 0.6201319

C 3.5981309 -2.0454252 0.0318107

C -1.7397456 -0.6502194 1.8851554

C -2.7836484 0.2168975 2.2093548

C -2.9966378 1.3642240 1.4457078

C -2.1631642 1.6495327 0.3640926

O -0.2501813 0.1939142 -2.1223584

H 2.7398685 2.7504942 -0.0941013

H 2.3521924 4.0900985 -1.1981685

H 2.2694725 4.3398068 0.5571826

H 0.3914152 2.7140083 0.7188890

H -0.9635862 3.9033116 -2.1853989

H -1.5916790 5.3945812 -1.5527876

H 0.9622302 2.2862575 -2.1291451

H 1.5915229 0.9302927 1.6937458

H 4.0821027 0.9155073 1.6686177

H 5.3356389 -0.9398647 0.6327639

H	4.1477278	-2.8678844	-0.4150467
H	-1.5491056	-1.5397475	2.4754796
H	-3.4219992	-0.0031381	3.0590931
H	-3.8083624	2.0416819	1.6916377
H	-2.3382399	2.5399255	-0.2301933

42

Energy+COSMO-RS = -1363.75646 ; # imag. freq. COSMO = 0 ; # imag. freq. vacuum = 0

C	-1.9644245	4.5954376	-0.3749941
C	-1.2305950	3.3725775	-0.9308664
C	0.2707627	3.6942553	-1.0809717
N	1.0997488	3.1864908	-0.1462505
O	0.6405972	4.4352227	-1.9911308
N	-1.5247479	2.2066351	-0.1034044
C	-1.3042171	0.9350167	-0.5347575
C	-1.8733377	-0.1496273	0.3429430
C	-1.0704385	-1.2153056	0.7792339
N	0.3387997	-1.1641041	0.5388632
C	1.1016655	-1.8144829	-0.4177389
C	0.5527251	-2.9278419	-1.2743758
F	-0.6371991	-2.6080927	-1.8349426
F	1.4065538	-3.2478268	-2.2596976
F	0.3442996	-4.0548265	-0.5291806
N	2.3519479	-1.4154639	-0.4529752
C	2.4484713	-0.4281507	0.5192908
C	1.1928558	-0.2576231	1.1548218
C	0.9863779	0.6564127	2.1893398
C	2.0909781	1.4157511	2.5745512
C	3.3455365	1.2684386	1.9452275
C	3.5430479	0.3500927	0.9174891
C	-1.6101905	-2.2705710	1.5084110
C	-2.9766764	-2.2850850	1.7933504
C	-3.7873351	-1.2310769	1.3727127
C	-3.2357940	-0.1653755	0.6595797
O	-0.7023441	0.6795885	-1.5756178
H	-3.0443743	4.4213772	-0.3724554
H	-1.6369951	4.8127978	0.6473870
H	-1.7510811	5.4646930	-0.9992933
H	-1.5748269	3.1635413	-1.9481335
H	0.7887787	2.4992971	0.5232277
H	2.0930651	3.3359208	-0.2401902

H	-2.0236417	2.3448437	0.7630956
H	0.0219257	0.7656990	2.6742684
H	1.9809662	2.1410572	3.3748501
H	4.1750194	1.8861250	2.2755736
H	4.5092237	0.2337254	0.4369462
H	-0.9592306	-3.0664530	1.8537184
H	-3.4005316	-3.1130385	2.3526934
H	-4.8496621	-1.2371147	1.5962663
H	-3.8716673	0.6460070	0.3180558

42

Energy+COSMO-RS = -1363.75613

C	1.5858750	3.3484180	-0.0636502
C	0.0840422	3.6053602	0.0912253
C	-0.6916641	4.0224280	-1.1845556
N	0.0488278	4.2951006	-2.2837797
O	-1.9107177	4.1745070	-1.1436075
N	-0.5977795	2.4734325	0.7119137
C	-0.8047391	1.3080539	0.0564351
C	-1.5408956	0.2580192	0.8462636
C	-1.0052620	-1.0321039	0.9842036
N	0.3037615	-1.2974348	0.4715768
C	0.6724449	-1.9758422	-0.6792548
C	-0.3049374	-2.7798611	-1.4980913
F	-1.4254404	-2.0780623	-1.7952210
F	0.2486988	-3.1869138	-2.6524817
F	-0.7107458	-3.8973027	-0.8236185
N	1.9522258	-1.8833663	-0.9580819
C	2.4870388	-1.0820100	0.0428890
C	1.4665509	-0.7019568	0.9498076
C	1.7080284	0.1030927	2.0625693
C	3.0204824	0.5324016	2.2436671
C	4.0488441	0.1702108	1.3477632
C	3.8010687	-0.6392522	0.2430524
C	-1.7029210	-2.0292173	1.6601838
C	-2.9664896	-1.7531309	2.1850966
C	-3.5134498	-0.4768368	2.0527897
C	-2.7984588	0.5260573	1.3958856
O	-0.4315156	1.1100486	-1.1005292
H	2.0118499	3.0877797	0.9079335
H	1.7767555	2.5173035	-0.7459886

H	2.0961856	4.2456174	-0.4260007
H	-0.0588305	4.4479480	0.7757146
H	1.0013059	3.9782103	-2.3639036
H	-0.4408582	4.5106766	-3.1401957
H	-0.9327253	2.5686821	1.6573281
H	0.9165709	0.3844283	2.7488270
H	3.2578639	1.1633736	3.0950366
H	5.0566337	0.5316852	1.5292946
H	4.5917557	-0.9219878	-0.4448458
H	-1.2529969	-3.0091140	1.7758074
H	-3.5153357	-2.5335559	2.7026806
H	-4.4963164	-0.2597134	2.4595699
H	-3.2285174	1.5165257	1.2797330

42

Energy+COSMO-RS = -1363.75459 ; # imag. freq. COSMO = 0 ; # imag. freq. vacuum = 0

C	1.5434214	3.1902034	-0.3666242
C	0.1179200	3.5717335	0.0566359
C	-0.5998256	4.3660959	-1.0595930
N	-1.0592557	3.6421134	-2.0984728
O	-0.6897808	5.5931395	-0.9822698
N	-0.6522149	2.4275805	0.5711148
C	-0.8512179	1.2387499	-0.0409838
C	-1.5665254	0.2124381	0.7979368
C	-1.0074224	-1.0621291	0.9828370
N	0.3032348	-1.3240147	0.4745903
C	0.6804537	-2.0296528	-0.6572544
C	-0.2868458	-2.8724521	-1.4483861
F	-1.4209659	-2.1978424	-1.7588425
F	0.2674166	-3.3023992	-2.5937119
F	-0.6710641	-3.9761943	-0.7398796
N	1.9586611	-1.9281733	-0.9387108
C	2.4835854	-1.0929803	0.0398469
C	1.4587055	-0.7016533	0.9368966
C	1.6902192	0.1328725	2.0299150
C	2.9980692	0.5808833	2.2009050
C	4.0304241	0.2088320	1.3135900
C	3.7921910	-0.6298829	0.2288255
C	-1.6828795	-2.0425329	1.7040831
C	-2.9458768	-1.7666964	2.2302073
C	-3.5140574	-0.5051520	2.0545132

C -2.8228739 0.4819402 1.3499756
O -0.5036984 0.9844528 -1.2004408
H 2.0651715 2.7187169 0.4695774
H 1.5381190 2.4969018 -1.2080386
H 2.0836902 4.0961963 -0.6551042
H 0.1752154 4.2698816 0.8926143
H -0.9051187 2.6383583 -2.1326474
H -1.4934646 4.1173927 -2.8757987
H -0.9769385 2.5077729 1.5227410
H 0.8958352 0.4205830 2.7103125
H 3.2282229 1.2348866 3.0366415
H 5.0337370 0.5866701 1.4857964
H 4.5862756 -0.9199249 -0.4519652
H -1.2149710 -3.0092719 1.8541442
H -3.4768727 -2.5345533 2.7837967
H -4.4956562 -0.2870299 2.4636862
H -3.2720551 1.4595927 1.2023338

42
Energy+COSMO-RS = -1363.75445 ; # imag. freq. COSMO = 0 ; # imag. freq. vacuum = 0
C -1.6318501 -2.2451535 -1.6158534
C -0.5833431 -2.6969880 -0.6116704
C 0.2170711 -3.9324935 -1.1053910
N 0.9566022 -4.5171734 -0.1363933
O 0.1702896 -4.3302023 -2.2675424
N 0.3320867 -1.5924385 -0.3166907
C 0.9509998 -1.4215157 0.8720275
C 1.7229408 -0.1410561 1.0331644
C 1.1693411 1.1268228 0.7856135
N -0.2012154 1.2445841 0.3861881
C -0.7358106 1.6461218 -0.8305594
C 0.1157816 2.0677287 -1.9983633
F 1.0883179 1.1383072 -2.2531782
F -0.6175891 2.2045149 -3.1116548
F 0.7558148 3.2457557 -1.7770282
N -2.0469353 1.5786637 -0.8764046
C -2.4332682 1.1028640 0.3696726
C -1.2889657 0.8817123 1.1766023
C -1.3678468 0.3894665 2.4793244
C -2.6464808 0.1178032 2.9591061
C -3.7980025 0.3313305 2.1704486

C	-3.7112597	0.8248097	0.8730377
C	1.9153837	2.2852400	1.0025305
C	3.2365488	2.1880825	1.4351611
C	3.7984960	0.9349137	1.6864302
C	3.0390578	-0.2181320	1.5025866
O	0.9050568	-2.2539119	1.7874516
H	-2.1953290	-1.4000064	-1.2127666
H	-1.1661448	-1.9487881	-2.5599111
H	-2.3237127	-3.0629991	-1.8274444
H	-1.0555404	-2.9654340	0.3385097
H	1.0292493	-4.0812427	0.7764313
H	1.5838059	-5.2697688	-0.3798045
H	0.5334763	-0.9447823	-1.0642581
H	-0.4842726	0.2153675	3.0830635
H	-2.7599534	-0.2713089	3.9663529
H	-4.7727622	0.1022354	2.5903773
H	-4.5943371	0.9886949	0.2637464
H	1.4528729	3.2524797	0.8421738
H	3.8176488	3.0912817	1.5924129
H	4.8240858	0.8565163	2.0334079
H	3.4607934	-1.1958221	1.7121393

42

Energy+COSMO-RS = -1363.75346

C	-1.8606136	-0.1525018	-3.5048407
C	-0.9011197	-0.0362030	-2.3151036
C	0.3946855	-0.8286059	-2.5847658
N	1.5567589	-0.1567163	-2.4195438
O	0.3349007	-2.0016542	-2.9449220
N	-0.6545406	1.3603187	-1.9751152
C	-1.1483047	2.0543084	-0.9154737
C	-1.7673184	1.3016126	0.2374685
C	-1.0950024	0.4113031	1.0926048
N	0.2740387	0.0574840	0.8710132
C	0.8427828	-1.2101435	0.7559153
C	0.0680841	-2.5076631	0.7656372
F	-1.1363167	-2.3990554	0.1497832
F	0.7586672	-3.4823128	0.1554319
F	-0.1794715	-2.9283853	2.0408371
N	2.1520119	-1.1892645	0.6692726
C	2.5085348	0.1491277	0.7374690

C	1.3476208	0.9515698	0.8745130
C	1.4087240	2.3393715	1.0080329
C	2.6784370	2.9104415	0.9646222
C	3.8423541	2.1287862	0.8033943
C	3.7764142	0.7440411	0.6927458
C	-1.7401915	-0.1122637	2.2162676
C	-3.0736805	0.1942838	2.4682431
C	-3.7601289	1.0594951	1.6148954
C	-3.1012828	1.6230876	0.5266748
O	-1.1230572	3.2866105	-0.8854898
H	-2.8186794	0.3097413	-3.2532163
H	-1.4453933	0.3526244	-4.3830522
H	-2.0206659	-1.2033305	-3.7514018
H	-1.3638950	-0.5068427	-1.4456518
H	1.5716008	0.7502333	-1.9800962
H	2.4220505	-0.6738498	-2.4695918
H	-0.3580681	1.9609548	-2.7366358
H	0.5211631	2.9482642	1.1288234
H	2.7728928	3.9878736	1.0595808
H	4.8089692	2.6224836	0.7738620
H	4.6684492	0.1356041	0.5826389
H	-1.1870603	-0.7537062	2.8920576
H	-3.5667949	-0.2285965	3.3377198
H	-4.7988106	1.3097836	1.8064063
H	-3.6165528	2.3257263	-0.1200702

42

Energy+COSMO-RS = -1363.75196

C	-1.7972906	0.0133382	-3.3333716
C	-0.6644443	-0.0354372	-2.2928872
C	0.5278281	-0.8339809	-2.8499406
N	0.4210108	-2.1745805	-2.7345761
O	1.4735360	-0.2719781	-3.3987218
N	-0.1933988	1.2941673	-1.9496686
C	-0.6769151	2.1119355	-0.9894123
C	-1.5540927	1.5218697	0.0911739
C	-1.1142121	0.5966401	1.0551117
N	0.2073745	0.0488519	0.9957519
C	0.6017534	-1.2822467	0.8753285
C	-0.3378624	-2.4612110	0.8896543
F	-1.4659534	-2.2435450	0.1516310

F	0.2584785	-3.5588935	0.3929773
F	-0.7544346	-2.7638441	2.1495439
N	1.9045249	-1.4396018	0.8258887
C	2.4364491	-0.1625974	0.9261586
C	1.3914387	0.7871218	1.0454902
C	1.6339719	2.1504021	1.2167555
C	2.9692679	2.5429844	1.2345155
C	4.0223275	1.6131283	1.0943094
C	3.7744602	0.2538184	0.9424812
C	-1.9490400	0.2228292	2.1108482
C	-3.2472298	0.7199366	2.1874324
C	-3.7039407	1.6220832	1.2253407
C	-2.8533134	2.0331623	0.2024517
O	-0.4234895	3.3197200	-0.9616254
H	-2.6386489	0.5837507	-2.9303419
H	-1.4487880	0.4982212	-4.2497867
H	-2.1425452	-0.9950539	-3.5784851
H	-1.0381531	-0.5125423	-1.3887130
H	-0.3580969	-2.6138108	-2.2722617
H	1.1438529	-2.7663299	-3.1166468
H	0.4289431	1.7233914	-2.6257506
H	0.8314691	2.8708331	1.3155037
H	3.2058515	3.5951574	1.3605876
H	5.0472363	1.9713622	1.1122914
H	4.5795879	-0.4676925	0.8470842
H	-1.5717984	-0.4505540	2.8714686
H	-3.8914932	0.4152040	3.0060931
H	-4.7128789	2.0186942	1.2814167
H	-3.1883247	2.7630977	-0.5276418

42

Energy+COSMO-RS = -1363.75155

C	2.7641110	2.5400808	-2.0860879
C	2.3330071	1.1302508	-1.6829214
C	3.4647057	0.3521347	-0.9725032
N	3.0771578	-0.7503385	-0.2912154
O	4.6420487	0.6692810	-1.1211808
N	1.0733802	1.1199393	-0.9284670
C	0.9539585	1.6712187	0.3117525
C	-0.4526646	1.8048561	0.8410276
C	-1.3788422	0.7511418	0.8999579

N -0.9933968 -0.5653896 0.4858832
C -1.3820790 -1.2934601 -0.6286434
C -2.3717994 -0.7708389 -1.6368251
F -2.0211419 0.4842915 -2.0583585
F -2.4319532 -1.5607556 -2.7169728
F -3.6251855 -0.6696200 -1.1250947
N -0.7844962 -2.4589632 -0.7314052
C 0.0579790 -2.5363826 0.3702256
C -0.0566622 -1.3544845 1.1472789
C 0.6816679 -1.1403564 2.3123216
C 1.5566001 -2.1582855 2.6824702
C 1.6860824 -3.3408513 1.9214944
C 0.9445865 -3.5488284 0.7631333
C -2.6513393 0.9434984 1.4381774
C -3.0272589 2.2054638 1.8935637
C -2.1164233 3.2623797 1.8447678
C -0.8354706 3.0566977 1.3379847
O 1.9168431 2.0560479 0.9718266
H 1.9606188 3.0182986 -2.6532738
H 2.9889210 3.1422369 -1.2040958
H 3.6576204 2.4907188 -2.7105609
H 2.1246624 0.5511475 -2.5901793
H 2.1086336 -0.9115182 -0.0656687
H 3.7718049 -1.2647506 0.2284329
H 0.2265986 1.0061520 -1.4657193
H 0.5937884 -0.2258197 2.8880881
H 2.1598929 -2.0356108 3.5764813
H 2.3857363 -4.1030357 2.2503780
H 1.0459103 -4.4553930 0.1754837
H -3.3313746 0.1017734 1.5058783
H -4.0216741 2.3556033 2.3017480
H -2.4006269 4.2446817 2.2090576
H -0.1160068 3.8686088 1.3129214

42
Energy+COSMO-RS = -1363.75145
C 3.0632364 1.2687142 -0.7054480
C 2.2352082 0.5855994 -1.8047931
C 2.7701295 -0.8295565 -2.0694819
N 2.4537963 -1.7677193 -1.1395612
O 3.4481010 -1.0761220 -3.0635890

N	0.8098773	0.5267697	-1.4618400
C	0.0189645	1.6285876	-1.4014591
C	-1.4187297	1.3839647	-1.0125795
C	-1.8046933	0.6134991	0.0978395
N	-0.8029069	0.0070820	0.9261130
C	-0.4136897	-1.3205254	0.9874427
C	-1.1496048	-2.4238032	0.2719413
F	-1.3073482	-2.1310900	-1.0540223
F	-0.4830404	-3.5846313	0.3554670
F	-2.3940866	-2.6203952	0.7774847
N	0.6690197	-1.5176454	1.7106449
C	1.0390716	-0.2574854	2.1674111
C	0.1323806	0.7146150	1.6767041
C	0.2715437	2.0769433	1.9396986
C	1.3594854	2.4437454	2.7267067
C	2.2689559	1.4887505	3.2312484
C	2.1250810	0.1318319	2.9630459
C	-3.1470816	0.4804786	0.4496605
C	-4.1292623	1.0887966	-0.3299431
C	-3.7629730	1.8562918	-1.4372311
C	-2.4177572	2.0152057	-1.7623607
O	0.4209564	2.7680290	-1.6374807
H	2.7240857	2.2962334	-0.5699061
H	2.9651437	0.7404513	0.2459843
H	4.1170830	1.2795110	-0.9971808
H	2.3535358	1.1361516	-2.7416441
H	2.0674245	-1.5333595	-0.2352429
H	2.8587626	-2.6878622	-1.2363441
H	0.3568539	-0.3742213	-1.4857524
H	-0.4190034	2.8122675	1.5419899
H	1.5150209	3.4935831	2.9550007
H	3.1023584	1.8267877	3.8393753
H	2.8259402	-0.6037062	3.3444528
H	-3.4107823	-0.0899707	1.3333063
H	-5.1749226	0.9755911	-0.0618888
H	-4.5246208	2.3386830	-2.0419186
H	-2.1228977	2.6271959	-2.6085108

42

Energy+COSMO-RS = -1363.75070 ; # imag. freq. COSMO = 0 ; # imag. freq. vacuum = 0

C	2.3963692	2.5697551	-2.4783412

C	1.8483828	1.2037143	-2.0513294
C	2.9001832	0.3682374	-1.2807993
N	3.2029785	0.8035594	-0.0410461
O	3.4326887	-0.6044026	-1.8151069
N	0.5603553	1.2841302	-1.3418399
C	0.2747202	1.9927640	-0.2279342
C	-1.1300498	1.8209568	0.2920339
C	-1.6158469	0.5765165	0.7245073
N	-0.7526824	-0.5669135	0.6896200
C	-0.7552611	-1.6496985	-0.1797704
C	-1.8216667	-1.8532790	-1.2218077
F	-1.9690841	-0.7344200	-2.0019740
F	-1.5250698	-2.8755410	-2.0341015
F	-3.0395538	-2.0973906	-0.6712659
N	0.2604273	-2.4641739	-0.0085805
C	1.0029124	-1.9114997	1.0265588
C	0.3838633	-0.7197179	1.4801067
C	0.9038339	0.0546941	2.5169824
C	2.0856297	-0.4017713	3.0938877
C	2.7214128	-1.5826252	2.6509642
C	2.1940980	-2.3513670	1.6186172
C	-2.8995706	0.4555705	1.2554311
C	-3.7230198	1.5776107	1.3318900
C	-3.2499175	2.8215749	0.9091619
C	-1.9556574	2.9437220	0.4068715
O	1.0754716	2.7314971	0.3589435
H	1.6566650	3.0887027	-3.0946928
H	2.6378920	3.1931404	-1.6173379
H	3.3019805	2.4196786	-3.0729737
H	1.6382535	0.6149865	-2.9449565
H	2.6817355	1.5664829	0.3770595
H	3.7973206	0.2330005	0.5419570
H	-0.1956527	0.7516441	-1.7443937
H	0.4243943	0.9697702	2.8459831
H	2.5328268	0.1683802	3.9025228
H	3.6442617	-1.8946228	3.1306482
H	2.6821469	-3.2572846	1.2742734
H	-3.2388066	-0.5106817	1.6117891
H	-4.7257119	1.4815688	1.7361721
H	-3.8864985	3.6982764	0.9773312
H	-1.5764334	3.9103409	0.0904897

References

(1) Cankařová, N.; Krchňák, V. Polymer-Supported Stereoselective Synthesis of Benzimidazolinopiperazinones. *J. Org. Chem.* **2012**, *77* (13), 5687–5695. https://doi.org/10.1021/jo300836c.

(2) Wavefunction Inc. Spartan'24, Version 1.2.0. Wavefunction Inc: Irvine, CA 2024.

(3) Weigend, F.; Ahlrichs, R. Balanced Basis Sets of Split Valence, Triple Zeta Valence and Quadruple Zeta Valence Quality for H to Rn: Design and Assessment of Accuracy. *Phys. Chem. Chem. Phys.* **2005**, *7* (18), 3297–3305. https://doi.org/10.1039/B508541A.

(4) Grimme, S.; Antony, J.; Ehrlich, S.; Krieg, H. A Consistent and Accurate Ab Initio Parametrization of Density Functional Dispersion Correction (DFT-D) for the 94 Elements H-Pu. *J. Chem. Phys.* **2010**, *132* (15), 154104. https://doi.org/10.1063/1.3382344.

(5) Grimme, S.; Ehrlich, S.; Goerigk, L. Effect of the Damping Function in Dispersion Corrected Density Functional Theory. *J. Comput. Chem.* **2011**, *32* (7), 1456–1465. https://doi.org/https://doi.org/10.1002/jcc.21759.

(6) University of Karlsruhe; Forschungszentrum Karlsruhe GmbH; TURBOMOLE GmbH. TURBOMOLE, Version 7.2. TURBOMOLE GmbH 2017.

(7) Ahlrichs, R.; Bär, M.; Häser, M.; Horn, H.; Kölmel, C. Electronic Structure Calculations on Workstation Computers: The Program System Turbomole. *Chem. Phys. Lett.* **1989**, *162* (3), 165–169. https://doi.org/https://doi.org/10.1016/0009-2614(89)85118-8.

(8) Klamt, A.; Schüürmann, G. COSMO: A New Approach to Dielectric Screening in Solvents with Explicit Expressions for the Screening Energy and Its Gradient. *J. Chem. Soc. Perkin Trans. 2* **1993**, No. 5, 799–805. https://doi.org/10.1039/P29930000799.

(9) Klamt, A. Conductor-like Screening Model for Real Solvents: A New Approach to the Quantitative Calculation of Solvation Phenomena. *J. Phys. Chem.* **1995**, *99* (7), 2224–2235. https://doi.org/10.1021/j100007a062.

(10) Gerlach, T.; Müller, S.; de Castilla, A. G.; Smirnova, I. An Open Source COSMO-RS Implementation and Parameterization Supporting the Efficient Implementation of Multiple Segment Descriptors. *Fluid Phase Equilib.* **2022**, *560*, 113472. https://doi.org/https://doi.org/10.1016/j.fluid.2022.113472.

(11) Müller, S.; Nevolianis, T.; Garcia-Ratés, M.; Riplinger, C.; Leonhard, K.; Smirnova, I. Predicting Solvation Free Energies for Neutral Molecules in Any Solvent with OpenCOSMO-RS. *Fluid Phase Equilib.* **2025**, *589*, 114250. https://doi.org/https://doi.org/10.1016/j.fluid.2024.114250.

(12) Neese, F. The ORCA Program System. *WIREs Comput. Mol. Sci.* **2012**, *2* (1), 73–78. https://doi.org/https://doi.org/10.1002/wcms.81.

(13) Neese, F. Software Update: The ORCA Program System, Version 4.0. *WIREs Comput. Mol. Sci.* **2018**, *8* (1), e1327. https://doi.org/https://doi.org/10.1002/wcms.1327.

(14) Neese, F.; Wennmohs, F.; Becker, U.; Riplinger, C. The ORCA Quantum Chemistry Program Package. *J. Chem. Phys.* **2020**, *152* (22), 224108. https://doi.org/10.1063/5.0004608.
